# Pd(ii)-catalyzed synthesis of bifunctionalized carboranes *via* cage B–H activation of 1-CH_2_NH_2_-*o*-carboranes†
†Electronic supplementary information (ESI) available: Experimental details, compound characterization and X-ray data in CIF format for **1a**, **3**, **4**, **6**, **10**, **17**, **28** and **34**. CCDC 1583775–1583782. For ESI and crystallographic data in CIF or other electronic format see DOI: 10.1039/c8sc01154k


**DOI:** 10.1039/c8sc01154k

**Published:** 2018-03-27

**Authors:** Xiaolei Zhang, Hong Yan

**Affiliations:** a School of Pharmaceutical Sciences , Jiangnan University , Wuxi , Jiangsu 214122 , P. R. China . Email: xlzhang@jiangnan.edu.cn; b State Key Laboratory of Coordination Chemistry , School of Chemistry and Chemical Engineering , Nanjing University , Nanjing , Jiangsu 210093 , P. R. China . Email: hyan1965@nju.edu.cn

## Abstract

Aminoalkyl carboranes are anticipated to be valuable synthons toward the synthesis of bifunctionalized carboranes. However, direct cage boron derivation of these carborane derivatives has not been solved. Herein, the reversible conversion of catalytically infeasible *o*-carboranyl methylamines (1-CH_2_NH_2_-*o*-carboranes) into bidentate imines initiates Pd-mediated cage B–H activation. As a result, an amine coordinated bicyclic Pd(ii) complex (**3**) has been isolated and proven to be the catalytically active intermediate for highly site-selective B–H diarylation of *o*-carboranyl methylamines. Using glyoxylic acid as an inexpensive and commercially available transient directing reagent, a wide range of cage B(4,5)-diarylated free primary *o*-carboranyl methylamines were prepared in good to excellent yields with the avoidance of the pre-installation and removal of a directing group. This method provides easy access to cage boron functionalized *o*-carboranyl methylamines with potential for application in pharmaceuticals.

## Abstract 2

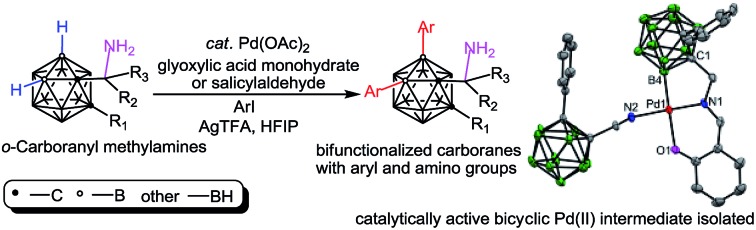
Pd-catalyzed, transient group-directed diarylation of cage B(4,5)–H bonds in 1-CH_2_NH_2_-*o*-carboranes was reported, including the isolation and structural characterization of key intermediates.

## Introduction

Polyhedral carboranes^1^ have found broad applications in biomedicine,^2^ materials^3^ and catalysis^4^ owing to their unique structural and chemical properties. Hydrophobic carborane clusters having covalent linkage with dissimilar polar functional groups (*e.g.* –OH, –NH_2_) may be water-soluble and serve as useful synthons for attachment to tumor seekers such as porphyrins^5^ or nucleic acids.6*a* Hence, aminoalkyl carboranes, which are also termed as carboranyl alkylamines, have attracted considerable interest due to their potential use in boron neutron capture therapy (BNCT).^6^ Additionally, the structural rigidity and three dimensional spatial features of the aminoalkyl carboranes can be put into promising use as enzyme inhibitors.^7^ This resurgence of interest has created a requirement for bifunctionalized carboranes bearing two different functional groups in one molecule. While cage B–H functionalization of aminoalkyl carboranes is highly desirable, as it enables rapid cage boron derivations toward bifunctionalized carboranes, until now, direct functionalization of the cage B–H bond in aminoalkyl carboranes remains unsolved. The apparent difficulties that cause these carboranes to be incompatible with B–H functionalization processes include (a) the vulnerability of the amine group to harsh B–H activation conditions such as oxidants8a,b or electrophiles,8c,d and (b) the strong binding ability^9^ of the amine group to a metal center leads to an unreactive bis(amine)metal complex for transition metal-promoted cage B–H activation.^10^,^11^

**Scheme 1:**
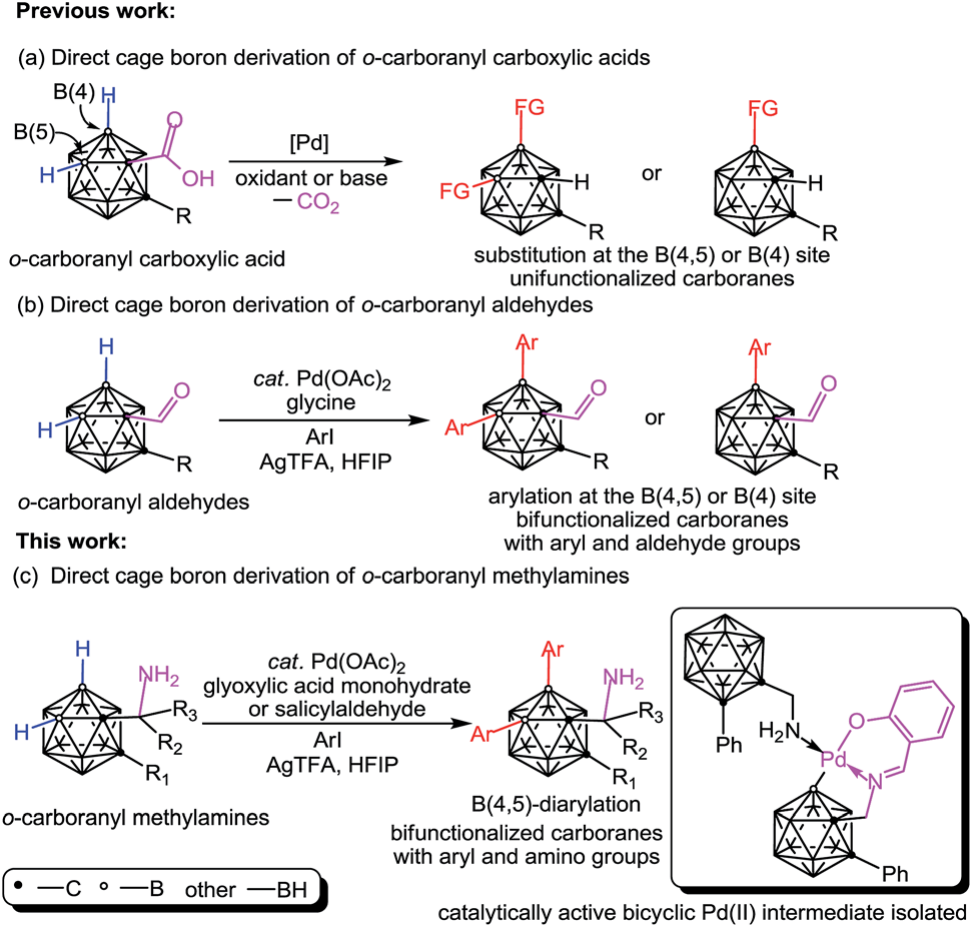
Direct cage boron derivation of *o*-carborane leading to unifunctionalized or bifunctionalized carboranes.

Inspired by the aforementioned challenges, here we report an efficient Pd-catalyzed cage B–H functionalization of aminomethyl-*o*-carboranes (also termed as *o*-carboranyl methylamines and 1-CH_2_NH_2_-*o*-carboranes) by using glyoxylic acid or salicylaldehyde as the catalytic and transient directing groups. A wide array of B(4,5)-diarylated *o*-carboranyl methylamines were prepared as free primary amines without protection or deprotection steps. In addition, a bicyclic palladium complex (**3**) has been isolated featuring *o*-carboranyl methylamine as an internal ligand and proven to be the catalytically active intermediate. Importantly, direct C–H functionalization of organic amines has been realized by using a transient directing strategy^12^ or a steric tethering approach;^13^ cage B–H functionalization of *o*-carboranyl carboxylic acids has been reported by Xie and co-workers,11*k* where the carboxylic acid group serves as a traceless directing group (DG) (Scheme 1a); *o*-carboranyl aldehydes have also been utilized, by us,11*l* as suitable substrates for cage boron derivation with a transient DG (Scheme 1b). Our present work (Scheme 1c) constitutes the first study of cage B–H functionalization of aminoalkyl carboranes, which affords bifunctionalized carboranes with both aryl and amino groups in one molecule.

## Results and discussion

**Scheme 2:**
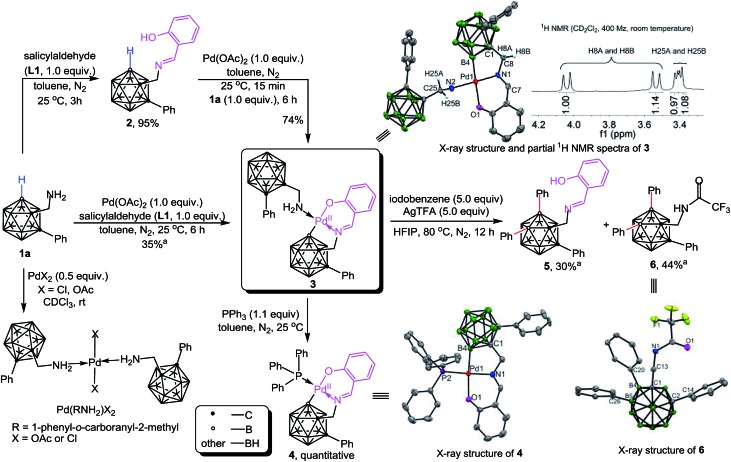
Synthesis of bicyclic palladium complex **3** through palladium-mediated B–H activation and its further transformations including ligand exchange and subsequent diarylation. ^a^Isolated yields based on *o*-carboranyl methylamine.

Initial studies involved the investigation of Pd-mediated selective B–H activation of 1-phenyl-*o*-carboranyl-2-methylamine **1a**. Treatment of **1a** with PdX_2_ (X = Cl or OAc) led to bis(amine)Pd(ii) complexes which was characterized (Schemes 2 and S4†) and found to be unreactive for the cage B–H activation process. Three component reaction of **1a** with salicylaldehyde (**L1**) and Pd(OAc)_2_ in toluene at 25 °C afforded a new palladium complex **3** in 35% yield (Scheme 2). Alternatively, condensation of **1a** with **L1** delivered *o*-carboranyl methylaldimine **2**, followed by sequential treatment with 1.0 equiv. of Pd(OAc)_2_ and **1a** also gave rise to **3**. In solution, as indicated by the ^1^H NMR spectrum, **3** exhibits typically two types of CH_2_ signals with the coupling of homocarbon hydrogens for each (Scheme 2, *δ* = 4.04 and 3.53 ppm for H8A and H8B, *J*_H–H_ = 15 Hz; *δ* = 3.42 and 3.40 ppm for H25A and H25B, *J*_H–H_ = 6 Hz). These results shed light on the ring annulation to form a five-membered B–C–C–N–Pd palladacycle after B–H activation, which is consistent with a single-crystal X-ray analysis in the solid state. Interestingly, here *o*-carboranyl methylamine **1a** plays a dual role in Pd-mediated B–H activation: (1) it acts as a substrate to form a 5,6-fused palladacycle through Pd–B bond formation (B4–Pd1 2.009(14) Å), and (2) it acts as a ligand to furnish a four coordinated Pd(ii) core with a square planar configuration.

In the current reaction, cage B–H activation was initiated by the dehydration of **1a** with salicylaldehyde to produce a bidentate imine/hydroxyl directing group, followed by treatment with Pd(ii) acetate to furnish a proximity-driven B–H palladation process. It is noteworthy that there have been pioneer reports in which some organic imines are utilized as transient directing groups for direct C–H bond functionalization of primary amines.^12^ However, reported examples of such a mechanism for inert C–H activation are proposed based on indirect evidence where the model intermediates have been isolated with the aid of an external ligand.12a,c Herein, complex **3** is formed with the aid of an internal ligand and it can mimic the real catalytic reaction conditions in which excess substrate (*o*-carboranyl methylamine) is present.

Complex **3** is reactive for further transformations. For example, ligand exchange of **3** with PPh_3_ delivered **4** in quantitative yield. Stoichiometric reaction of **3** with 5.0 equiv. of iodobenzene and AgTFA in HFIP at 80 °C afforded two B(4,5)-diarylated species which were identified as salicylaldimine **5** and trifluoroacetamide **6** in moderate yields. Compound **6** could be converted to its free amine **7** under basic and moisture conditions. Compounds **5–7** were fully characterized by NMR spectroscopy, infrared spectroscopy (IR) and high resolution mass spectrometry (HRMS). The structure of **6** was further confirmed by single-crystal X-ray diffraction analysis (Scheme 2).

**Scheme 3:**
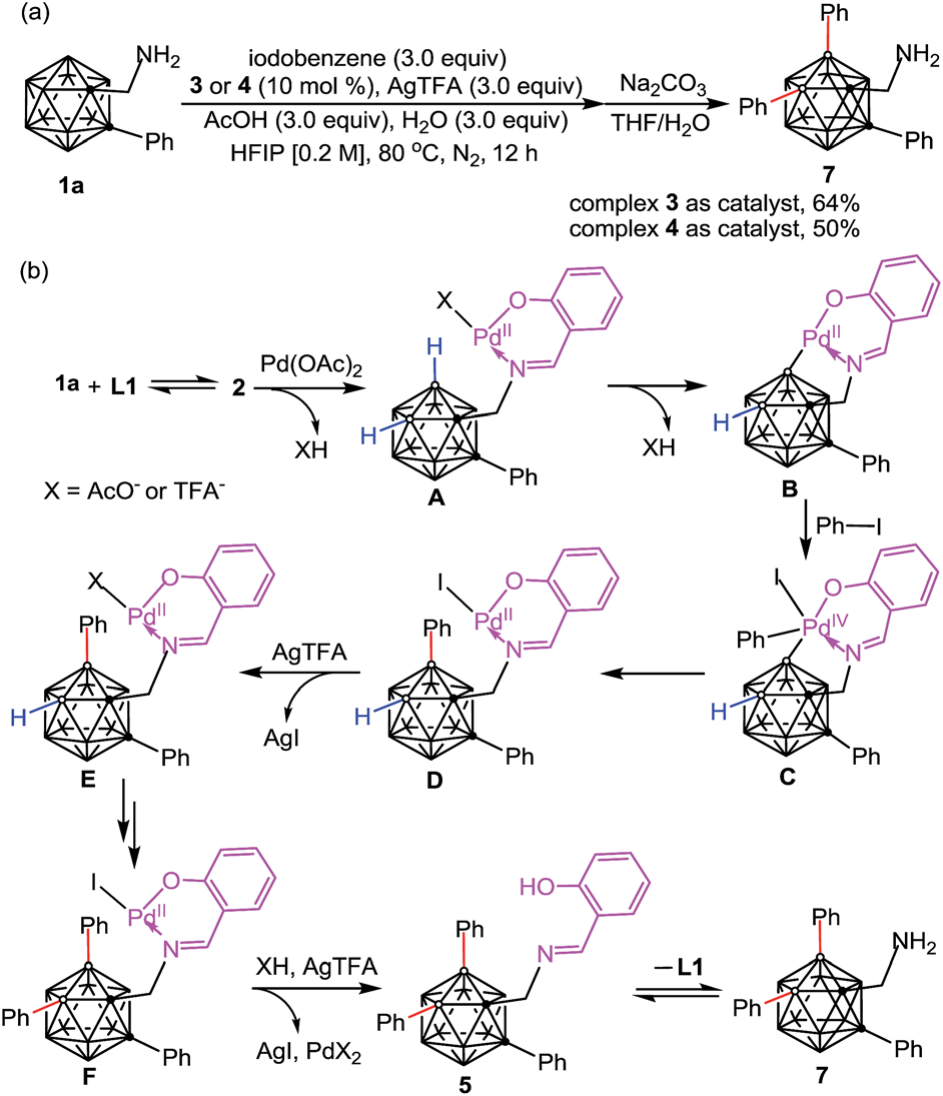
(a) Catalytic performance of **3** and **4** in cage B–H diarylation. (b) A plausible reaction mechanism.

The catalytic performance of **3** and **4** has been evaluated. We found that the addition of 3.0 equiv. of AcOH and H_2_O for each was essential for catalytic reactions (Table S1†). Using complex **3** as a catalyst (10 mol%) under catalytic conditions (Scheme 3a) afforded B–H diarylated product **7** in 64% yield. Thus, complex **3** is most likely involved in the catalytic cycle. Interestingly, complex **4**, a PPh_3_ adduct, also exhibited catalytic activity, albeit with decreased efficiency. On the basis of the above results a proposed reaction pathway for the formation of the above-mentioned species is depicted in Scheme 3b. Dehydration between **1a** and **L1** forms **2** which can subsequently replace the acetate of Pd(OAc)_2_ to afford **A**. Then, B–H activation occurs at the B(4) site to yield a bicyclic palladium intermediate **B**. Oxidative addition of **B** with 1.0 equiv. of PhI affords a Pd(iv) intermediate **C**, followed by reductive elimination to generate **D**. Then iodide abstraction with AgTFA leads to **E**, before the repetition of a similar tandem sequence at the B(5) site to deliver **F**. Iodide abstraction and protonation gives rise to B(4,5)-diarylated salicyladimine **5**, followed by hydrolysis to furnish the free amine **7** with release of the Pd catalyst and salicylaldehyde. It is noteworthy that *o*-carboranyl methylamines (**1a** or **7**) can stabilize the Pd-involved intermediates through H_2_N → Pd coordination, for example, the combination of **1a** and **B** can produce **3**.

Considering the importance of a directing ligand for selective cage B–H activation, we continued our investigation of palladium-catalyzed B–H diarylation of **1a** with iodobenzene with focus on ligand screening (Table 1). Gratifyingly, either salicylaldehyde (**L1**) or 3,5-di-*tert*-butylsalicylaldehyde (**L3**) can afford the desired product **7** in 70% and 67% yields, respectively. Utilizing 3,5-dichloro-salicylaldehyde (**L2**) with electron-withdrawing groups reduced the yield. Pyridine-based ligands (**L4** and **L5**) led to decomposition of **1a**. The reaction could be performed with glyoxylic acid monohydrate (**L6**) in 85% yield whereas the yield was decreased with 2-oxopropanoic (**L7**) or phenylglyoxylic acid (**L8**), indicating the importance of an aldehyde moiety. In the absence of ligands, no reaction was observed, demonstrating the necessity of a transient DG for B–H activation (Table S1,† entry 8). Neither benzaldehyde (**L9**) nor butyraldehyde (**L10**) was reactive, which again shows the importance of bidentate chelation of imine and hydroxyl moieties.

**Table 1:** Evaluation of the transient DGs^*a*^ ^,^^*b*^

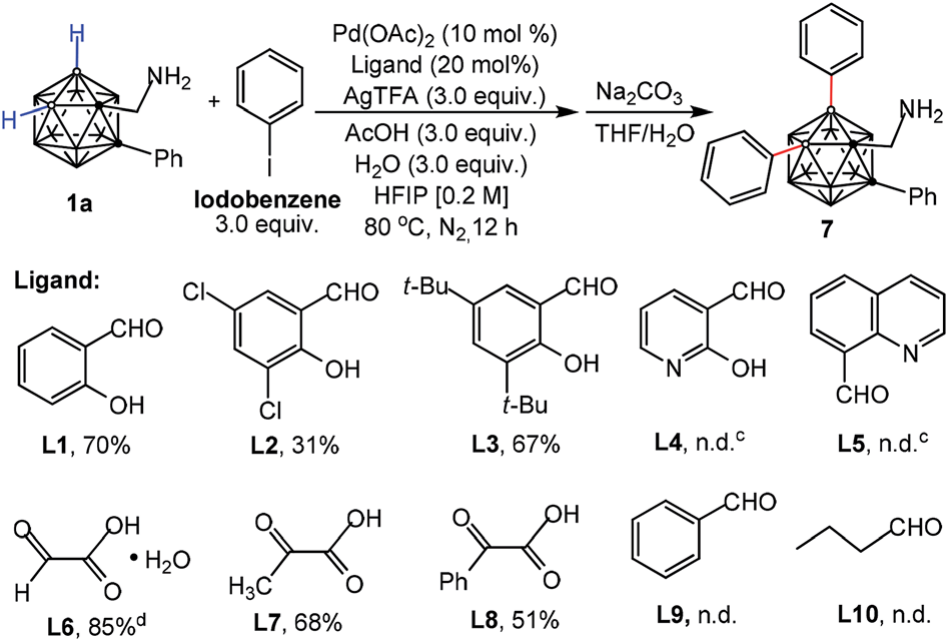

^*a*^Conditions: **1a** (0.1 mmol), iodobenzene (0.3 mmol), Pd(OAc)_2_ (10 mol%), ligands (20 mol%), AgTFA (0.3 mmol), AcOH (0.3 mmol), H_2_O (0.3 mmol), HFIP (0.5 mL), 80 °C, N_2_ atmosphere, 12 h.

^*b*^Isolated yield.

^*c*^ **1a** was decomposed.

^*d*^Optimal conditions. n.d.: desired product not detected.

**Fig. 1:**
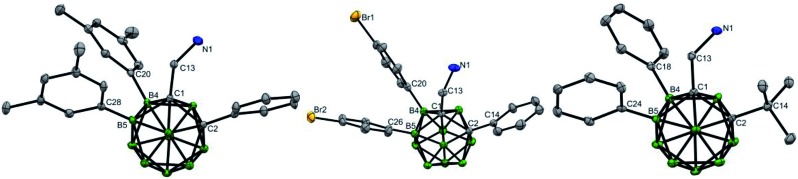
Molecular structures of **10** (left), **17** (middle) and **28** (right) (ellipsoids at 30% probability and H atoms partially omitted for clarity). Selected bond distances [Å] for **10**: C1–C2 1.694(4), B4–C20 1.569(4), B5–C28 1.578(4), C1–C13 1.530(4), and C13–N1 1.448(4). For **17**: C1–C2 1.698(2), B4–C20 1.586(3), B5–C26 1.586(2), C1–C13 1.537(2), and C13–N1 1.441(2). For **28**: C1–C2 1.743(3), B4–C18 1.585(3), B5–C24 1.583(3), C1–C13 1.530(3), and C13–N1 1.459(3).

The scope of the aryl iodides and *o*-carboranyl methylamines was further investigated by the use of the cheap DG (**L6**) (Table 2). Alkyl, phenyl, alkoxy, trifluoromethyl, alkoxylcarbonyl and acetyl substituted aryl iodides were well tolerated in this process, furnishing the B(4,5)-diarylated *o*-carboranyl methylamines (**7–14** and **19–22**) in good to excellent yields with high site-selectivity (Table 2). Halogenated (fluoro, chloro or bromo) aryl iodides were also found to be viable (**15–18**). Unfortunately, B(4,5)-diarylation for *ortho*-iodotoluene was not compatible under the current conditions, presumably due to the steric effect of the 2-tolyl group. In addition, C-aryl, -alkyl or -α-methyl substituted *o*-carboranyl methylamines were effective substrates, providing the corresponding B(4,5)-diarylated products in synthetically satisfactory yields (**23–29**). However, 1-CH_2_NH_2_-*o*-C_2_B_10_H_11_ without a substituent at the carbon vertex afforded an inseparable mixture. These observations suggested that the substitution at the carbon site can contribute to B(4)/B(5)-selectivity, which was consistent with our previous reports.11*l* The *α*-dimethyl substituent also gave rise to B(4,5)-diarylated *o*-carborane (**30**) with an *in situ* removal of the methylamine group *via* C_cage_–C bond cleavage. After treatment with Na_2_CO_3_ in THF/H_2_O, non-protected primary amines could be obtained without the need for further tactics to remove the DGs. All of the new compounds (**7–29**) were fully characterized by ^1^H, ^11^B, and ^13^C NMR spectroscopy, IR and HRMS. The structures of **10**, **17** and **28** were further confirmed by single-crystal X-ray diffraction analysis (Fig. 1).

**Table 2:** Substrate scope^*a*^ ^,^^*b*^

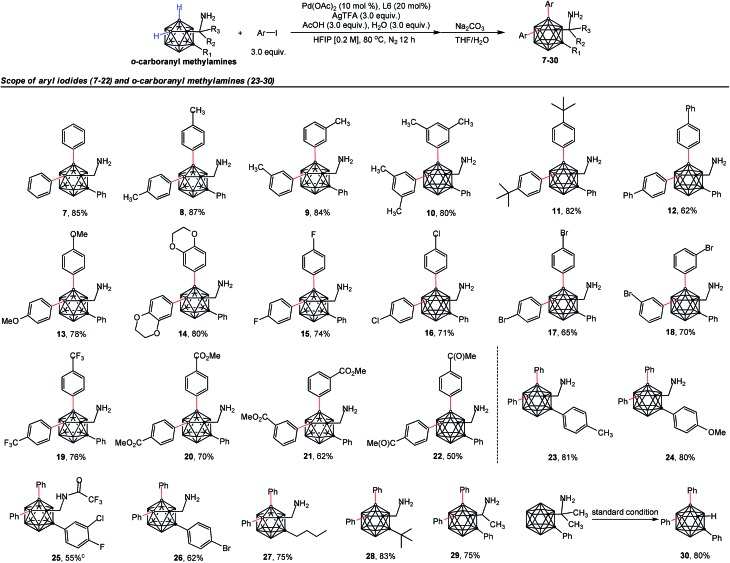

^*a*^Reaction conditions: *o*-carboranyl methylamines (0.1 mmol), Ar–I (3.0 equiv., 0.3 mmol), Pd(OAc)_2_ (10 mol%, 0.01 mmol), glyoxylic acid monohydrate (**L6**, 20 mol%, 0.02 mmol), AgTFA (3.0 equiv., 0.3 mmol), AcOH (3.0 equiv., 0.3 mmol), H_2_O (3.0 equiv., 0.3 mmol), HFIP (0.2 M, 0.5 mL), 80 °C, N_2_ atmosphere, 12 h.

^*b*^Isolated yield.

^*c*^Isolated without treatment with Na_2_CO_3_ in THF/H_2_O.

**Scheme 4:**
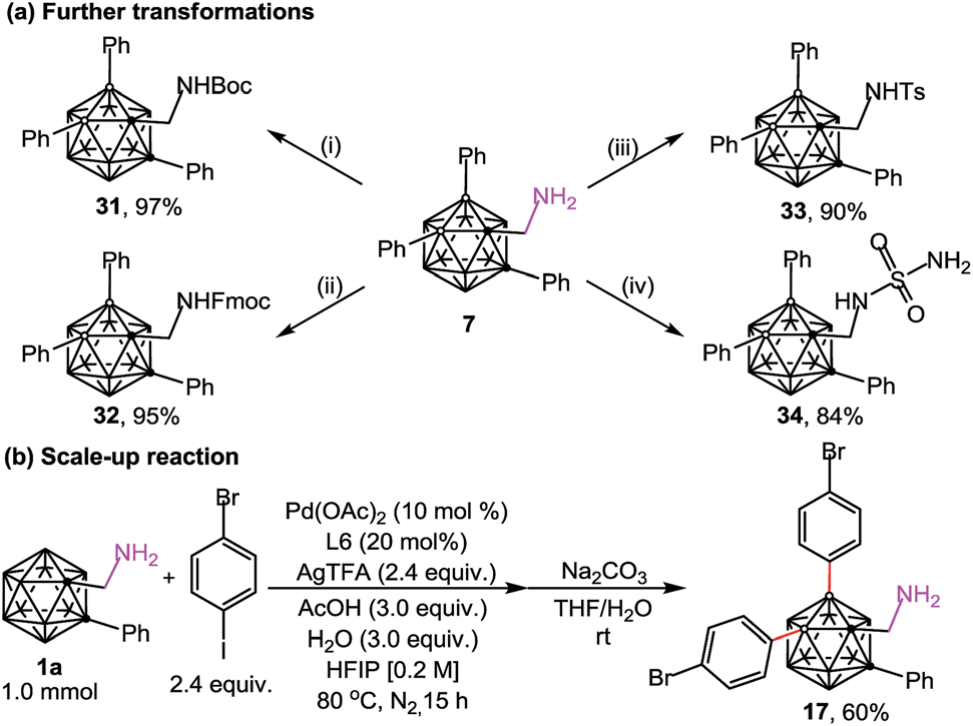
Further transformations and scale up reaction. Reaction conditions: (i) (Boc)_2_O (4.0 equiv.), Et_3_N (4.0 equiv.), CH_2_Cl_2_, rt; (ii) Fmoc-Osu (4.0 equiv.), CH_3_CN, rt; (iii) 4-toluene sulfonyl chloride (2.0 equiv.), Na_2_CO_3_ (2.0 equiv.), THF/H_2_O, rt; and (iv) sulfamide (3.0 equiv.), Na_2_CO_3_ (2.0 equiv.), 1,4-dioxane, 100 °C.

**Fig. 2:**
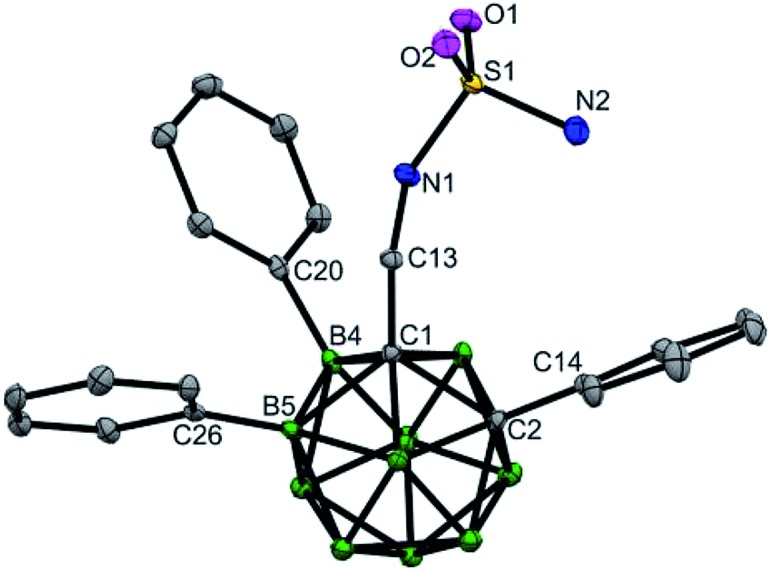
Molecular structures of **34** (ellipsoids at 30% probability and H atoms omitted for clarity). Selected bond distances [Å]: C1–C2 1.723(4), B4–C20 1.579(4), B5–C26 1.571(4), C1–C13 1.529(3), C13–N1 1.452(3), S1–N1 1.610(2), and S1–N2 1.592(3).

The substrates (aminomethyl-*o*-carboranes) used in this study are stable under the current catalytic conditions and can be readily transformed into other derivatives. As demonstrated in Scheme 4a, B(4,5)-diarylated *o*-carboranyl methylamine **7** could be readily converted to its Boc, Fmoc or tosyl amide derivatives (**31–33**) in excellent yields. In addition, attachment of the sulfamide moiety by using transamination between **7** and sulfamide gave rise to **34** in 84% yield (Scheme 4a). Since three-dimensional carboranyl sulfamides are promising inhibitors for carbonic anhydrase isozymes,7*a* the cage B–H activation strategy on carboranyl methylamines would be beneficial to the structure-based design of these specific inhibitors. The structure of **34** was determined by X-ray diffraction analysis (Fig. 2). Furthermore, when the reaction of **1a** and 4-bromo-1-iodobenzene was scaled up to 1.0 mmol, the B(4,5)-diarylated product **17** was isolated in 60% yield after silica chromatography (Scheme 4b).

## Conclusions

In conclusion, we have developed aminomethyl-*o*-carboranes as ideal candidates for cage B–H activation reaction. This reaction demonstrates high site-selectivity for B(4,5)-diarylation at the carboranyl unit, as well as good functional group compatibility. In the presence of salicylaldehyde, the bidentate nature of the *in situ* generated imine-hydroxyl ligand favours the formation of a bicyclic palladium complex (**3**) featuring cage B–H activation at the B(4) site. With *o*-carboranyl methylamine as an internal ligand, a bicyclic palladium complex (**3**) has been isolated and proven to be the catalytically active intermediate for catalytic B–H diarylation. Through the use of glyoxylic acid as an inexpensive and transient directing agent, a series of B(4,5)-diarylated free primary *o*-carboranyl methylamines were obtained without further tactics to install and remove the DGs. Considering the importance of aminoalkyl carboranes in biological systems, the methodology reported here will be beneficial to the synthesis of bifunctionalized carboranes for drug discovery.

## Conflicts of interest

There are no conflicts to declare.

## Supplementary Material

Supplementary information

Crystal structure data
