# Supplementary material for: Pd(ii)-catalyzed synthesis of bifunctionalized carboranes via cage B–H activation of 1-CH2NH2-o-carboranes
Source: Chem Sci. 2018 Mar 27;9(16):3964–9. doi: 10.1039/c8sc01154k (PMC5941203; doi:10.1039/c8sc01154k)
Supplement: Supplementary file 1 [file SC-009-C8SC01154K-s001.pdf]

Electronic Supplementary Material (ESI) for Chemical Science.
This journal is © The Royal Society of Chemistry 2018

Supplementary material

Pd(II)-Catalyzed Synthesis of Bifunctionalized Carboranes via Cage B−H Activation of 1-CH_2NH_2-*o*-carboranes

Xiaolei Zhang[a]*, Hong Yan[b]*

[a]School of Pharmaceutical Sciences, Jiangnan University, Wuxi, Jiangsu 214122, P. R. China
[b]State Key Laboratory of Coordination Chemistry, School of Chemistry and Chemical Engineering, Nanjing University, Nanjing, Jiangsu 210093, P. R. China

Corresponding Author
*Email: xlzhang@jiangnan.edu.cn
*Email: hyan1965@nju.edu.cn

Table of contents

1. General Considerations………………………………………………S2-S3

2. Experimental Section……………………………………………..S3-S42

3. References………………………………. ……………… S43-S43

4. Crystallographic data…………………………..……...…S43-S44

5. NMR Spectra……………………………………..……S45-S136

1. General Considerations

C-monoarylated *o*-carboranes[S1–S2], C-monoalkylated *o*-carboranes[S3], *o*-carboranyl methylamine **1f**[S4–S5] and phthalimide protected propargylamines[S6] were synthesized according to literature methods. The process for the synthesis of *o*-Carboranyl methylamine **1a** has been optimized according to literature report.[S5, S7] Unless otherwise noted, all the solvents and commercially available reagents were purchased from commercial sources and used directly. Hexanes, THF, TBME (tert-butyl methyl ether) and Et_2O were refluxed and distilled over sodium/benzophenone under nitrogen. CH_2Cl_2 and toluene were refluxed and distilled over GaH_2 under nitrogen. Glass-backed Silica Gel 60 thin-layer chromatography (TLC) plates were used as received. Column chromatography was performed on Silica Gel 60 (200–300 or 300–400 Mesh). TLC samples for carborane-containing compounds were stained with 1 wt. % $PdCl_2$ in 6 M HCl and were developed with high heat using a heat gun.

Spectroscopic Measurements. ^1H, ^{11}B, $^{11}B\{^1H\}$, $^{13}C\{^1H\}$, ^{19}F, ^{31}P spectra were recorded using Bruker DRX 400 MHz NMR spectrometers in ambient conditions unless otherwise stated. All chemical shifts were reported in δ units with references to the residual solvent resonances of the deuterated solvents for proton and carbon chemical shifts. Note that H_2O resonances are often present due to high humidity. ^{11}B chemical shifts were measured utilizing external $Et_2O \cdot BF_3$ ($\delta^{11}B$ = 0 ppm) as reference. ^{19}F NMR spectra were referenced to fluorobenzene (δ = –113.15). ^{31}P spectra were referenced to external 85% phosphoric acid (δ = 0 ppm). The high resolution mass spectra (HRMS) were recorded on Waters (Micromass) GCT-Premier for ESI-MS. IR spectrums were recorded on Thermo Scientific Nicolet IS 50 Fourier transform infrared spectrometer

X-ray Crystallography. X-ray diffraction data of **1a**, **3**, **4**, **6**, **10**, **17, 28** and **34** (CCDC No. 1583775-1583782) were collected on a Bruker SMART Apex II CCD diffractometer or Bruker D8 Quest diffractometer by means of graphitemonochromated Mo Kα radiation at 291 K. During the collection of the

intensity data, no significant decay was observed. The intensities were corrected for Lorentz polarization effects and empirical absorption by using the SADABS program.[S8] The structures were solved by direct methods with the SHELXL-97 program.[S9] All non-hydrogen atom positions were determined utilizing the difference Fourier synthesis. The hydrogen atoms were placed at geometrically calculated positions, which were refined using a riding model. All calculations were performed by applying the Bruker SMART program.

2. Experimental Section

2.1 Substrate preparation

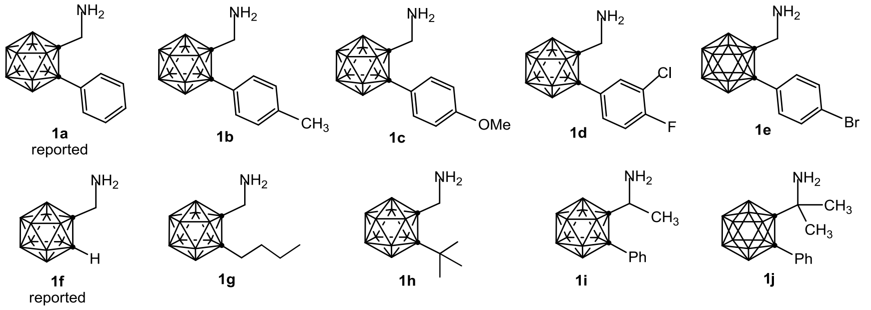

Scheme S1. Schematic presentation for the *o*-carboranyl methylamines **1a-1j** used in this work. **1a** and **1f** were previously reported and the others are new.

2.1.1 Preparation of *o*-carboranyl methylamines (1a-1c, 1g-1h)-method 1:

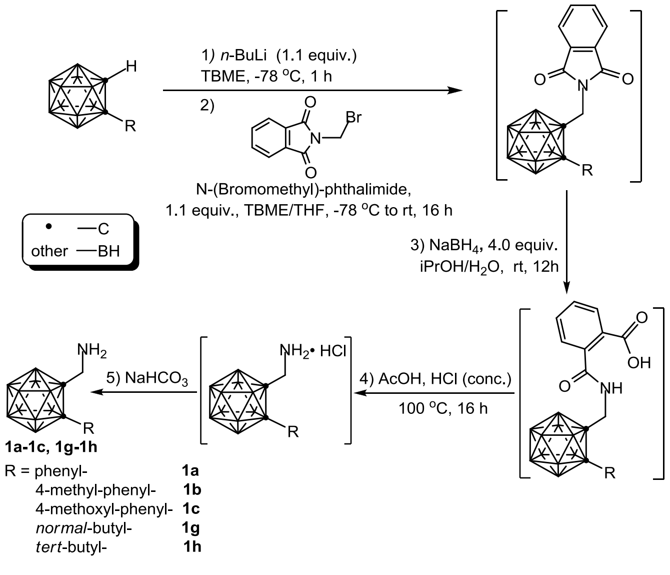

Scheme S2. Synthetic procedure of *o*-carboranyl methylamines **1a-1c, 1g-1h**.

Synthesis of phthalimide protected *o*-carboranyl methylamine: To a 100 mL Schlenk bottom were added the appropriate C-monosubstituted *o*-carborane (3.0 mmol), *tert*-butyl methyl ether (TBME, 10 mL) and cooled to –78 ^{o}C under N_2 atmosphere. ^{n}BuLi (1.1 equiv., 1.6 M in hexane, 2.1 mL) was slowly added into the mixture and the resulting solution was stirred for 1 h at –78 ^{o}C with a magnetic stir bar. Then THF (10 mL) was added as a co-solvent. N-(Bromomethyl)-phthalimide (1.1 equiv., 792 mg) was added in one portion at –78 ^{o}C under the protection of N_2. The reaction mixture was slowly warmed to room temperature and stirred for 16 h. Upon completion, the reaction was quenched with H_2O (5 mL) and concentrated under *vacuo* to obtain a crude product of the phthalimide protected *o*-carboranyl methylamine, which was directly used in subsequent step.

Deprotection: To the 100 mL Schlenk bottom containing the crude product were added iPrOH (10 ml) and H_2O (2 ml). Then $NaBH_4$ (4.0 equiv., 454 mg) was added slowly in portions under N_2 atmosphere. The mixture was stirred at room temperature for 12 h. Upon completion, diluted HCl (1 N) solution was slowly added to quench the unreacted $NaBH_4$. The solvents were removed under *vacuo*. Then, acetic acid (10 mL) and concentrated HCl (35%, 2 mL) were added and the resulting solution was heated for 16 h at 100 ^{o}C. The volatiles were removed under *vacuo*, water (20 mL) was added and the crude product was extracted with Et_2O or THF (10 mL × 3, THF with saturated brine). The combined organic extracts were evaporated under *vacuo*. The hydrochloride salts of the related *o*-carboranyl methylamine (**1a-1c, 1g-1h**) could be purified through chromatography on a silica gel column using hexanes/THF as an eluent. Alternatively, they can be recrystallized using CH_2Cl_2 (for **1a•HCl** and **1b•HCl**), hexanes/EtOAc (for **1c•HCl**) or hexanes/CH_2Cl_2 (for **1g•HCl** and **1h•HCl**).

Basification: The purified hydrochloride salt of the *o*-carboranyl methylamine was added into a 100 mL Schlenk bottom containing CH_2Cl_2/H_2O (20 mL/20 mL). Then, $NaHCO_3$ (excess, 9.0 mmol, 750 mg) was added and the mixture was stirred for 12 h at room temperature. Upon completion, the organic phase was collected and the water

phase was extracted with CH_2Cl_2 (15 mL × 3). The combined organic extracts were dried with anhydrous Na_2SO_4, filtrated and concentrated under *vacuo* to obtain the *o*-carboranyl methylamine (**1a-1c, 1g-1h**).

Note: Single crystal of **1a** suitable for X-ray analysis was obtained by slow evaporation of a hexane/CH_2Cl_2 solution of **1a** at room temperature.

2.1.2 Practical synthesis of 1a.

To a 100 mL Schlenk bottom were added 1-phenyl-*o*-carborane (9.0 mmol, 1.98 g), *tert*-butyl methyl ether (TBME, 30 mL) and cooled to –78 ^{o}C under N_2 atmosphere. ^{n}BuLi (1.1 equiv., 1.6 M in hexane, 6.2 mL) was slowly added into the mixture and the resulting solution was stirred for 1 h at –78 ^{o}C with a magnetic stir bar. Then THF (30 mL) was added as a co-solvent. N-(Bromomethyl)-phthalimide (1.1 equiv., 2.4 g) was added in one portion at –78 ^{o}C under the protection of N_2. The reaction mixture was slowly warmed to room temperature and stirred for 20 h. Upon completion, the reaction was quenched with H_2O (10 mL) and concentrated under *vacuo* to obtain a crude product of the phthalimide protected *o*-carboranyl methylamine, which was directly used in subsequent step.

Deprotection: To the 100 mL Schlenk bottom containing the crude product were added iPrOH (30 ml) and H_2O (5 ml). Then $NaBH_4$ (4.0 equiv., 1.4 g) was added slowly in portions under N_2 atmosphere. The mixture was stirred at room temperature for 12 h. Upon completion, diluted HCl (1 N) solution was slowly added to quench the unreacted $NaBH_4$. The solvents were removed under *vacuo*. Then, acetic acid (30 mL) and concentrated HCl (35%, 5 mL) were added and the resulting solution was heated for 16 h at 100 ^{o}C. The volatiles were removed under *vacuo*, water (30 mL) was added and the crude product was extracted with THF (20 mL × 3, THF with saturated brine). The combined organic extracts were evaporated to dryness under *vacuo*. The hydrochloride salts of the **1a** can be recrystallized from CH_2Cl_2 (25 mL) at 25 ^{o}C and filtrated to obtain a white cake.

Note: 1-phenyl-*o*-carborane (*ca.* 500 mg) can be recovered from the filtrate by column chramotography using hexanes as the eluent.

Basification: The purified hydrochloride salt of **1a** was added into a 100 mL Schlenk bottom containing CH_2Cl_2/H_2O (30 mL/30 mL) to form a white slurry. Then, $NaHCO_3$ (excess, 24 mmol, 2.0 g) was added and the mixture was stirred for 12 h at room temperature until the white slurry disappeared to form a clear solution. Upon completion, the organic phase was collected and the water phase was extracted with CH_2Cl_2 (15 mL × 3). The combined organic extracts were dried with anhydrous Na_2SO_4, filtrated and concentrated under *vacuo* to obtain **1a** (1.00-1.12 g, yield, 45-50%).

2.1.3 Preparation of *o*-carboranyl methylamines (1d-1e, 1i-1j)-method 2:

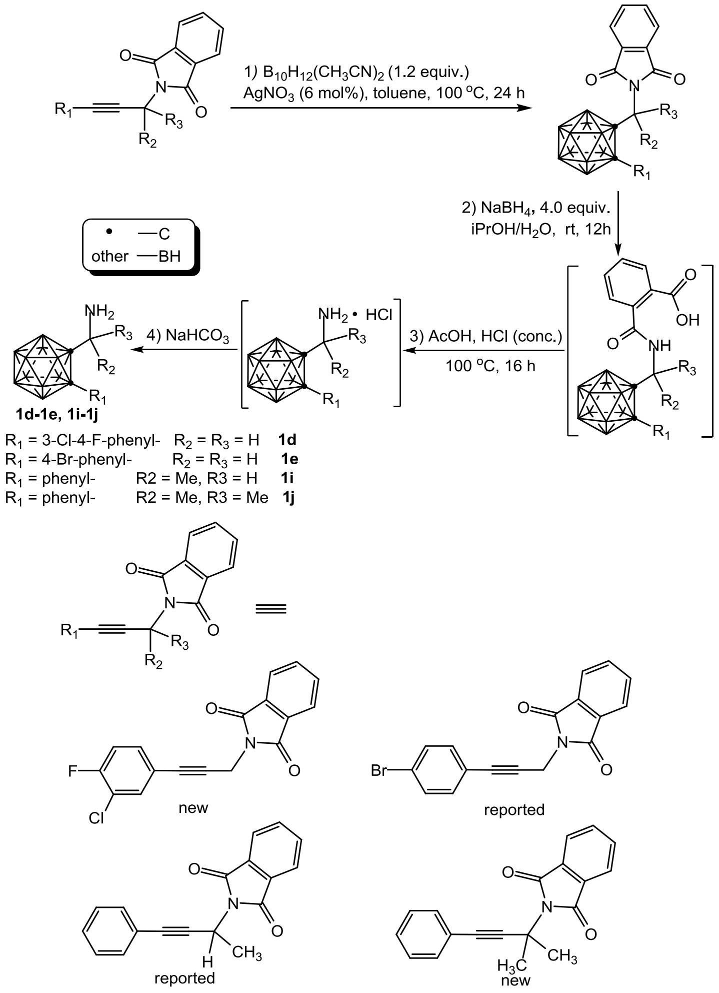

Scheme S3. Synthetic procedure of *o*-carboranyl methylamines **1d-1e, 1i-1j**.

Synthesis of phthalimide protected *o*-carboranyl methylamine: To a 100 mL Schlenk bottom were added the appropriate phthalimide protected propargylamines (2.0 mmol), $B_{10}H_{12}(CH_3CN)_2$ (1.2 equiv. 2.4 mmol, 490 mg) and $AgNO_3$ (6 mol%, 0.12 mmol, 20 mg). The reaction vessel was degassed and filled with N_2 before dry toluene (30 mL) was added. The mixture was heated to 100 ^{o}C under N_2 and stirred for 24 h with a magnetic stir bar. After cooling to room temperature, the solvent was removed under reduced pressure and the desired phthalimide protected *o*-carboranyl methylamines was isolated by silica gel chromatography using hexanes/EtOAc as the eluent.

Deprotection: The purified phthalimide protected *o*-carboranyl methylamines, iPrOH (10 ml) and H_2O (2 ml) were added to the 100 mL Schlenk bottom. Then $NaBH_4$ (4.0 equiv., 300 mg) was added slowly in portions under N_2 atmosphere. The mixture was stirred at room temperature for 12 h. Upon completion, diluted HCl (1 N) solution was slowly added to quench the unreacted $NaBH_4$. The solvents were removed under *vacuo*. Then, acetic acid (10 mL) and concentrated HCl (35%, 2 mL) were added and the resulting solution was heated for 16 h at 100 ^{o}C. The volatiles were removed under *vacuo*, water (20 mL) was added and the crude product was extracted with Et_2O or THF (10 mL × 3, THF with saturated brine). The combined organic extracts were evaporated under *vacuo*. The hydrochloride salts of the related *o*-carboranyl methylamine (**1d-1e, 1i-1j**) could be purified through chromatography on a silica gel column (200–300 mesh) using hexanes/THF as an eluent.

Basification: The purified hydrochloride salt of the *o*-carboranyl methylamine was added into a 100 mL Schlenk bottom containing CH_2Cl_2/H_2O (20 mL/20 mL). Then, $NaHCO_3$ (9.0 mmol, 750 mg) was added and the mixture was stirred for 12 h at room temperature. Upon completion, the organic phase was collected and the water phase was extracted with CH_2Cl_2 (15 mL × 3). The combined organic extracts were dried with anhydrous Na_2SO_4, filtrated and concentrated under *vacuo* to obtain the *o*-carboranyl methylamine (**1d-1e, 1i-1j**) in 40% to 60% yield.

2.1.4 Compound data for *o*-carboranyl methylamines

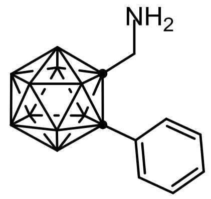

1a: Yield 50%. White solid. ^{1}H NMR (400 MHz, $CDCl_3$): δ 7.63 (d, *J* = 8.0 Hz, 2H), 7.46 (t, *J* = 8.0 Hz, 1H), 7.39 (t, *J* = 8.0 Hz, 2H) (phenyl−**H**), 2.98 (s, 2H) (C**H**$_2$); ^{11}B {^{1}H} NMR (128 MHz, $CDCl_3$): δ −4.6 (2B), −10.6 (2B), −11.3 (2B), −12.2 (2B), −13.4 (2B); ^{13}C NMR (100 MHz, $CDCl_3$): δ 130.9, 130.8, 130.4, 129.1 (phenyl−**C**), 84.8, 83.0 (cage−**C**), 46.2 (**C**H$_2$). IR (neat, cm^{-1}): 2584. HRMS (ESI, positive mode): *m/z* calcd for $C_9B_{10}H_{20}N$ [M+H$^+$]: 250.2590. Found: 250.2586.

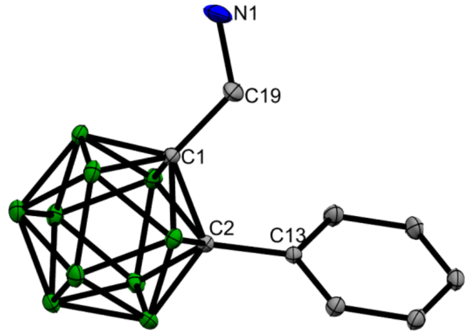

Figure S1. Molecular structure of compound **1a** (ellipsoids at 30% probability and H atoms omitted for clarity)**.** Selected bond distances [Å]: C1–C2 1.710(3, C1–C19 1.539(3), C19–N1 1.403(3).

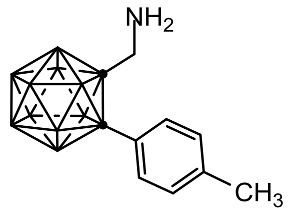

1b: Yield 61%. White solid. ^{1}H NMR (400 MHz, $CDCl_3$): δ 7.49 (d, *J* = 8.0 Hz, 2H), 7.17 (d, *J* = 8.0 Hz, 2H) (phenyl−**H**), 2.98 (s, 2H) (C**H**$_2$), 2.36 (s, 3H) (C**H**$_3$); ^{11}B {^{1}H} NMR (128 MHz, $CDCl_3$): δ −4.6 (2B), −10.6 (2B), −11.4 (2B), −12.2 (2B), −13.4

(2B); ^{13}C NMR (100 MHz, $CDCl_3$): δ 141.2, 130.8, 129.7, 127.6 (phenyl−**C**), 84.8, 83.2 (cage−**C**), 46.2 (**C**H_2), 21.0 (**C**H_3). IR (neat, cm^{-1}): 2581. HRMS (ESI, positive mode): *m/z* calcd for $C_{10}B_{10}NH_{22}$ [M+H$^+$]: 264.2746. Found: 264.2750.

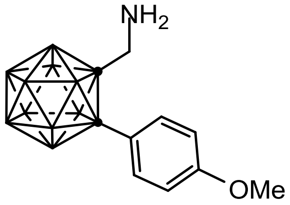

1c: Yield 65%. White solid. ^1H NMR (400 MHz, $CDCl_3$): δ 7.53 (d, *J* = 8.0 Hz, 2H), 6.87 (d, *J* = 8.0 Hz, 2H) (phenyl−**H**), 3.82 (s, 3H) (C**H**$_3$), 2.99 (s, 2H) (C**H**$_2$); ^{11}B {^1H} NMR (128 MHz, $CDCl_3$): δ −4.4 (1B), −5.0 (1B), −10.6 (2B), −11.5 (2B), −12.2 (2B), −13.4 (2B); ^{13}C NMR (100 MHz, $CDCl_3$): δ 161.4, 132.4, 122.5, 114.3 (phenyl−**C**), 85.0, 83.5 (cage−**C**), 55.5 (**C**H_3), 46.1 (**C**H_2). IR (neat, cm^{-1}): 2581. HRMS (ESI, positive mode): *m/z* calcd for $C_{10}B_{10}NOH_{22}$ [M+H$^+$]: 280.2696. Found: 280.2698.

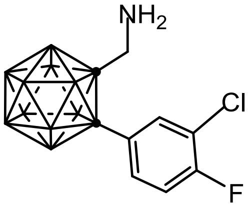

1d: Yield 66%. White solid. ^1H NMR (400 MHz, $CDCl_3$): δ 7.69 (m, 1H), 7.53 (m, 1H), 7.16 (t, *J* = 8.0 Hz, 1H) (phenyl−**H**), 3.01 (s, 2H) (C**H**$_2$); ^{11}B {^1H} NMR (128 MHz, $CDCl_3$): δ −4.3 (2B), −11.1 (3B), −12.1 (2B), −13.4 (3B); ^{13}C NMR (100 MHz, $CDCl_3$): δ 159.6 (d, J_{CF} = 245 Hz), 133.4, 131.0 (d, J_{CF} = 7 Hz), 127.8 (d, J_{CF} = 4 Hz), 122.0 (d, J_{CF} = 18 Hz), 117.1 (d, J_{CF} = 22 Hz) (phenyl−**C**), 84.8, 80.9 (cage−**C**), 46.2 (**C**H_2); ^{19}F NMR (376 MHz, $CDCl_3$): δ −110.9. IR (neat, cm^{-1}): 2581. HRMS (ESI, positive mode): *m/z* calcd for $C_9B_{10}NFClH_{18}$ [M+H$^+$]: 302.2124. Found: 302.2112.

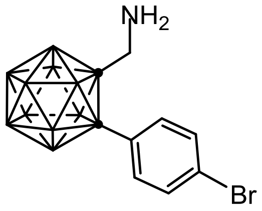

1e: Yield 62%. White solid. ^{1}H NMR (400 MHz, $CDCl_3$): δ 7.48-7.54 (m, 4H) (phenyl−**H**), 2.99 (s, 2H) (C**H**$_2$); ^{11}B {^{1}H} NMR (128 MHz, $CDCl_3$): δ −4.4 (2B), −11.3 (3B), −12.3 (2B), −13.6 (3B); ^{13}C NMR (100 MHz, $CDCl_3$): δ 132.3, 130.8, 129.6, 125.8 (phenyl−**C**), 84.7, 82.0 (cage−**C**), 46.2 (**C**H$_2$), 21.0 (**C**H$_3$). IR (neat, cm^{-1}): 2571. HRMS (ESI, positive mode): *m/z* calcd for $C_9B_{10}NBrH_{19}$ [M+H$^+$]: 329.1699. Found: 329.1682.

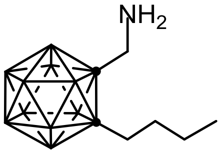

1g: Yield 54%. Colorless oil. ^{1}H NMR (400 MHz, $CDCl_3$): δ 3.35 (s, 2H) (C**H**$_2$), 2.14 (t, J = 8.0 Hz, 2H), 1.50 (m, 2H), 1.32 (m, 2H), 0.91 (t, J = 8.0 Hz, 3H) (n-butyl); ^{11}B {^{1}H} NMR (128 MHz, $CDCl_3$): δ −5.9 (2B), −12.1 (4B), −12.8 (4B); ^{13}C NMR (100 MHz, $CDCl_3$): δ 80.9, 78.5 (cage−**C**), 45.2 (**C**H$_2$), 33.8, 30.8, 21.4, 12.6 (n-butyl). IR (neat, cm^{-1}): 2575. HRMS (ESI, positive mode): *m/z* calcd for $C_7B_{10}NH_{24}$ [M+H$^+$]: 230.2917. Found: 230.2906.

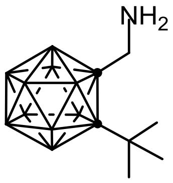

1h: Yield 62%. Colorless oil. ^{1}H NMR (400 MHz, $CDCl_3$): δ 3.51 (s, 2H) (C**H**$_2$), 1.33 (s, 9H) (CH_3); ^{11}B {^{1}H} NMR (128 MHz, $CDCl_3$): δ −5.0 (2B), −11.6 (5B), −13.0 (3B); ^{13}C NMR (100 MHz, $CDCl_3$): δ 91.8, 85.4 (cage−**C**), 47.3 (**C**H$_2$), 38.8, 33.6, (t-butyl). IR (neat, cm^{-1}): 2575. HRMS (ESI, positive mode): *m/z* calcd for $C_7B_{10}NH_{24}$ [M+H$^+$]: 230.2917. Found: 230.2908.

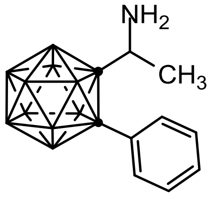

1i: Yield 52%. White solid. ^{1}H NMR (400 MHz, CDCl$_3$): δ 7.63 (d, *J* = 8.0 Hz, 2H), 7.46 (t, *J* = 8.0 Hz, 1H), 7.39 (t, *J* = 8.0 Hz, 2H) (phenyl−**H**), 2.80 (q, *J* = 6.5 Hz, 1H) (C**H**), 1.22 (d, *J* = 6.5 Hz, 3H) (C**H**$_3$); ^{11}B {^{1}H} NMR (128 MHz, CDCl$_3$): δ −3.8 (1B), −5.0 (1B), −10.2 (1B), −11.0 (3B), −12.1 (1B), −13.5 (3B); ^{13}C NMR (100 MHz, CDCl$_3$): δ 131.0, 130.8, 130.6, 129.1 (phenyl−**C**), 90.0, 84.7 (cage−**C**), 49.1 (**C**H), 24.2 (**C**H$_3$). IR (neat, cm^{-1}): 2583. HRMS (ESI, positive mode): *m/z* calcd for $C_{10}B_{10}NH_{22}$ [M+H$^+$]: 264.2746. Found: 264.2751.

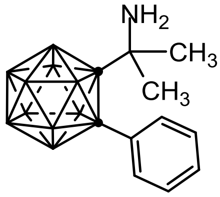

1j: Yield 41%. White solid. ^{1}H NMR (400 MHz, CDCl$_3$): δ 7.70 (m, 2H), 7.43 (m, 1H), 7.33 (m, 2H) (phenyl−**H**), 1.19 (s, 6H) (C**H**$_3$); ^{11}B {^{1}H} NMR (128 MHz, CDCl$_3$): δ −4.0 (2B), −9.4 (2B), −12.0 (3B), −13.3 (3B); ^{13}C NMR (100 MHz, CDCl$_3$): δ 132.6, 131.2, 130.6, 128.9 (phenyl−**C**), 95.4, 83.7 (cage−**C**), 56.5 (**C**Me$_2$), 33.0 (**C**H$_3$). IR (neat, cm^{-1}): 2577. HRMS (ESI, positive mode): *m/z* calcd for $C_{11}B_{10}NH_{24}$ [M+H$^+$]: 278.2914. Found: 278.2907.

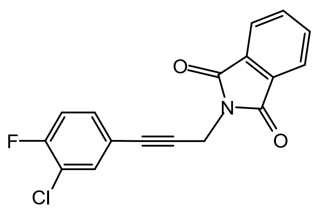

According to the literature report,[S6] this compound was synthesized via sonogashira coupling with aryl iodides.

Yield 90%. Light yellow solid. ^{1}H NMR (400 MHz, CDCl$_3$): δ 7.90 (m, 2H), 7.75 (m, 2H), 7.47 (m, 1H), 7.29 (m, 1H), 7.04 (t, *J* = 8.0 Hz, 1H) (phenyl−**H**), 4.66 (s, 2H)

(C**H**$_2$); ^{13}C NMR (100 MHz, CDCl$_3$): δ 167.0 (**C**=O), 158.2 (d, J_{CF} = 251 Hz), 134.2, 134.1, 132.0, 131.9 (d, J_{CF} = 7 Hz), 123.6, 121.0 (d, J_{CF} = 19 Hz), 119.5 (d, J_{CF} = 4 Hz), 116.6 (d, J_{CF} = 22 Hz), 83.5, 80.8, 27.7; ^{19}F NMR (376 MHz, CDCl$_3$): δ −113.2. IR (neat, cm^{-1}): 2583, 2266, 1710. HRMS (ESI, positive mode): *m/z* calcd for $C_{17}NO_2FClH_{10}$ [M+H$^+$]: 314.0388. Found: 314.0376.

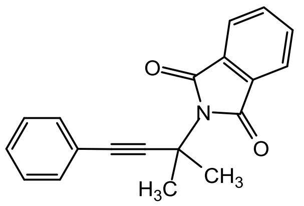

According to the literature report,[S6] this compound was synthesized from the propargyl alcohol precursors.

Yield 42%. Light yellow oil. ^{1}H NMR (400 MHz, CDCl$_3$): δ 7.82 (m, 2H), 7.70 (m, 2H), 7.45 (m, 2H), 7.28 (m, 2H), (phenyl−**H**), 2.06 (s, 6H) (C**H**$_3$); ^{13}C NMR (100 MHz, CDCl$_3$): δ 168.0 (**C**=O), 133.9, 132.0, 131.8, 128.1, 122.9, 91.3, 82.9, 52.8, 29.4. IR (neat, cm^{-1}): 3059, 2989, 2579, 1720. HRMS (ESI, positive mode): *m/z* calcd for $C_{19}NO_2H_{16}$ [M+H$^+$]: 290.1181. Found: 290.1173.

2.2 Organometallic investigations

2.2.1 Coordination between 1a and PdX$_2$ (X = OAc and Cl).

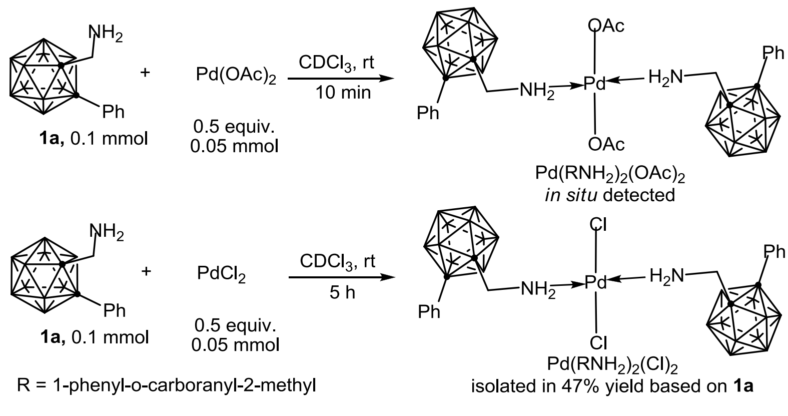

Scheme S4. Coordination between *o*-carboranyl methylamine **1a** and PdX$_2$ (X = OAc, Cl) in CDCl$_3$ at room temperature.

^{1}H NMR and HRMS detection on the formation of ($Pd(RNH_2)_2(OAc)_2$).

o-Carboranyl methylamine **1a** (0.1 mmol, 25 mg) and $Pd(OAc)_2$ (0.05 mmol, 11 mg) was added into a NMR tube, followed by the addition of $CDCl_3$ (0.6 mL) as a solvent. The mixture was shaken and allowed for ^{1}H NMR test in 10 min. For comparison, the ^{1}H NMR of **1a** and $Pd(OAc)_2$ in $CDCl_3$ have also been tested (Figure S2). Analysis: As is known,[S10] $Pd(OAc)_2$ exists as the $Pd_3(OAc)_6$ trimer in $CHCl_3$ (Figure S2a). As shown in the ^{1}H NMR spectrum (Figure S2c), after combination of **1a** with $Pd(OAc)_2$ in $CDCl_3$, the trimer are depolymerized along with the consumption of **1a**, a major product could be observed as shown in Scheme 4. The integration (2 : 2 : 3) between amino (NH_2, $\delta = 4.45$), aminomethyl (CH_2, $\delta = 2.91$) and acetate (CH_3, $\delta = 1.58$) indicated that a bis(amine) Pd(II) complex ($Pd(RNH_2)_2(OAc)_2$) formed. In addition, ESI-HRMS analysis of the reaction mixture also supported the generation of $Pd(RNH_2)_2(OAc)_2$ species (Figure S3). These findings are consistant with the literature report for the coordination chemistry between amine and $Pd(OAc)_2$.[S11]

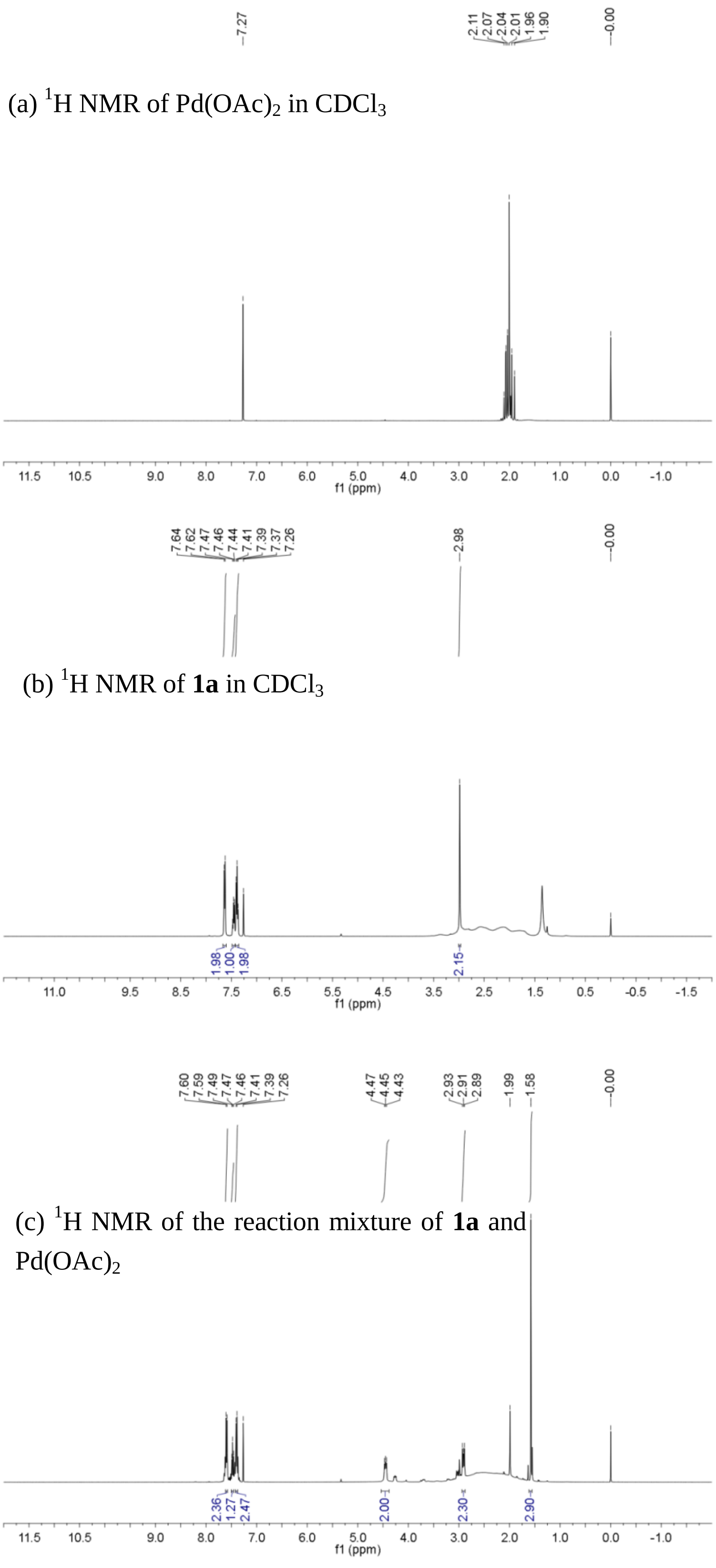

(a) ^{1}H NMR of $Pd(OAc)_2$ in $CDCl_3$

(b) ^{1}H NMR of **1a** in $CDCl_3$

(c) ^{1}H NMR of the reaction mixture of **1a** and $Pd(OAc)_2$

Figure S2. ^{1}H NMR spectrums of illustrating the coordination between *o*-carboranyl methylamine **1a** and $Pd(OAc)_2$ to for bis(amine) Pd(II) complex.

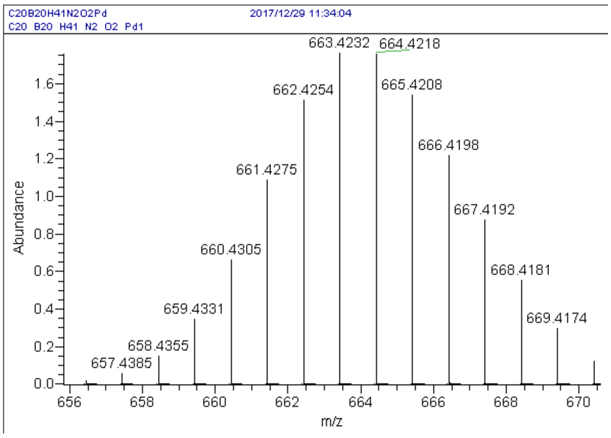

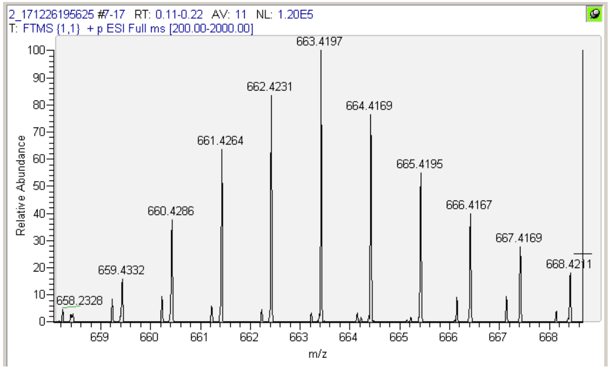

Figure S3. ESI-HRMS of $Pd(RNH_2)_2(OAc)_2$. HRMS (ESI, positive mode): *m/z* calcd for $C_{20}B_{20}H_{41}N_2O_2Pd$ $[M{-}OAc^{-}]^{+}$: 663.4232 (up). Found: 663.4197 (down).

Synthesis and characterization of ($Pd(RNH_2)_2(Cl)_2$).

o-Carboranyl methylamine **1a** (0.1 mmol, 25 mg) and $PdCl_2$ (0.05 mmol, 9 mg) was added into a reaction tube (10 mL) with a magnetic stir bar, followed by the addition of $CDCl_3$ (1.0 mL) as a solvent. The mixture was stirred for 5h at 25 ^{o}C. Upon completion, the reaction mixture was concentrated in *vacuo* and purified by silica gel chromatography using hexanes/CH_2Cl_2 (v/v = 1/2) as the eluent to afford $Pd(RNH_2)_2(Cl)_2$ as a light yellow solid in nearly quantitative conversion. (32 mg, yield: 47% based on **1a**)

$Pd(RNH_2)_2(Cl)_2$: Yield 47%. Light yellow solid. ^{1}H NMR (400 MHz, $CDCl_3$): δ 7.52-7.57 (m, 3H), 7.41-7.45 (m, 2H), 3.03-3.07 (m, 2H), 2.83-2.87 (m, 2H); ^{11}B {^{1}H} NMR (128 MHz, $CDCl_3$): δ −3.1 (2B), −4.6 (4B), −11.1 (8B), −12.4 (2B), −13.6 (4B); ^{13}C NMR (100 MHz, $CDCl_3$): δ 131.6, 131.1, 129.6, 83.7, 78.7, 47.2. IR (neat, cm^{-1}):

2583. HRMS (ESI, positive mode): *m/z* calcd for $C_{18}B_{20}H_{38}N_2PdCl$ $[M-Cl^-]^+$: 640.3718. Found: 640.3732.

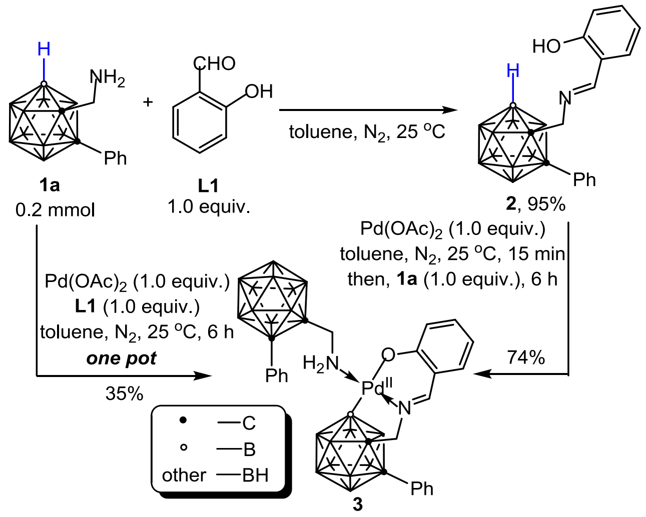

Scheme S5. Stoichiometric reactions toward Pd-mediated B−H activation of *o*-carboranyl methylamine **1a**.

2.2.2 Synthesis of 2.

A reaction tube (10 mL) with a magnetic stir bar was charged with **1a** (0.2 mmol, 50 mg), salicylaldehyde (**L1**, 1.0 equiv. 25 mg) and toluene (2 mL). The reaction tube was sealed under N_2 atmosphere and allowed to stir at 25 °C for 3 h. Upon completion, the reaction mixture was concentrated in *vacuo* and purified by silica gel chromatography using hexanes/CH_2Cl_2 (v/v = 2/1) as the eluent to afford **2**. (67 mg, yield: 95% based on **1a**)

2.2.3 Synthesis of 3.

Method 1: A reaction tube (10 mL) with a magnetic stir bar was charged with **1a** (0.2 mmol, 50 mg), salicylaldehyde (**L1**, 1.0 equiv. 25 mg), $Pd(OAc)_2$ (1.0 equiv., 0.2 mmol, 46 mg) and toluene (2 mL). The reaction tube was sealed under N_2 atmosphere and allowed to stir at 25 °C for 6 h. Upon completion, the reaction mixture was

concentrated in *vacuo* and purified by silica gel chromatography using hexanes/CH_2Cl_2 (v/v = 2/1 to 1/2 to CH_2Cl_2) as the eluent to afford **3** as a light yellow solid. (49 mg, yield: 35% based on **1a** (0.2 mmol)).

Method 2: A reaction tube (10 mL) with a magnetic stir bar was charged with **2** (0.1 mmol, 35 mg), $Pd(OAc)_2$ (1.0 equiv., 0.1 mmol, 23 mg) and toluene (1 mL). The reaction tube was degassed and filled with N_2 and allowed to stir at 25 ^oC for 15 min to form a yellow solution. Then another portion of **1a** (1.0 equiv. 0.1 mmol, 25 mg) was added and the mixture was stirred at 25 ^oC for 6 h. Upon completion, the reaction mixture was concentrated in *vacuo* and purified by silica gel chromatography using hexanes/CH_2Cl_2 (v/v = 2/1 to 1/2 to CH_2Cl_2) as the eluent to afford **3** as a light yellow solid. (53 mg, yield: 75% based on **2**)

Note: Single crystal of **3** suitable for X-ray analysis was obtained by diffusion of hexane into a CH_2Cl_2 solution of **3** at 0 ^oC.

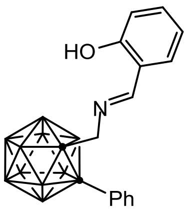

2: Yield 95%. Light yellow solid. ^1H NMR (400 MHz, $CDCl_3$): δ 12.4 (s, 1H) (O**H**), 7.75 (s, 1H), 7.67 (d, *J* = 8.0 Hz, 2H), 7.47 (t, *J* = 8.0 Hz, 1H), 7.39 (d, *J* = 8.0 Hz, 2H), 7.35 (m, 1H), 7.09 (m, 1H), 6.98 (d, *J* = 8.0 Hz, 1H), 6.88 (m, 1H) (phenyl−**H** and **H**C=N), 3.87 (s, 2H) (C**H**$_2$); ^{11}B {^1H} NMR (128 MHz, $CDCl_3$): δ −4.3 (3B), −11.5 (5B), −13.2 (2B); ^{13}C NMR (100 MHz, $CDCl_3$): δ 167.5, 160.9, 133.4, 132.0, 130.9, 130.2, 129.0, 119.0, 117.9, 117.2 (phenyl−**C** and H**C**=N), 82.6, 79.5 (cage−**C**), 61.5 (**C**H$_2$). IR (neat, cm^{-1}): 2927, 2573. HRMS (ESI, positive mode): *m/z* calcd for $C_{16}B_{10}NOH_{24}$ [M+H$^+$]: 354.2852. Found: 354.2856.

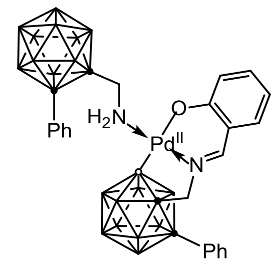

3: Yield 35% based on **1a**. Light yellow solid. ^{1}H NMR (400 MHz, CD_2Cl_2): δ 7.66 (d, *J* = 8.0 Hz, 2H), 7.62 (d, *J* = 8.0 Hz, 2H), 7.56 (s, 1H), 7.51 (t, *J* = 8.0 Hz, 1H), 7.36-7.46 (m, 5H), 7.24 (t, *J* = 8.0 Hz, 1H), 6.95 (d, *J* = 8.0 Hz, 1H), 6.57 (d, *J* = 8.0 Hz, 1H), 6.40 (t, *J* = 8.0 Hz, 1H) (phenyl−**H** and **H**C=N), 4.04 (d, *J* = 15 Hz, 1H), 3.53 (d, *J* = 15 Hz, 1H), 3.42 (d, *J* = 6 Hz, 1H), 3.40 (d, *J* = 6 Hz, 1H) (C**H**$_2$); $^{11}B\{^{1}H\}$ NMR (128 MHz, CD_2Cl_2): δ −3.0 (2B), −4.2 (2B), −5.2 (2B), −10.4 (8B), −11.7 (2B), −12.6 (4B); ^{13}C NMR (100 MHz, CD_2Cl_2): δ 164.8, 135.7, 135.0, 131.3, 131.1, 131.0, 130.6, 130.4, 129.5, 129.4, 129.1, 129.0, 122.9, 119.2, 113.5 (phenyl−**C** and H**C**=N), 84.0, 82.6, 81.1 (cage−**C**), 66.8, 47.5 (**C**H$_2$). IR (neat, cm^{-1}): 2958, 2581. HRMS (ESI, positive mode): *m/z* calcd for $C_{25}B_{20}N_2OPdH_{41}$ [M+H$^+$]: 707.4255. Found: 707.4250.

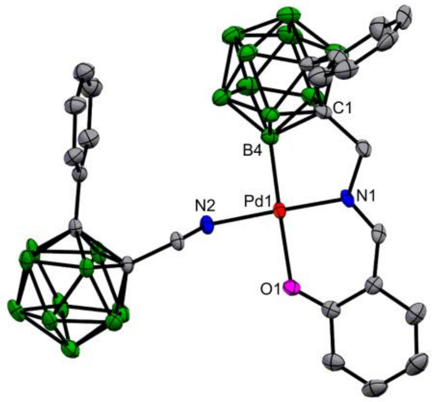

Figure S4. Molecular structure of compound **3** (ellipsoids at 20% probability and H atoms omitted for clarity). Selected bond distances [Å] and angles [°]: N1–Pd1 2.021(10), O1–Pd1 2.108(4), B4–Pd1 2.009(14), N2–Pd1 2.080(5), B4–Pd1–O1 178.4(4), N1–Pd1–N2 173.1(3).

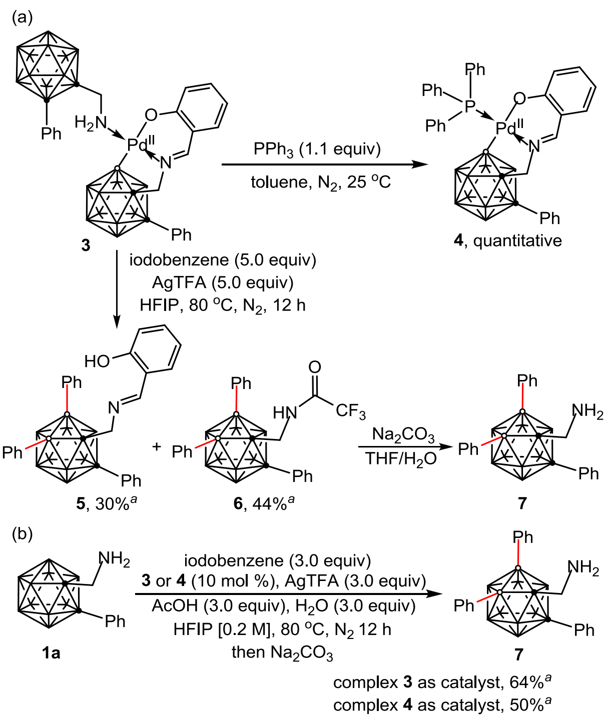

Scheme S6. Ligand exchange, stoichiometric diarylation and catalytic performance of 5,6-fused bicylic palladacycle **3**.

2.2.4 Synthesis of 4 (ligand exchange).

A reaction tube (10 mL) with a magnetic stir bar was charged with **3** (0.1 mmol, 71 mg) and toluene (1 mL). The reaction tube was degassed and filled with N_2 before PPh_3 (1.1 equiv., 0.11 mmol, 29 mg) was added into the mixture. The reaction was stirred at 25 °C for 5 min. Then , the reaction mixture was concentrated in *vacuo* and purified by silica gel chromatography using CH_2Cl_2 as the eluent to afford **4** as a light yellow solid. (72 mg, yield: quantitative based **1a**)

Note: complex **4** exhibits low solubility in conventional organic solvent such as CH_2Cl_2, $CHCl_3$, THF, DMSO, toluene etc. at room temperature. NMR was conducted after heating the d_8-toluene solution of **4** using a heat gun. Single crystal of **4** suitable for X-ray analysis was obtained by slow evaporation of a diluted CH_2Cl_2 solution of **4** at room temperature.

2.2.5 Synthesis of 5 and 6 (subsequent arylation)

A reaction tube (10 mL) with a magnetic stir bar was charged with **3** (0.1 mmol, 71 mg), iodobenzene (5.0 equiv. 0.5 mmol, 100 mg), AgTFA (5.0 equiv. 0.5 mmol, 110 mg) and HFIP (1 mL). The reaction tube was sealed under N_2 atmosphere and allowed to stir at ambient temperature for 30 minutes, then heated to 80 ^oC for 12 hours. Upon completion, the reaction mixture was cooled to room temperature and diluted with 5 mL of CH_2Cl_2. The mixture was filtered through a silica gel plug (3 cm) and concentrated in *vacuo*. The crude reaction mixture was purified on preparative TLC using hexanes/CH_2Cl_2 (v/v = 2/1 to 1/1) as the eluent to afford the desired product **5** (30 mg, yield: 30% based on *o*-carboranyl methylamine unit in **3** (0.2 mmol)) and **6** (43 mg, yield: 44% based on *o*-carboranyl methylamine unit in **3** (0.2 mmol)).
Note: Single crystal of **6** suitable for X-ray analysis was obtained by slow diffusion of hexane into a CH_2Cl_2 solution of **6** at room temperature under N_2 atmosphere.

2.2.6 Deprotection of 6

Trifluoroacetamide **6** (0.1 mmol, 50 mg) was dissolved in THF (2 mL) in a reaction tube (10 mL) with a magnetic stir bar. Na_2CO_3 (0.3 mmol, 34 mg) and H_2O (2.0 mL) was added into the reaction tube. The mixture was stirred vigorously at 25 ^oC for 4 h. Upon completion (TLC monitoring), the reaction mixture was diluted with 10 mL of CH_2Cl_2, 10 mL of H_2O and was extracted with CH_2Cl_2 (10 mL × 3). The combined organic extracts were dried with anhydrous Na_2SO_4, filtrated and concentrated under *vacuo* to obtain the deprotected *o*-carboranyl methylamine (**7**) in 90% yield based on **7**.

2.2.7 Catalytic performance of 3 and 4

A reaction tube (10 mL) with a magnetic stir bar was charged with **1a** (0.1 mmol, 25 mg), iodobenzene (3.0 equiv., 0.3 mmol, 60 mg), complex **3** or **4** (10 mol%, 0.01

mmol), AgTFA (3.0 equiv. 0.3 mmol, 66 mg), AcOH (3.0 equiv., 0.3 mmol, 18 mg), H_2O (3.0 equiv., 0.3 mmol, 5 mg) and HFIP (0.5 mL). The reaction tube was sealed under N_2 atmosphere and allowed to stir at ambient temperature for 30 minutes, then heated to 80 ^{o}C for 12 hours. Upon completion, the reaction mixture was cooled to room temperature and diluted with 2 mL of THF and 2 mL of H_2O. Na_2CO_3 (0.6 mmol, 66 mg) was added into the mixture and stirred for 3 h at room temperature. The mixture was filtered through a silica gel plug and concentrated under *vacuo* to remove the organic solvent. The residue was extracted with CH_2Cl_2 (10 mL × 3) from water. The combined organic extracts were concentrated under *vacuo*. The crude reaction mixture was purified on preparative TLC using hexanes/CH_2Cl_2 (v/v = 1/2) as the eluent to afford the desired product **7**.

Isolated yield: 31 mg using **3** as the catalyst, yield: 64% based on *o*-carboranyl methylamine unit (0.12 mmol) in **1a** (0.1 mmol) and **3** (0.02 mmol). 22 mg using **4** as the catalyst, yield: 50% based on *o*-carboranyl methylamine unit (0.11 mmol) in **1a** (0.1 mmol) and **3** (0.01 mmol).

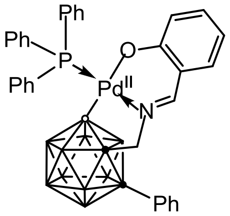

4: Yield 99% based on **3**. Light yellow solid. ^{1}H NMR (400 MHz, d_8-toluene): δ 7.80-7.85 (m, 5H), 7.43 (d, *J* = 8.0 Hz, 2H), 6.92-7.13 (overlapped with toluene), 6.68 (m, 1H), 6.57 (d, *J* = 8.0 Hz, 1H), 6.36 (m, 1H) (phenyl−**H** and **H**C=N), 3.55 (d, *J* = 15 Hz, 1H), 3.21 (d, *J* = 15 Hz, 1H) (C**H**$_2$); ^{11}B {^{1}H} NMR (128 MHz, d_8-toluene): δ 1.0 (2B), 0.1 (2B), −5.2 (4B), −7.9 (2B); ^{13}C NMR (100 MHz, d_8-toluene): δ 174.7, 169.5, 140.5, 140.4, 139.7, 137.2, 136.6, 135.8, 135.2, 135.1, 132.8, 128.9, 123.9, 117.6 (phenyl−**C** and H**C**=N), 88.3, 87.3 (cage−**C**), 70.8 (**C**H$_2$). ^{31}P NMR (162 MHz, $CDCl_3$): δ 39.9 (**P**Ph$_3$). IR (neat, cm^{-1}): 2923, 2849, 2585. HRMS (ESI, positive mode): *m/z* calcd for $C_{34}B_{10}NOPdPH_{37}$ [M+H^{+}]: 720.2645. Found: 720.2652.

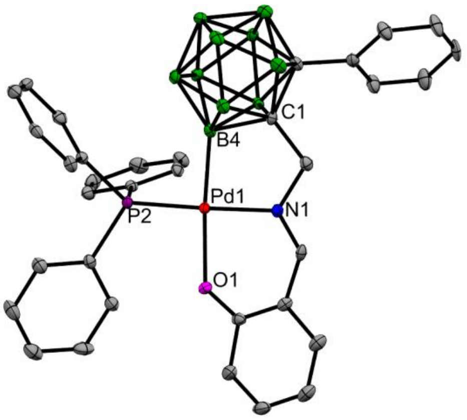

Figure S5. Molecular structure of compound **4** (ellipsoids at 30% probability and H atoms omitted for clarity)**.** Selected bond distances [Å] and angles [^{o}]: N1–Pd1 2.064(3), O1–Pd1 2.096(3), B4–Pd1 2.049(5), P2–Pd1 2.2560(11), B4–Pd1–O1 175.06(15), N1–Pd1–P2 176.05(10).

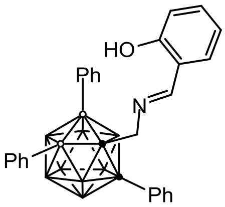

5: Yield 30%. Light yellow solid. ^{1}H NMR (400 MHz, $CDCl_3$): δ 11.39 (s, 1H) (O**H**), 7.72 (d, *J* = 8.0 Hz, 2H), 7.49 (d, *J* = 8.0 Hz, 4H), 7.31 (t, *J* = 8.0 Hz, 4H), 7.24 (m, 6H), 6.80-6.83 (m, 2H), 6.72 (t, *J* = 8.0 Hz, 1H), 6.59 (m, 1H) (phenyl−**H**), 4.25 (s, 2H) (C**H**$_2$); ^{11}B {^{1}H} NMR (128 MHz, $CDCl_3$): δ −2.9 (3B), −3.9 (1B), −11.0 (3B), −12.8 (3B); ^{13}C NMR (100 MHz, $CDCl_3$): δ 167.8, 160.4, 134.6, 133.0, 131.9, 130.3, 128.9, 128.6, 127.8, 118.6, 117.7, 117.0 (phenyl−**C** and H**C**=N), 85.4, 81.5 (cage−**C**), 59.8 (**C**H$_2$). IR (neat, cm^{-1}): 3059, 2581. HRMS (ESI, positive mode): *m/z* calcd for $C_{28}B_{10}NOH_{32}$ [M+H^{+}]: 506.3501. Found: 506.3491.

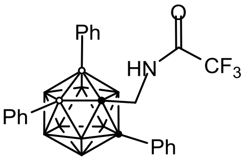

6: Yield 44%. White solid. ^{1}H NMR (400 MHz, $CDCl_3$): δ 7.65 (d, *J* = 8.0 Hz, 2H), 7.51 (t, *J* = 8.0 Hz, 1H), 7.48 (m, 4H), 7.43 (m, d, *J* = 8.0 Hz, 2H), 7.37 (m, 2H), 7.30 (t, *J* = 8.0 Hz, 4H) (phenyl−**H**), 5.54 (br m, 1H) (N**H**), 4.08 (d, *J* = 6.0 Hz, 2H) (C**H**$_2$);

$^{11}B\{^{1}H\}$ NMR (128 MHz, $CDCl_3$): δ −3.0 (4B), −10.2 (2B), −12.8 (4B); ^{13}C NMR (100 MHz, $CDCl_3$): δ 155.3 (q, J_{C-F} = 37 Hz) (**C**=O), 134.6, 131.6, 131.0, 129.5, 129.3 (d, J_{C-F} = 4 Hz), 128.4 (phenyl−**C**), 114.9 (q, J_{C-F} = 286 Hz) (**C**F_3), 84.9, 78.1 (cage−**C**), 38.6 (**C**H_2); ^{19}F NMR (376 MHz, $CDCl_3$): δ −76.8. IR (neat, cm^{-1}): 2575, 1743. HRMS (ESI, negative mode): *m/z* calcd for $C_{23}B_{10}NOF_3H_{25}$ $[M-H]^-$: 496.2883. Found: 496.2890.

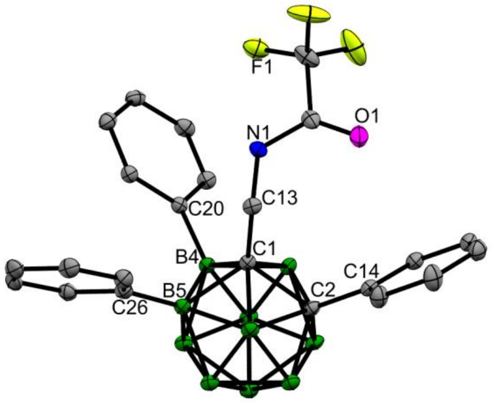

Figure S6. Molecular structure of compound **6** (ellipsoids at 30% probability and H atoms omitted for clarity)**.** Selected bond distances [Å]: C1–C2 1.7462(17), B4–C20 1.5826(19), B5–C26 1.5755(19), C1–C13 1.5288(18), C13–N1 1.4498(17).

2.3 Condition screening

2.3.1 Solvent screening

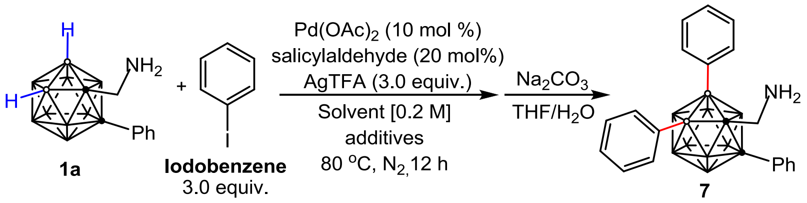

Table S1. Optimization of Pd(II)-catalyzed B–H diarylation of *o*-carboranyl methylamine (**1a**).[a]

entry	solvent [0.2 M]	additives	yield of **7**[b]
1	DCE[c]	/	trace
2	Toluene	/	n.d.
3	AcOH	/	32%
4	CH_3CN		n.d.
5	HFIP[d]	/	45%
6	HFIP	H_2O (3.0 equiv.)	58%
7	HFIP	AcOH (3.0 equiv.)<br>H_2O (3.0 equiv.)	70%
9[e]	HFIP	AcOH (3.0 equiv.)<br>H_2O (3.0 equiv.)	n.r.
8[f]	HFIP	AcOH (3.0 equiv.)<br>H_2O (3.0 equiv.)	42%

[a] Conditions: 0.1 mmol of **1a**, 3.0 equiv. of iodobenzene (**2a**), 10 mol% of $Pd(OAc)_2$, 20 mol% of salicylaldehyde, 3.0 equiv. of AgTFA, 0.5 mL of solvent, 80 ^oC, sealed under N_2 atmosphere, 12 h. [b] Isolated yield after workup. [c] DCE: 1,2-dichloroethane. [d] HFIP: hexafluoroisopropanol. [e] No salicylaldehyde as transient direct group. [f] Sealed under air atmosphere. n.d. = desired product not detected. n.r. = no reaction.

Procedure: A reaction tube (10 mL) with a magnetic stir bar was charged with **1a** (0.1 mmol, 25 mg), iodobenzene (3.0 equiv. 0.3 mmol), $Pd(OAc)_2$ (10 mol %, 0.01 mmol, 2.3 mg), AgTFA (3.0 equiv. 0.3 mmol, 66 mg), salicylaldehyde (20 mol%, 0.02 mmol, 2.4 mg) and solvent (0.5 mL). The reaction tube was sealed under N_2 atmosphere and allowed to stir at ambient temperature for 30 minutes, then heated to 80 ^oC for 12 hours. Upon completion, the reaction mixture was cooled to room temperature and diluted with 2 mL of THF and 2 mL of water. Na_2CO_3 (0.6 mmol, 66 mg) was added into the mixture and stirred for 3 h at room temperature. The mixture was filtered through a silica gel plug and concentrated under *vacuo* to remove the organic solvent.

The residue was extracted with CH_2Cl_2 (10 mL × 3) from water. The combined organic extracts were concentrated under *vacuo*. The crude reaction mixture was purified on preparative TLC using hexanes/CH_2Cl_2 (v/v = 1/2) as the eluent to obtain **7**.

2.3.2 Ligand screening

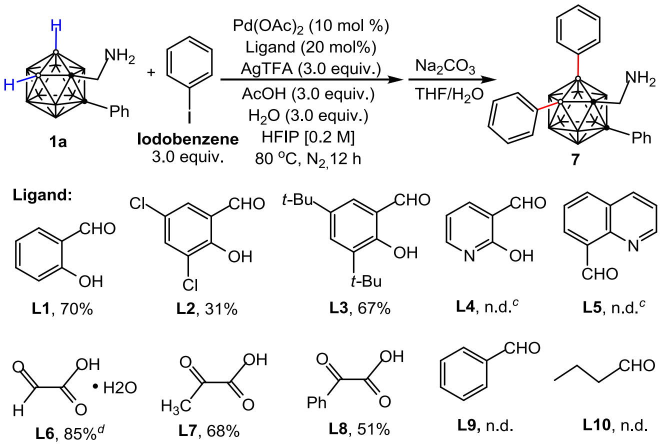

[a]Conditions: **1a** (0.1 mmol), iodobenzene (0.3 mmol), $Pd(OAc)_2$ (10 mol%), Ligand (20 mol%), AgTFA (0.3 mmol), AcOH (0.3 mmol), H_2O (0.3 mmol), HFIP (0.5 mL), 80 ^{o}C, N_2 atmosphere, 12 h. [b]Isolated yield. [c]**1a** was decomposed. [d]optimal condition. n.d.: desired product not detected.

Procedure: A reaction tube (10 mL) with a magnetic stir bar was charged with **1a** (0.1 mmol, 25 mg), iodobenzene (3.0 equiv. 0.3 mmol), $Pd(OAc)_2$ (10 mol %, 0.01 mmol, 2.3 mg), AgTFA (3.0 equiv. 0.3 mmol, 66 mg), ligand (20 mol%, 0.02 mmol), AcOH (3.0 equiv., 0.3 mmol, 18 mg), H_2O (3.0 equiv., 0.3 mmol, 5.4 mg) and HFIP (0.5 mL). The reaction tube was sealed under N_2 atmosphere and allowed to stir at ambient temperature for 30 minutes, then heated to 80 ^{o}C for 12 hours. Upon completion, the reaction mixture was cooled to room temperature and diluted with 2 mL of THF and 2 mL of water. Na_2CO_3 (0.6 mmol, 66 mg) was added into the mixture and stirred for 3 h at room temperature. The mixture was filtered through a silica gel plug and concentrated under *vacuo* to remove the organic solvent. The residue was extracted

with CH_2Cl_2 (10 mL × 3) from water. The combined organic extracts were concentrated under *vacuo*. The crude reaction mixture was purified on preparative TLC using hexanes/CH_2Cl_2 (v/v = 1/2) as the eluent to obtain **7**.

2.4 General procedure for Pd-catalyzed B(4,5)-diarylation of *o*-carboranyl methylamines with a catalytic transient directing group

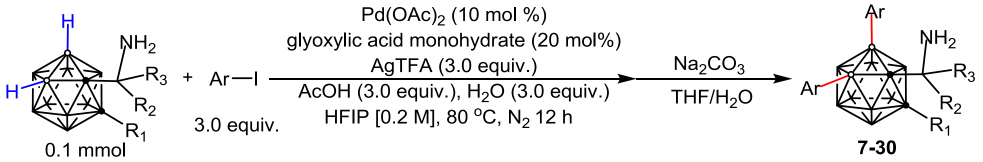

A reaction tube (10 mL) with a magnetic stir bar was charged with *o*-carboranyl methylamine substrate (0.1 mmol), aryl iodide (3.0 equiv. 0.3 mmol), $Pd(OAc)_2$ (10 mol %, 0.01 mmol, 2.3 mg), AgTFA (3.0 equiv. 0.3 mmol, 66 mg), glyoxylic acid monohydrate (20 mol%, 0.02 mmol, 1.8 mg), AcOH (3.0 equiv., 0.3 mmol, 18 mg), H_2O (3.0 equiv., 0.3 mmol, 5.4 mg) and HFIP (0.5 mL). The reaction tube was sealed under N_2 atmosphere and allowed to stir at ambient temperature for 30 minutes, then heated to 80 ^oC for 12 hours. Upon completion, the reaction mixture was cooled to room temperature and diluted with 2 mL of THF and 2 mL of water. Na_2CO_3 (0.6 mmol, 66 mg) was added into the mixture and stirred for 3 h at room temperature. The mixture was filtered through a silica gel plug and concentrated under *vacuo* to remove the organic solvent. The residue was extracted with CH_2Cl_2 (10 mL × 3) from water. The combined organic extracts were concentrated under *vacuo*. The crude reaction mixture was purified on preparative TLC or flash column chromatography using hexanes/CH_2Cl_2 as the eluent.

Compound data:

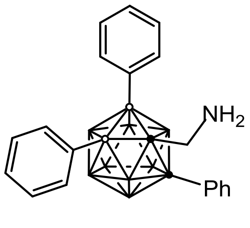

7: Yield 85%. White solid. ^{1}H NMR (400 MHz, $CDCl_3$): δ 7.76 (d, *J* = 8.0 Hz, 2H), 7.48 (t, *J* = 8.0 Hz, 1H), 7.43 (m, 5H), 7.31 (m, 2H), 7.24 (m, 5H) (phenyl−**H**), 3.44 (s, 2H) (C**H**$_2$); ^{11}B {^{1}H} NMR (128 MHz, $CDCl_3$): δ −3.0 (3B), −4.4 (1B), −11.4 (2B), −12.9 (4B); ^{13}C NMR (100 MHz, $CDCl_3$): δ 134.5, 130.9, 130.7, 130.6, 129.1, 128.5, 127.8 (phenyl−**C**), 83.4, 82.1 (cage−**C**), 43.6 (**C**H$_2$). IR (neat, cm^{-1}): 2577. HRMS (ESI, positive mode): *m/z* calcd for $C_{21}B_{10}NH_{28}$ [M+H$^+$]: 402.3239. Found: 402.3221.

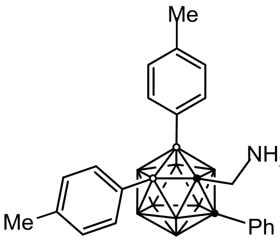

8: Yield 87%. White solid. ^{1}H NMR (400 MHz, $CDCl_3$): δ 7.76 (d, *J* = 8.0 Hz, 2H), 7.48 (t, *J* = 8.0 Hz, 1H), 7.41 (m, 2H), 7.33 (d, *J* = 8.0 Hz, 4H), 7.07 (d, *J* = 8.0 Hz, 4H) (phenyl−**H**), 3.41 (s, 2H) (C**H**$_2$), 2.32 (s, 3H) (C**H**$_3$); ^{11}B {^{1}H} NMR (128 MHz, $CDCl_3$): δ −3.0 (3B), −4.6 (1B), −11.2 (2B), −13.2 (4B); ^{13}C NMR (100 MHz, $CDCl_3$): δ 138.3, 134.5, 130.9, 130.8, 130.7, 129.1, 128.7 (phenyl−**C**), 83.4, 81.9 (cage−**C**), 43.7 (**C**H$_2$), 21.2 (**C**H$_3$). IR (neat, cm^{-1}): 2575. HRMS (ESI, positive mode): *m/z* calcd for $C_{23}B_{10}NH_{32}$ [M+H$^+$]: 430.3530. Found: 430.3529.

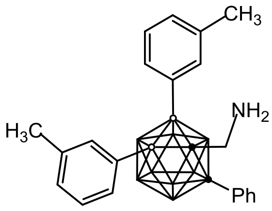

9: Yield 84%. White solid. ^{1}H NMR (400 MHz, $CDCl_3$): δ 7.77 (d, *J* = 8.0 Hz, 2H), 7.50 (m, 1H), 7.41 (m, 2H), 7.28 (s, 2H), 7.19 (m, 2H), 7.13 (m, 4H) (phenyl−**H**), 3.42 (s, 2H) (C**H**$_2$), 2.27 (s, 3H) (C**H**$_3$); ^{11}B {^{1}H} NMR (128 MHz, $CDCl_3$): δ −3.0 (3B), −4.6 (1B), −11.2 (2B), −13.1 (4B); ^{13}C NMR (100 MHz, $CDCl_3$): δ 137.1, 135.5, 131.5, 130.9, 130.7, 130.6, 129.3, 129.2, 127.7 (phenyl−**C**), 83.5, 82.0 (cage−**C**), 43.7 (**C**H$_2$), 21.5 (**C**H$_3$). IR (neat, cm^{-1}): 2571. HRMS (ESI, positive mode): *m/z* calcd for $C_{23}B_{10}NH_{32}$ [M+H$^+$]: 430.3530. Found: 430.3531.

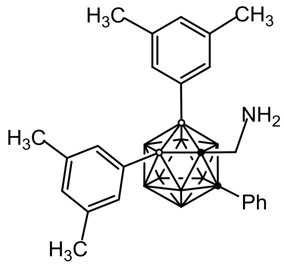

10: Yield 80%. White solid. ^{1}H NMR (400 MHz, $CDCl_3$): δ 7.77 (d, *J* = 8.0 Hz, 2H), 7.47 (m, 1H), 7.42 (m, 2H), 7.05 (s, 4H), 6.94 (s, 2H) (phenyl−**H**), 3.39 (s, 2H) (C$\mathbf{H}_2$), 2.21 (s, 6H) (C$\mathbf{H}_3$); ^{11}B {^{1}H} NMR (128 MHz, $CDCl_3$): δ −3.0 (3B), −4.6 (1B), −11.2 (2B), −13.2 (4B); ^{13}C NMR (100 MHz, $CDCl_3$): δ 136.9, 132.5, 130.8, 130.7, 130.1, 129.2 (phenyl−**C**), 83.6, 82.0 (cage−**C**), 43.8 (**C**H_2), 21.3 (**C**H_3). IR (neat, cm^{-1}): 2573. HRMS (ESI, positive mode): *m/z* calcd for $C_{25}B_{10}NH_{36}$ [M+H^{+}]: 458.3851. Found: 458.3868.

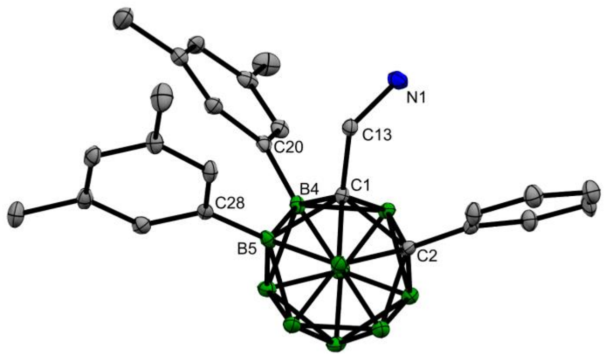

Figure S7. Molecular structure of compound **10** (ellipsoids at 30% probability and H atoms omitted for clarity). Selected bond distances [Å]: C1–C2 1.694(4), B4–C20 1.569(4), B5–C28 1.578(4), C1–C13 1.530(4), C13–N1 1.448(4).

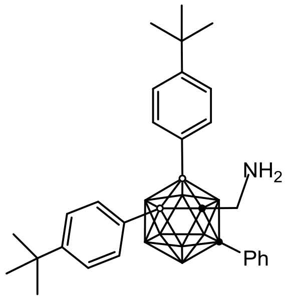

11: Yield 82%. Colorless oil. ^{1}H NMR (400 MHz, $CDCl_3$): δ 7.76 (d, J = 8.0 Hz, 2H), 7.47 (m, 1H), 7.42 (m, 2H), 7.38 (d, J = 8.0 Hz, 4H), 7.26 (d, J = 8.0 Hz, 4H) (phenyl−**H**), 3.41 (s, 2H) (C**H**$_2$), 1.30 (s, 18H) (C**H**$_3$); ^{11}B {^{1}H} NMR (128 MHz, $CDCl_3$): δ −2.9 (3B), −4.6 (1B), −11.2 (2B), −13.0 (4B); ^{13}C NMR (100 MHz, $CDCl_3$): δ 151.4, 134.4, 130.9, 130.8, 129.1, 124.8 (phenyl−**C**), 83.5, 81.9 (cage−**C**), 43.7 (**C**H$_2$), 34.5, 31.2 (t-butyl). IR (neat, cm^{-1}): 2577. HRMS (ESI, positive mode): *m/z* calcd for $C_{29}B_{10}NH_{44}$ [M+H$^+$]: 514.4478. Found: 514.4471.

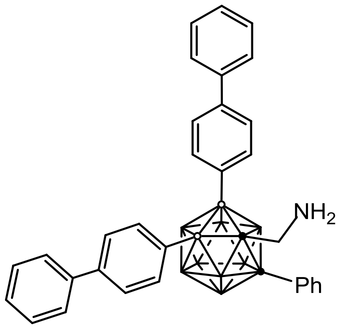

12: Yield 62%. White solid. ^{1}H NMR (400 MHz, $CDCl_3$): δ 7.79 (d, J = 8.0 Hz, 2H), 7.58-7.60 (m, 4H), 7.49-7.55 (m, 9H), 7.40-7.45 (m, 6H), 7.33 (m, 2H) (phenyl−**H**), 3.50 (s, 2H) (C**H**$_2$); ^{11}B {^{1}H} NMR (128 MHz, $CDCl_3$): δ −3.0 (3B), −4.2 (1B), −11.3 (2B), −12.9 (4B); ^{13}C NMR (100 MHz, $CDCl_3$): δ 141.2, 140.6, 135.0, 131.0, 130.7, 130.6, 129.2, 128.8, 127.4, 127.0, 126.5 (phenyl−**C**), 83.4, 82.2 (cage−**C**), 43.7 (**C**H$_2$). IR (neat, cm^{-1}): 2571. HRMS (ESI, positive mode): *m/z* calcd for $C_{33}B_{10}NH_{36}$ [M+H$^+$]: 554.3845. Found: 554.3851.

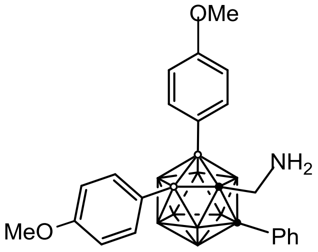

13: Yield 78%. White solid. ^{1}H NMR (400 MHz, $CDCl_3$): δ 7.76 (d, J = 8.0 Hz, 2H), 7.48 (m, 1H), 7.41 (m, 2H), 7.36 (d, J = 8.0 Hz, 4H), 7.26 (d, J = 8.0 Hz, 4H), 6.81 (d, J = 8.0 Hz, 4H) (phenyl−**H**), 3.79 (s, 6H) (C**H**$_3$), 3.39 (s, 2H) (C**H**$_2$); ^{11}B {^{1}H} NMR

(128 MHz, $CDCl_3$): δ −3.0 (3B), −4.7 (1B), −11.4 (2B), −13.4 (4B); ^{13}C NMR (100 MHz, $CDCl_3$): δ 160.0, 135.9, 130.9, 130.8, 130.7, 129.2, 113.5 (phenyl−**C**), 83.1, 81.9 (cage−**C**), 55.0 (**C**H_3), 43.6 (**C**H_2). IR (neat, cm^{-1}): 2575. HRMS (ESI, positive mode): *m/z* calcd for $C_{23}B_{10}NO_2H_{32}$ [M+H^+]: 462.3430. Found: 462.3432.

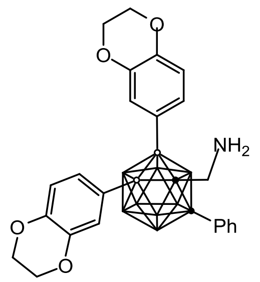

14: Yield 80%. White solid. ^{1}H NMR (400 MHz, $CDCl_3$): δ 7.74 (d, *J* = 8.0 Hz, 2H), 7.47 (m, 1H), 7.40 (m, 2H), 7.02 (s, 1H), 7.01 (s, 1H), 6.85-6.87 (m, 2H), 6.75 (s, 1H), 6.73 (s, 1H) (phenyl−**H**), 4.21-4.26 (m, 8H), 3.39 (s, 2H) (C**H**$_2$); ^{11}B {^{1}H} NMR (128 MHz, $CDCl_3$): δ −3.1 (3B), −4.7 (1B), −11.4 (2B), −13.2 (4B); ^{13}C NMR (100 MHz, $CDCl_3$): δ 144.1, 143.1, 130.9, 130.7, 130.6, 129.2, 127.8, 123.4, 116.9 (phenyl−**C**), 83.2, 81.7 (cage−**C**), 64.5, 64.3, 43.7 (**C**H_2). IR (neat, cm^{-1}): 2573. HRMS (ESI, positive mode): *m/z* calcd for $C_{25}B_{10}NO_4H_{32}$ [M+H^+]: 518.3329. Found: 518.3327.

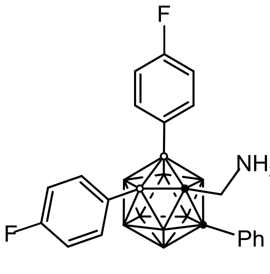

15: Yield 74%. Colorless oil. ^{1}H NMR (400 MHz, $CDCl_3$): δ 7.76 (d, *J* = 8.0 Hz, 2H), 7.50 (m, 1H), 7.37-7.43 (m, 6H), 6.96 (t, *J* = 8.0 Hz, 4H) (phenyl−**H**), 3.39 (s, 2H) (C**H**$_2$); ^{11}B {^{1}H} NMR (128 MHz, $CDCl_3$): δ −2.9 (1B), −3.6 (2B), −4.4 (1B), −11.2 (2B), −12.9 (4B); ^{13}C NMR (100 MHz, $CDCl_3$): δ 163.4 (d, J_{C-F} = 247 Hz), 136.3 (d, J_{C-F} = 8 Hz), 131.0, 130.7, 130.5, 129.2, 115.0 (d, J_{C-F} = 20 Hz) (phenyl−**C**), 83.1, 82.1 (cage−**C**), 43.6 (**C**H_2); ^{19}F NMR (376 MHz, $CDCl_3$): δ −112.7. IR (neat, cm^{-1}):

2569. HRMS (ESI, positive mode): *m/z* calcd for $C_{21}B_{10}NF_2H_{26}$ [M+H$^+$]: 438.3031. Found: 438.3033.

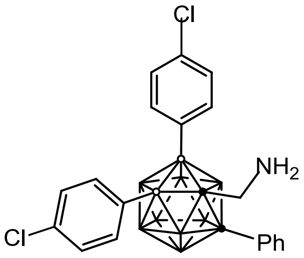

16: Yield 71%. White solid. ^{1}H NMR (400 MHz, CDCl$_3$): δ 7.75 (d, *J* = 8.0 Hz, 2H), 7.50 (m, 1H), 7.43 (m, 2H), 7.34 (d, *J* = 8.0 Hz, 4H), 7.25 (d, *J* = 8.0 Hz, 4H) (phenyl−**H**), 3.38 (s, 2H) (C**H**$_2$); ^{11}B {^{1}H} NMR (128 MHz, CDCl$_3$): δ −3.0 (2B), −3.9 (2B), −11.2 (2B), −12.8 (4B); ^{13}C NMR (100 MHz, CDCl$_3$): δ 135.7, 135.2, 131.1, 130.7, 130.4, 129.3, 128.2 (phenyl−**C**), 83.1, 82.2 (cage−**C**), 43.6 (**C**H$_2$). IR (neat, cm^{-1}): 2579. HRMS (ESI, positive mode): *m/z* calcd for $C_{21}B_{10}NCl_2H_{26}$ [M+H$^+$]: 470.2440. Found: 470.2439.

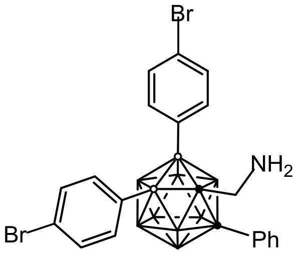

17: Yield 65%. White solid. ^{1}H NMR (400 MHz, CDCl$_3$): δ 7.75 (d, *J* = 8.0 Hz, 2H), 7.49 (m, 1H), 7.43 (d, *J* = 8.0 Hz, 2H), 7.39 (d, *J* = 8.0 Hz, 4H), 7.27 (d, *J* = 8.0 Hz, 4H) (phenyl−**H**), 3.38 (s, 2H) (C**H**$_2$); ^{11}B {^{1}H} NMR (128 MHz, CDCl$_3$): δ −3.5 (4B), −11.0 (2B), −12.6 (4B); ^{13}C NMR (100 MHz, CDCl$_3$): δ 136.0, 131.2, 131.1, 130.7, 130.4, 129.3, 123.7 (phenyl−**C**), 83.0, 82.2 (cage−**C**), 43.6 (**C**H$_2$). IR (neat, cm^{-1}): 2575. HRMS (ESI, positive mode): *m/z* calcd for $C_{21}B_{10}NBr_2H_{26}$ [M+H$^+$]: 560.1409. Found: 560.1418.

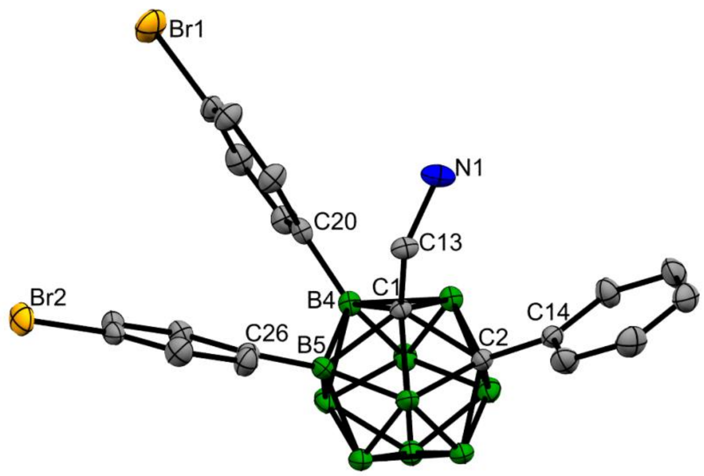

Figure S8. Molecular structure of compound **17** (ellipsoids at 30% probability and H atoms omitted for clarity). Selected bond distances [Å]: C1–C2 1.698(2), B4–C20 1.586(3), B5–C26 1.586(2), C1–C13 1.537(2), C13–N1 1.441(2).

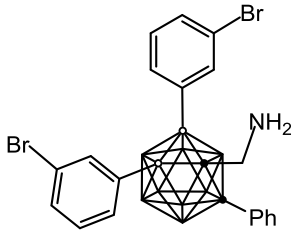

18: Yield 70%. White solid. ^{1}H NMR (400 MHz, $CDCl_3$): δ 7.76 (d, *J* = 8.0 Hz, 2H), 7.62 (s, 2H), 7.50 (m, 1H), 7.42-7.45 (m, 4H), 7.27 (s, 2H), 7.11 (t, *J* = 8.0 Hz, 2H) (phenyl−**H**), 3.40 (s, 2H) (C**H**$_2$); ^{11}B {^{1}H} NMR (128 MHz, $CDCl_3$): δ −2.8 (2B), −4.0 (2B), −11.0 (2B), −12.4 (4B); ^{13}C NMR (100 MHz, $CDCl_3$): δ 137.2, 132.8, 131.7, 130.7, 130.3, 129.5, 129.3, 122.7 (phenyl−**C**), 83.2, 82.1 (cage−**C**), 43.6 (**C**H$_2$). IR (neat, cm^{-1}): 2577. HRMS (ESI, positive mode): *m/z* calcd for $C_{21}B_{10}NBr_2H_{26}$ [$M+H^+$]: 560.1409. Found: 560.1416.

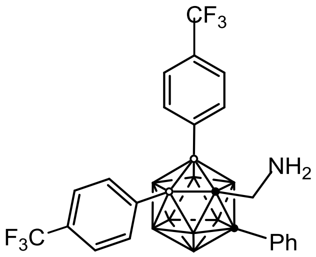

19: Yield 76%. White solid. ^{1}H NMR (400 MHz, $CDCl_3$): δ 7.77 (d, *J* = 8.0 Hz, 2H), 7.50-7.56 (m, 9H), 7.44 (m, 2H) (phenyl−**H**), 3.44 (s, 2H) (C$\mathbf{H}_2$); ^{11}B {^{1}H} NMR (128 MHz, $CDCl_3$): δ −2.9 (2B), −3.9 (2B), −11.1 (2B), −12.5 (4B); ^{13}C NMR (100 MHz, $CDCl_3$): δ 134.7, 131.3, 130.9, 130.7, 130.6, 130.2, 129.3, 124.6 (q, J_{C-F} = 4 Hz) (phenyl−**C**), 124.08 (q, J_{C-F} = 271 Hz) (**C**F_3), 83.2, 82.4 (cage−**C**), 43.6 (**C**H_2). IR (neat, cm^{-1}): 2571. HRMS (ESI, positive mode): *m/z* calcd for $C_{23}B_{10}NF_6H_{26}$ [M+H$^+$]: 538.2976. Found: 538.2967.

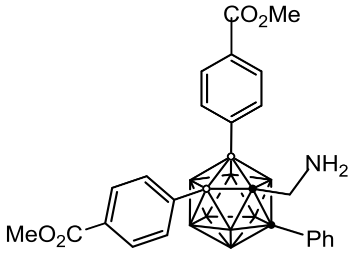

20: Yield 70%. Colorless oil. ^{1}H NMR (400 MHz, $CDCl_3$): δ 7.82 (d, *J* = 8.0 Hz, 4H), 7.72 (d, *J* = 8.0 Hz, 2H), 7.41 (d, *J* = 8.0 Hz, 4H), 7.32-7.37 (m, 3H) (phenyl−**H**), 3.90 (s, 6H) (OC$\mathbf{H}_3$), 3.44 (s, 2H) (C$\mathbf{H}_2$); ^{11}B {^{1}H} NMR (128 MHz, $CDCl_3$): δ −3.3 (4B), −11.1 (2B), −12.5 (4B); ^{13}C NMR (100 MHz, $CDCl_3$): δ 166.8 (**C**=O), 134.5, 131.8, 131.4, 130.9, 129.8, 129.3, 128.6 (phenyl−**C**), 85.6, 77.2 (overlapped with $CHCl_3$) (cage−**C**), 52.3 (O**C**H_3), 40.0 (**C**H_2). IR (neat, cm^{-1}): 2579, 1722. HRMS (ESI, positive mode): *m/z* calcd for $C_{25}B_{10}NO_4H_{32}$ [M+H$^+$]: 518.3320. Found: 518.3327.

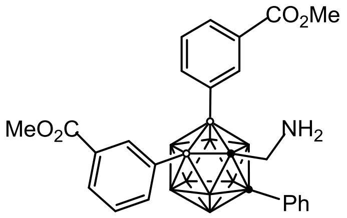

21: Yield 62%. Colorless oil. ^{1}H NMR (400 MHz, $CDCl_3$): δ 8.12 (s, 2H), 7.99 (d, *J* = 8.0 Hz, 2H), 7.79 (d, *J* = 8.0 Hz, 2H), 7.61 (d, *J* = 8.0 Hz, 4H), 7.51 (m, 1H), 7.44 (m, 2H), 7.32 (t, *J* = 8.0 Hz, 2H) (phenyl−**H**), 3.84 (s, 6H) (OC$\mathbf{H}_3$), 3.44 (s, 2H) (C$\mathbf{H}_2$); ^{11}B {^{1}H} NMR (128 MHz, $CDCl_3$): δ −3.4 (4B), −10.9 (2B), −12.3 (4B); ^{13}C NMR

(100 MHz, $CDCl_3$): δ 167.0 (**C**=O), 139.1, 135.1, 131.1, 130.8, 130.4, 129.7, 129.2, 128.0 (phenyl−**C**), 83.4, 82.0 (cage−**C**), 52.0 (O**C**H_3), 43.7 (**C**H_2). IR (neat, cm^{-1}): 2579, 1720. HRMS (ESI, positive mode): *m/z* calcd for $C_{25}B_{10}NO_4H_{32}$ [M+H$^+$]: 518.3320. Found: 518.3321.

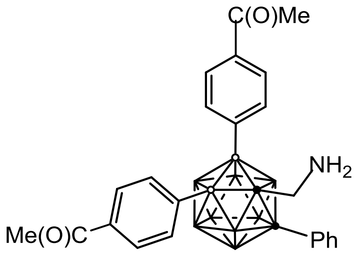

22: Yield 50%. White solid. ^{1}H NMR (400 MHz, $CDCl_3$): δ 7.82 (d, *J* = 8.0 Hz, 4H), 7.77 (d, *J* = 8.0 Hz, 2H), 7.65 (m, 1H), 7.52 (m, 4H), 7.44 (m, 2H) (phenyl−**H**), 3.45 (s, 2H) (C**H**$_2$), 2.58 (s, 6H) (C**H**$_3$),; ^{11}B {^{1}H} NMR (128 MHz, $CDCl_3$): δ −3.2 (4B), −10.9 (2B), −12.4 (4B); ^{13}C NMR (100 MHz, $CDCl_3$): δ 198.0 (**C**=O), 167.0, 139.1, 135.1, 131.1, 130.8, 130.4, 129.7, 129.2, 128.0 (phenyl−**C**), 83.3, 82.4 (cage−**C**), 43.7 (**C**H_2), 26.6 (**C**H_3). IR (neat, cm^{-1}): 2579, 1732. HRMS (ESI, positive mode): *m/z* calcd for $C_{25}B_{10}NO_2H_{32}$ [M+H$^+$]: 486.3431. Found: 486.3432.

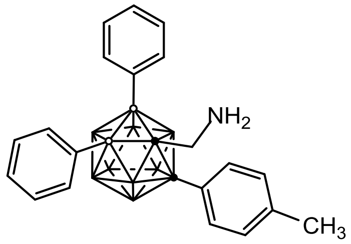

23: Yield 81%. Colorless oil. ^{1}H NMR (400 MHz, $CDCl_3$): δ 7.64 (d, *J* = 8.0 Hz, 2H), 7.43 (m, 4H), 7.31 (m, 2H), 7.20-7.26 (m, 6H) (phenyl−**H**), 3.44 (s, 2H) (C**H**$_2$), 2.38 (s, 3H) (C**H**$_3$); ^{11}B {^{1}H} NMR (128 MHz, $CDCl_3$): δ −2.8 (3B), −4.2 (1B), −11.0 (2B), −12.7 (4B); ^{13}C NMR (100 MHz, $CDCl_3$): δ 141.4, 134.5, 130.6, 129.9, 128.5, 127.8 (phenyl−**C**), 83.5, 82.3 (cage−**C**), 43.7 (**C**H_2), 21.0 (**C**H_3). IR (neat, cm^{-1}): 2577. HRMS (ESI, positive mode): *m/z* calcd for $C_{22}B_{10}NH_{30}$ [M+H$^+$]: 416.3376. Found: 416.3380.

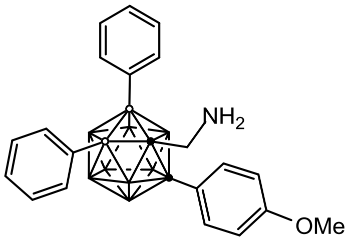

24: Yield 80%. Colorless oil. ^{1}H NMR (400 MHz, $CDCl_3$): δ 7.66 (d, *J* = 8.0 Hz, 2H), 7.42 (m, 4H), 7.30 (m, 2H), 7.23 (m, 4H), 6.89 (d, *J* = 8.0 Hz, 2H) (phenyl−**H**), 3.83 (s, 3H) (OC**H**$_3$), 3.43 (s, 2H) (C**H**$_2$); ^{11}B {^{1}H} NMR (128 MHz, $CDCl_3$): δ −3.2 (3B), −4.5 (1B), −11.2 (2B), −12.8 (4B); ^{13}C NMR (100 MHz, $CDCl_3$): δ 161.5, 134.5, 132.1, 128.5, 127.8, 122.7, 114.4 (phenyl−**C**), 83.6, 82.6 (cage−**C**), 55.4 (O**C**H$_3$), 43.5 (**C**H$_2$). IR (neat, cm^{-1}): 2575. HRMS (ESI, positive mode): *m/z* calcd for $C_{22}B_{10}NOH_{30}$ [M+H$^+$]: 432.3325. Found: 432.3333.

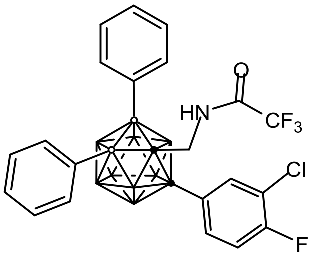

25: Yield 55%. White solid. ^{1}H NMR (400 MHz, $CDCl_3$): δ 7.69 (m, 1H), 7.52 (m, 1H), 7.47 (m, 4H), 7.39 (m, 2H), 7.32 (m, 4H), 7.18 (t, *J* = 8.0 Hz, 1H) (phenyl−**H**), 5.55 (br m, 1H) (N**H**), 4.11 (d, *J* = 2.4 Hz, 2H) (C**H**$_2$); ^{11}B {^{1}H} NMR (128 MHz, $CDCl_3$): δ −2.7 (4B), −10.0 (2B), −12.8 (4B); ^{13}C NMR (100 MHz, $CDCl_3$): δ 160.1 (q, J_{C-F} = 255 Hz) (**C**=O), 155.5 (d, J_{C-F} = 38 Hz), 134.5, 133.6, 131.1 (d, J_{C-F} = 8 Hz), 129.5, 128.5, 126.8 (d, J_{C-F} = 3 Hz), 122.4 (d, J_{C-F} = 18 Hz), 117.4 (d, J_{C-F} = 22 Hz) (phenyl−**C**), 114.9 (q, J_{C-F} = 286 Hz) (**C**F$_3$), 83.0, 78.8 (cage−**C**), 38.3 (**C**H$_2$); ^{19}F NMR (376 MHz, $CDCl_3$): δ −76.8, −109.6. IR (neat, cm^{-1}): 2581, 1733. HRMS (ESI, negative mode): *m/z* calcd for $C_{23}B_{10}NOClF_4H_{23}$ [M−H]$^-$: 549.2413. Found: 549.2402.

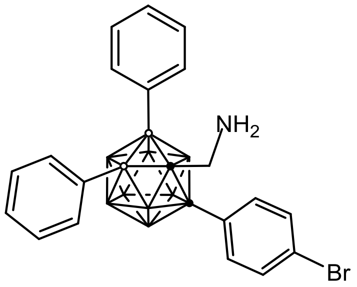

26: Yield 62%.White solid. ^{1}H NMR (400 MHz, $CDCl_3$): δ 7.63 (d, *J* = 8.0 Hz, 2H), 7.55 (d, *J* = 8.0 Hz, 2H), 7.42 (m, 4H), 7.31 (m, 2H), 7.24 (m, 4H) (phenyl−**H**), 3.44 (s, 2H) (C**H**$_2$); ^{11}B {^{1}H} NMR (128 MHz, $CDCl_3$): δ −2.9 (3B), −4.4 (1B), −11.0 (2B), −13.1 (4B); ^{13}C NMR (100 MHz, $CDCl_3$): δ 134.5, 132.3, 132.2, 129.9, 128.6, 127.9, 125.9 (phenyl−**C**), 83.3, 81.1 (cage−**C**), 43.6 (**C**H$_2$). IR (neat, cm^{-1}): 2577. HRMS (ESI, positive mode): *m/z* calcd for $C_{21}B_{10}NBrH_{27}$ [M+H$^+$]: 481.2325. Found: 481.2319.

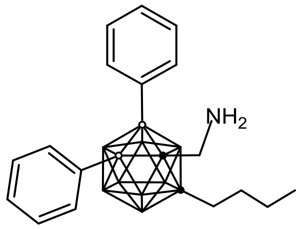

27: Yield 75%. Colorless oil. ^{1}H NMR (400 MHz, $CDCl_3$): δ 7.39 (d, *J* = 8.0 Hz, 4H), 7.30 (m, 2H), 7.23 (m, 4H) (phenyl−**H**), 3.77 (s, 2H) (C**H**$_2$), 2.29 (t, *J* = 8.0 Hz, 2H), 1.61 (m, 2H), 1.37 (m, 2H), 0.94 (t, *J* = 8.0 Hz, 3H) (n-butyl); ^{11}B {^{1}H} NMR (128 MHz, $CDCl_3$): δ −3.6 (3B), −5.6 (1B), −11.0 (4B), −14.5 (2B); ^{13}C NMR (100 MHz, $CDCl_3$): δ 134.6, 128.4, 127.8 (phenyl−**C**), 79.5, 77.2 (overlapped with $CHCl_3$) (cage−**C**), 43.5 (CH_2), 34.9, 31.8, 22.5, 13.7 (n-butyl). IR (neat, cm^{-1}): 2569. HRMS (ESI, positive mode): *m/z* calcd for $C_{19}B_{10}NH_{32}$ [M+H$^+$]: 382.3535. Found: 382.3532.

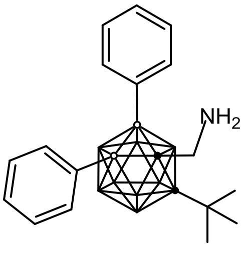

28: Yield 83%. White solid. ^{1}H NMR (400 MHz, $CDCl_3$): δ 7.45 (d, *J* = 8.0 Hz, 4H), 7.29 (m, 2H), 7.22-7.26 (m, 4H) (phenyl−**H**), 3.85 (s, 2H) (C$\mathbf{H}_2$), 1.44 (s, 9H) (C$\mathbf{H}_3$); ^{11}B {^{1}H} NMR (128 MHz, $CDCl_3$): δ −2.9 (3B), −4.8 (1B), −10.4 (2B), −12.6 (2B), −14.2 (2B); ^{13}C NMR (100 MHz, $CDCl_3$): δ 134.6, 128.4, 127.8 (phenyl−**C**), 93.7, 85.4 (cage−**C**), 44.4 (**C**H_2), 39.6, 33.6 (t-butyl). IR (neat, cm^{-1}): 2567. HRMS (ESI, positive mode): *m/z* calcd for $C_{19}B_{10}NH_{32}$ [M+H$^+$]: 382.3534. Found: 382.3532.

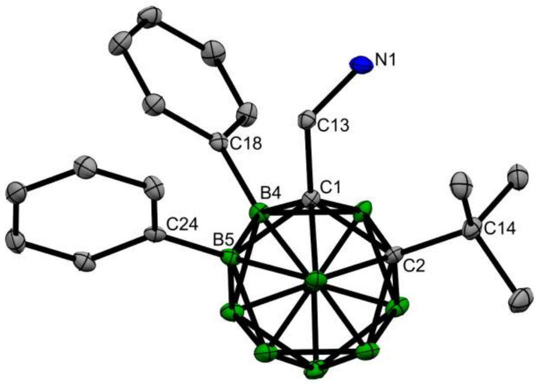

Figure S9. Molecular structure of compound **28** (ellipsoids at 30% probability and H atoms omitted for clarity). Selected bond distances [Å]: C1–C2 1.743(3), B4–C18 1.585(3), B5–C24 1.583(3), C1–C13 1.530(3), C13–N1 1.459(3).

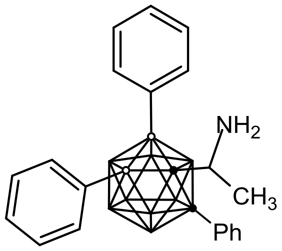

29: Yield 75%. White solid. ^{1}H NMR (400 MHz, $CDCl_3$): δ 7.81 (d, *J* = 8.0 Hz, 2H), 7.61 (d, *J* = 8.0 Hz, 2H), 7.45 (m, 3H), 7.37 (m, 2H), 7.28-7.34 (m, 4H), 7.22 (m, 2H) (phenyl−**H**), 3.94 (q, *J* = 6.8 Hz, 1H) (C**H**), 0.74 (d, *J* = 6.8 Hz, 3H) (C$\mathbf{H}_3$); ^{11}B {^{1}H} NMR (128 MHz, $CDCl_3$): δ −2.7 (3B), −4.2 (1B), −10.8 (2B), −12.3 (4B); ^{13}C NMR (100 MHz, $CDCl_3$): δ 134.6, 134.5, 132.0, 131.1, 130.6, 128.8, 128.5, 128.4, 128.0, 127.7 (phenyl−**C**), 87.1, 82.0 (cage−**C**), 50.4 (**C**H), 22.9 (**C**H_3). IR (neat, cm^{-1}): 2571.

HRMS (ESI, positive mode): *m/z* calcd for $C_{22}B_{10}NH_{30}$ [M+H$^+$]: 416.3376. Found: 416.3378.

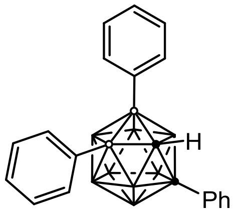

30: Yield 80%. White solid. ^{1}H NMR (400 MHz, $CDCl_3$): δ 7.61 (m, 2H), 7.43 (m, 1H), 7.38 (m, 2H), 7.28 (m, 4H), 7.21 (m, 2H), 7.15 (m, 4H) (phenyl−**H**), 4.41 (br s, 1H) (cage-C**H**); ^{11}B {^{1}H} NMR (128 MHz, $CDCl_3$): δ −3.5 (2B), −4.6 (1B), −5.5 (1B), −9.1 (2B), −11.8 (2B), −14.4 (2B); ^{13}C NMR (100 MHz, $CDCl_3$): δ 133.5, 132.8, 130.1, 129.0, 128.2, 127.9, 127.7 (phenyl−**C**), 75.0, 60.3 (cage−**C**). IR (neat, cm^{-1}): 3052, 2568. HRMS (EI): m/z calcd for $C_{20}B_{10}H_{24}$ [M$^+$]: 372.2885. Found: 372.2883.

2.5 Further transformations and scale up reaction

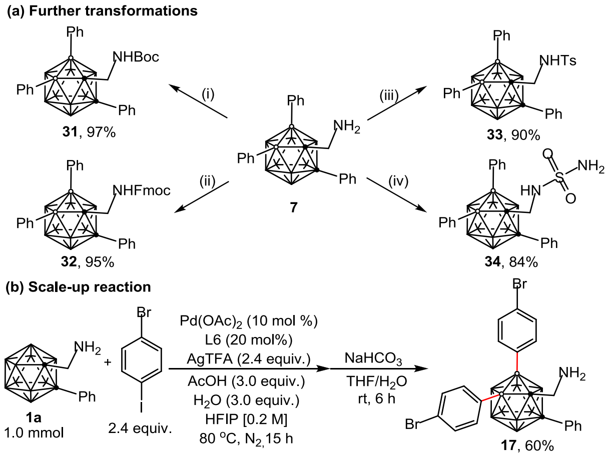

(i) Boc protection: **7** (0.1 mmol, 40 mg), $(Boc)_2O$ (4.0 equiv., 0.4 mmol, 88 mg), Et_3N (4.0 equiv., 0.4 mmol, 40 mg) and CH_2Cl_2 (4 mL) were added into a reaction tube (10 mL) and allowed to stir at room temperature for 8 h. Upon completion as monitored by TLC, the crude reaction mixture was concentrated under *vacuo* and purified on preparative TLC using hexanes/CH_2Cl_2 (v/v = 1/1) as the eluent to afford the desired product **31** in nearly quantitative yield (48 mg, yield: 97%).

(ii) Fmoc protection: **7** (0.1 mmol, 40 mg), Fmoc-Osu (4.0 equiv., 0.4 mmol, 136 mg), and CH_3CN (4 mL) were added into a reaction tube (10 mL) and allowed to stir at room temperature for 8 h. Upon completion as monitored by TLC, the crude reaction mixture was concentrated under *vacuo* and purified on preparative TLC using hexanes/CH_2Cl_2 (v/v = 1/1) as the eluent to afford the desired product **32** (62 mg, yield: 95%).

(iii) Tosyl protection: **7** (0.1 mmol, 40 mg), 4-toluene sulfonyl chloride (2.0 equiv., 0.2 mmol, 68 mg), Na_2CO_3 (2.0 equiv., 0.2 mmol, 23 mg), H_2O (1 mL) and THF (4 mL) were added into a reaction tube (10 mL) and allowed to stir at room temperature for 12 h. Upon completion as monitored by TLC, the crude reaction mixture was filtrated and the organic filtrate was concentrated under *vacuo* and purified by flash column chromatography using CH_2Cl_2 (v/v = 1/1) as the eluent to afford the desired product **33** (52 mg, yield: 90%).

(iv) Reaction with sulfamide: 7 (0.2 mmol, 40 mg), sulfamide (3.0 equiv., 0.6 mmol, 58 mg), Na_2CO_3 (2.0 equiv., 0.4 mmol, 45 mg) and 1,4-dioxane (4 mL) were added into a reaction tube (10 mL) and allowed to stir at 100 ^oC for 4 h. Upon completion, the crude reaction mixture was filtrated and the organic filtrate was concentrated under *vacuo* and purified by flash column chromatography using CH_2Cl_2/EtOAc (v/v = 2/1) as the eluent to afford the desired product **34** (80 mg, yield: 84%).

Scale up reaction:

A reaction tube (50 mL) with a magnetic stir bar was charged with **1a** (1.0 mmol, 250 mg), 1-bromo-4-iodobenzene (2.4 equiv. 2.4 mmol, 677 mg), $Pd(OAc)_2$ (10 mol %, 0.1 mmol, 23 mg), AgTFA (2.4 equiv. 2.4 mmol, 530 mg), glyoxylic acid monohydrate (20 mol%, 0. 2 mmol, 18 mg), AcOH (3.0 equiv., 3.0 mmol, 180 mg), H_2O (3.0 equiv., 3.0 mmol, 54 mg) and HFIP (5 mL). The reaction tube was sealed under N_2 atmosphere and allowed to stir at ambient temperature for 30 minutes, then heated to 80 ^oC for 15 hours. Upon completion, the reaction mixture was cooled to room temperature and diluted with 10 mL of THF and 10 mL of water. Na_2CO_3 (6 mmol, 660 mg) was added into the mixture and stirred for 4 h at room temperature.

The mixture was filtered through a silica gel plug and concentrated under *vacuo* to remove the organic solvent. The residue was then extracted with CH_2Cl_2 (10 mL × 3) from water. The combined organic extracts were concentrated under *vacuo*. The crude reaction mixture was purified on flash column chromatography using hexanes/CH_2Cl_2 (v/v = 2/1 to 1/2) as the eluent to obtain compound **17** as a white solid (335 mg, yield: 60%).

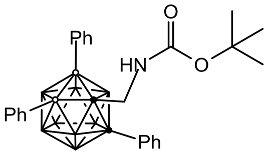

31: Yield 97%. White solid. ^{1}H NMR (400 MHz, $CDCl_3$): δ 7.72 (d, *J* = 8.0 Hz, 2H), 7.38-7.47 (m, 7H), 7.33 (m, 2H), 7.24-7.28 (m, 4H) (phenyl−**H**), 3.95 (s, 2H) (C**H**$_2$), 1.16 (s, 9H) (C**H**$_3$); ^{11}B {^{1}H} NMR (128 MHz, $CDCl_3$): δ −3.0 (3B), −4.20 (1B), −10.8 (2B), −13.0 (4B); ^{13}C NMR (100 MHz, $CDCl_3$): δ 153.5 (**C**=O), 134.6, 131.0, 130.9, 130.3, 129.0, 128.7, 128.0 (phenyl−**C**), 85.6, 79.8 (cage−**C**), 60.4 (**C**Me$_3$), 40.8 (**C**H$_2$), 28.1 (**C**H$_3$). IR (neat, cm^{-1}): 2579, 1720. HRMS (ESI, negative mode): *m/z* calcd for $C_{26}B_{10}NO_2H_{34}$ [M−H]$^-$: 500.3598. Found: 500.3598.

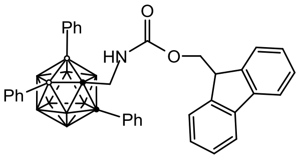

32: Yield 95%. White solid. ^{1}H NMR (400 MHz, $CDCl_3$): δ 7.76 (d, *J* = 8.0 Hz, 2H), 7.69 (d, *J* = 8.0 Hz, 2H), 7.50 (d, *J* = 8.0 Hz, 4H), 7.35-7.43 (m, 5H), 7.28-7.32 (m, 10H) (phenyl−**H**), 4.20 (t, *J* = 6.0 Hz, 1H) (C**H**), 4.04 (d, *J* = 6.0 Hz, 2H) (OC**H**$_2$), 3.94 (s, 2H) (C**H**$_2$); ^{11}B {^{1}H} NMR (128 MHz, $CDCl_3$): δ −3.2 (4B), −10.8 (2B), −12.9 (4B); ^{13}C NMR (100 MHz, $CDCl_3$): δ 154.1 (**C**=O), 143.6, 141.2, 134.7, 131.1, 130.1, 128.9, 128.1, 127.8, 127.0, 125.0, 120.0 (phenyl−**C**), 84.2, 79.8 (cage−**C**), 67.0 (O**C**H$_2$), 46.7 (**C**H$_2$), 41.0 (**C**H). IR (neat, cm^{-1}): 2579, 1736. HRMS (ESI, positive mode): *m/z* calcd for $C_{36}B_{10}NO_2H_{36}Na$ [M+Na$^+$]: 646.3720. Found: 646.3727.

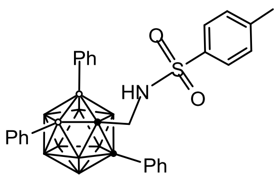

33: Yield 90%. White solid. ^{1}H NMR (400 MHz, $CDCl_3$): δ 7.71 (d, *J* = 8.0 Hz, 2H), 7.51 (t, *J* = 8.0 Hz, 1H), 7.41 (t, *J* = 8.0 Hz, 2H), 7.31 (t, *J* = 8.0 Hz, 2H), 7.23-7.26 (m, 5H), 7.14-7.16 (m, 7H) (phenyl−**H**), 3.74 (t, *J* = 6.4 Hz, 1H) (N**H**), 3.51 (d, *J* = 6.4 Hz, 2H) (C**H**$_2$), 2.38 (s, 3H) (C**H**$_3$); ^{11}B {^{1}H} NMR (128 MHz, $CDCl_3$): δ −2.0 (4B), −9.4 (2B), −11.8 (4B); ^{13}C NMR (100 MHz, $CDCl_3$): δ 143.9, 135.0 134.5, 131.2, 131.1, 129.6, 129.5, 129.1, 128.9, 128.1, 126.7 (phenyl−**C**), 100.0, 84.2 (cage−**C**), 43.3 (**C**H$_2$), 21.5 (**C**H$_3$). IR (neat, cm^{-1}): 2571. HRMS (ESI, positive mode): *m/z* calcd for $C_{28}B_{10}NO_2SH_{33}Na$ [M+Na$^+$]: 578.3127. Found: 578.3133.

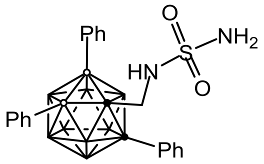

34: Yield 84%. White solid. ^{1}H NMR (400 MHz, $CDCl_3$): δ 7.77 (d, *J* = 8.0 Hz, 2H), 7.49 (m, 1H), 7.41-7.46 (m, 6H), 7.35 (m, 2H), 7.25-7.29 (m, 4H) (phenyl−**H**), 3.82 (d, *J* = 7.2 Hz, 2H) (C**H**$_2$), 3.64 (t, *J* = 7.2 Hz, 1H) (N**H**), 3.44 (s, 2H) (N**H**$_2$); ^{11}B {^{1}H} NMR (128 MHz, $CDCl_3$): δ −3.0 (4B), −10.6 (2B), −12.6 (4B); ^{13}C NMR (100 MHz, $CDCl_3$): δ 134.6, 131.3 131.2, 130.2, 129.3, 129.1, 128.2 (phenyl−**C**), 82.9, 77.3 (overlapped with $CHCl_3$) (cage−**C**), 43.4 (**C**H$_2$). IR (neat, cm^{-1}): 2573. HRMS (ESI, negative mode): *m/z* calcd for $C_{21}B_{10}N_2O_2SH_{27}$ [M−H]$^-$: 479.2806. Found: 479.2801.

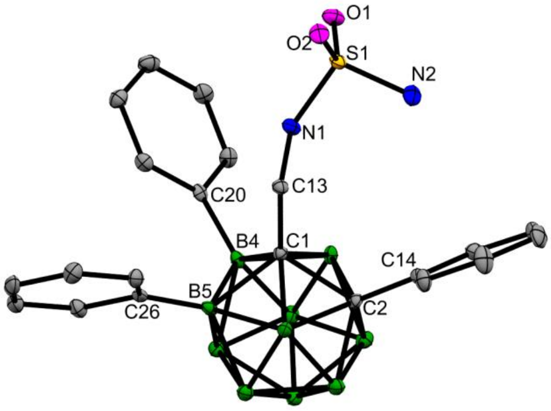

Figure S10. Molecular structure of compound **34** (ellipsoids at 30% probability and H atoms omitted for clarity)**.** Selected bond distances [Å]: C1–C2 1.723(4), B4–C20 1.579(4), B5–C26 1.571(4), C1–C13 1.529(3), C13–N1 1.452(3), S1–N1 1.610(2), S1–N2 1.592(3).

3. References

(S1) K. Ohta, T. Goto, Y. Endo, *Inorg. Chem.* 2005, **44**, 23.

(S2) C. Tang, Z. Xie, *Angew. Chem. Int. Ed.* 2015, **54**, 7662.

(S3) C. Viñas, R. Benakki, F. Teixidor, Casabó, J. *Inorg. Chem.* 1995, **34**, 3844.

(S4) L. I. Zakharkin, A. I. L'vov, *Zh. Obsh. Chim.* 1966, **36**, 761.

(S5) J. Brynda, P. Mader, V. Šícha, M. Fábry, K. Poncová, M. Bakardiev, B. Grüner, P. Cígler, P. Řezáčová, *Angew. Chem. Int. Ed*. 2013, **52**, 13760.

(S6) A. Kolleth, S. Christoph, S. Arseniyadis, J. Cossy, *Chem. Comm.* 2012, **48**, 10511.

(S7) A. Toppino, A. R. Genady, M. E. El-Zaria, J. Reeve, F. Mostofian, J. Kent, J. F. Valliant, *Inorg. Chem.* 2013, **52**, 8743.

(S8) G. M. Sheldrick, SADABS, *A Program for Empirical Absorption Correction*; University of Göttingen; Göttingen, Germany, 1998.

(S9) G. M. Sheldrick, SHELXL-97, *Program for the Refinement of Crystal Structures*; University of Göttingen; Göttingen, Germany, 1997.

(S10) A. D. Ryabov, *Chem. Rev.* 1990, **90**, 403.

(S11) J. Vicente, I. Saura-Llamas, M. G. Palin, P. G. Jones, M. C. Ram ŕezde Arellano, *Organometallics* 1997, **16**, 826.

4. Crystallographic data

Table S2. Crystallographic data of compounds **1a**, **3**, **4**, **6**, **10**, **17**, **28** and **34**.

	**1a**	**3**	**4**	**6**
Empirical	$C_9H_{19}B_{10}N$	$C_{25}H_{40}B_{20}N_2OPd$	$C_{34}H_{35}B_{10}NOPPd$	$C_{23}H_{26}B_{10}F_3NO$
Formula wt	249.35	707.19	719.10	497.55
Crystal system	Triclinic	Monoclinic	Monoclinic	Monoclinic
Space group	P-1	P 21/c	P2(1)/c	P2(1)/n
a/ Å	7.3461(17)	14.4456(7)	10.5093(6)	18.1161(10)
b/ Å	7.8073(19)	13.2757(6)	22.2105(13)	8.1173(5)
c/ Å	12.765(3)	19.9354(8)	15.7027(7)	18.6508(11)
α/deg	81.469(4)	90	90	90
β/deg	78.051(4)	90.292(2)	105.021(2)	113.681(2)
γ/deg	86.599(4)	90	90	90
V/ $Å^3$	708.0(3)	3823.1(3)	3540.0(3)	2511.7(3)
Z	2	4	4	4
ρ_{calcd} (g cm^{-3})	1.170	1.229	1.349	1.316
abs coeff (mm^{-1})	0.057	0.510	0.599	0.088
F(000)	260	1432	1460	1024
θ range (deg)	0.994/25.990	2.82/25.31	0.981/25.350	0.990/26.00
no. of rflns collected	5162	20022	20949	43718
no. of indep rflns	2763	6956	6359	4878
no. of obsd rflns (I>2σ(I))	1768	4963	4611	4335
GoF	0.977	1.051	1.000	1.118
R1/wR2 (I> 2σ(I))	0.0607/ 0.1541	0.0587/0.1535	0.0481/0.0984	0.0518/0.2127
R1/wR2 (all data)	0.0995/ 0.1751	0.0947/0.1740	0.0792/0.1090	0.0571/0.2324
largest peak/hole (e $Å^{-1}$)	0.259/−0.240	1.255/−0.366	0.673/−0.466	0.319/−0.355

	**10**	**17**	**28**	**34**
Empirical	$C_{25}H_{35}B_{10}N$	$C_{21}H_{21}B_{10}Br_2N$	$C_{19}H_{31}B_{10}N$	$C_{21}H_{28}B_{10}N_2O_2S$
Formula wt	457.64	555.31	381.55	480.61
Crystal system	Orthorhombic	Monoclinic	Monoclinic	Triclinic
Space group	P2(1)2(1)2(1)	P2(1)/n	C2/c	P-1
a/ Å	8.0494(7)	12.4232(5)	17.6118(12)	7.7385(6)
b/ Å	15.8108(12)	14.9794(6)	12.1001(9)	10.4142(8)
c/ Å	20.486(2)	13.9157(5)	20.3538(16)	15.9824(11)
α/deg	90	90	90	92.169(3)
β/deg	90	109.5090(10)	94.094(3)	102.976(2)

γ/deg	90	90	90	99.951(3)
V/ Å^3	2607.2(4)	2440.93(16)	4326.4(6)	1232.30(16)
Z	4	4	8	2
ρ_{calcd} (g cm^{-3})	1.166	1.511	1.172	1.295
abs coeff (mm^{-1})	0.061	3.333	0.060	0.156
F(000)	968	1096	1616	500
θ range (deg)	0.99/25.990	3.02/27.53	0.985/25.390	0.991/25.320
no. of rflns collected	12407	43196	15563	9903
no. of indep rflns	5069	5596	3919	4473
no. of obsd rflns ($I>2\sigma(I)$)	3572	4950	2609	3091
GoF	0.953	1.053	1.018	0.893
R1/wR2 ($I> 2\sigma(I)$)	0.0620/0.1271	0.0257/0.0619	0.0589/0.1165	0.0530/0.1212
R1/wR2 (all data)	0.1101/0.1429	0.0327/0.0653	0.1079/0.1303	0.0946/0.1362
largest peak/hole (e Å^{-1})	0.225/−0.255	0.598/−0.639	0.212/−0.228	0.293/−0.403

5. NMR Spectra

5.1 Starting materials

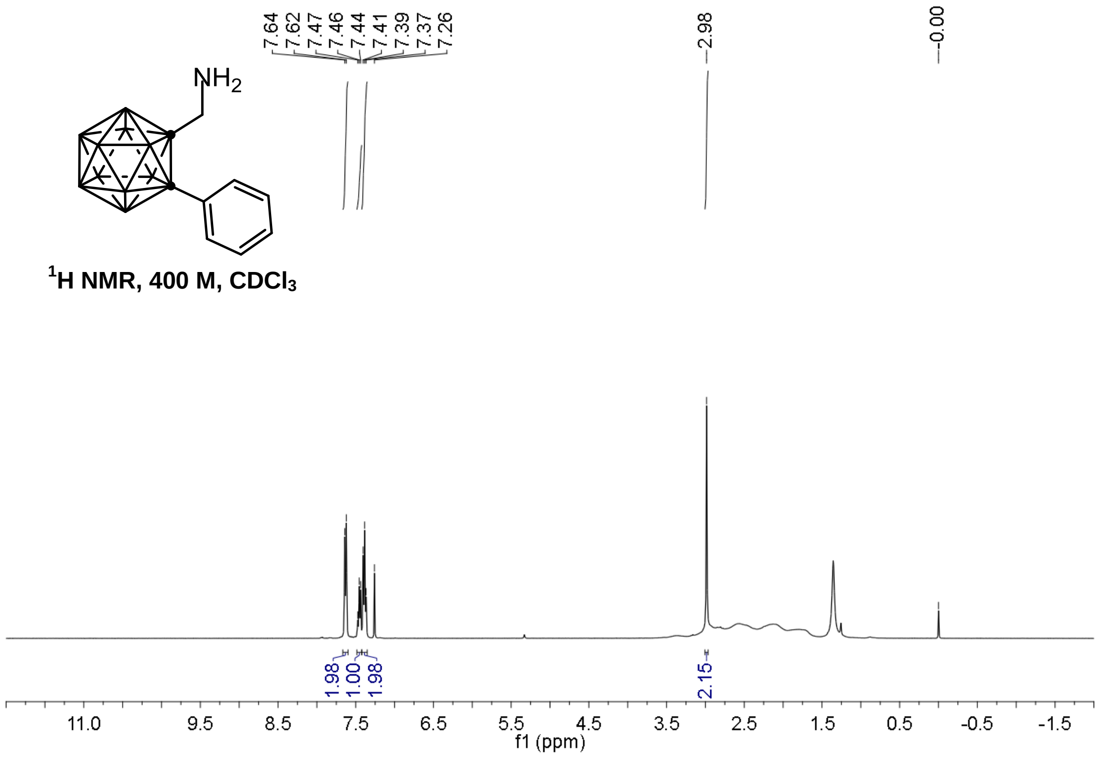

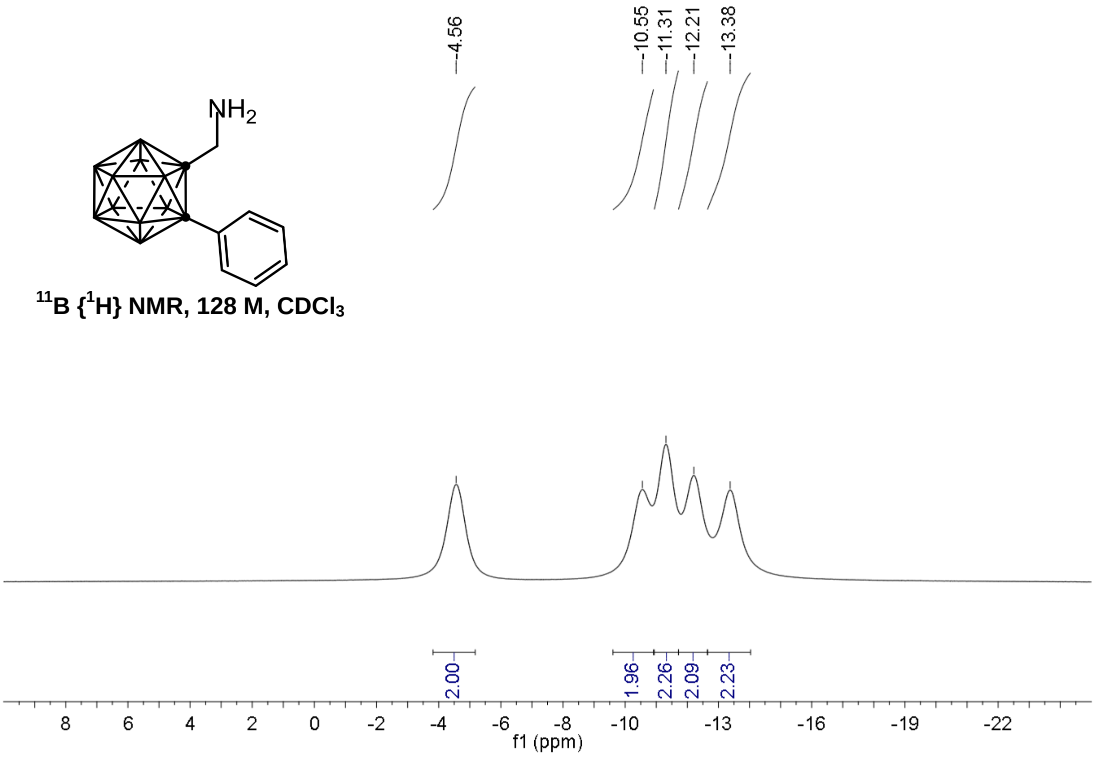

NH2
11B {1H} NMR, 128 M, CDCl3
-4.56
-10.55
-11.31
-12.21
-13.38
2.00
1.96
2.26
2.09
2.23
8 6 4 2 0 -2 -4 -6 -8 -10 -13 -16 -19 -22
f1 (ppm)

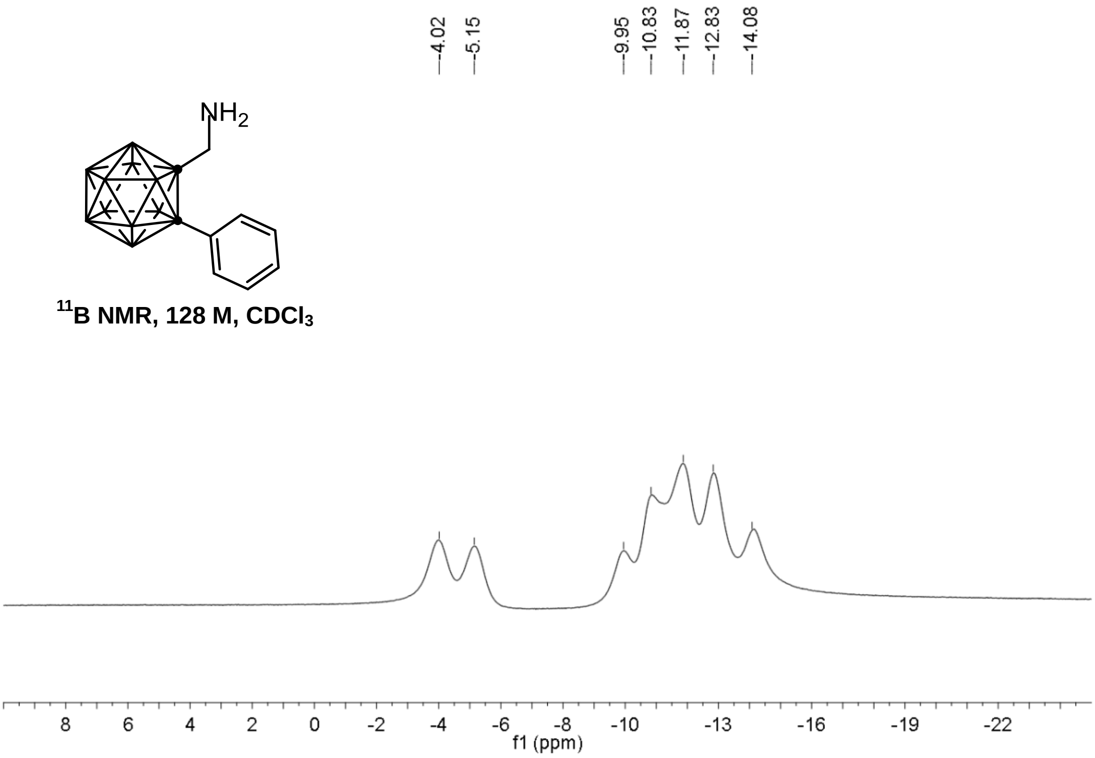

NH2
11B NMR, 128 M, CDCl3
-4.02
-5.15
-9.95
-10.83
-11.87
-12.83
-14.08
8 6 4 2 0 -2 -4 -6 -8 -10 -13 -16 -19 -22
f1 (ppm)

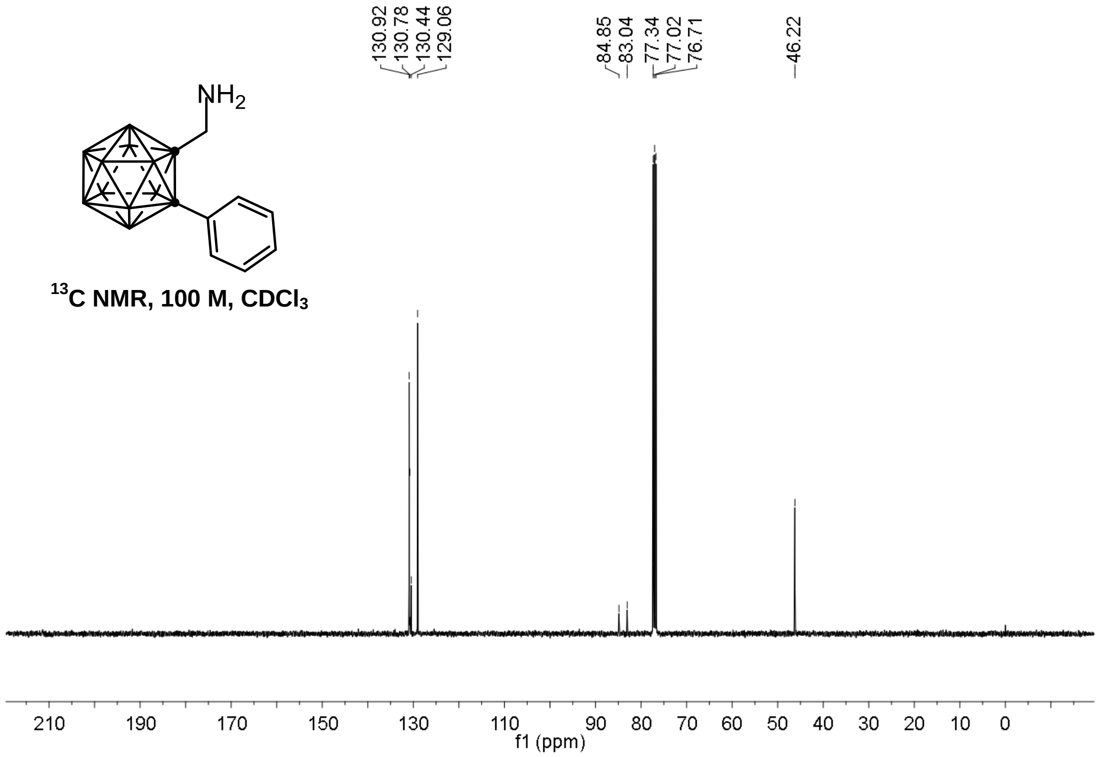
NH2
13C NMR, 100 M, CDCl3
130.92
130.78
130.44
129.06
84.85
83.04
77.34
77.02
76.71
46.22
210 190 170 150 130 110 90 80 70 60 50 40 30 20 10 0
f1 (ppm)

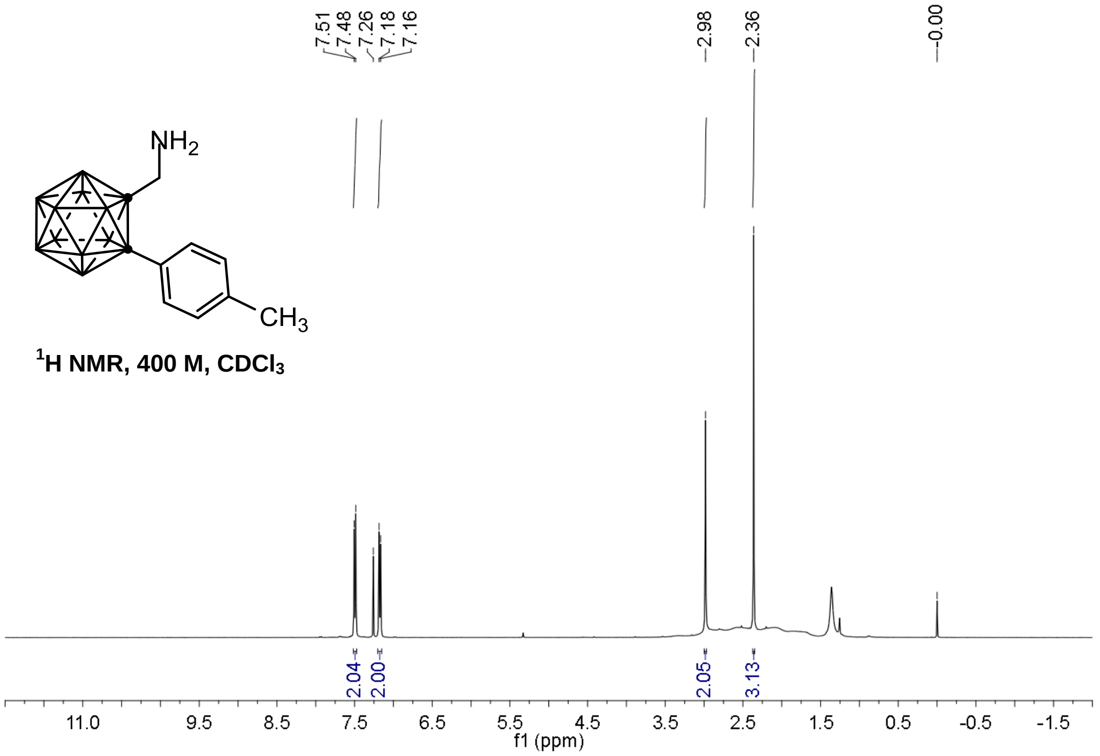
NH2
CH3
1H NMR, 400 M, CDCl3
7.51
7.48
7.26
7.18
7.16
2.98
2.36
0.00
2.04
2.00
2.05
3.13
11.0 9.5 8.5 7.5 6.5 5.5 4.5 3.5 2.5 1.5 0.5 -0.5 -1.5
f1 (ppm)

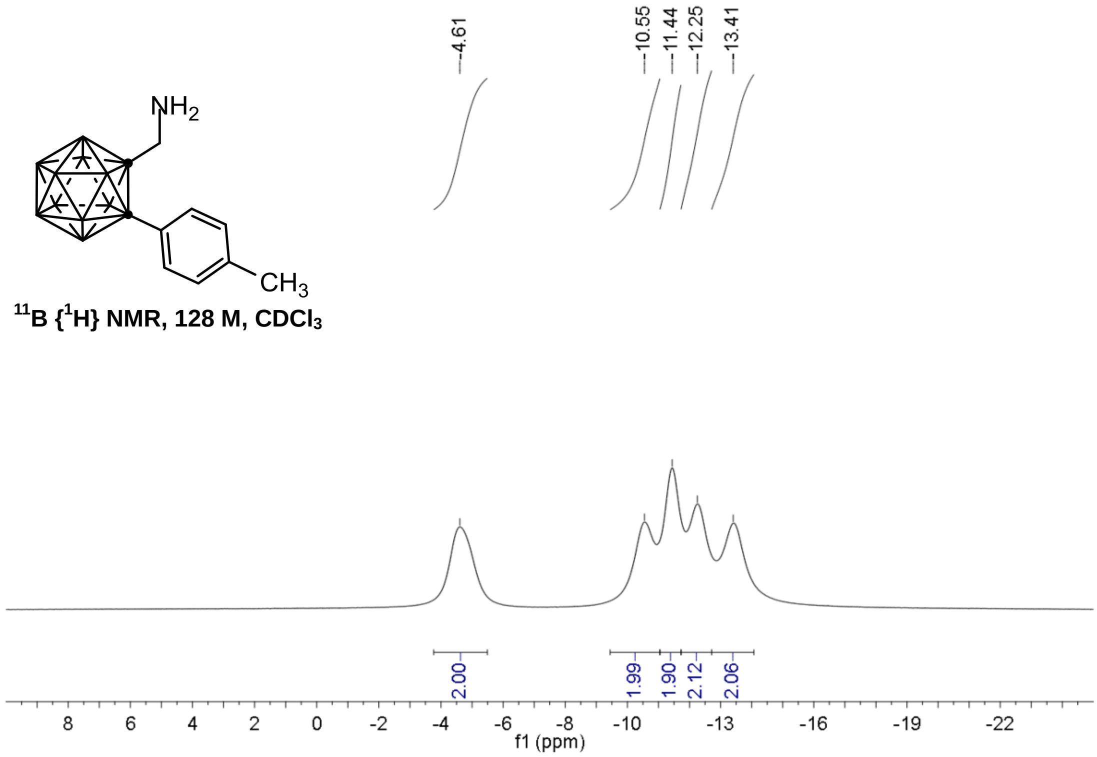

NH2
CH3
$^{11}B\ \{^1H\}$ NMR, 128 M, CDCl3
-4.61
-10.55
-11.44
-12.25
-13.41
2.00
1.99
1.90
2.12
2.06
8 6 4 2 0 -2 -4 -6 -8 -10 -13 -16 -19 -22
f1 (ppm)

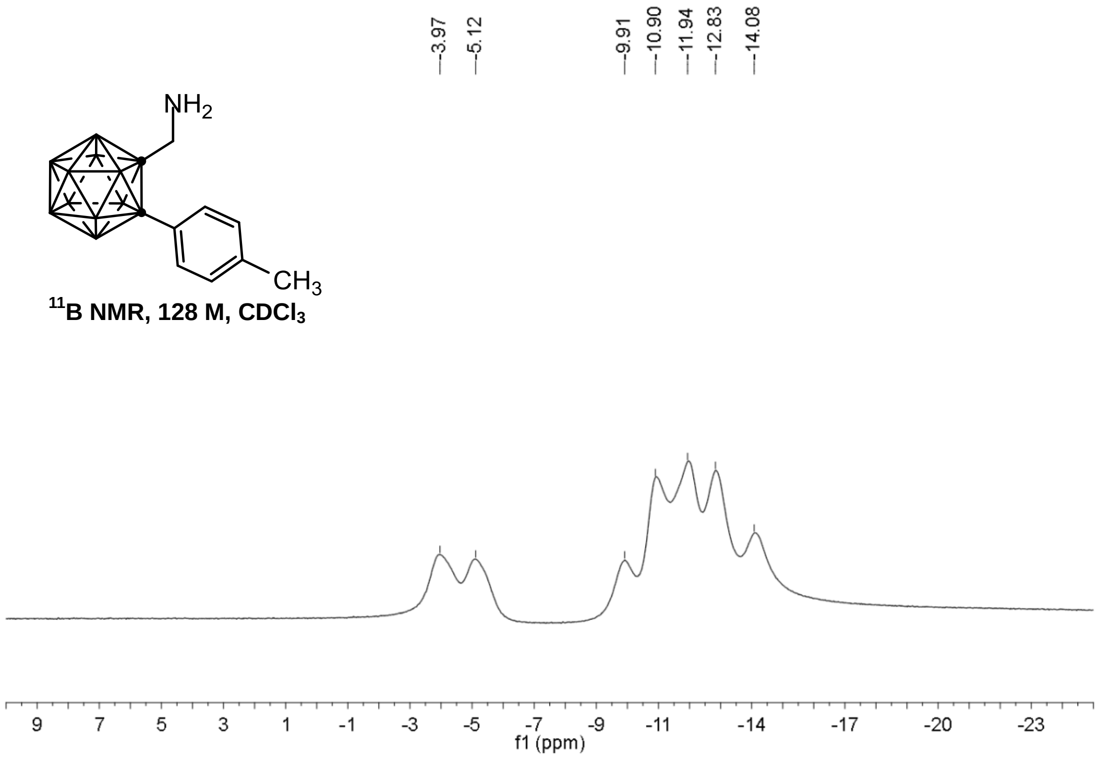

NH2
CH3
^{11}B NMR, 128 M, CDCl3
-3.97
-5.12
-9.91
-10.90
-11.94
-12.83
-14.08
9 7 5 3 1 -1 -3 -5 -7 -9 -11 -14 -17 -20 -23
f1 (ppm)

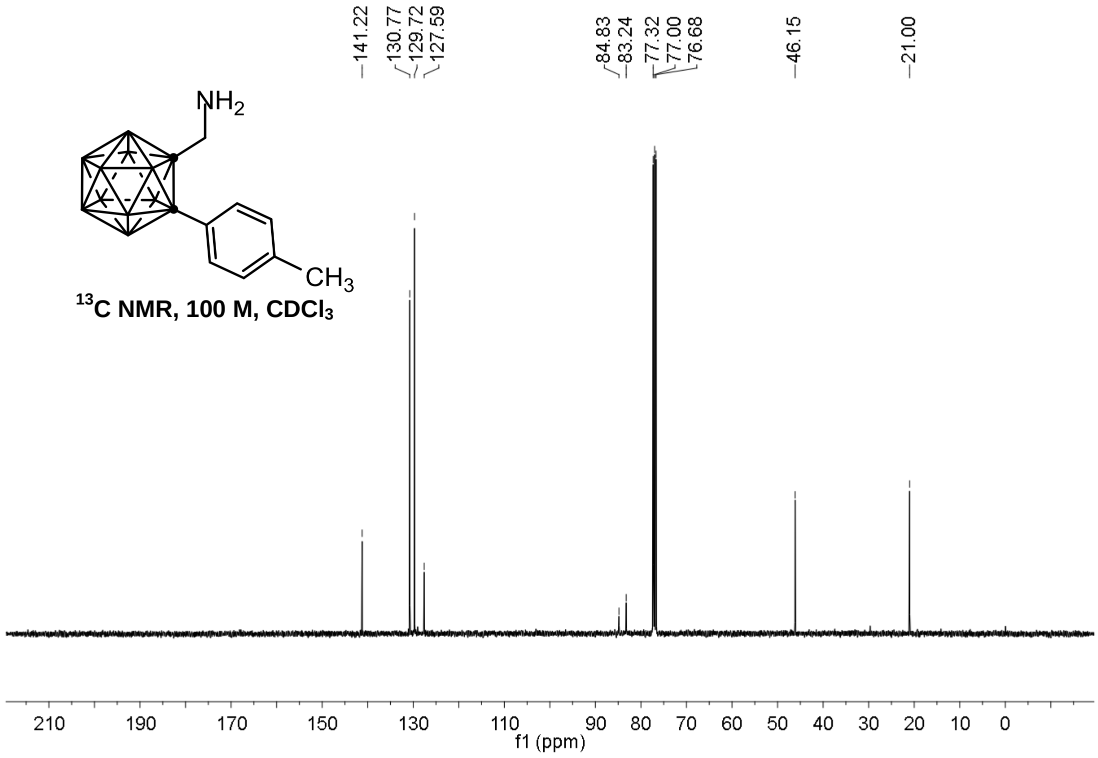

141.22
130.77
129.72
127.59
84.83
83.24
77.32
77.00
76.68
46.15
21.00
NH2
CH3
13C NMR, 100 M, CDCl3
210 190 170 150 130 110 90 80 70 60 50 40 30 20 10 0
f1 (ppm)

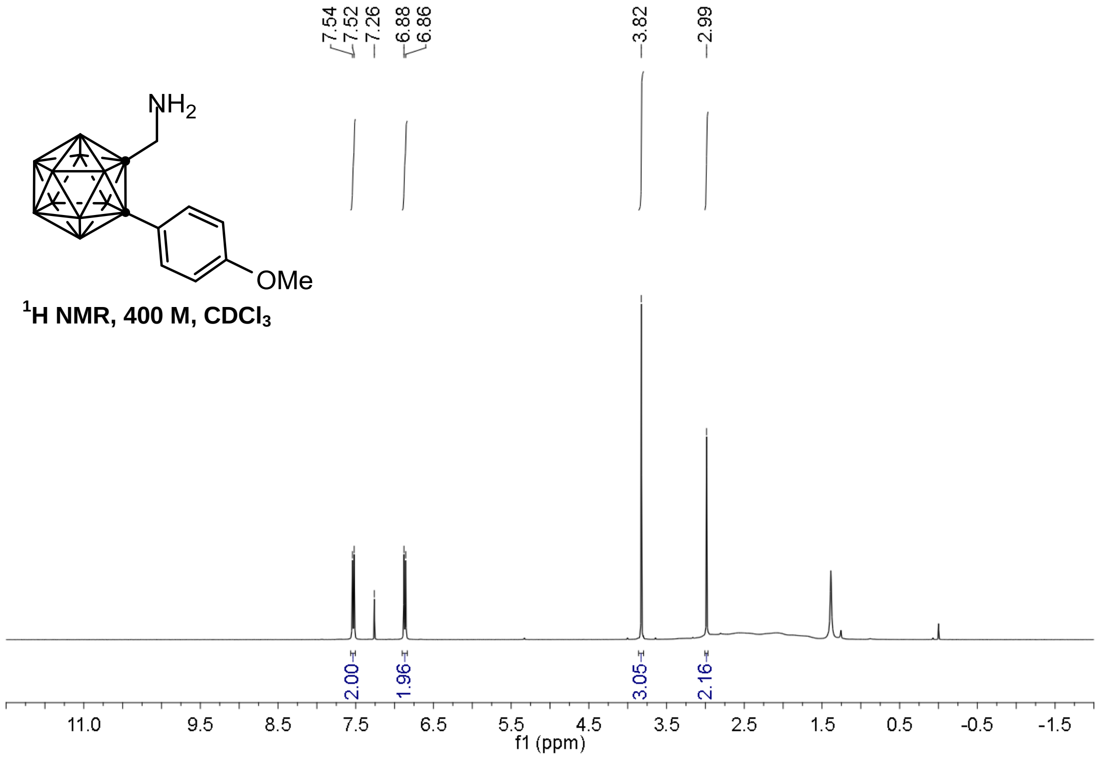

7.54
7.52
7.26
6.88
6.86
3.82
2.99
NH2
OMe
1H NMR, 400 M, CDCl3
2.00
1.96
3.05
2.16
11.0 9.5 8.5 7.5 6.5 5.5 4.5 3.5 2.5 1.5 0.5 -0.5 -1.5
f1 (ppm)

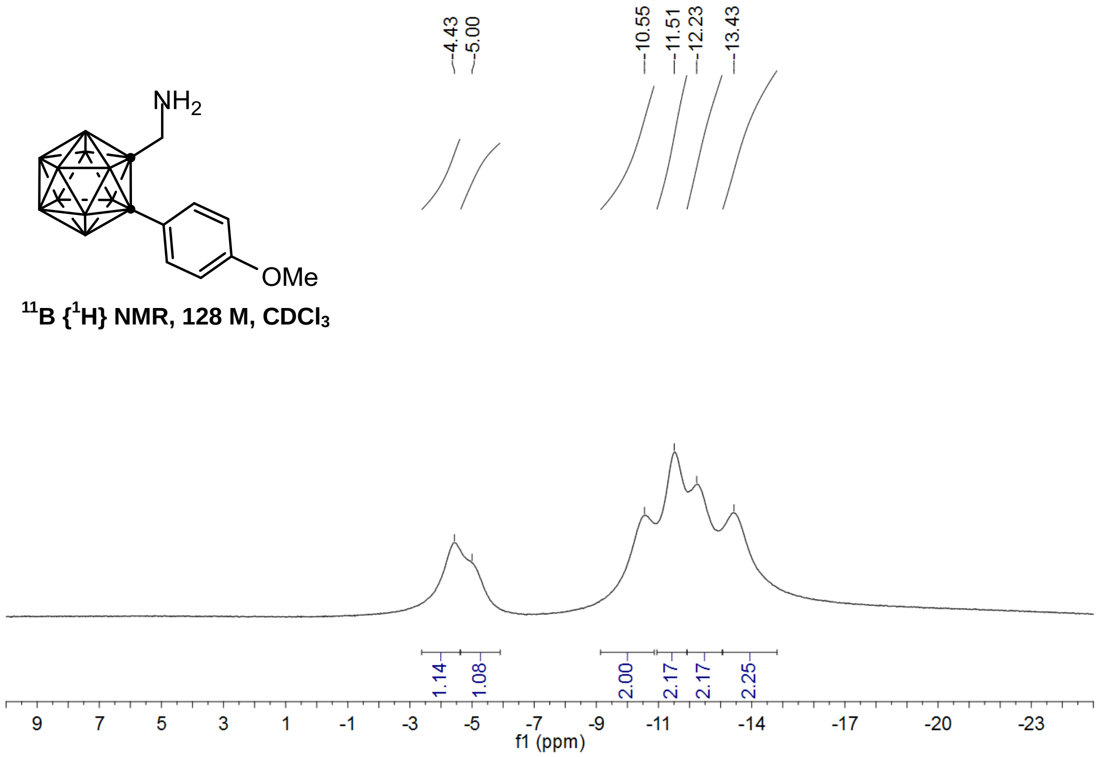

NH2
OMe
11B {1H} NMR, 128 M, CDCl3
-4.43
-5.00
-10.55
-11.51
-12.23
-13.43
1.14
1.08
2.00
2.17
2.17
2.25
9 7 5 3 1 -1 -3 -5 -7 -9 -11 -14 -17 -20 -23
f1 (ppm)

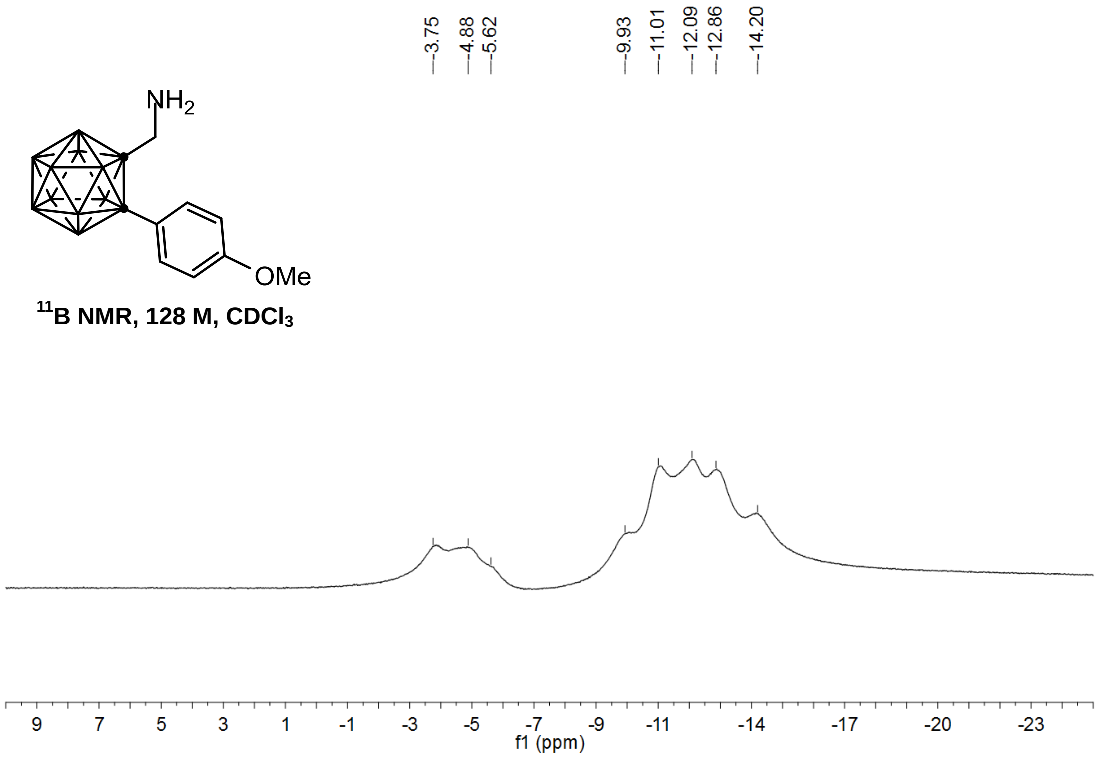

NH2
OMe
11B NMR, 128 M, CDCl3
-3.75
-4.88
-5.62
-9.93
-11.01
-12.09
-12.86
-14.20
9 7 5 3 1 -1 -3 -5 -7 -9 -11 -14 -17 -20 -23
f1 (ppm)

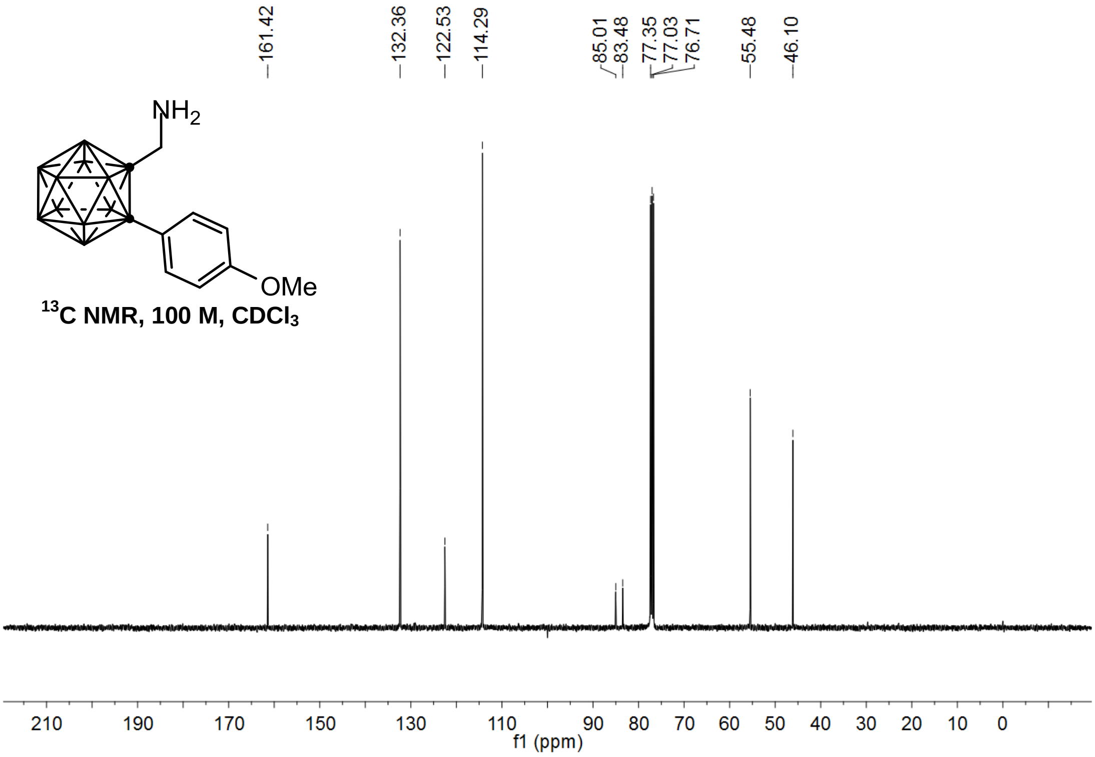

161.42
132.36
122.53
114.29
85.01
83.48
77.35
77.03
76.71
55.48
46.10
NH2
OMe
13C NMR, 100 M, CDCl3
210
190
170
150
130
110
90
80
70
60
50
40
30
20
10
0
f1 (ppm)

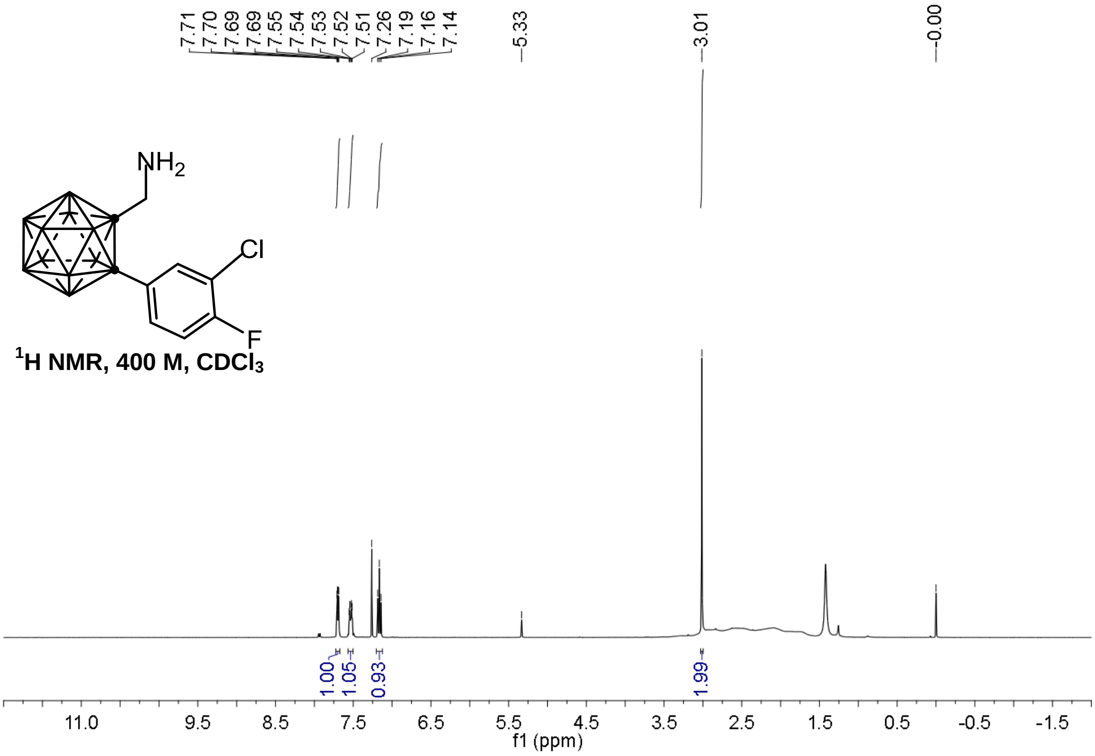

7.71
7.70
7.69
7.69
7.55
7.54
7.53
7.52
7.51
7.26
7.19
7.16
7.14
5.33
3.01
0.00
NH2
Cl
F
1H NMR, 400 M, CDCl3
1.00
1.05
0.93
1.99
11.0
9.5
8.5
7.5
6.5
5.5
4.5
3.5
2.5
1.5
0.5
-0.5
-1.5
f1 (ppm)

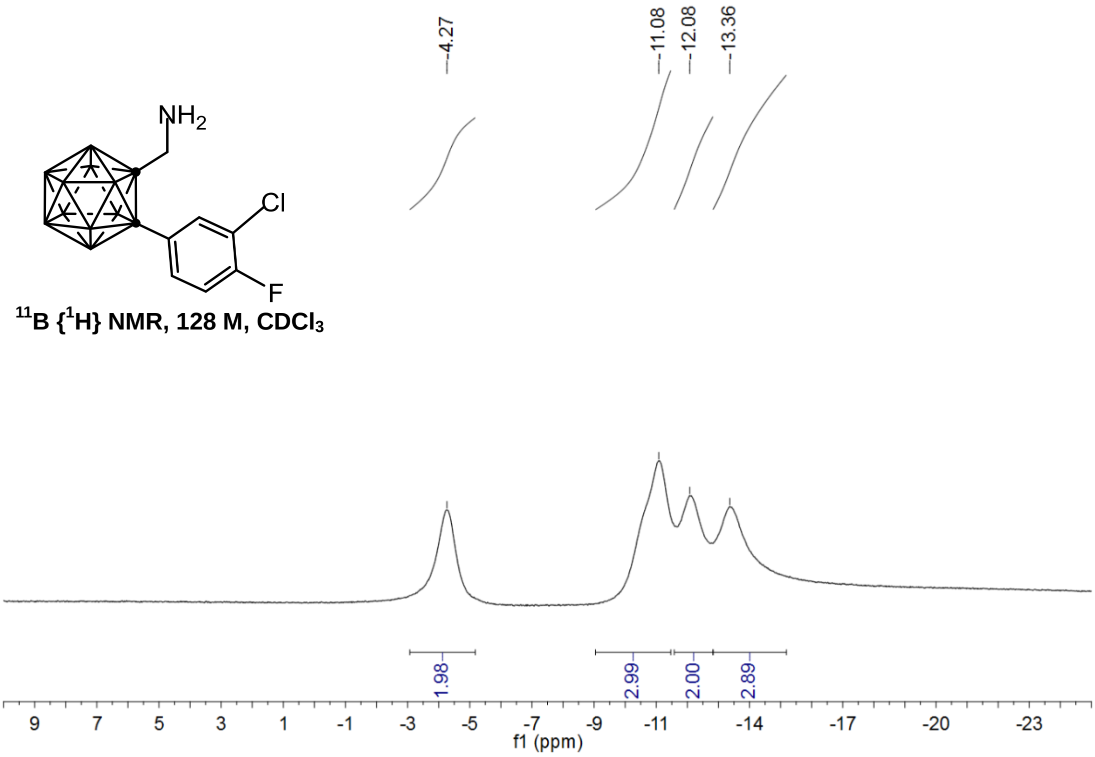

^{11}B {^{1}H} NMR, 128 M, CDCl$_3$

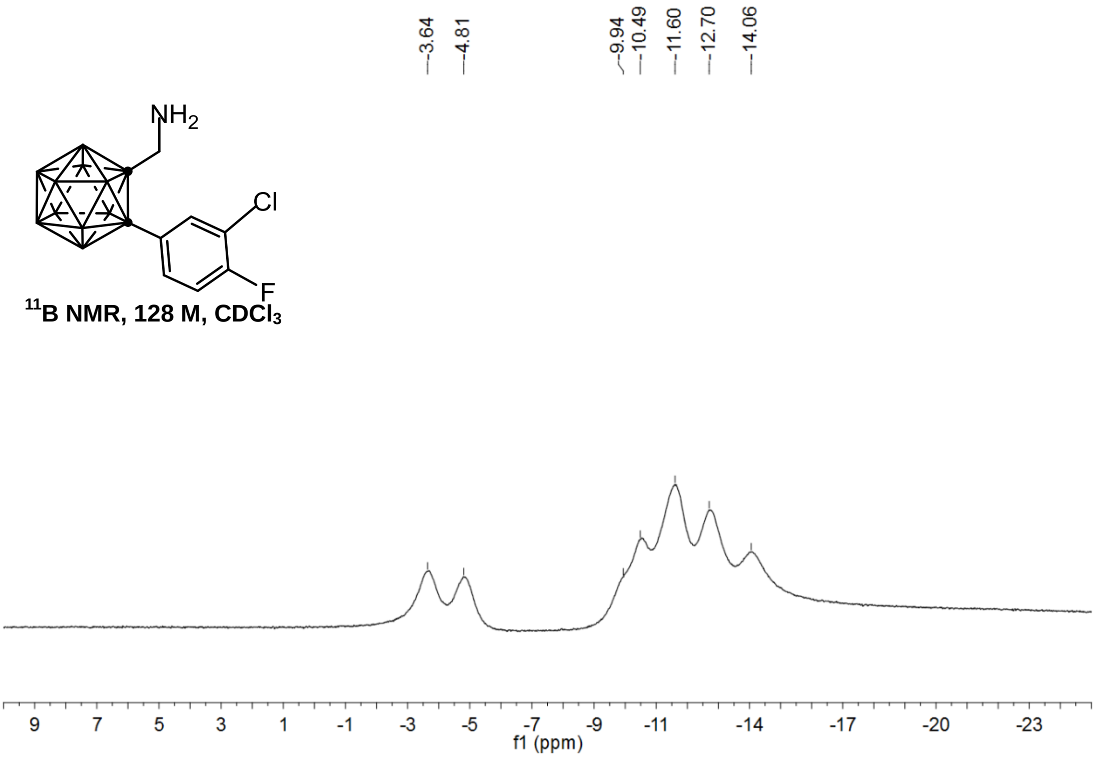

^{11}B NMR, 128 M, CDCl$_3$

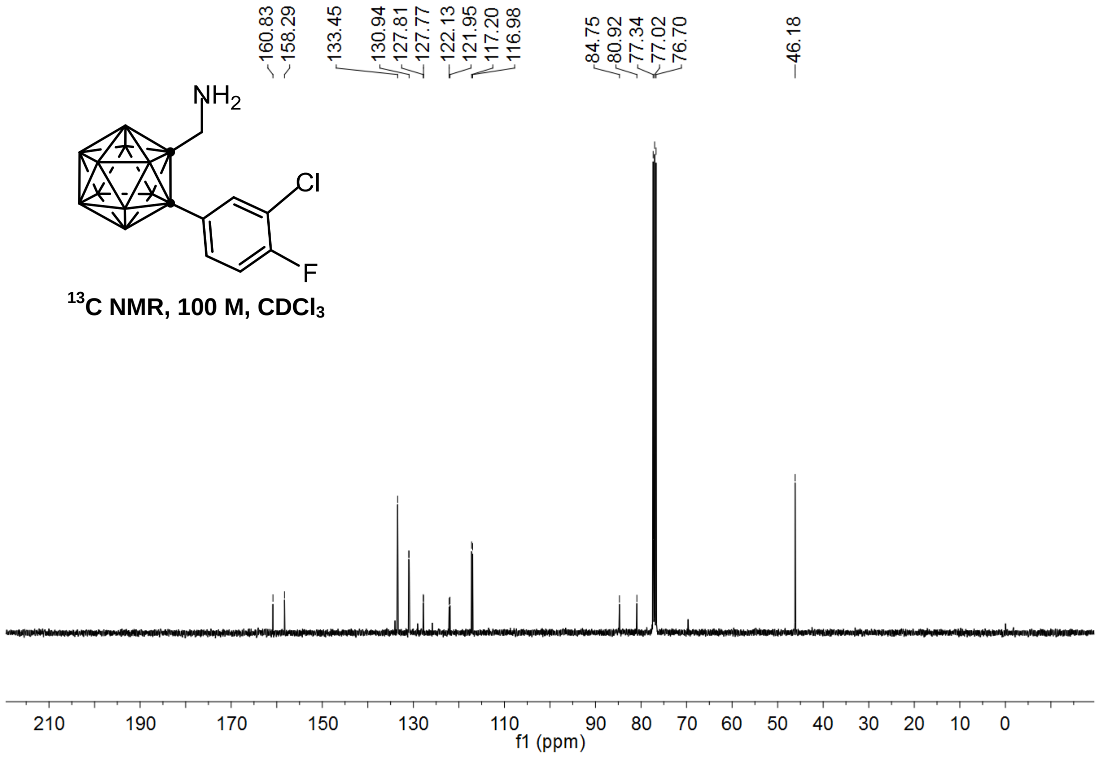
160.83
158.29
133.45
130.94
127.81
127.77
122.13
121.95
117.20
116.98
84.75
80.92
77.34
77.02
76.70
46.18
NH2
Cl
F
13C NMR, 100 M, CDCl3
210
190
170
150
130
110
90
80
70
60
50
40
30
20
10
0
f1 (ppm)

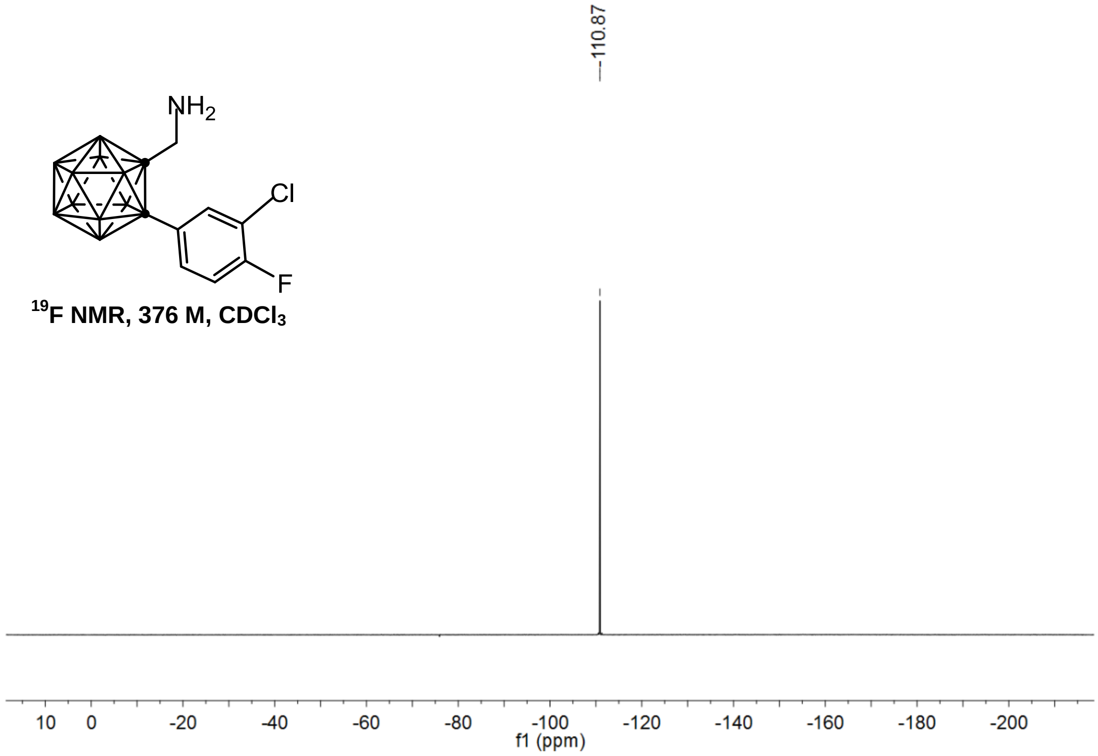
-110.87
NH2
Cl
F
19F NMR, 376 M, CDCl3
10
0
-20
-40
-60
-80
-100
-120
-140
-160
-180
-200
f1 (ppm)

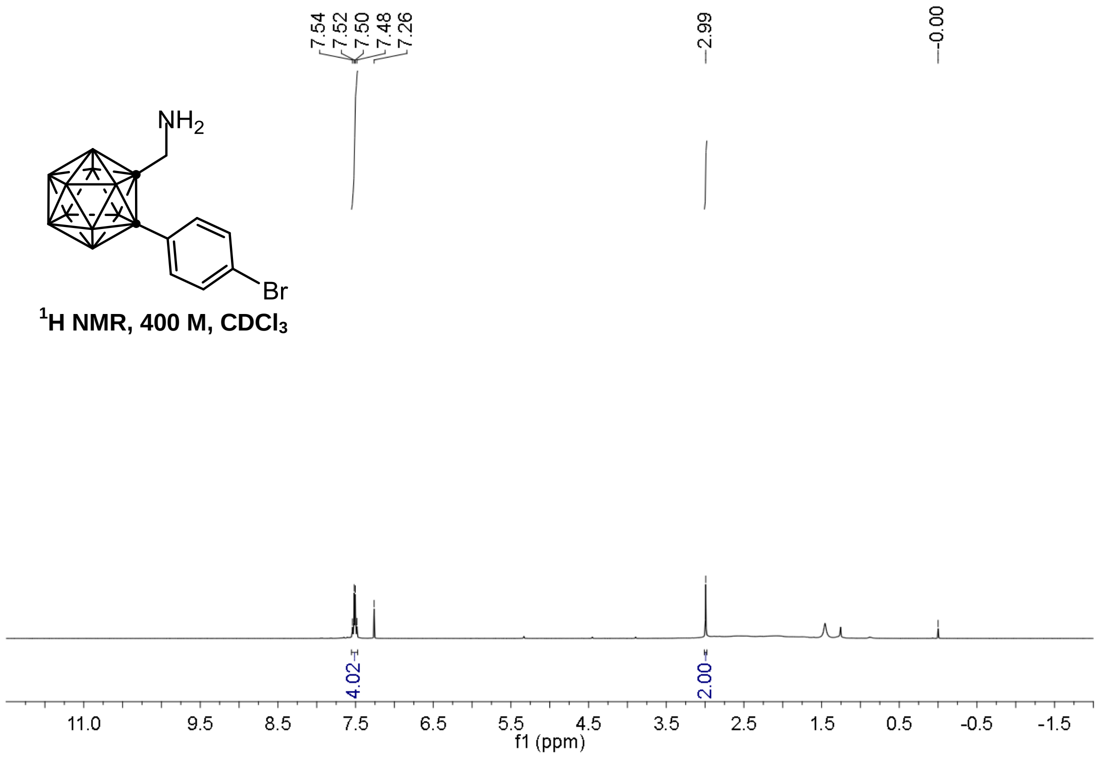
NH2
Br
1H NMR, 400 M, CDCl3
7.54
7.52
7.50
7.48
7.26
2.99
0.00
4.02
2.00
f1 (ppm)

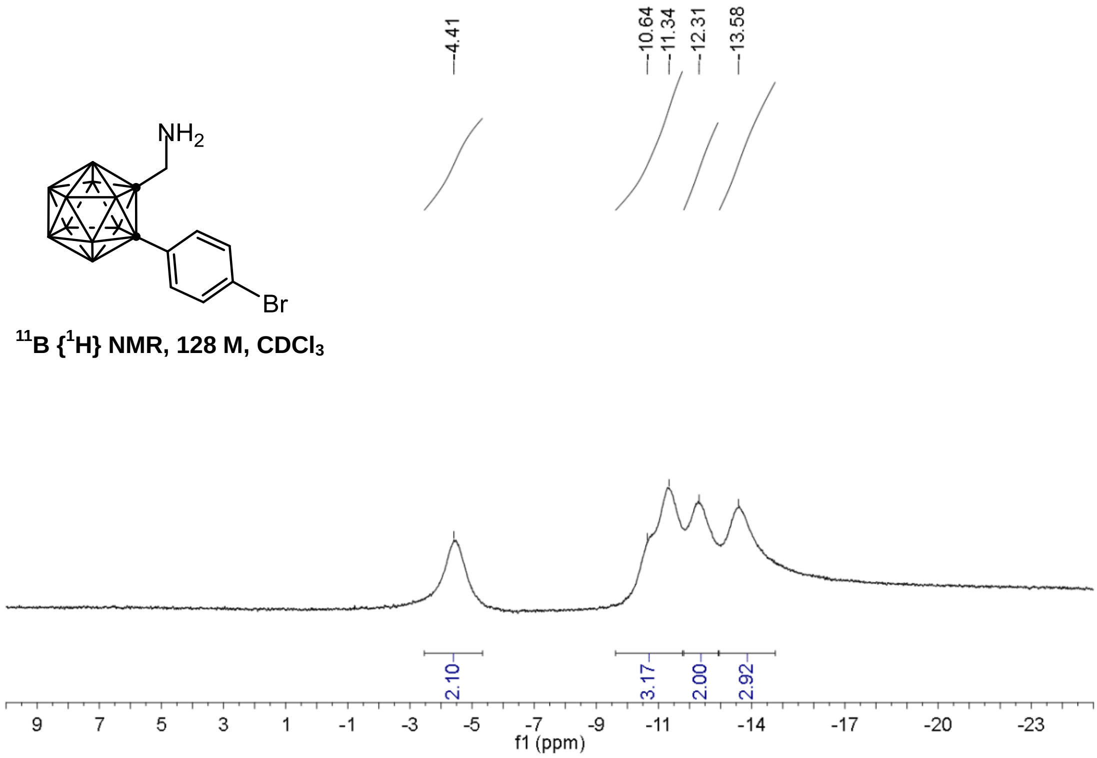
NH2
Br
11B {1H} NMR, 128 M, CDCl3
-4.41
-10.64
-11.34
-12.31
-13.58
2.10
3.17
2.00
2.92
f1 (ppm)

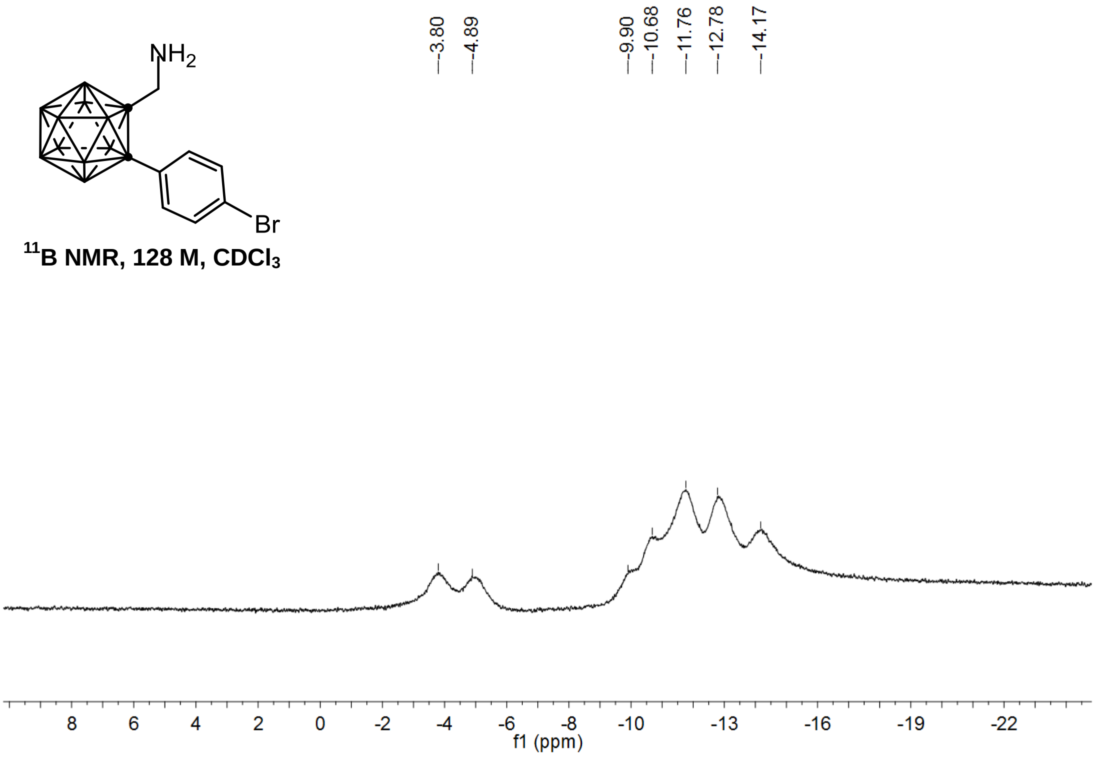
NH2
Br
11B NMR, 128 M, CDCl3
-3.80
-4.89
-9.90
-10.68
-11.76
-12.78
-14.17
f1 (ppm)

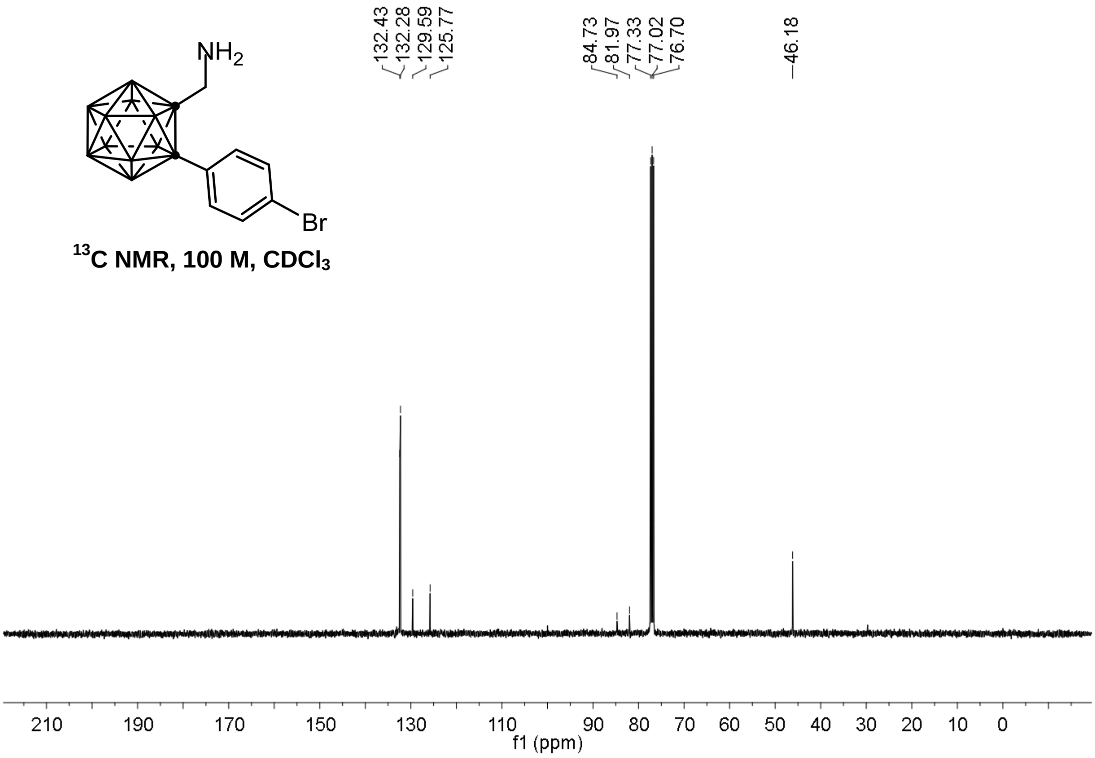
NH2
Br
13C NMR, 100 M, CDCl3
132.43
132.28
129.59
125.77
84.73
81.97
77.33
77.02
76.70
46.18
f1 (ppm)

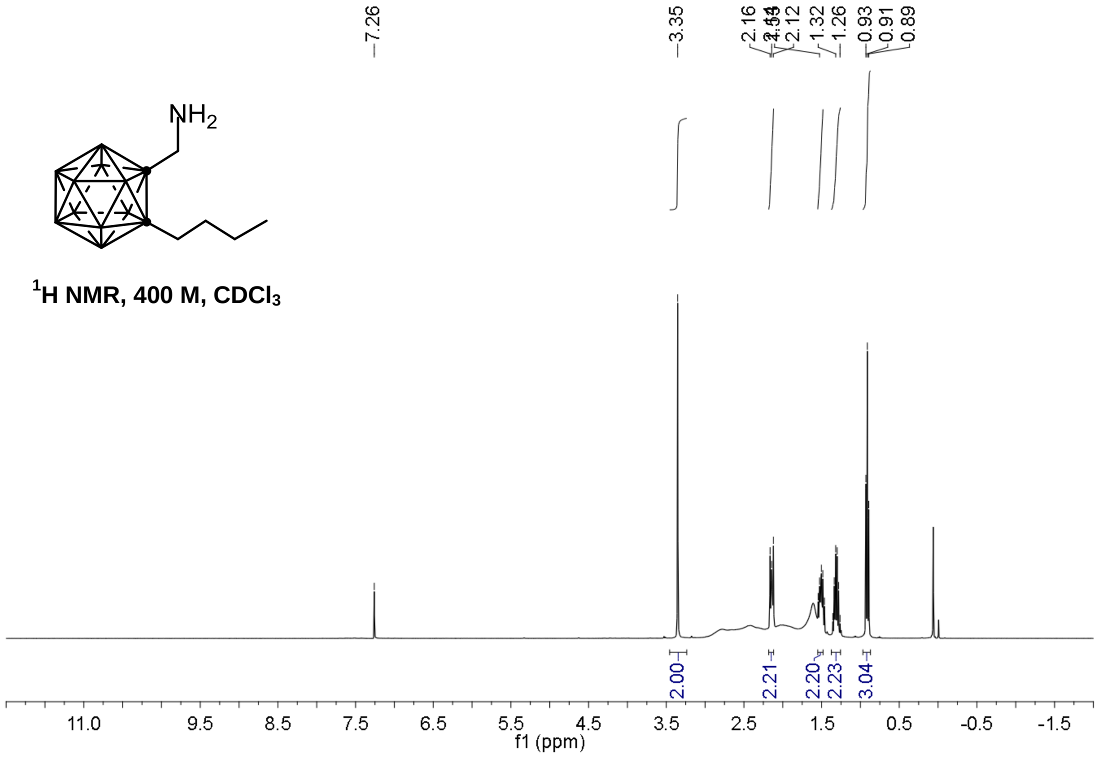

^{1}H NMR, 400 M, $CDCl_3$

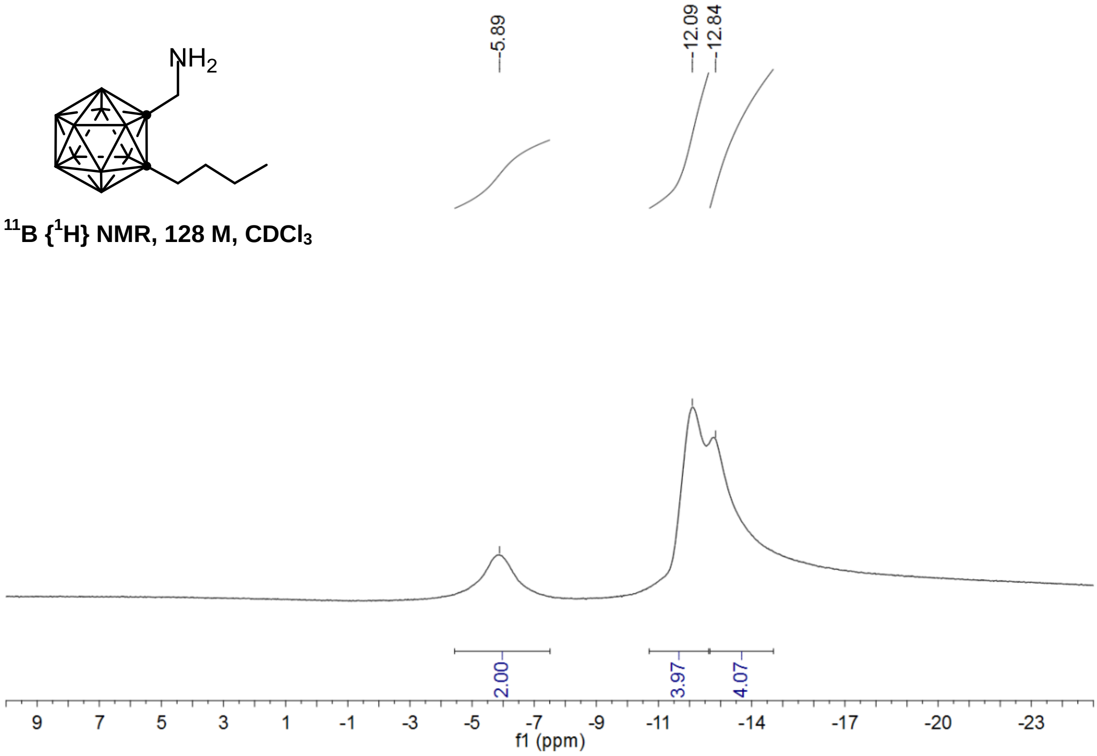

^{11}B {^{1}H} NMR, 128 M, $CDCl_3$

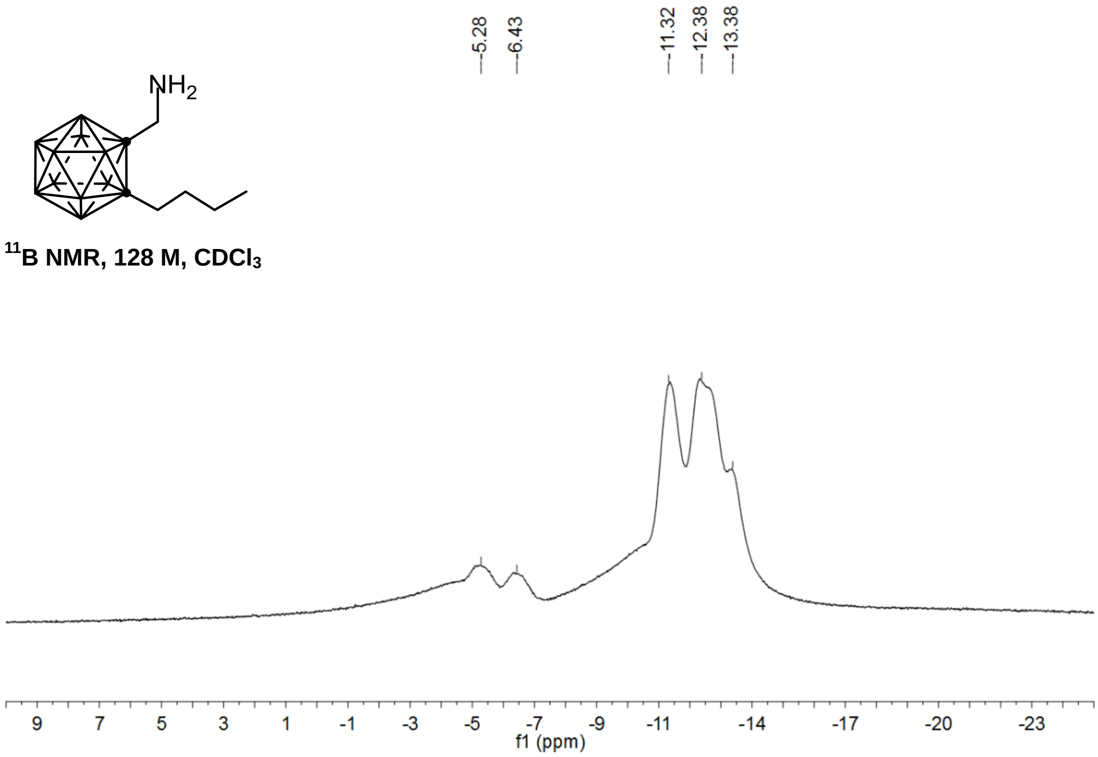

^{11}B NMR, 128 M, $CDCl_3$

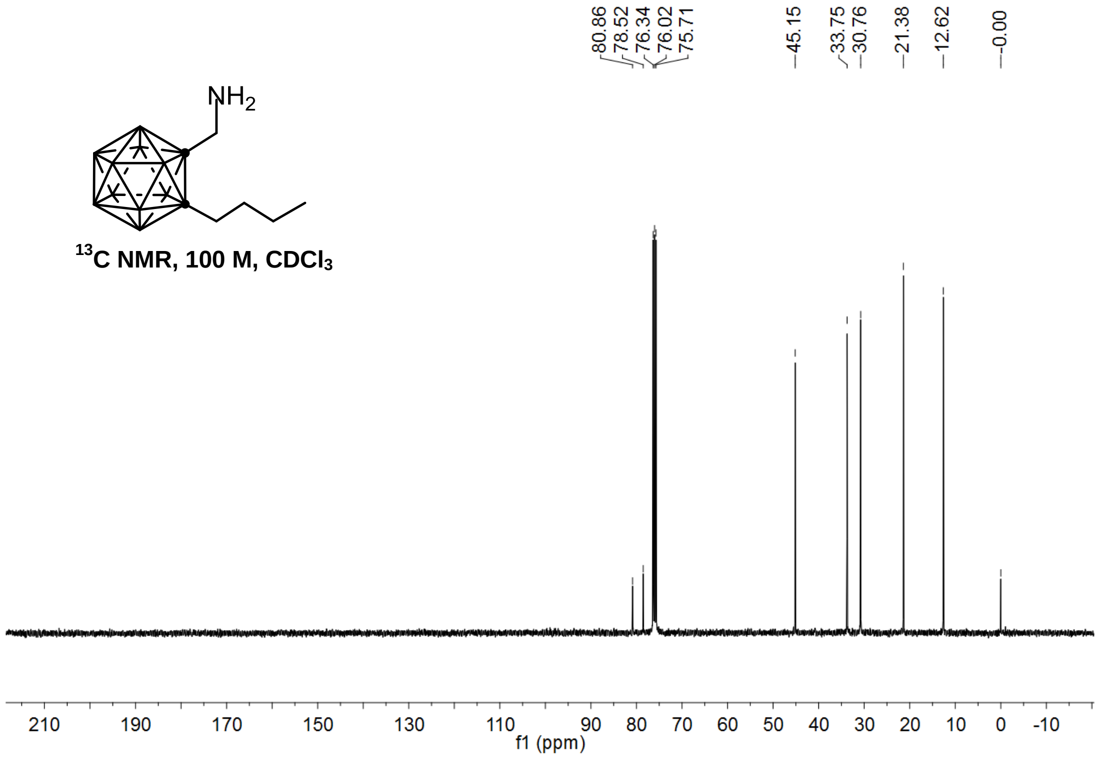

^{13}C NMR, 100 M, $CDCl_3$

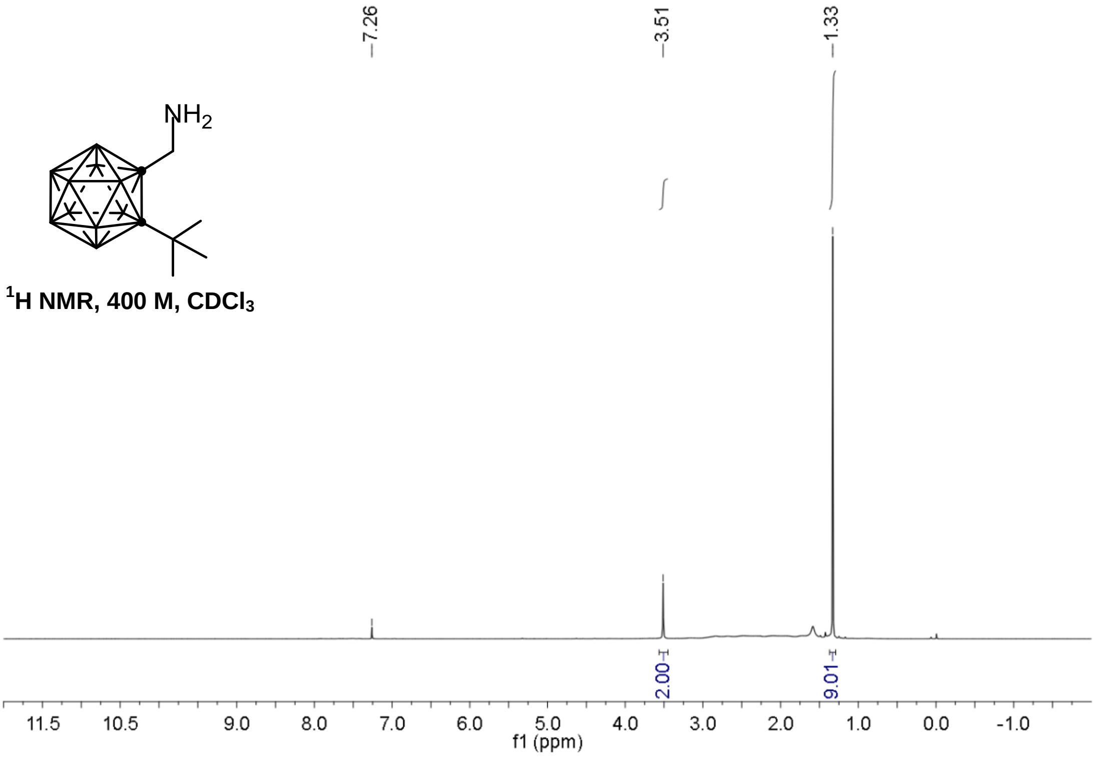

^{1}H NMR, 400 M, $CDCl_3$

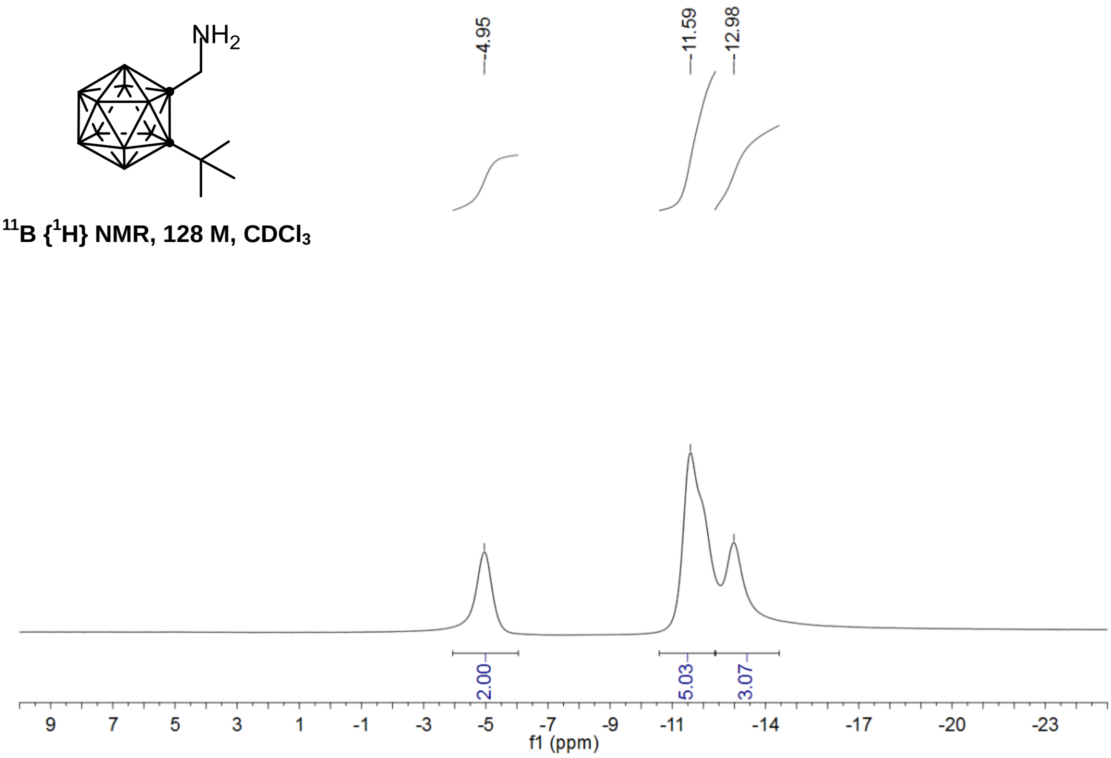

^{11}B {^{1}H} NMR, 128 M, $CDCl_3$

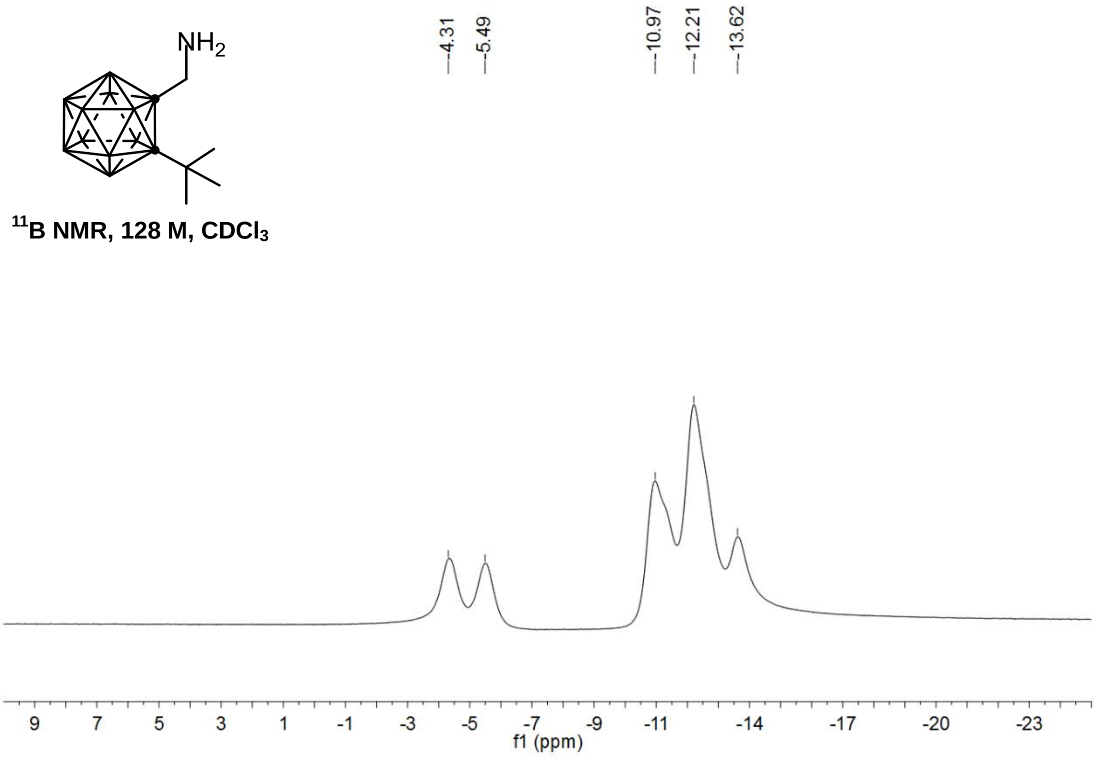
NH2
11B NMR, 128 M, CDCl3
-4.31
-5.49
-10.97
-12.21
-13.62
9
7
5
3
1
-1
-3
-5
-7
-9
-11
-14
-17
-20
-23
f1 (ppm)

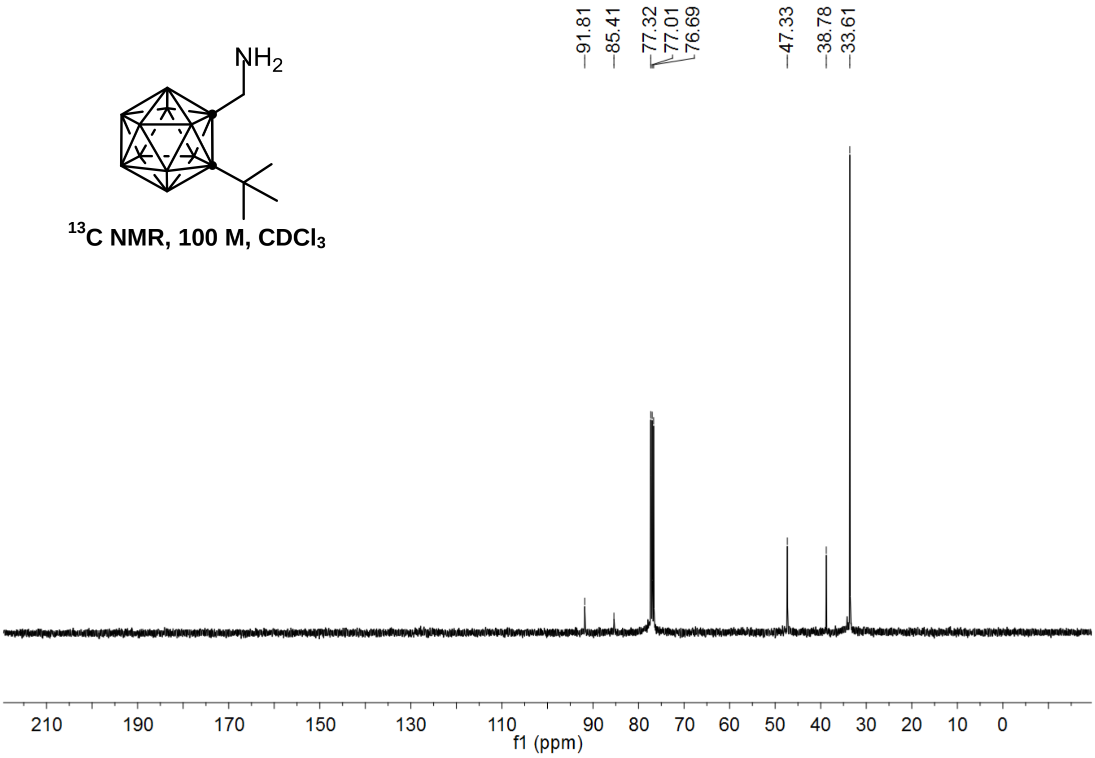
NH2
13C NMR, 100 M, CDCl3
91.81
85.41
77.32
77.01
76.69
47.33
38.78
33.61
210
190
170
150
130
110
90
80
70
60
50
40
30
20
10
0
f1 (ppm)

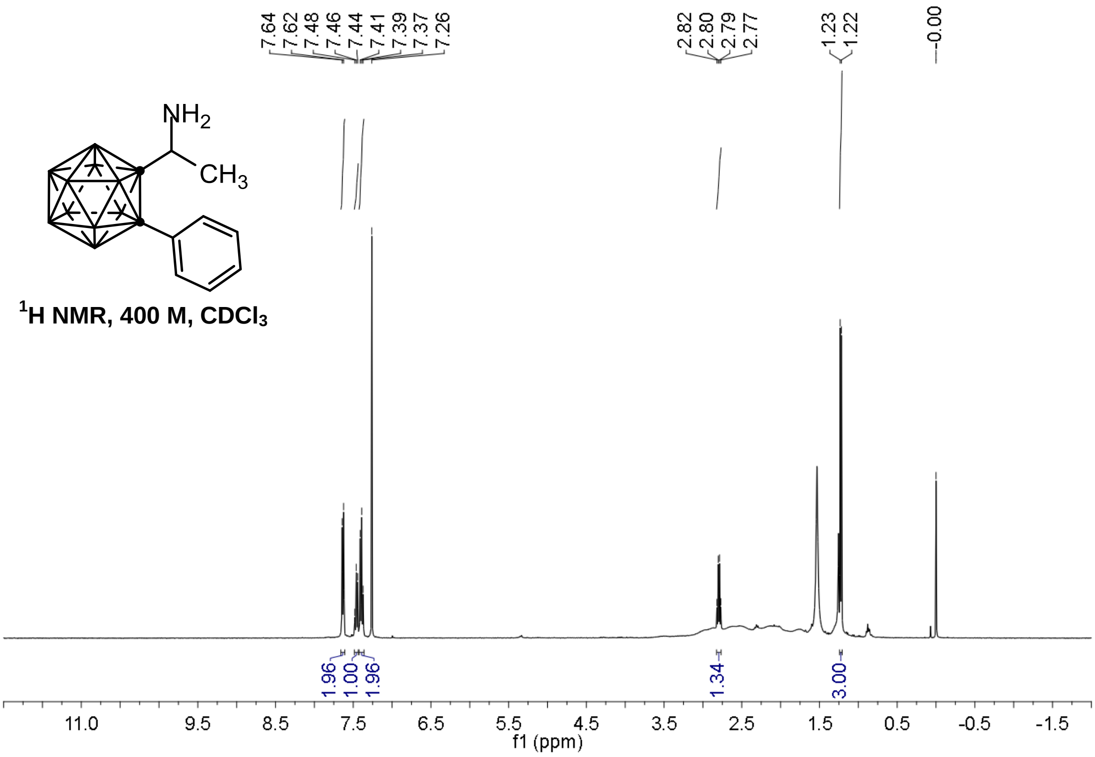

7.64
7.62
7.48
7.46
7.44
7.41
7.39
7.37
7.26
2.82
2.80
2.79
2.77
1.23
1.22
0.00
NH2
CH3
1H NMR, 400 M, CDCl3
1.96
1.00
1.96
1.34
3.00
11.0
9.5
8.5
7.5
6.5
5.5
4.5
3.5
2.5
1.5
0.5
-0.5
-1.5
f1 (ppm)

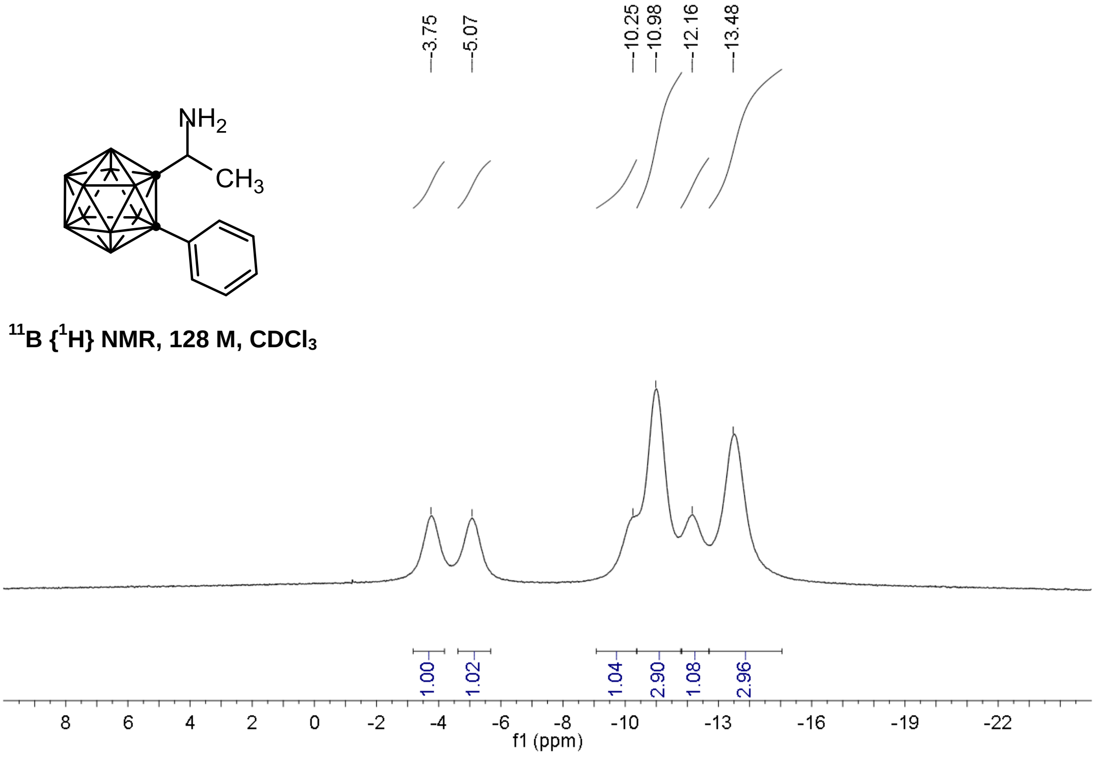

-3.75
-5.07
-10.25
-10.98
-12.16
-13.48
NH2
CH3
11B {1H} NMR, 128 M, CDCl3
1.00
1.02
1.04
2.90
1.08
2.96
8
6
4
2
0
-2
-4
-6
-8
-10
-13
-16
-19
-22
f1 (ppm)

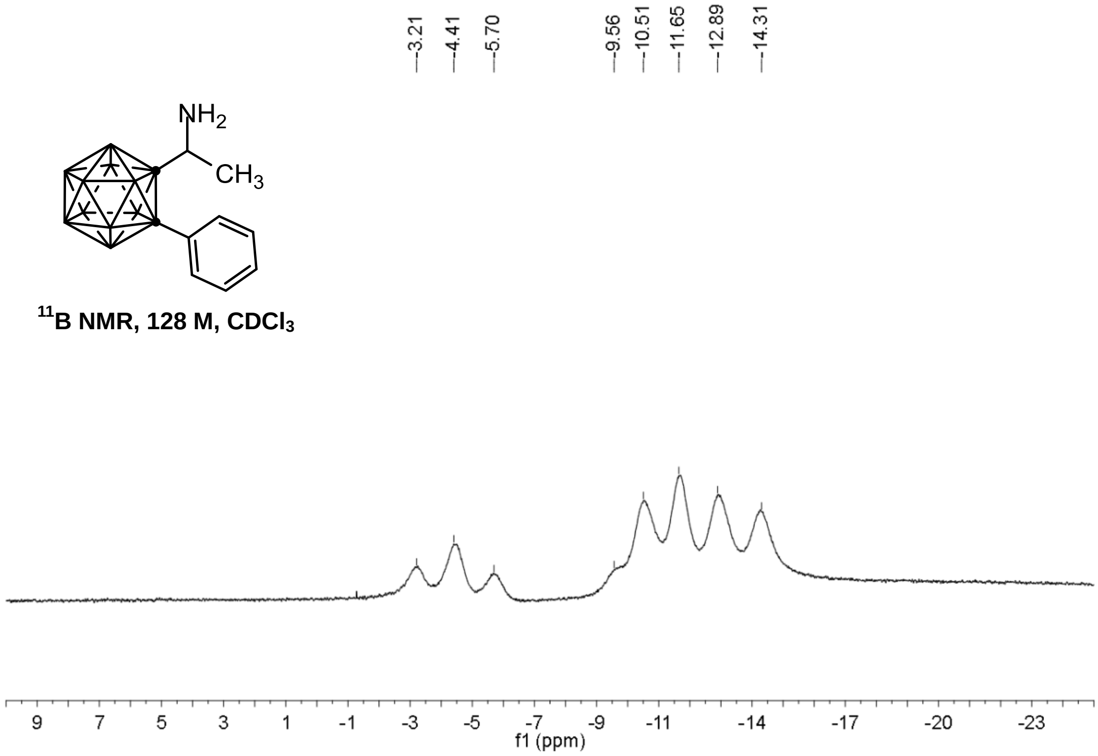
3.21
4.41
5.70
9.56
10.51
11.65
12.89
14.31
NH2
CH3
11B NMR, 128 M, CDCl3
9 7 5 3 1 -1 -3 -5 -7 -9 -11 -14 -17 -20 -23
f1 (ppm)

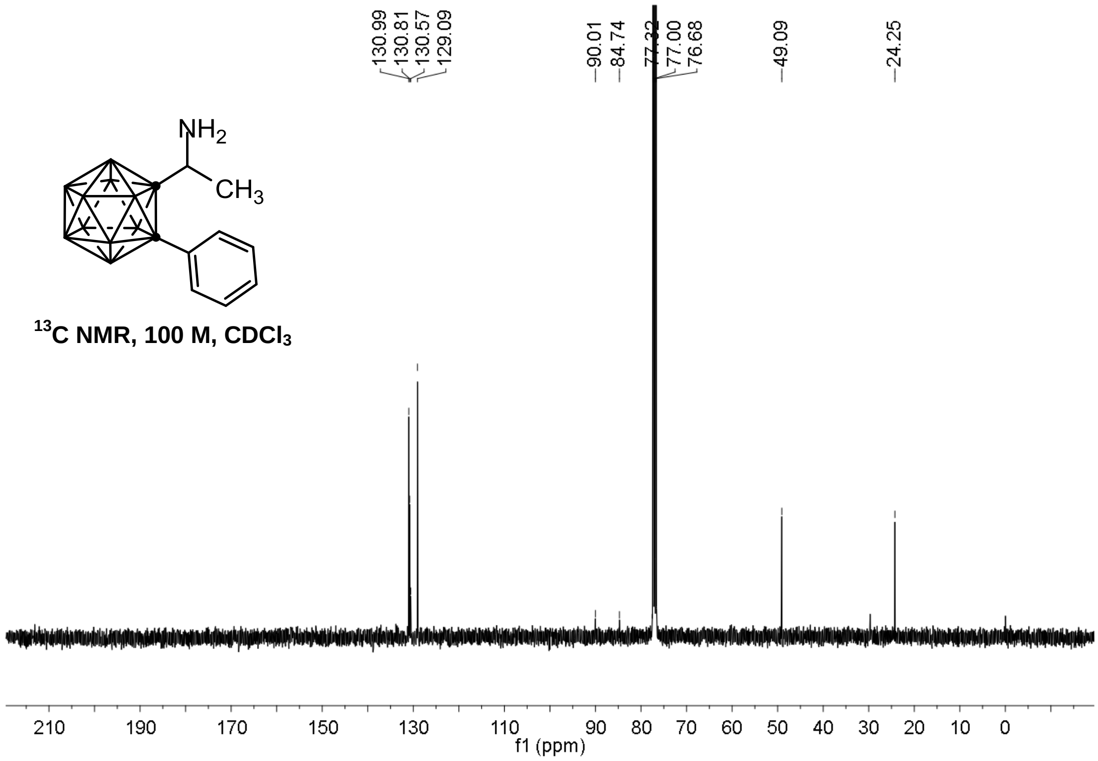
130.99
130.81
130.57
129.09
90.01
84.74
77.00
76.68
49.09
24.25
NH2
CH3
13C NMR, 100 M, CDCl3
210 190 170 150 130 110 90 80 70 60 50 40 30 20 10 0
f1 (ppm)

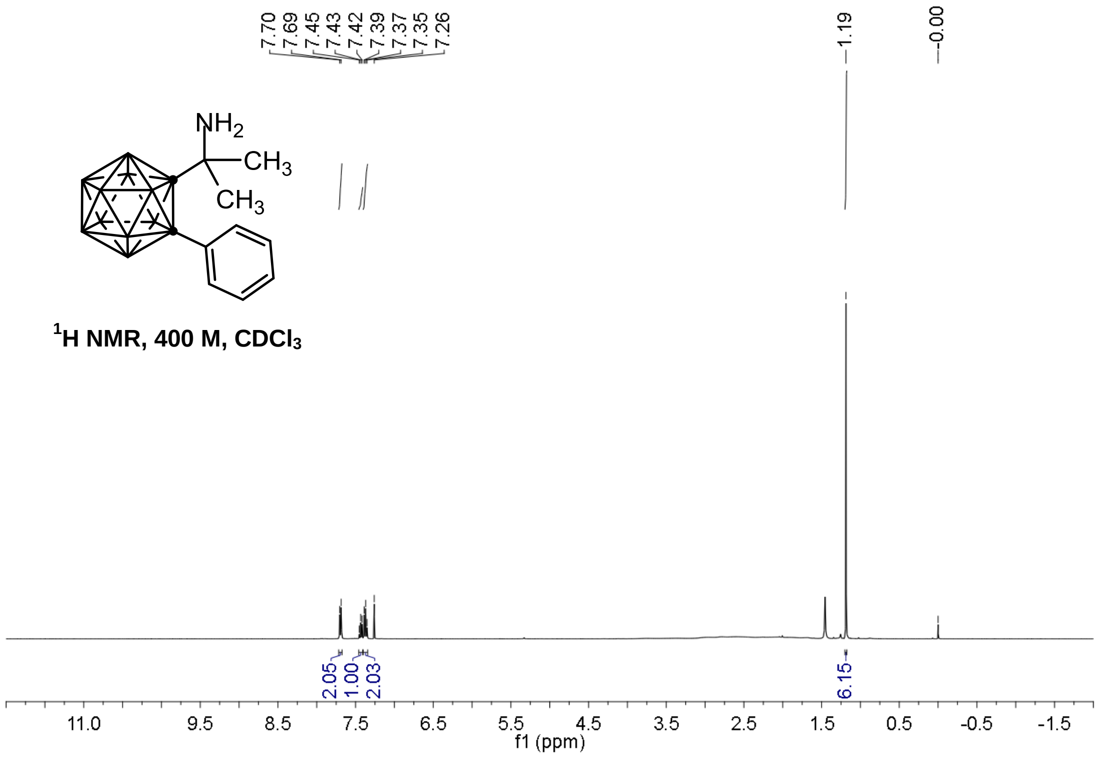
7.70
7.69
7.45
7.43
7.42
7.39
7.37
7.35
7.26
1.19
0.00
NH2
CH3
CH3
1H NMR, 400 M, CDCl3
2.05
1.00
2.03
6.15
11.0
9.5
8.5
7.5
6.5
5.5
4.5
3.5
2.5
1.5
0.5
-0.5
-1.5
f1 (ppm)

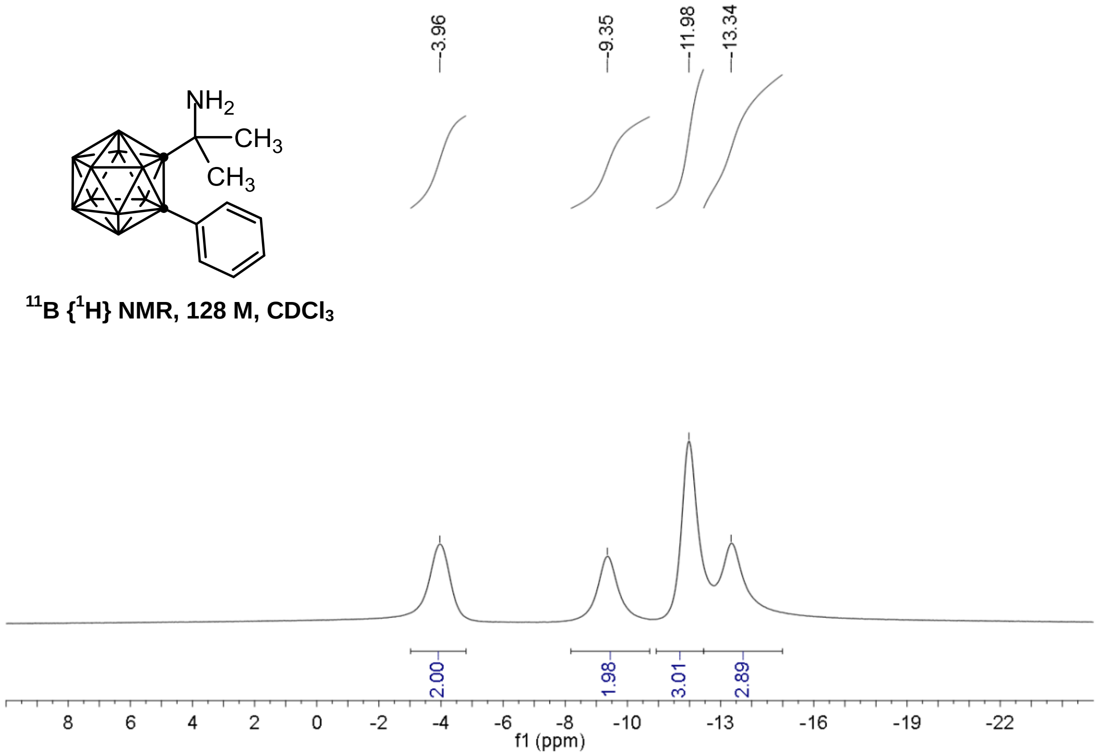
-3.96
-9.35
-11.98
-13.34
NH2
CH3
CH3
11B {1H} NMR, 128 M, CDCl3
2.00
1.98
3.01
2.89
8
6
4
2
0
-2
-4
-6
-8
-10
-13
-16
-19
-22
f1 (ppm)

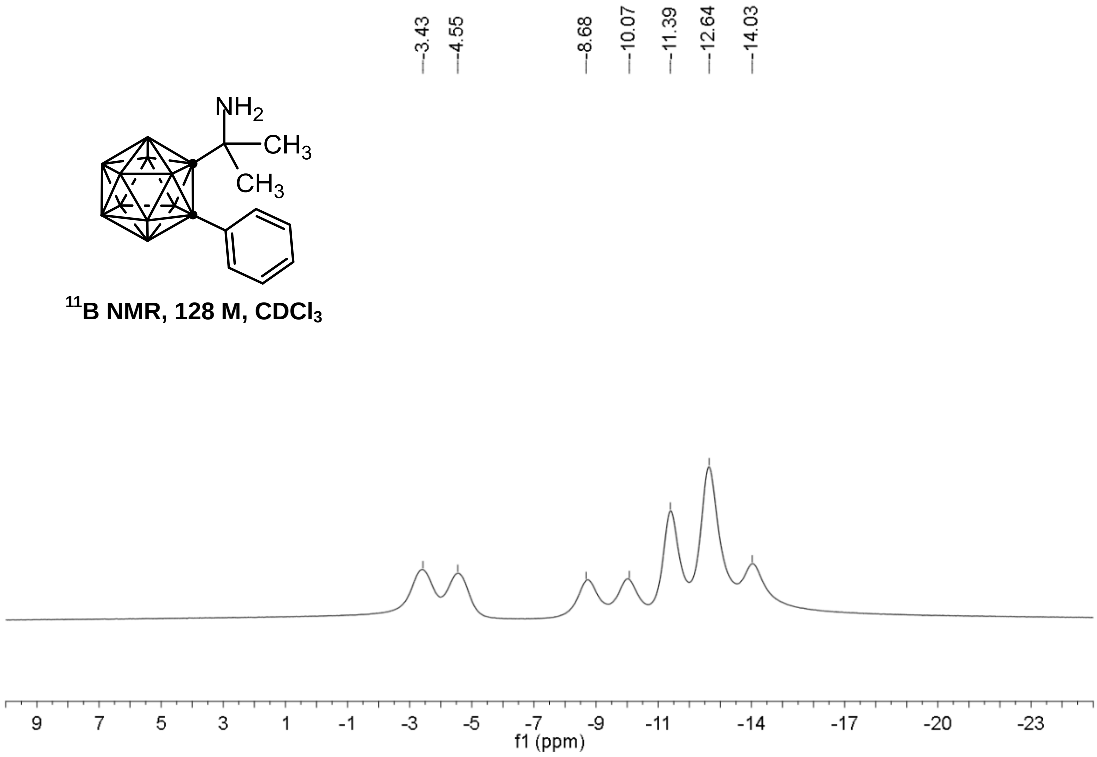

^{11}B NMR, 128 M, CDCl$_3$

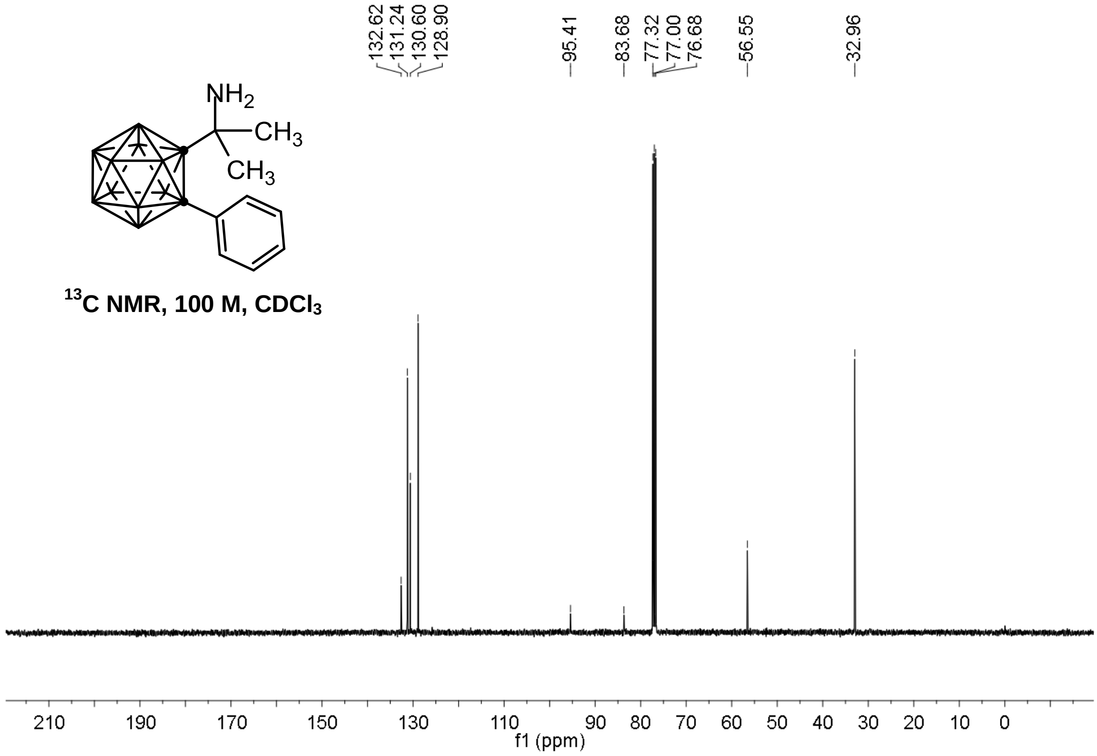

^{13}C NMR, 100 M, CDCl$_3$

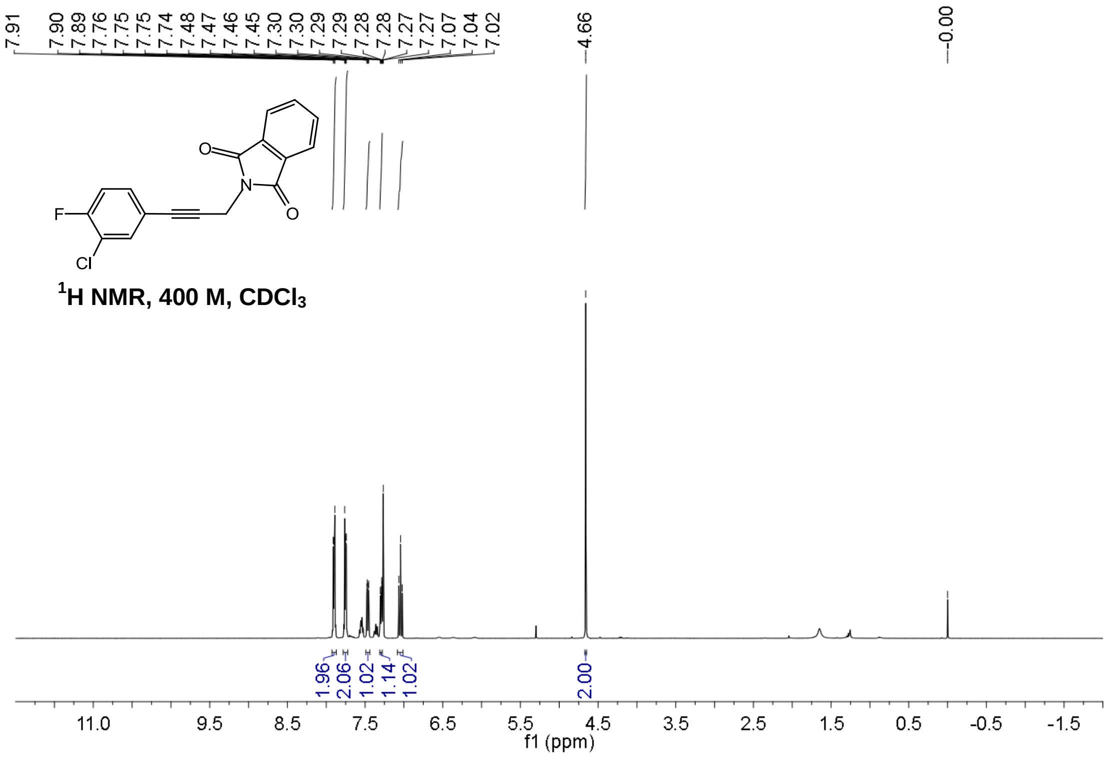
7.91
7.90
7.89
7.76
7.75
7.75
7.74
7.48
7.47
7.46
7.45
7.30
7.30
7.29
7.29
7.28
7.28
7.27
7.27
7.07
7.04
7.02
4.66
0.00
F
Cl
N
O
O
¹H NMR, 400 M, CDCl₃
1.96
2.06
1.02
1.14
1.02
2.00
11.0
9.5
8.5
7.5
6.5
5.5
4.5
3.5
2.5
1.5
0.5
-0.5
-1.5
f1 (ppm)

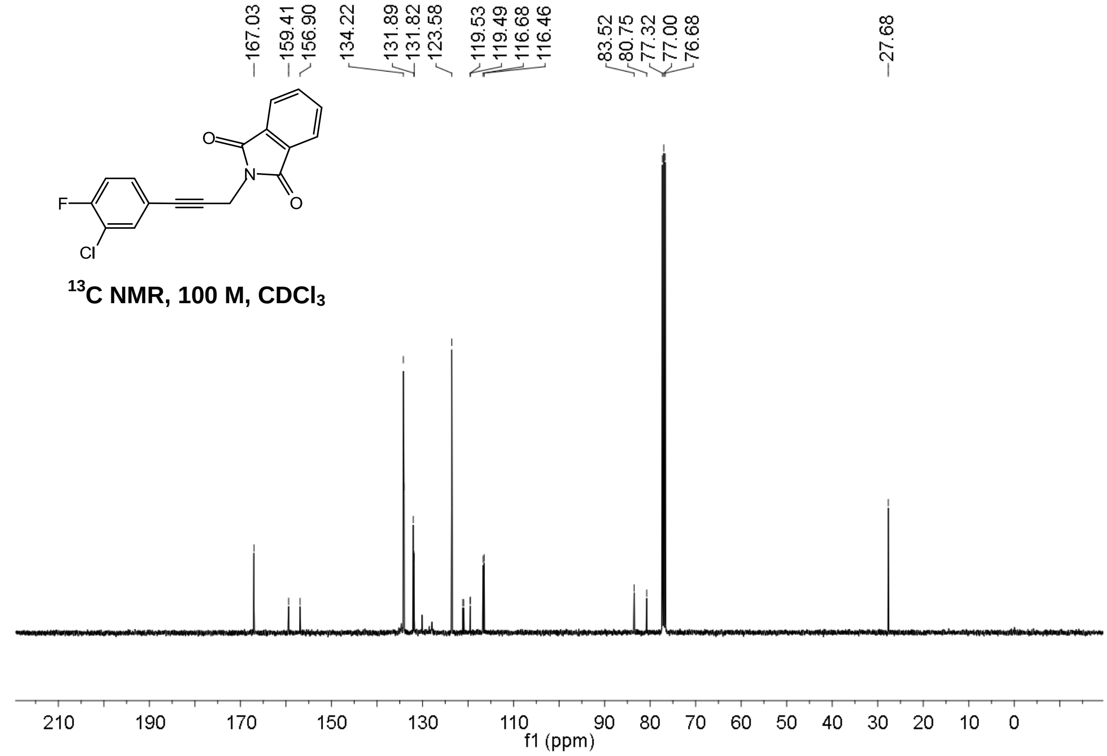
167.03
159.41
156.90
134.22
131.89
131.82
123.58
119.53
119.49
116.68
116.46
83.52
80.75
77.32
77.00
76.68
27.68
F
Cl
N
O
O
¹³C NMR, 100 M, CDCl₃
210
190
170
150
130
110
90
80
70
60
50
40
30
20
10
0
f1 (ppm)

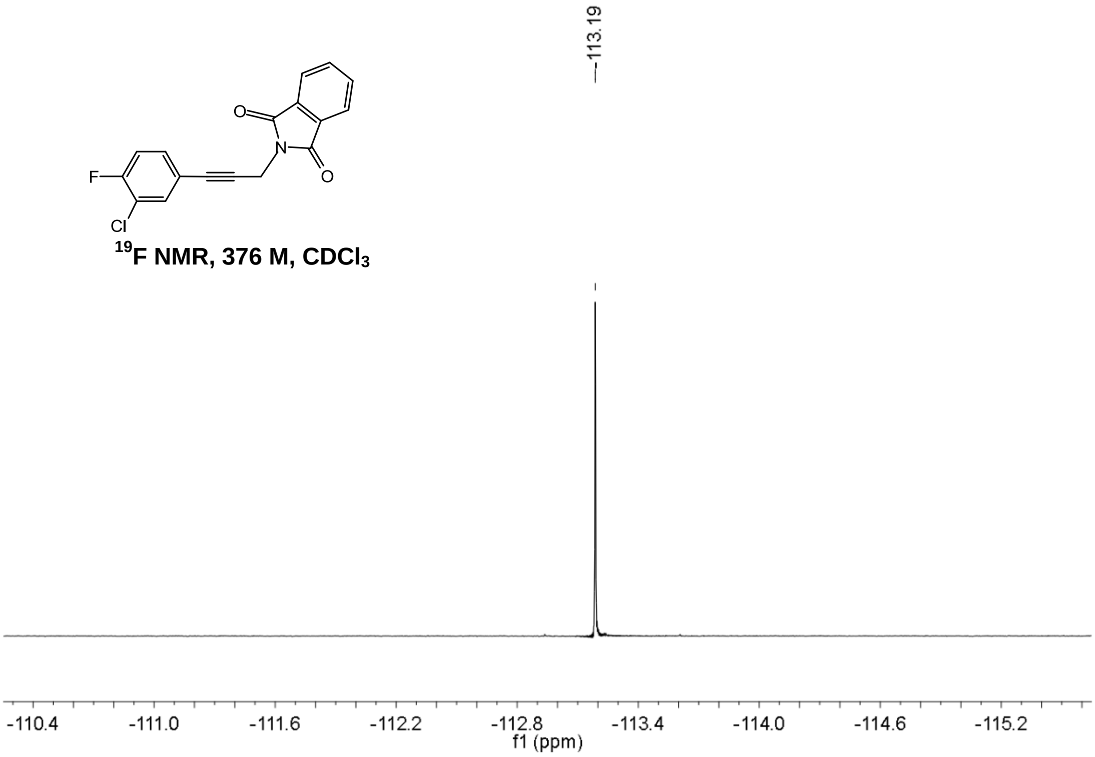

-113.19
F
Cl
O
N
O
19F NMR, 376 M, CDCl3
-110.4
-111.0
-111.6
-112.2
-112.8
-113.4
-114.0
-114.6
-115.2
f1 (ppm)

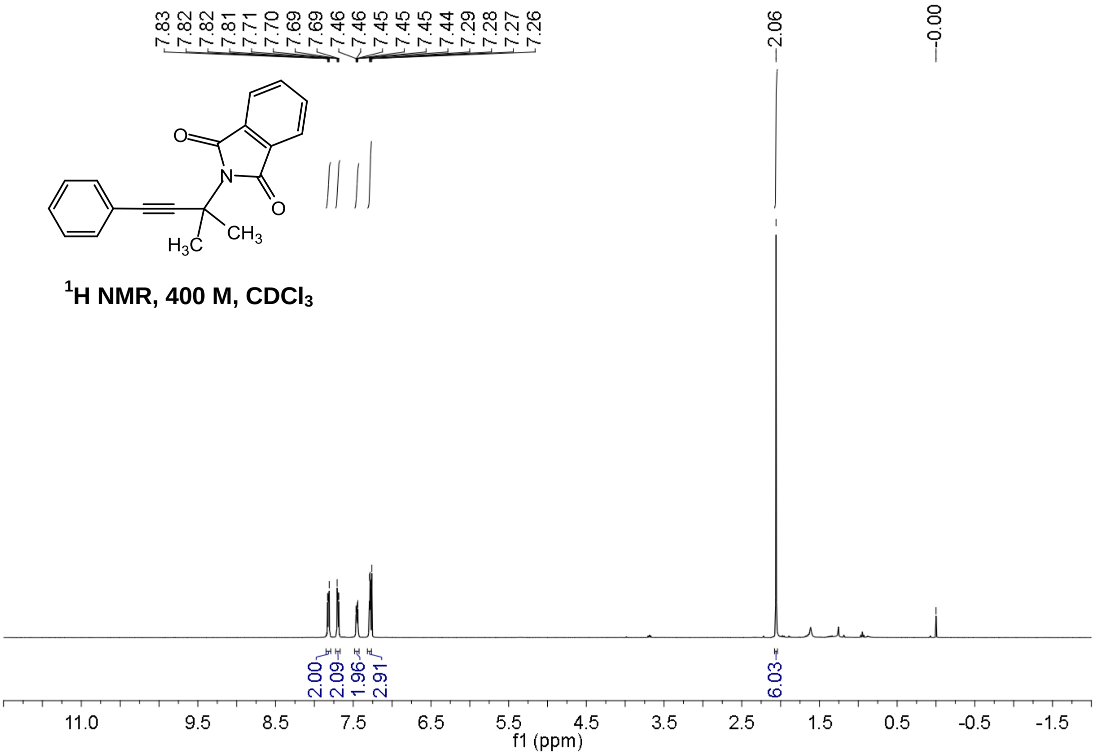

7.83 7.82 7.82 7.81 7.71 7.70 7.69 7.69 7.46 7.46 7.45 7.45 7.45 7.44 7.29 7.28 7.27 7.26
2.06
0.00
O
N
O
H3C
CH3
1H NMR, 400 M, CDCl3
2.00
2.09
1.96
2.91
6.03
11.0
9.5
8.5
7.5
6.5
5.5
4.5
3.5
2.5
1.5
0.5
-0.5
-1.5
f1 (ppm)

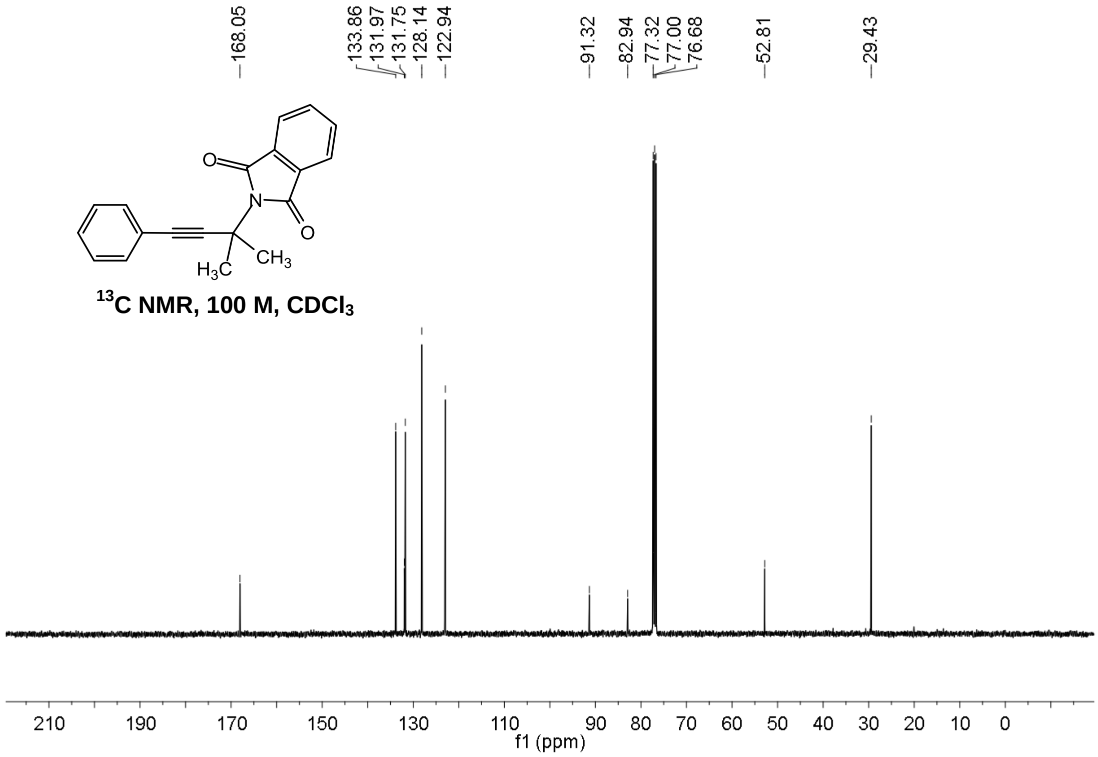

5.2 B(4,5)-diarylated compounds

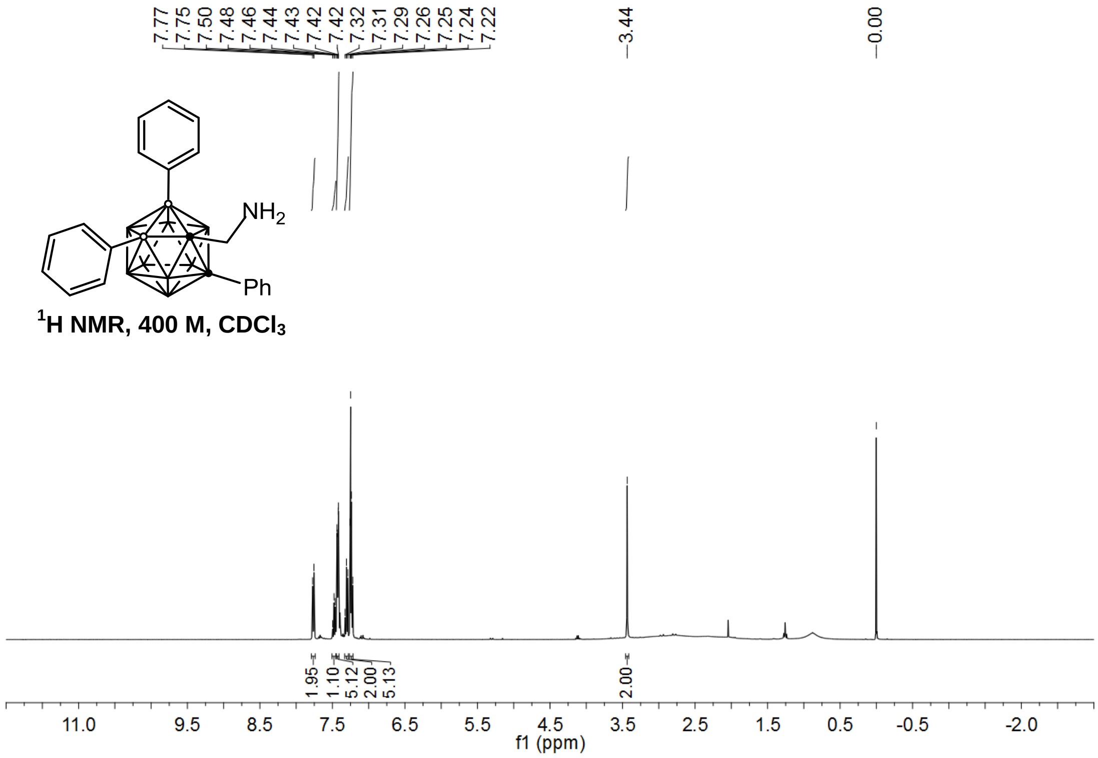

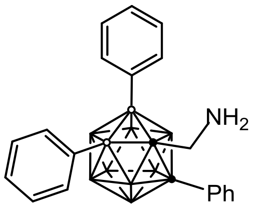

^{11}B {^{1}H} NMR, 128 M, CDCl$_3$

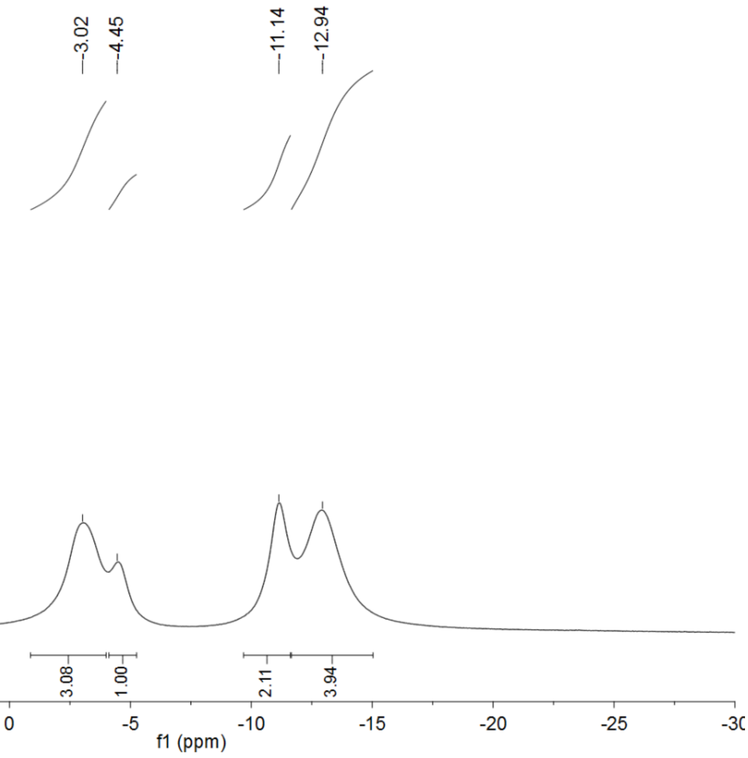

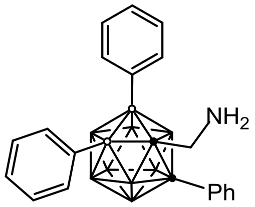

^{11}B NMR, 128 M, CDCl$_3$

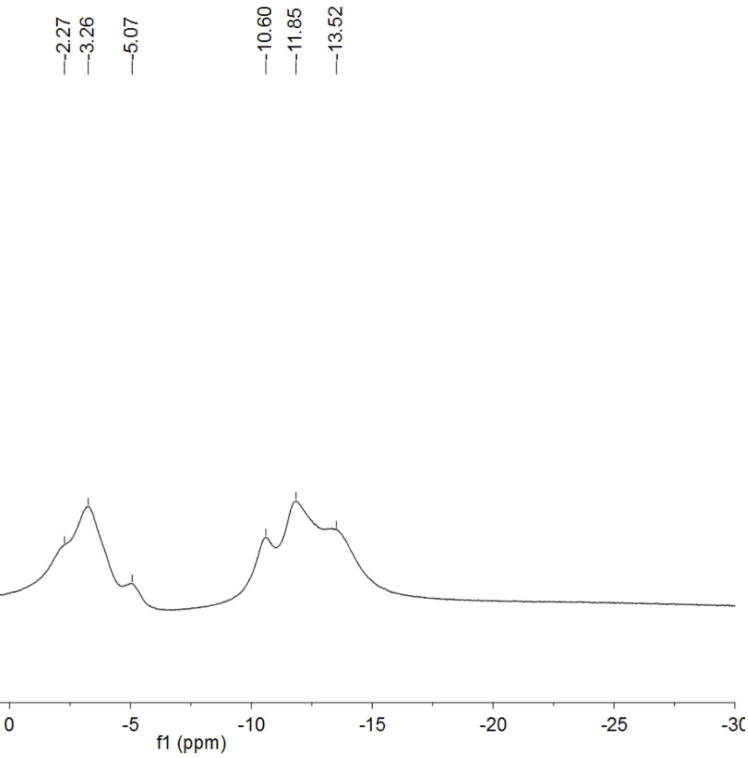

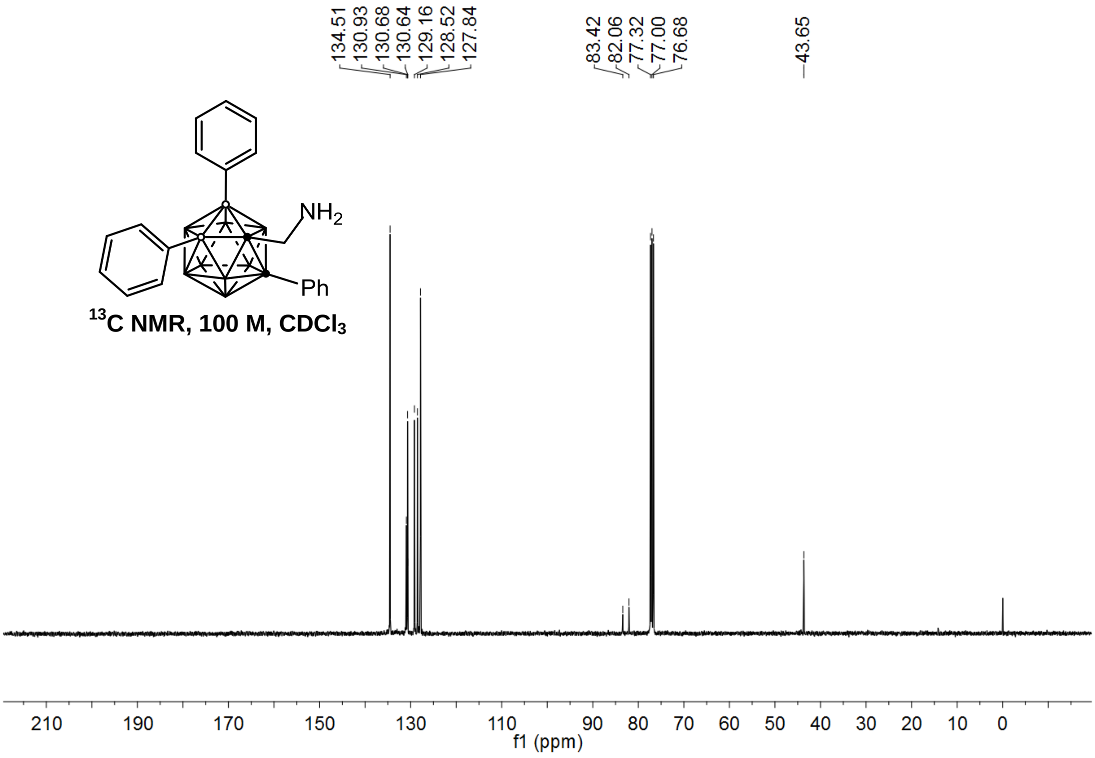
134.51
130.93
130.68
130.64
129.16
128.52
127.84
83.42
82.06
77.32
77.00
76.68
43.65
NH2
Ph
13C NMR, 100 M, CDCl3
210 190 170 150 130 110 90 80 70 60 50 40 30 20 10 0
f1 (ppm)

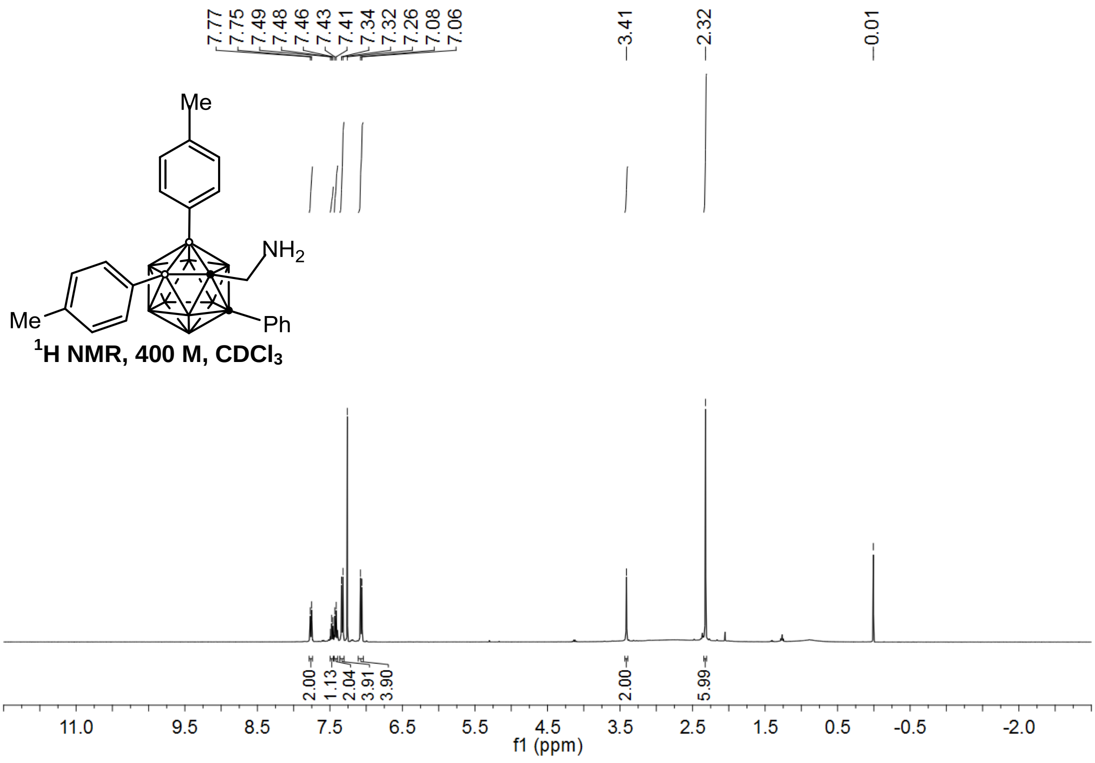
7.77
7.75
7.49
7.48
7.46
7.43
7.41
7.34
7.32
7.26
7.08
7.06
3.41
2.32
-0.01
Me
NH2
Me
Ph
1H NMR, 400 M, CDCl3
2.00
1.13
2.04
3.91
3.90
2.00
5.99
11.0 9.5 8.5 7.5 6.5 5.5 4.5 3.5 2.5 1.5 0.5 -0.5 -2.0
f1 (ppm)

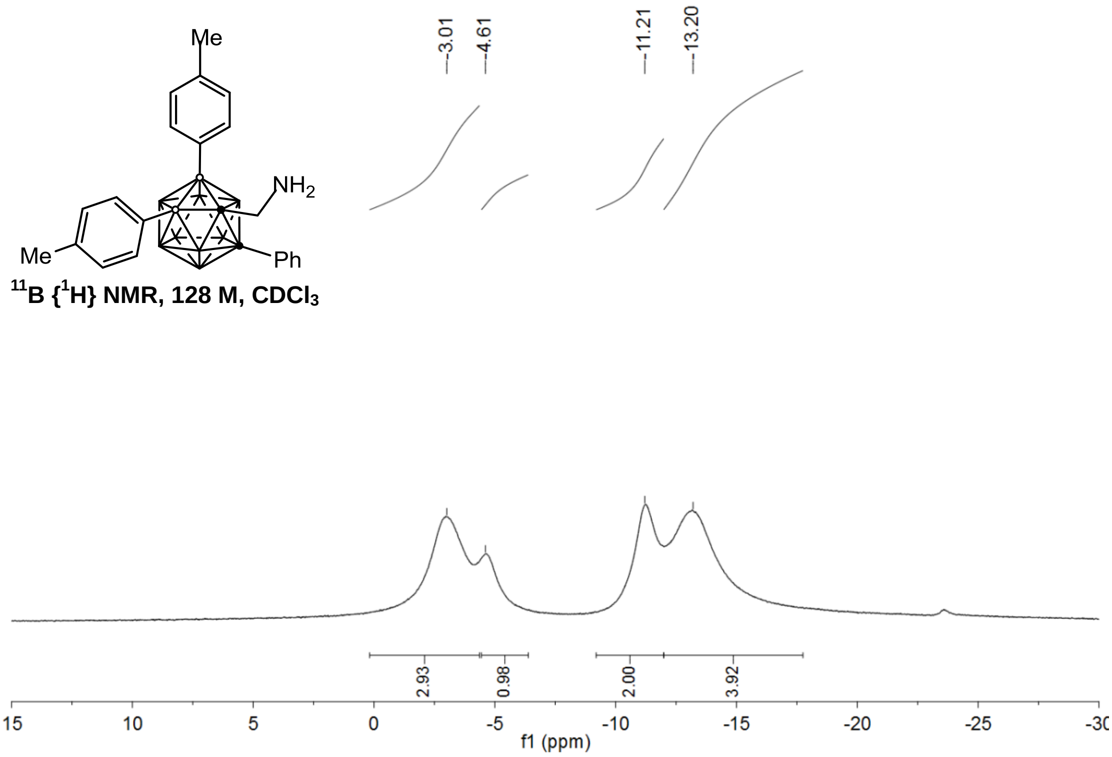
Me
NH2
Me
Ph
11B {1H} NMR, 128 M, CDCl3
3.01
4.61
11.21
13.20
2.93
0.98
2.00
3.92
15
10
5
0
-5
-10
-15
-20
-25
-30
f1 (ppm)

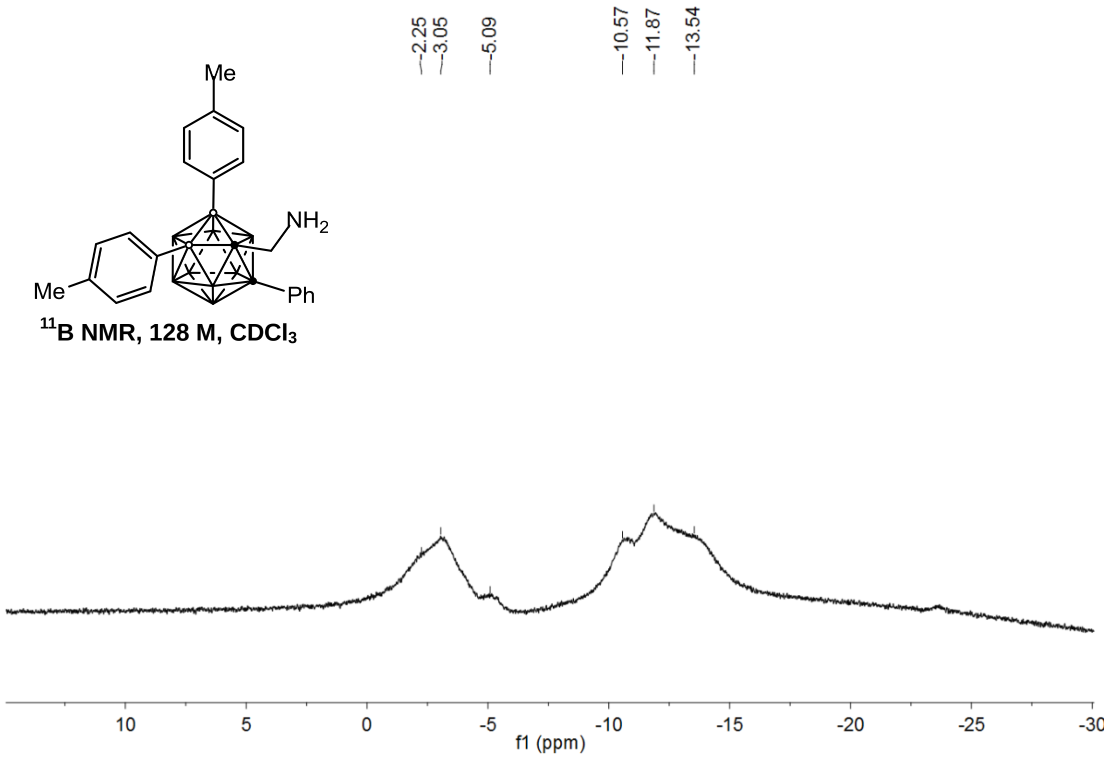
Me
NH2
Me
Ph
11B NMR, 128 M, CDCl3
2.25
3.05
5.09
10.57
11.87
13.54
10
5
0
-5
-10
-15
-20
-25
-30
f1 (ppm)

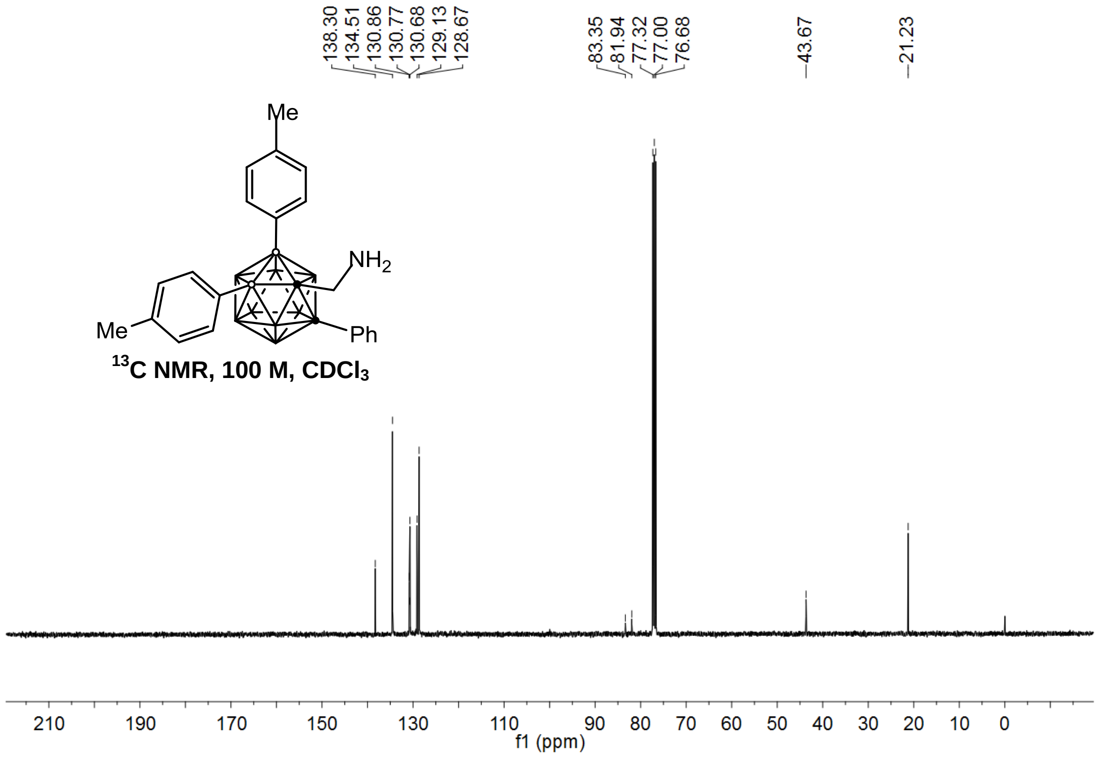
138.30
134.51
130.86
130.77
130.68
129.13
128.67
83.35
81.94
77.32
77.00
76.68
43.67
21.23
Me
NH2
Me
Ph
13C NMR, 100 M, CDCl3
210 190 170 150 130 110 90 80 70 60 50 40 30 20 10 0
f1 (ppm)

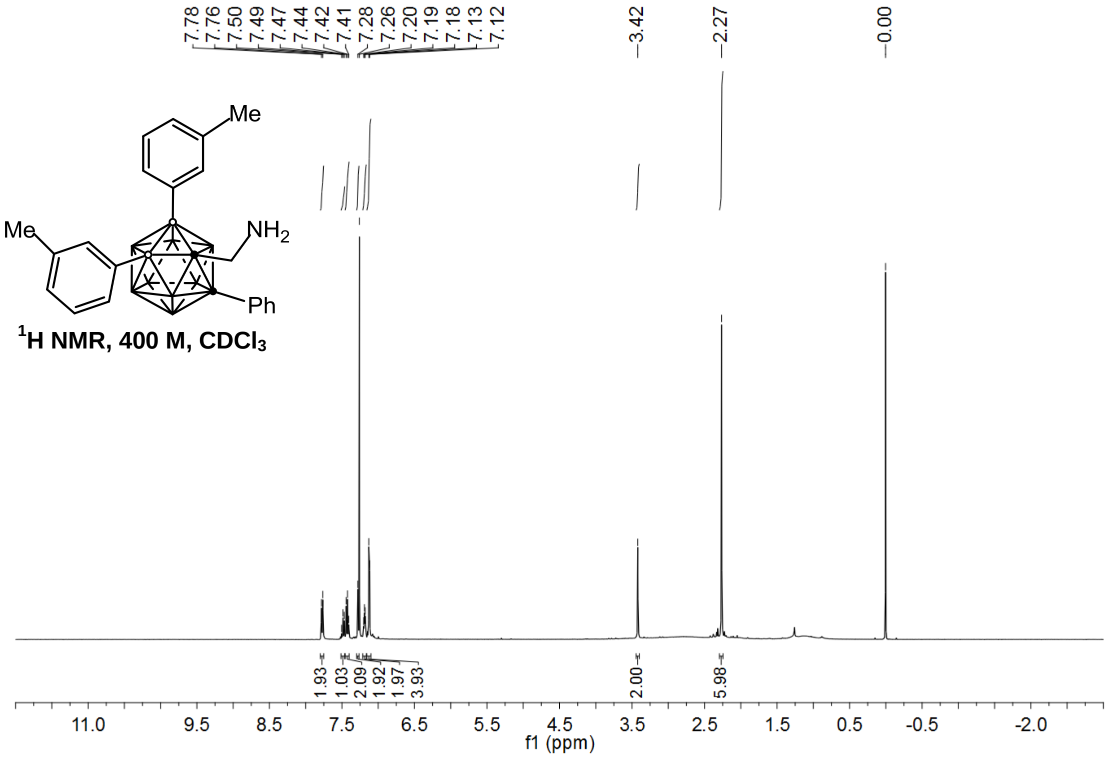
7.78
7.76
7.50
7.49
7.47
7.44
7.42
7.41
7.28
7.26
7.20
7.19
7.18
7.13
7.12
3.42
2.27
0.00
Me
Me
NH2
Ph
1H NMR, 400 M, CDCl3
1.93
1.03
2.09
1.92
1.97
3.93
2.00
5.98
11.0 9.5 8.5 7.5 6.5 5.5 4.5 3.5 2.5 1.5 0.5 -0.5 -2.0
f1 (ppm)

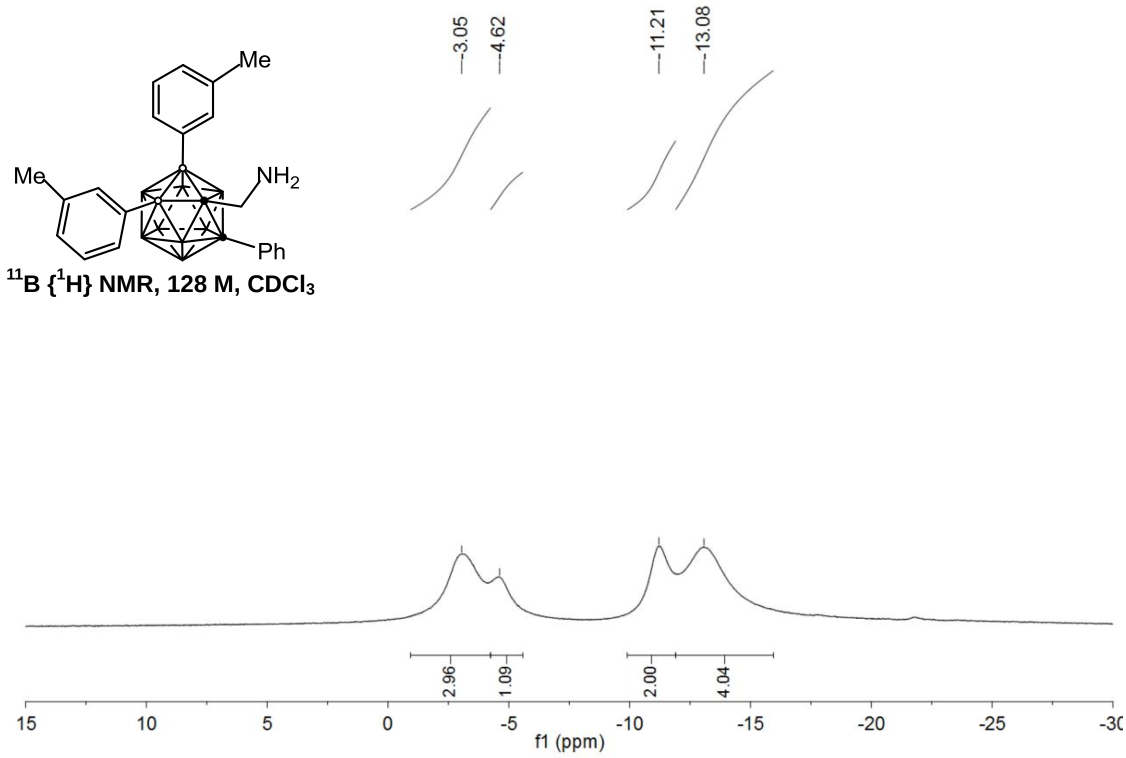

Me
Me
NH2
Ph
11B {1H} NMR, 128 M, CDCl3
3.05
4.62
11.21
13.08
2.96
1.09
2.00
4.04
f1 (ppm)

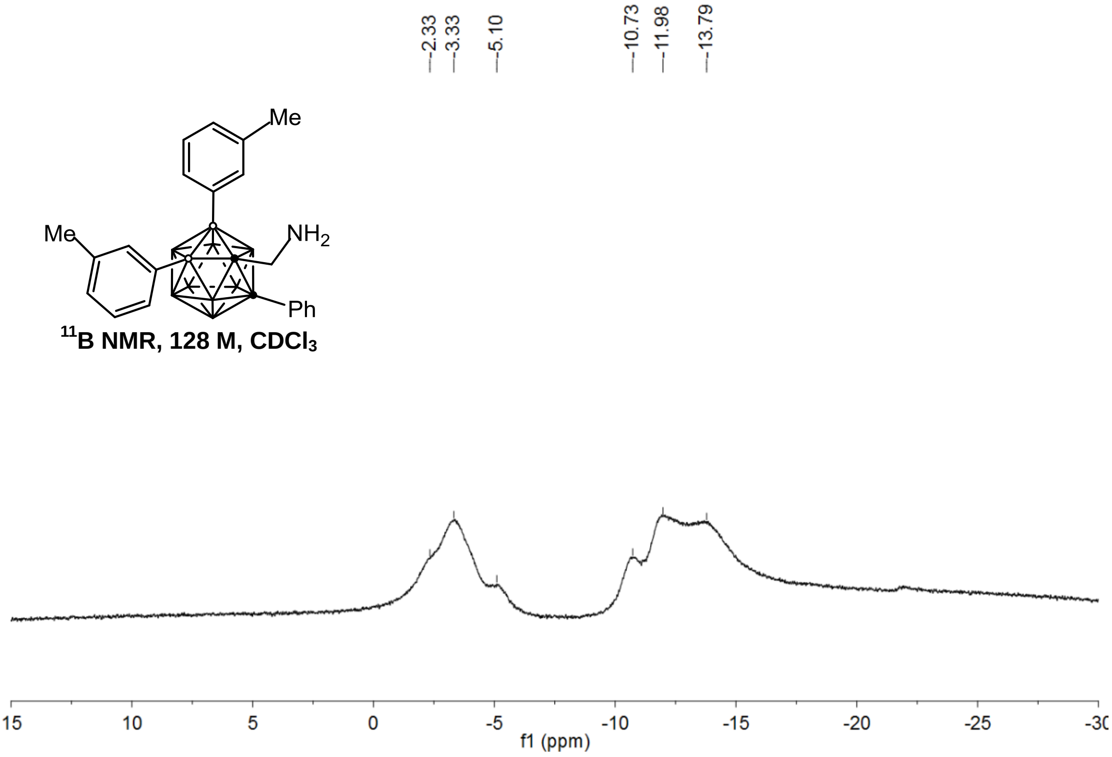

Me
Me
NH2
Ph
11B NMR, 128 M, CDCl3
2.33
3.33
5.10
10.73
11.98
13.79
f1 (ppm)

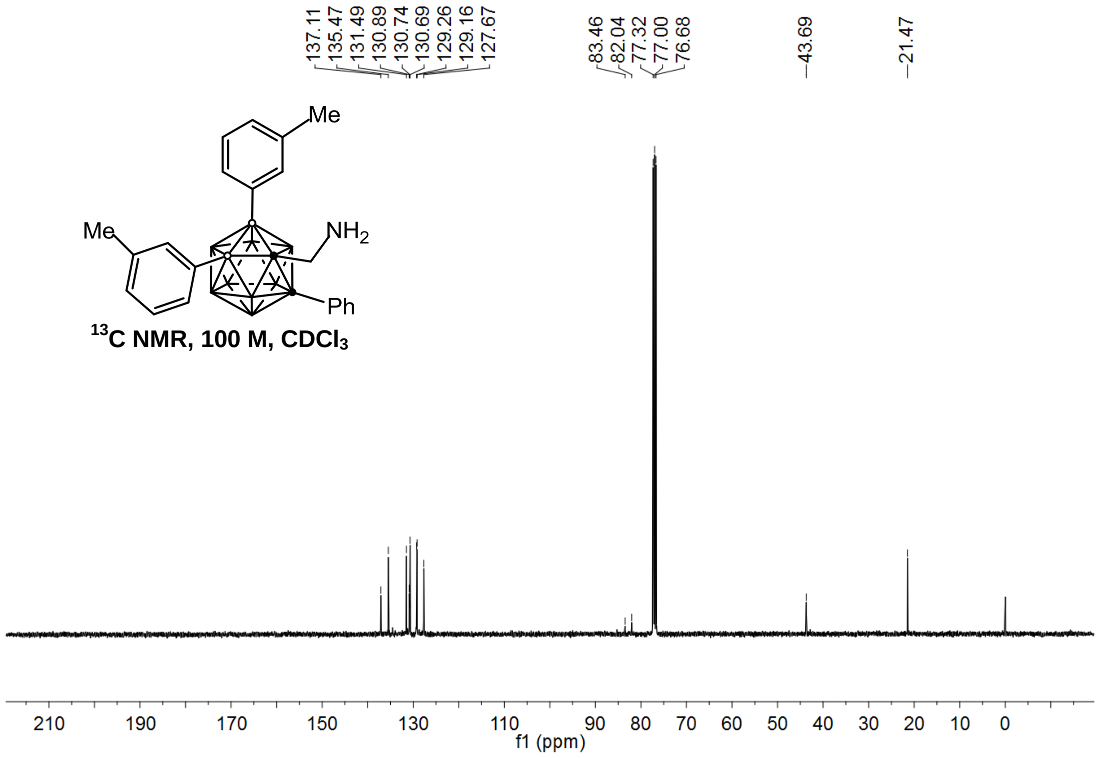

137.11
135.47
131.49
130.89
130.74
130.69
129.26
129.16
127.67
83.46
82.04
77.32
77.00
76.68
43.69
21.47
Me
Me
NH2
Ph
13C NMR, 100 M, CDCl3
f1 (ppm)

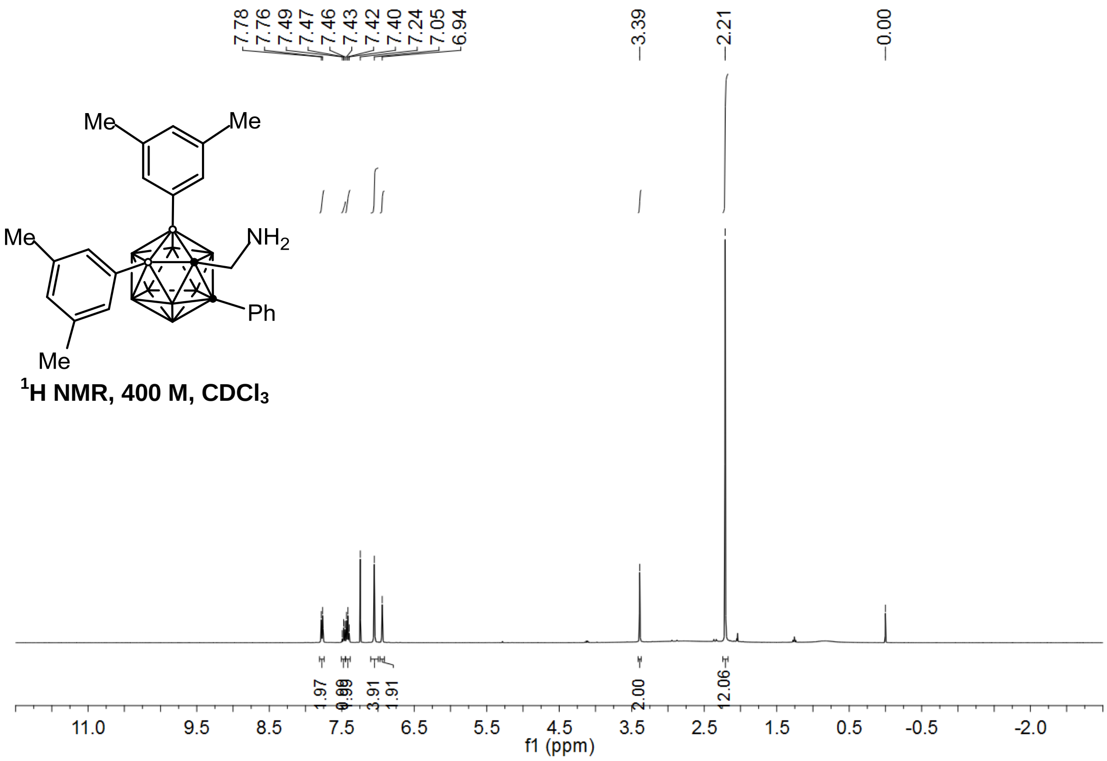

7.78
7.76
7.49
7.47
7.46
7.43
7.42
7.40
7.24
7.05
6.94
3.39
2.21
0.00
Me
Me
Me
Me
NH2
Ph
1H NMR, 400 M, CDCl3
1.97
3.91
1.91
2.00
12.06
f1 (ppm)

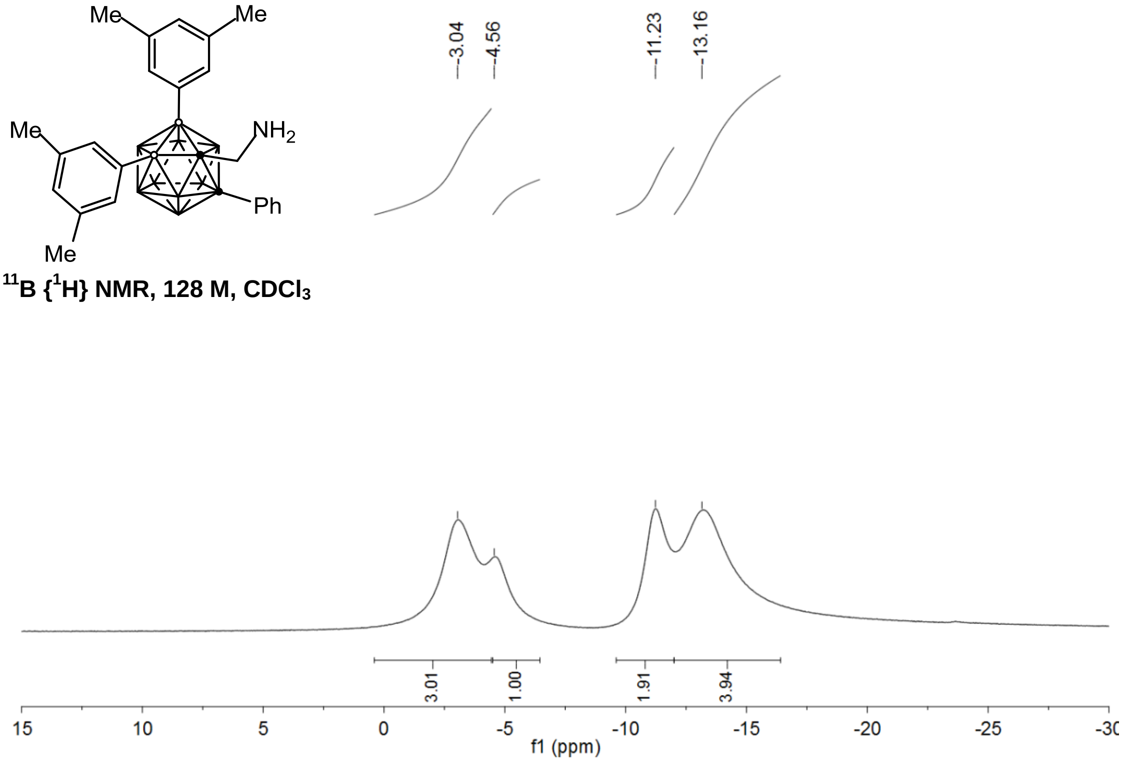
Me
Me
Me
NH2
Ph
Me
11B {1H} NMR, 128 M, CDCl3
-3.04
-4.56
-11.23
-13.16
3.01
1.00
1.91
3.94
15 10 5 0 -5 -10 -15 -20 -25 -30
f1 (ppm)

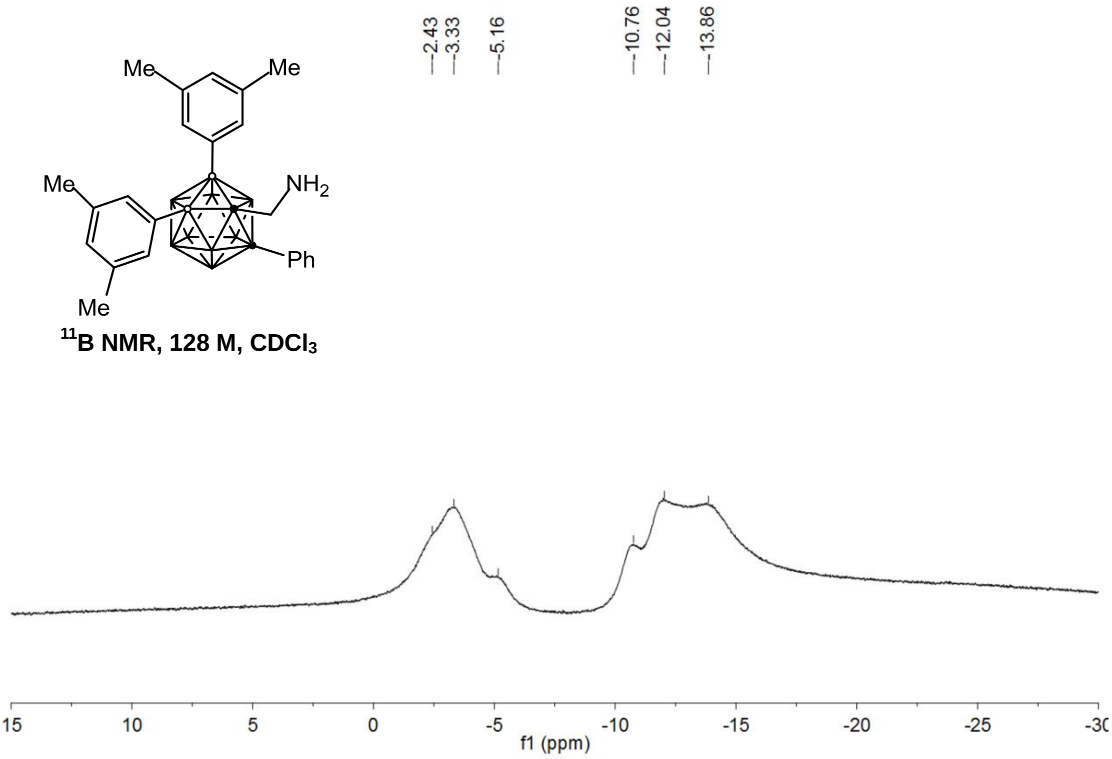
Me
Me
Me
NH2
Ph
Me
11B NMR, 128 M, CDCl3
-2.43
-3.33
-5.16
-10.76
-12.04
-13.86
15 10 5 0 -5 -10 -15 -20 -25 -30
f1 (ppm)

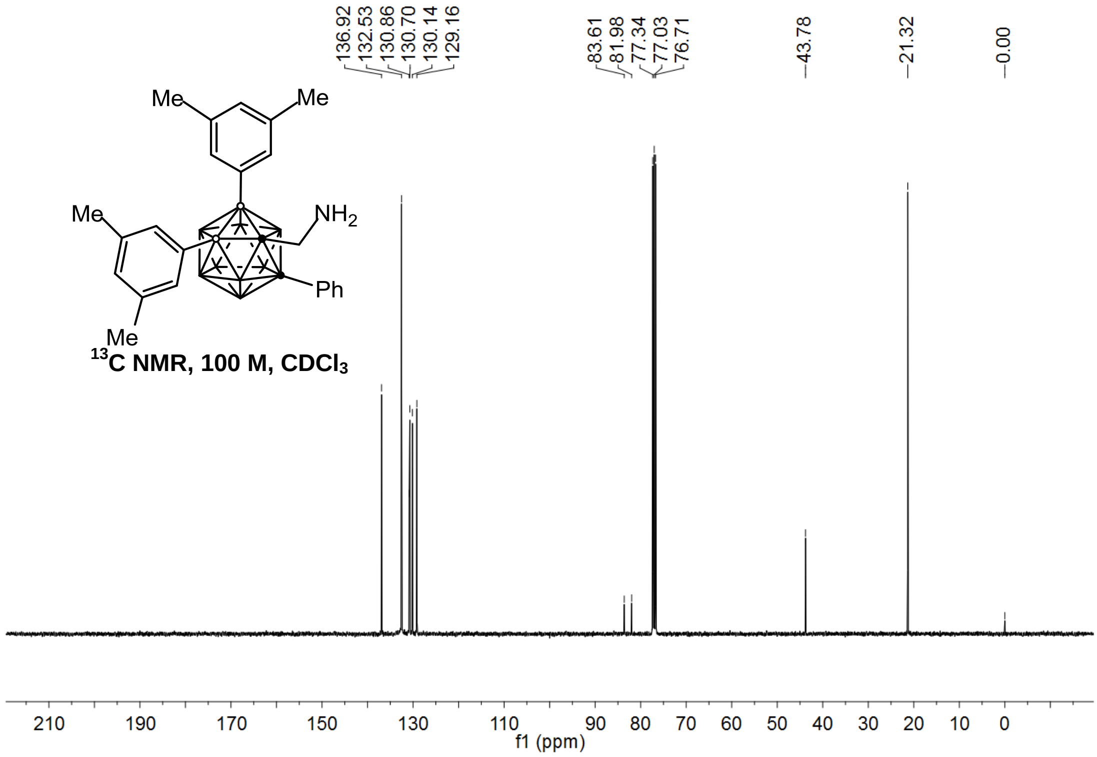
136.92
132.53
130.86
130.70
130.14
129.16
83.61
81.98
77.34
77.03
76.71
43.78
21.32
0.00
Me
Me
Me
Me
NH2
Ph
13C NMR, 100 M, CDCl3
210 190 170 150 130 110 90 80 70 60 50 40 30 20 10 0
f1 (ppm)

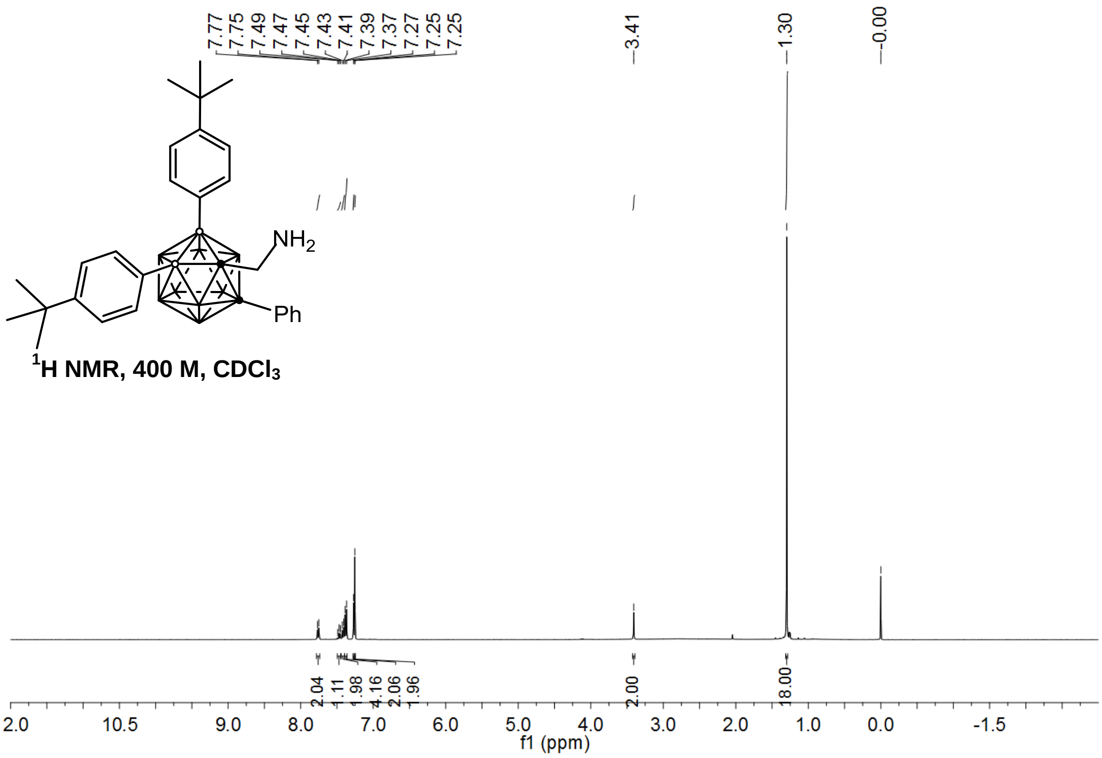
7.77
7.75
7.49
7.47
7.45
7.43
7.41
7.39
7.37
7.27
7.25
7.25
3.41
1.30
0.00
NH2
Ph
1H NMR, 400 M, CDCl3
2.04
1.11
1.98
4.16
2.06
1.96
2.00
18.00
2.0 10.5 9.0 8.0 7.0 6.0 5.0 4.0 3.0 2.0 1.0 0.0 -1.5
f1 (ppm)

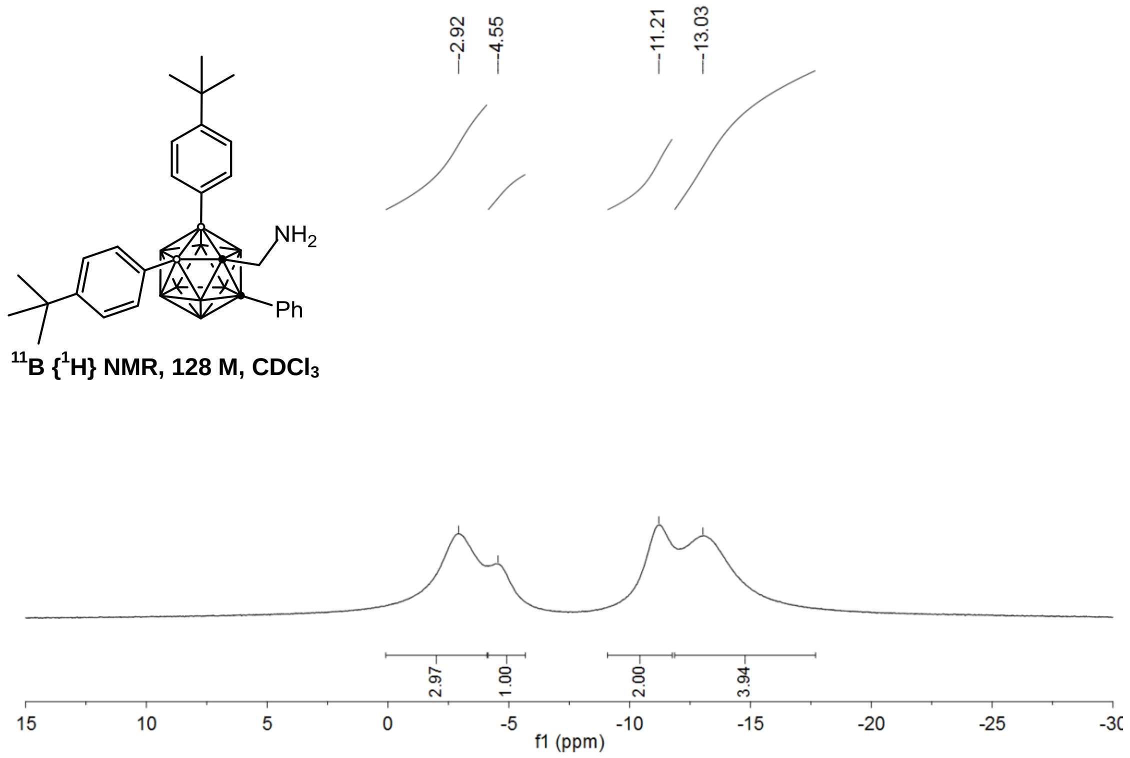
2.92
4.55
11.21
13.03
NH2
Ph
11B {1H} NMR, 128 M, CDCl3
2.97
1.00
2.00
3.94
15
10
5
0
-5
-10
-15
-20
-25
-30
f1 (ppm)

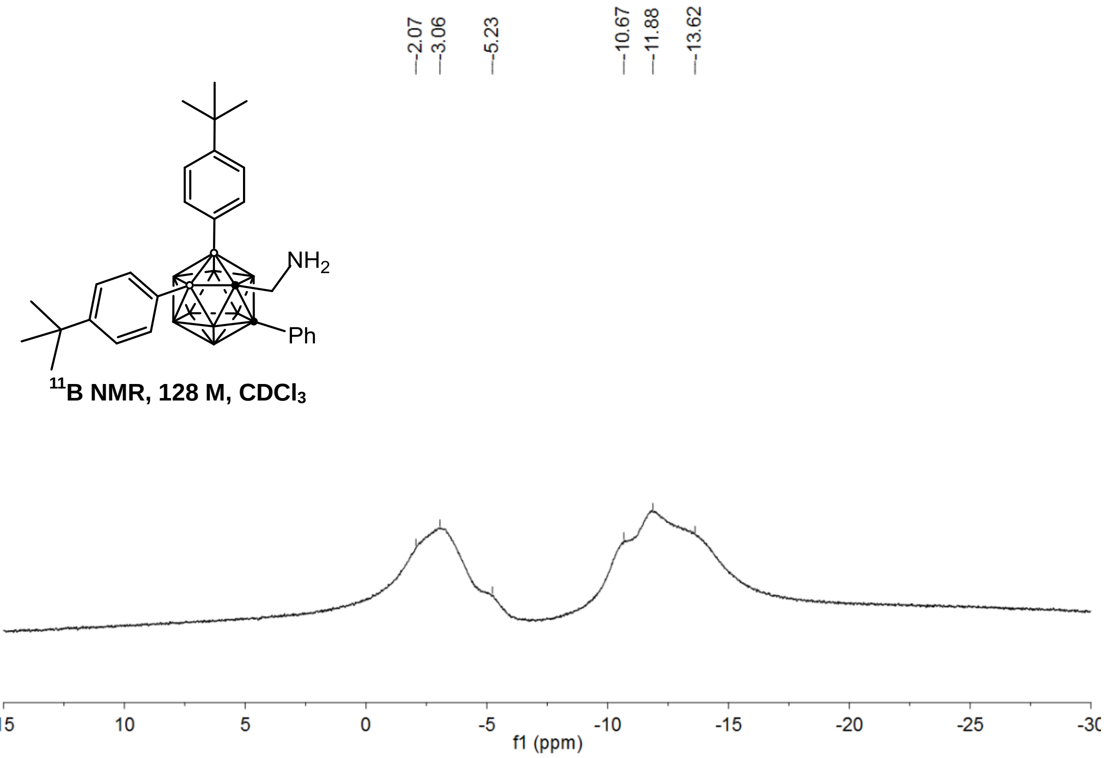
2.07
3.06
5.23
10.67
11.88
13.62
NH2
Ph
11B NMR, 128 M, CDCl3
15
10
5
0
-5
-10
-15
-20
-25
-30
f1 (ppm)

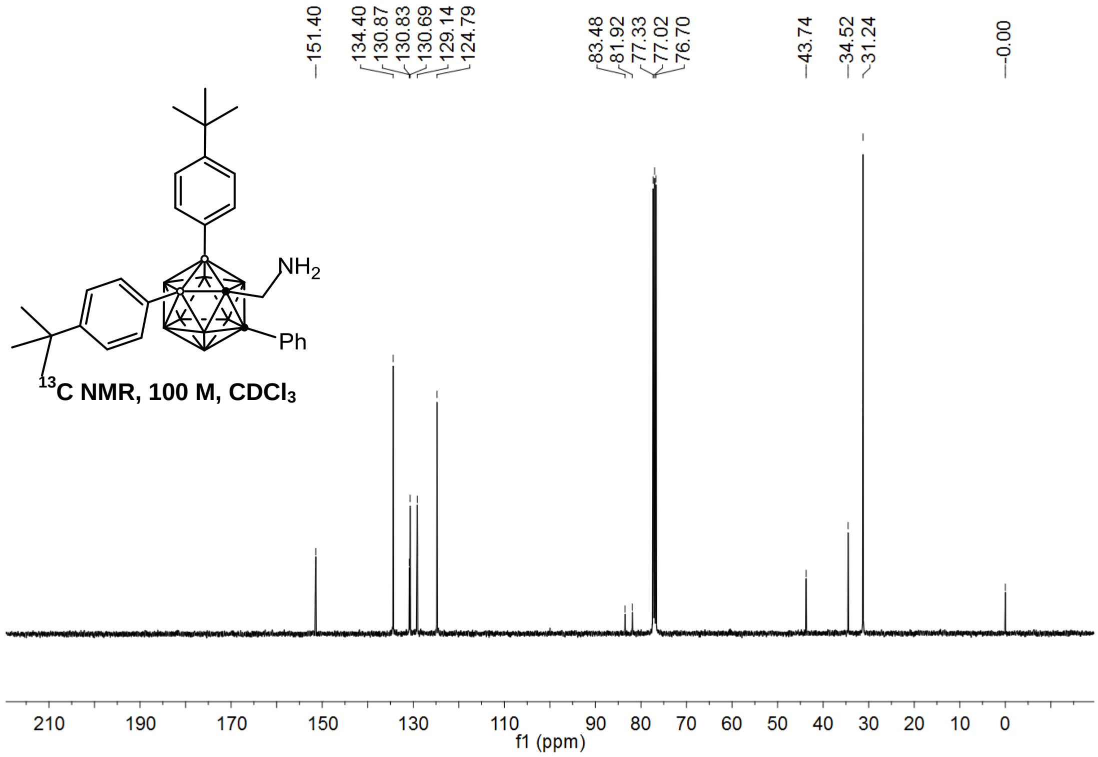
151.40
134.40
130.87
130.83
130.69
129.14
124.79
83.48
81.92
77.33
77.02
76.70
43.74
34.52
31.24
0.00
NH2
Ph
13C NMR, 100 M, CDCl3
f1 (ppm)

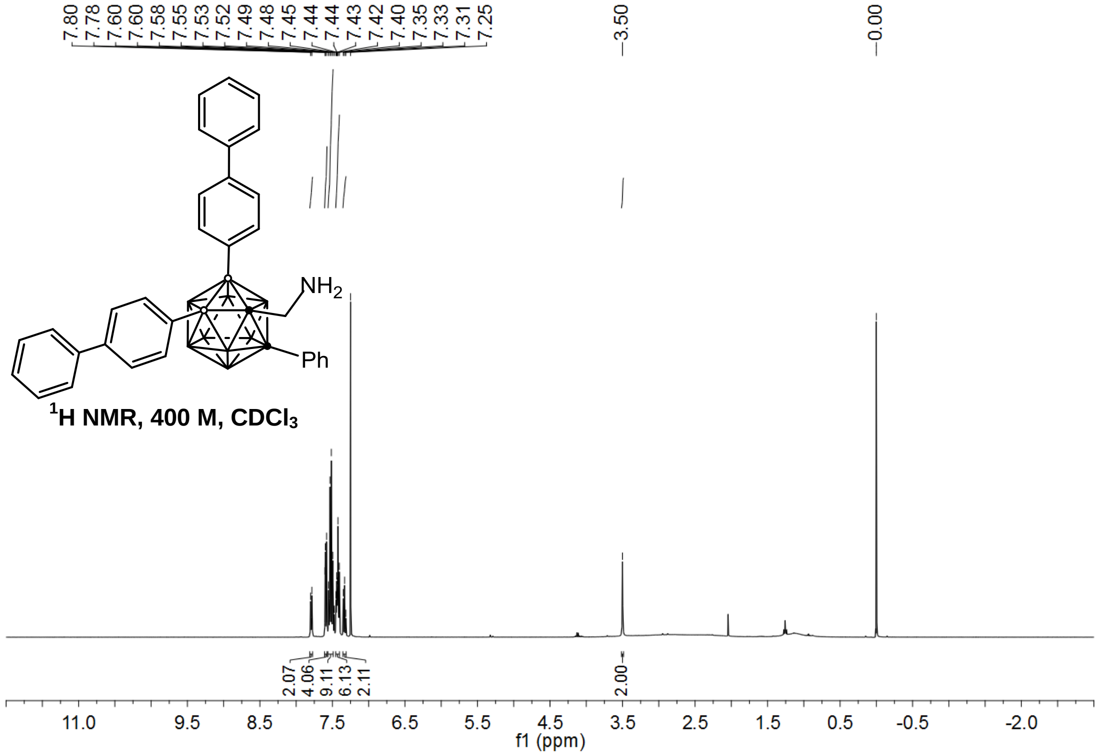
7.80
7.78
7.60
7.60
7.58
7.55
7.53
7.52
7.49
7.48
7.45
7.44
7.44
7.43
7.42
7.40
7.35
7.33
7.31
7.25
3.50
0.00
NH2
Ph
1H NMR, 400 M, CDCl3
2.07
4.06
9.11
6.13
2.11
2.00
f1 (ppm)

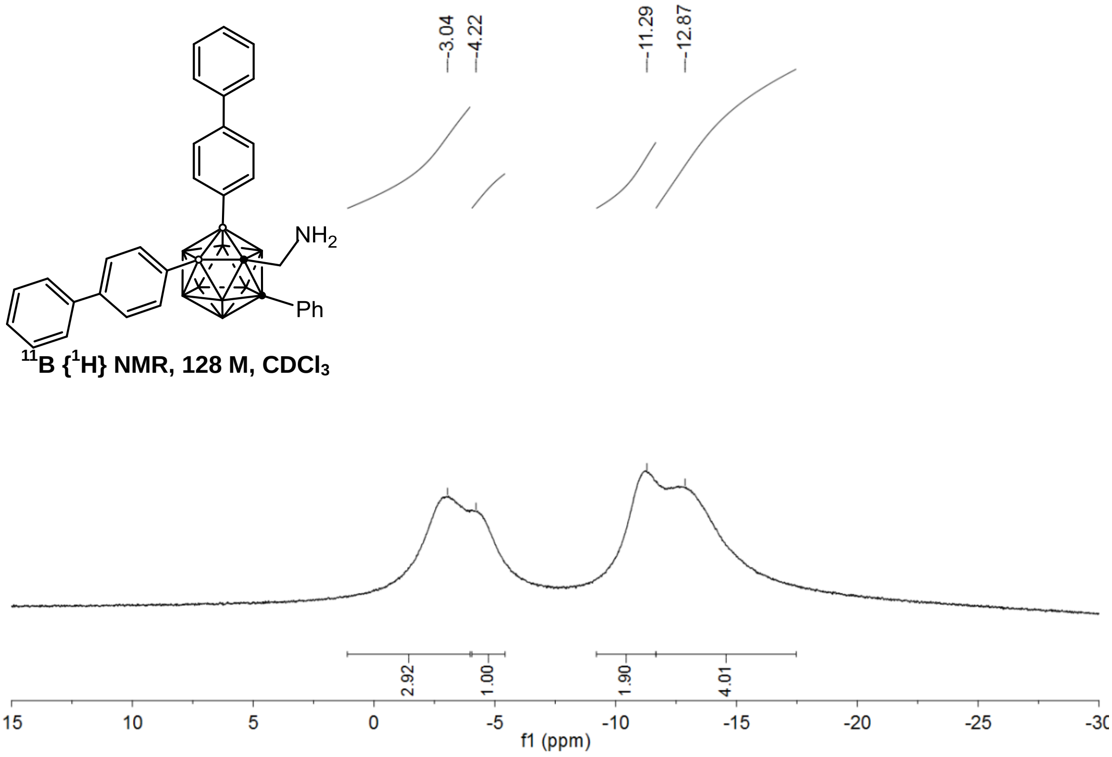

NH2
Ph
11B {1H} NMR, 128 M, CDCl3
-3.04
-4.22
-11.29
-12.87
2.92
1.00
1.90
4.01
15
10
5
0
-5
-10
-15
-20
-25
-30
f1 (ppm)

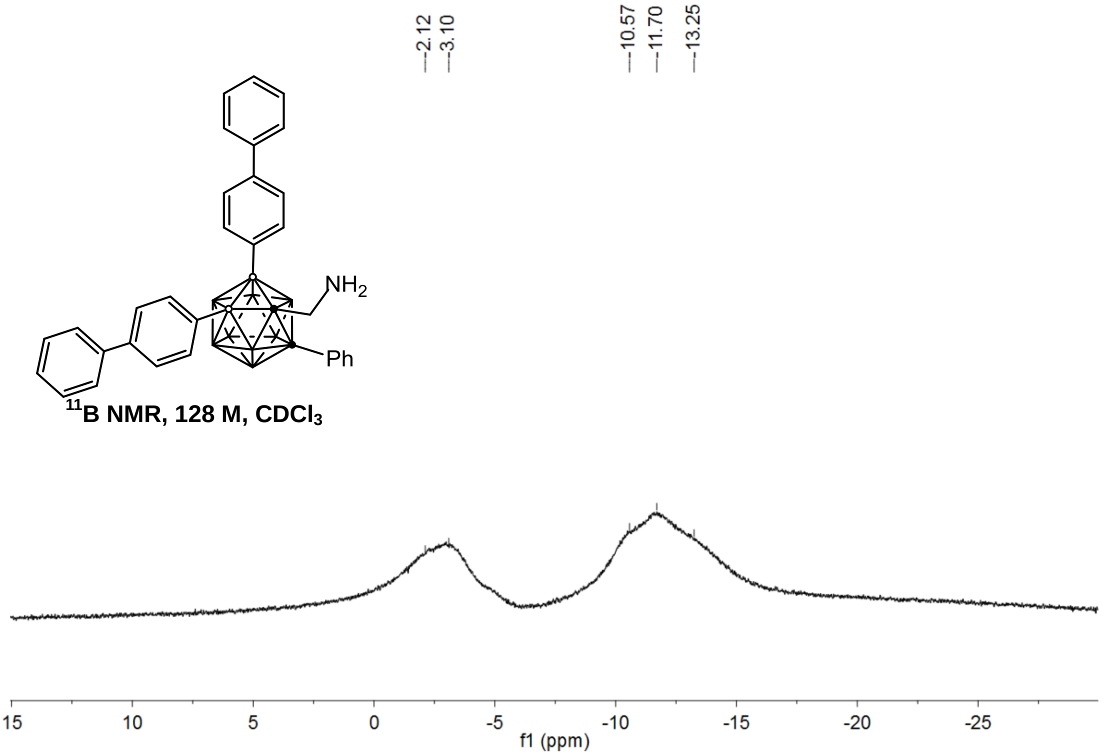

NH2
Ph
11B NMR, 128 M, CDCl3
-2.12
-3.10
-10.57
-11.70
-13.25
15
10
5
0
-5
-10
-15
-20
-25
f1 (ppm)

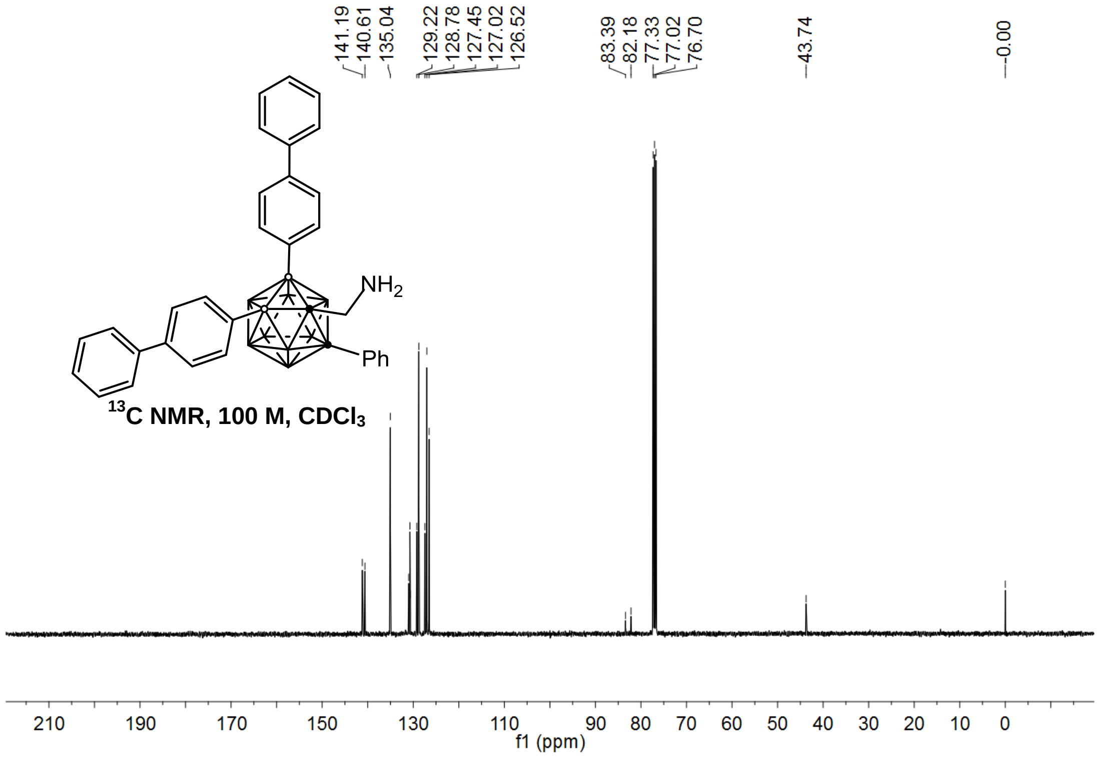
141.19
140.61
135.04
129.22
128.78
127.45
127.02
126.52
83.39
82.18
77.33
77.02
76.70
43.74
-0.00
NH2
Ph
13C NMR, 100 M, CDCl3
210 190 170 150 130 110 90 80 70 60 50 40 30 20 10 0
f1 (ppm)

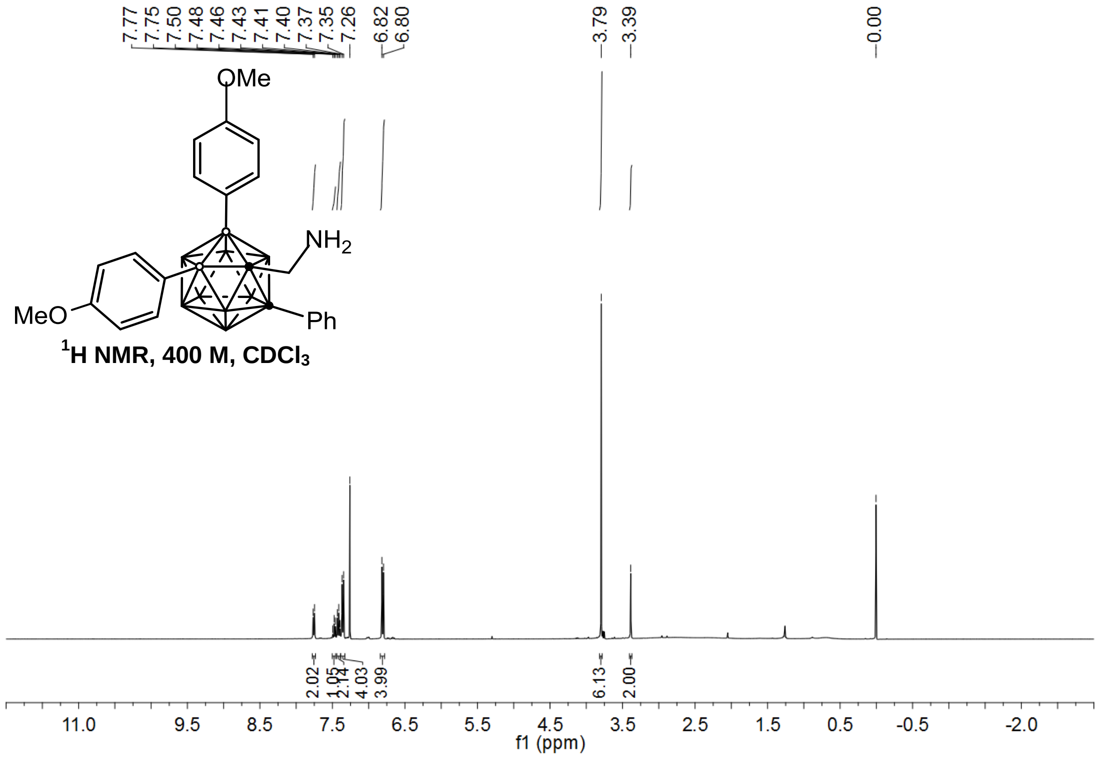
7.77
7.75
7.50
7.48
7.46
7.43
7.41
7.40
7.37
7.35
7.26
6.82
6.80
3.79
3.39
0.00
OMe
NH2
MeO
Ph
1H NMR, 400 M, CDCl3
2.02
1.05
2.14
4.03
3.99
6.13
2.00
11.0 9.5 8.5 7.5 6.5 5.5 4.5 3.5 2.5 1.5 0.5 -0.5 -2.0
f1 (ppm)

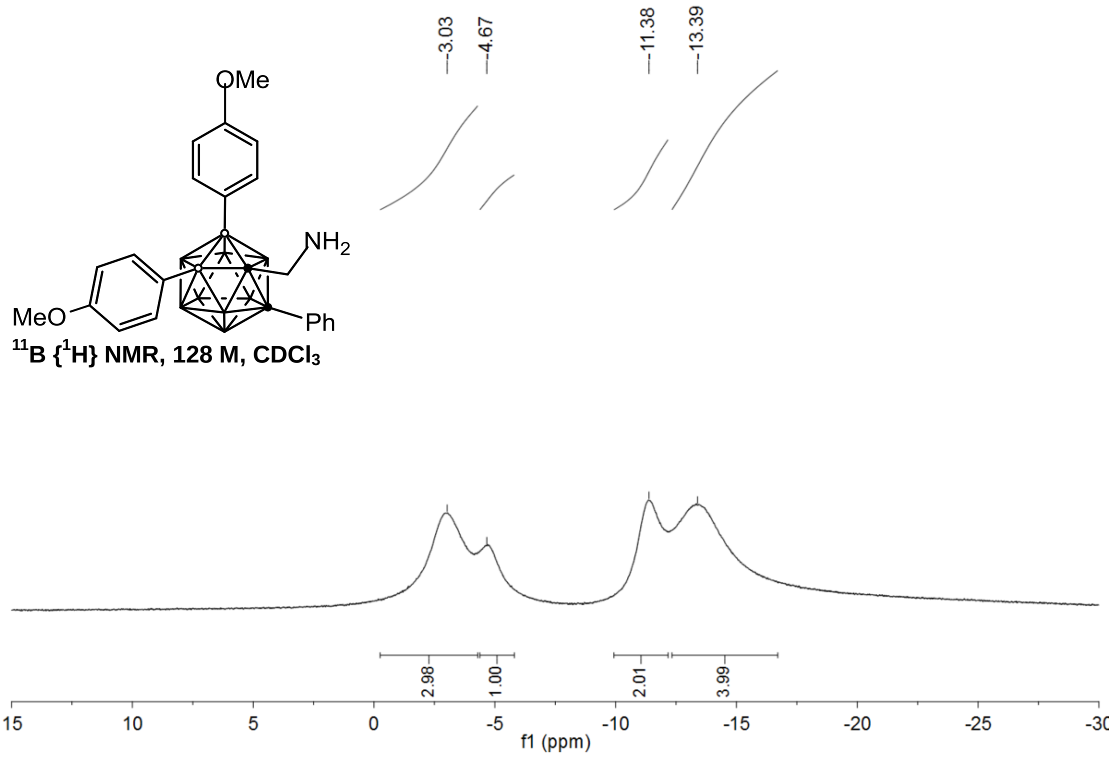
OMe
NH2
MeO
Ph
11B {1H} NMR, 128 M, CDCl3
-3.03
-4.67
-11.38
-13.39
2.98
1.00
2.01
3.99
15
10
5
0
-5
-10
-15
-20
-25
f1 (ppm)

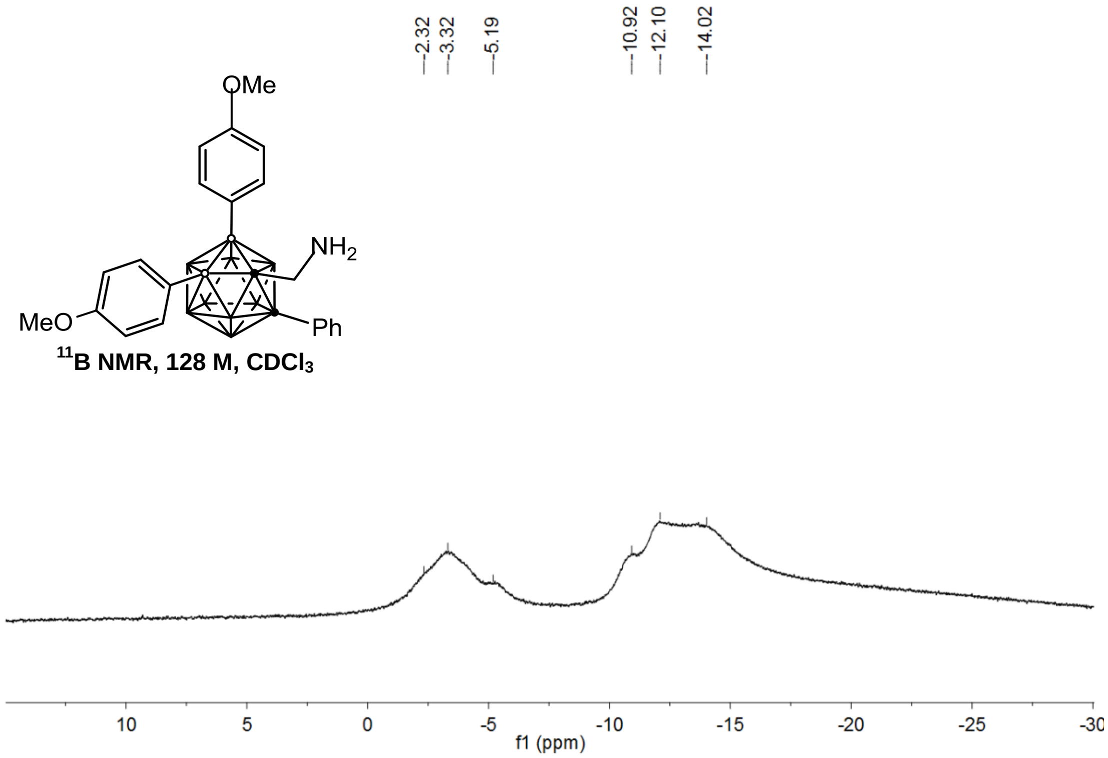
OMe
NH2
MeO
Ph
11B NMR, 128 M, CDCl3
-2.32
-3.32
-5.19
-10.92
-12.10
-14.02
10
5
0
-5
-10
-15
-20
-25
f1 (ppm)

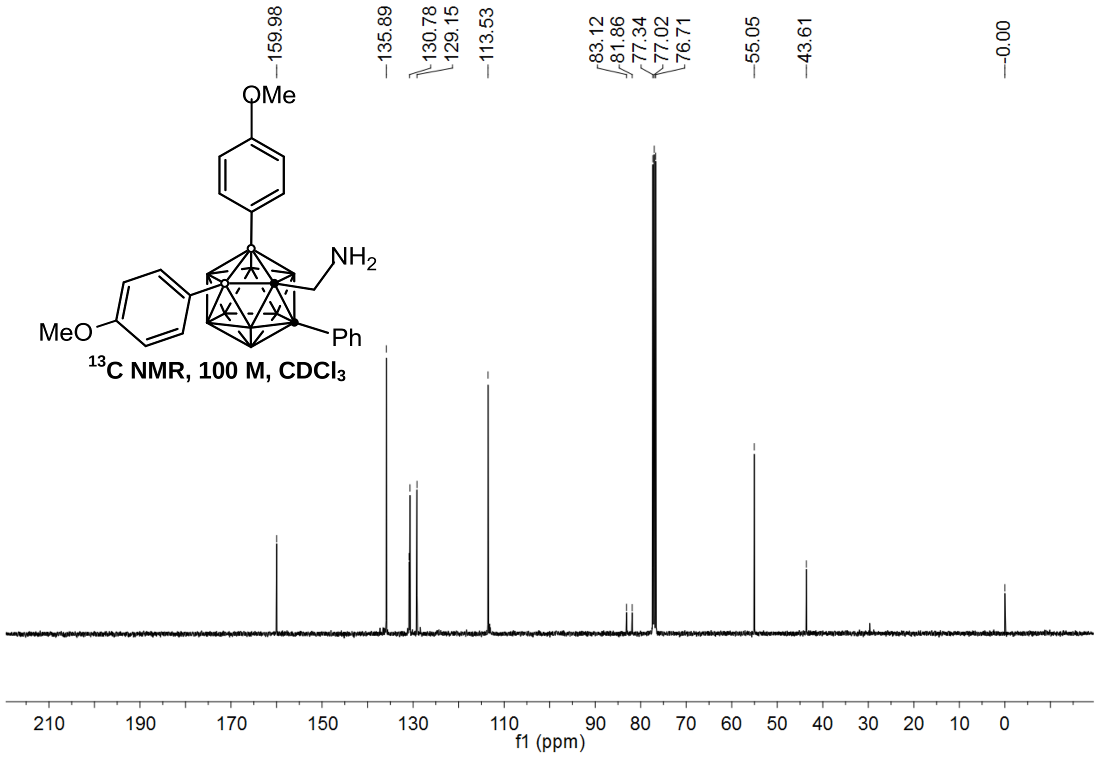
159.98
135.89
130.78
129.15
113.53
83.12
81.86
77.34
77.02
76.71
55.05
43.61
0.00
OMe
NH2
MeO
Ph
13C NMR, 100 M, CDCl3
210 190 170 150 130 110 90 80 70 60 50 40 30 20 10 0
f1 (ppm)

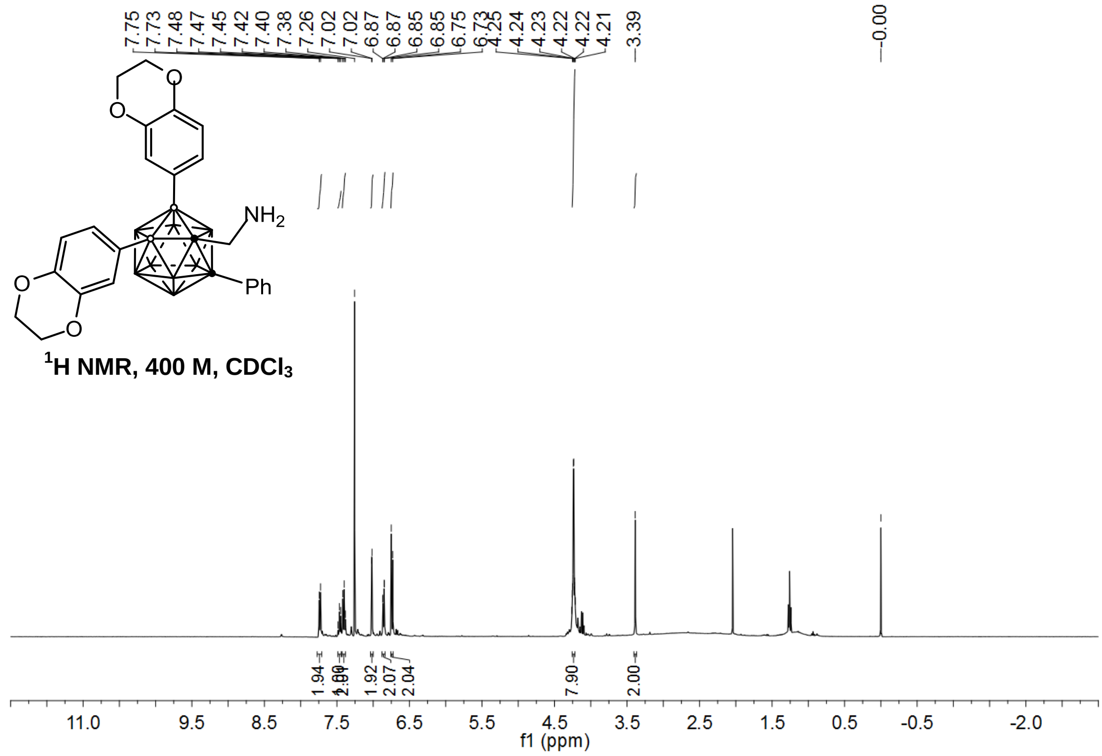
7.75
7.73
7.48
7.47
7.45
7.42
7.40
7.38
7.26
7.02
7.02
6.87
6.87
6.85
6.75
6.73
4.25
4.24
4.23
4.22
4.22
4.21
3.39
0.00
O
O
NH2
O
O
Ph
1H NMR, 400 M, CDCl3
1.94
2.07
1.92
2.07
2.04
7.90
2.00
11.0 9.5 8.5 7.5 6.5 5.5 4.5 3.5 2.5 1.5 0.5 -0.5 -2.0
f1 (ppm)

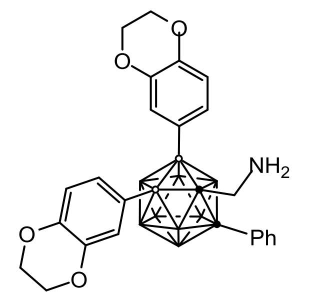

$^{11}B\{^1H\}$ NMR, 128 M, $CDCl_3$

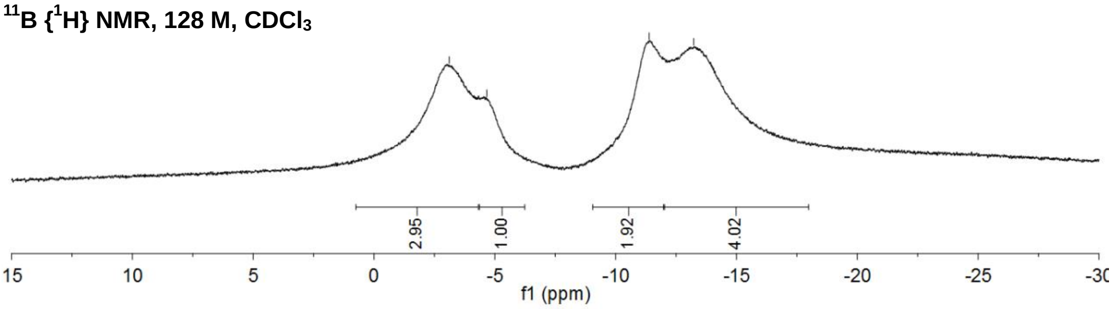

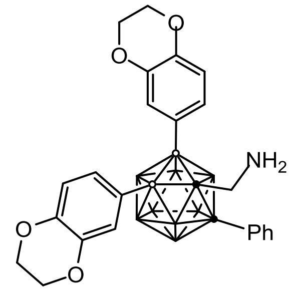

^{11}B NMR, 128 M, $CDCl_3$

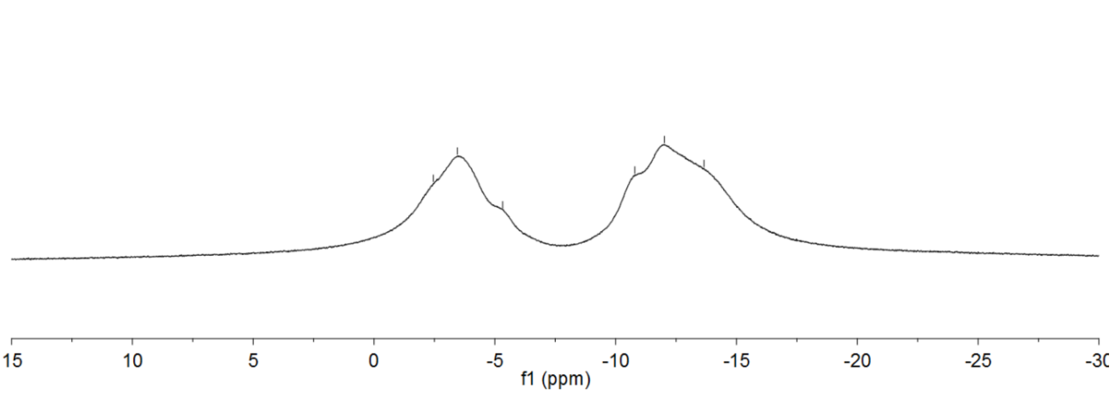

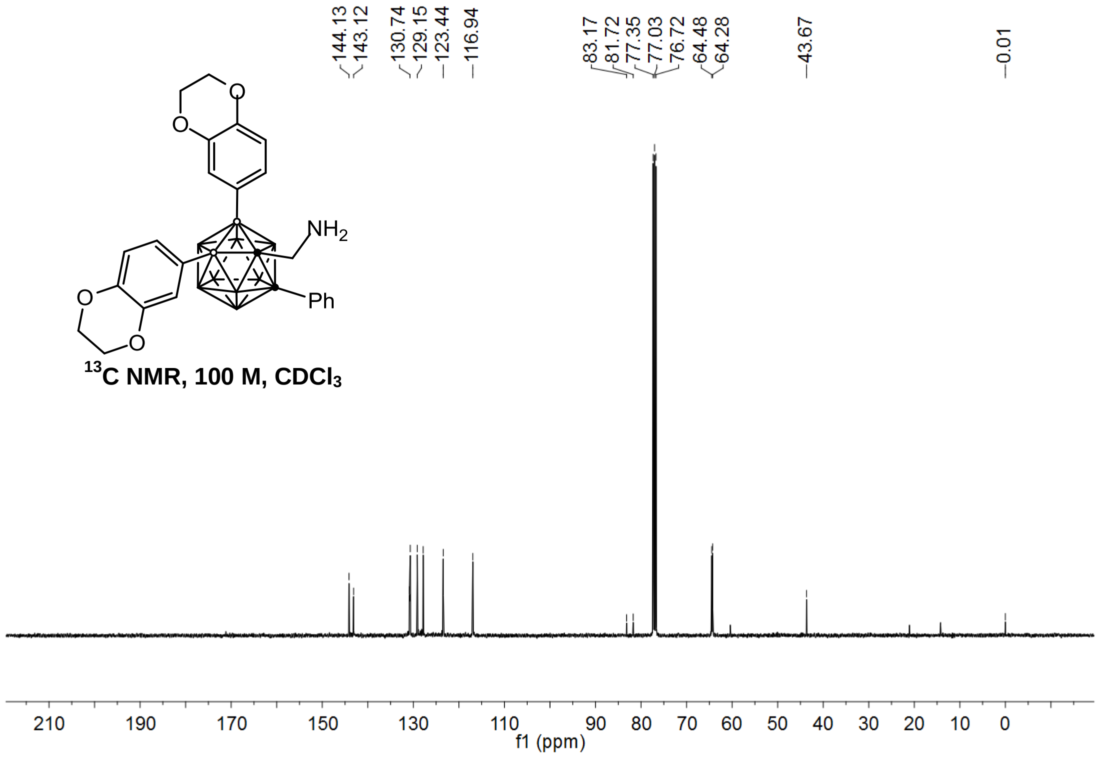
144.13
143.12
130.74
129.15
123.44
116.94
83.17
81.72
77.35
77.03
76.72
64.48
64.28
43.67
-0.01
NH2
Ph
O
13C NMR, 100 M, CDCl3
f1 (ppm)

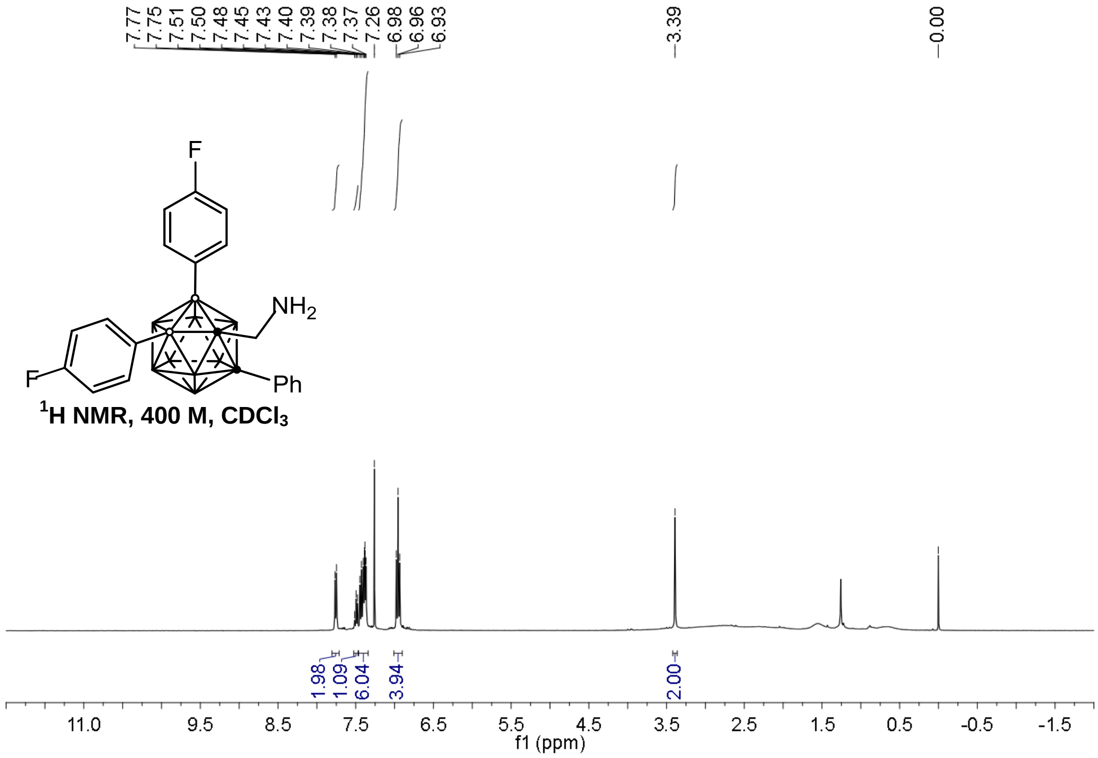
7.77
7.75
7.51
7.50
7.48
7.45
7.43
7.40
7.39
7.38
7.37
7.26
6.98
6.96
6.93
3.39
0.00
F
NH2
Ph
1H NMR, 400 M, CDCl3
1.98
1.09
6.04
3.94
2.00
f1 (ppm)

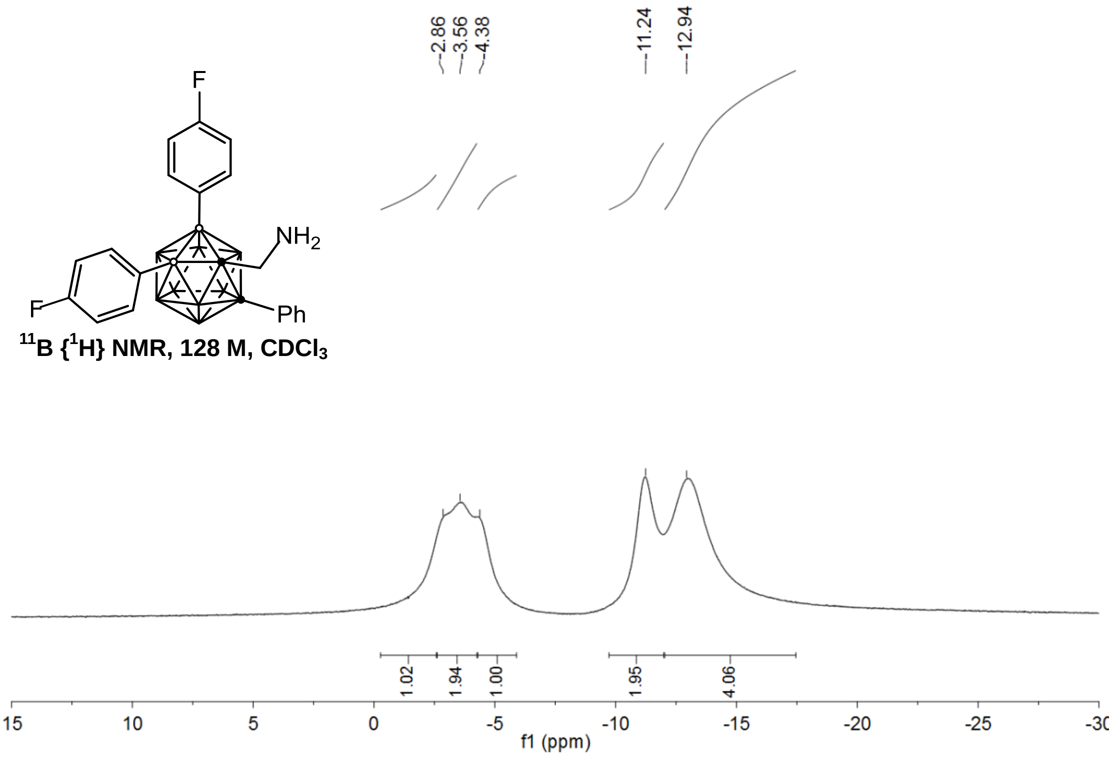

F
NH2
Ph
11B {1H} NMR, 128 M, CDCl3
-2.86
-3.56
-4.38
-11.24
-12.94
1.02
1.94
1.00
1.95
4.06
15
10
5
0
-5
-10
-15
-20
-25
-30
f1 (ppm)

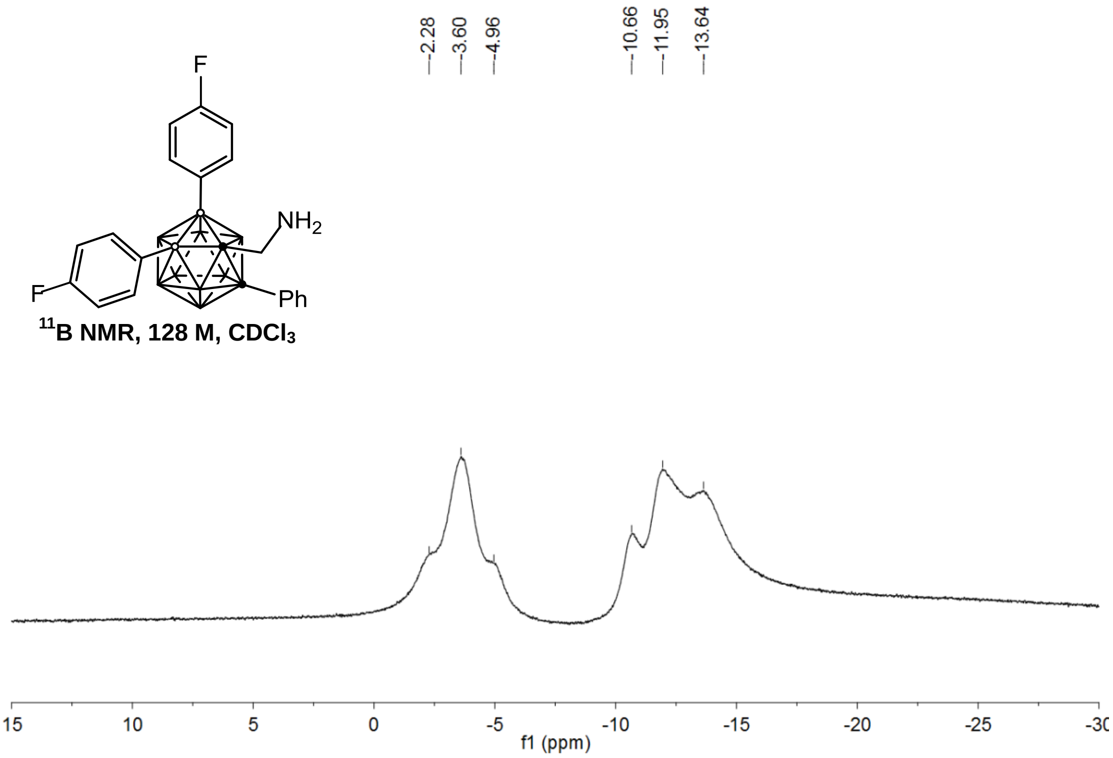

F
NH2
Ph
11B NMR, 128 M, CDCl3
-2.28
-3.60
-4.96
-10.66
-11.95
-13.64
15
10
5
0
-5
-10
-15
-20
-25
-30
f1 (ppm)

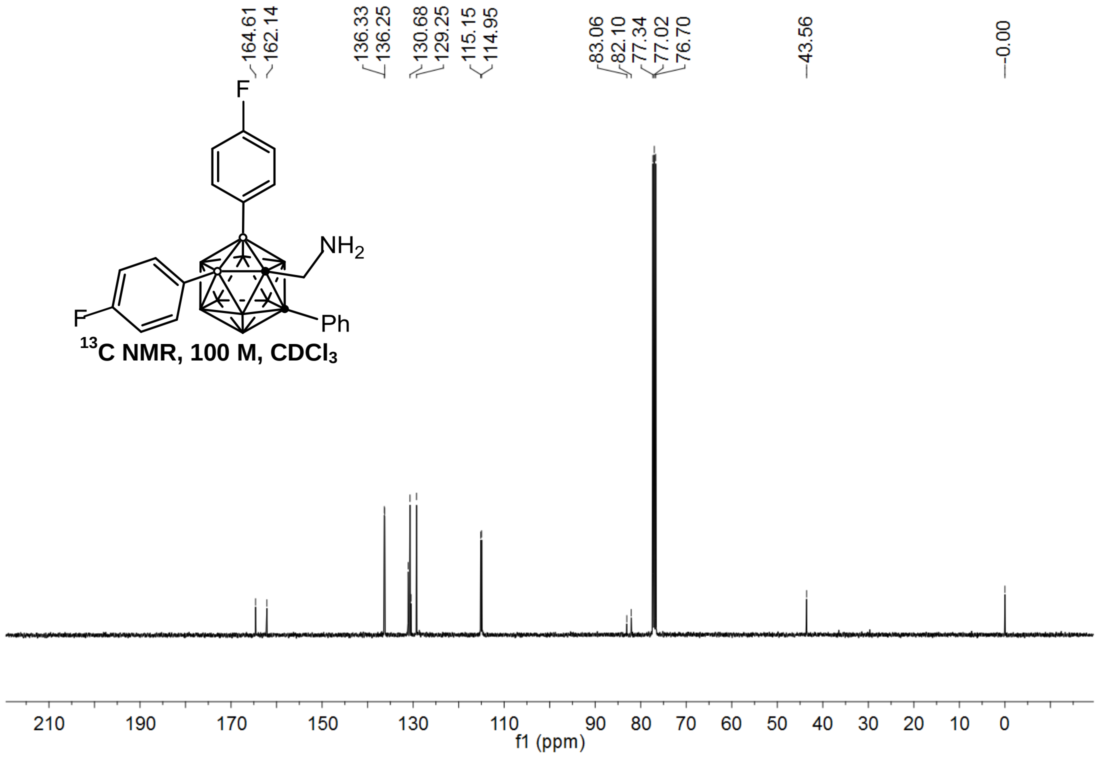

164.61
162.14
136.33
136.25
130.68
129.25
115.15
114.95
83.06
82.10
77.34
77.02
76.70
43.56
0.00
F
NH2
F
Ph
13C NMR, 100 M, CDCl3
210
190
170
150
130
110
90
80
70
60
50
40
30
20
10
0
f1 (ppm)

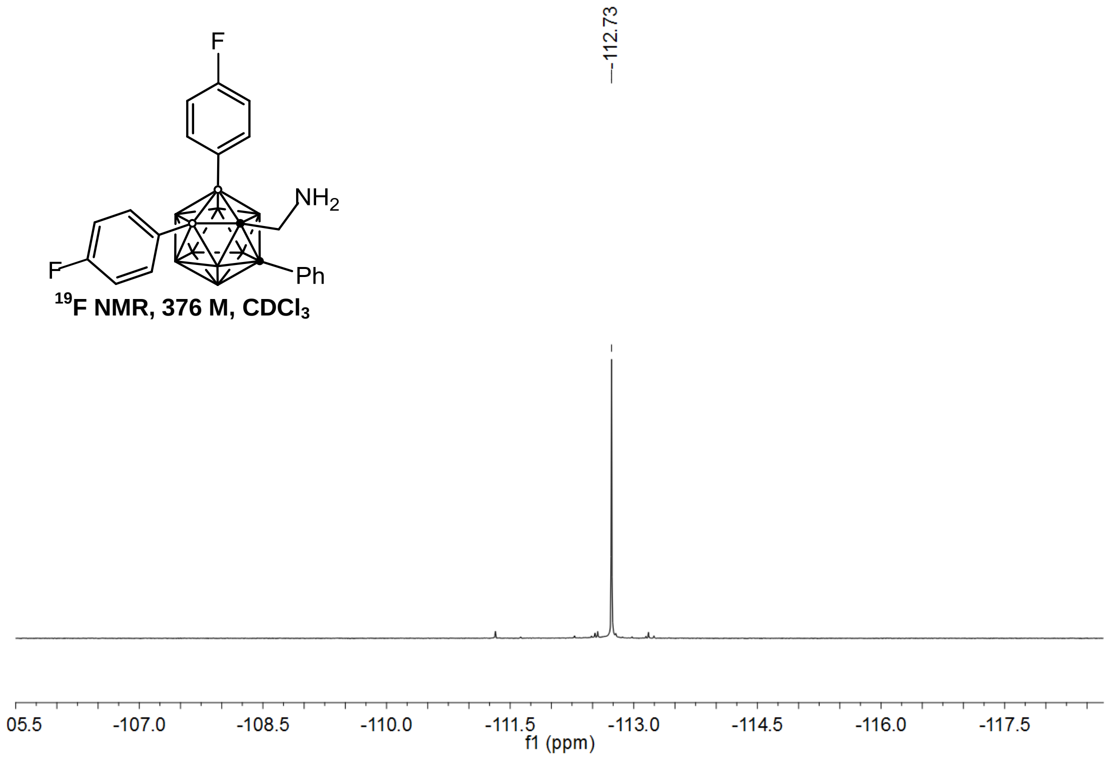

-112.73
F
NH2
F
Ph
19F NMR, 376 M, CDCl3
05.5
-107.0
-108.5
-110.0
-111.5
-113.0
-114.5
-116.0
-117.5
f1 (ppm)

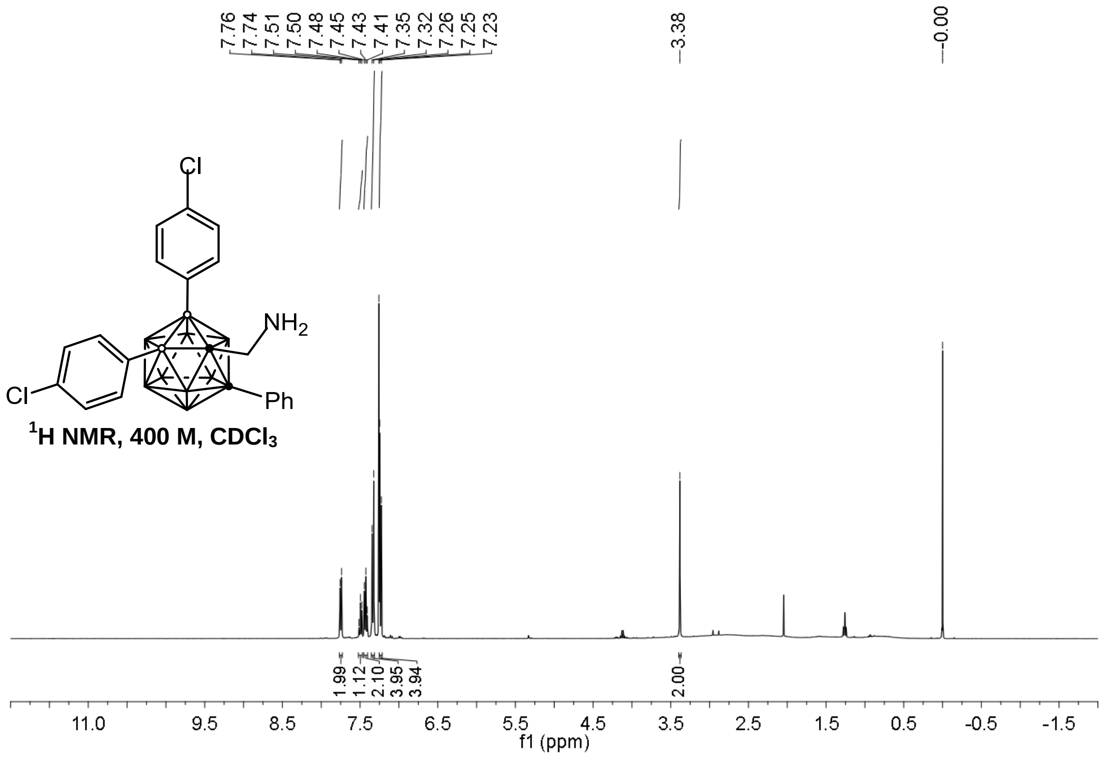

7.76
7.74
7.51
7.50
7.48
7.45
7.43
7.41
7.35
7.32
7.26
7.25
7.23
3.38
0.00
Cl
NH2
Cl
Ph
1H NMR, 400 M, CDCl3
1.99
1.12
2.10
3.95
3.94
2.00
11.0
9.5
8.5
7.5
6.5
5.5
4.5
3.5
2.5
1.5
0.5
-0.5
-1.5
f1 (ppm)

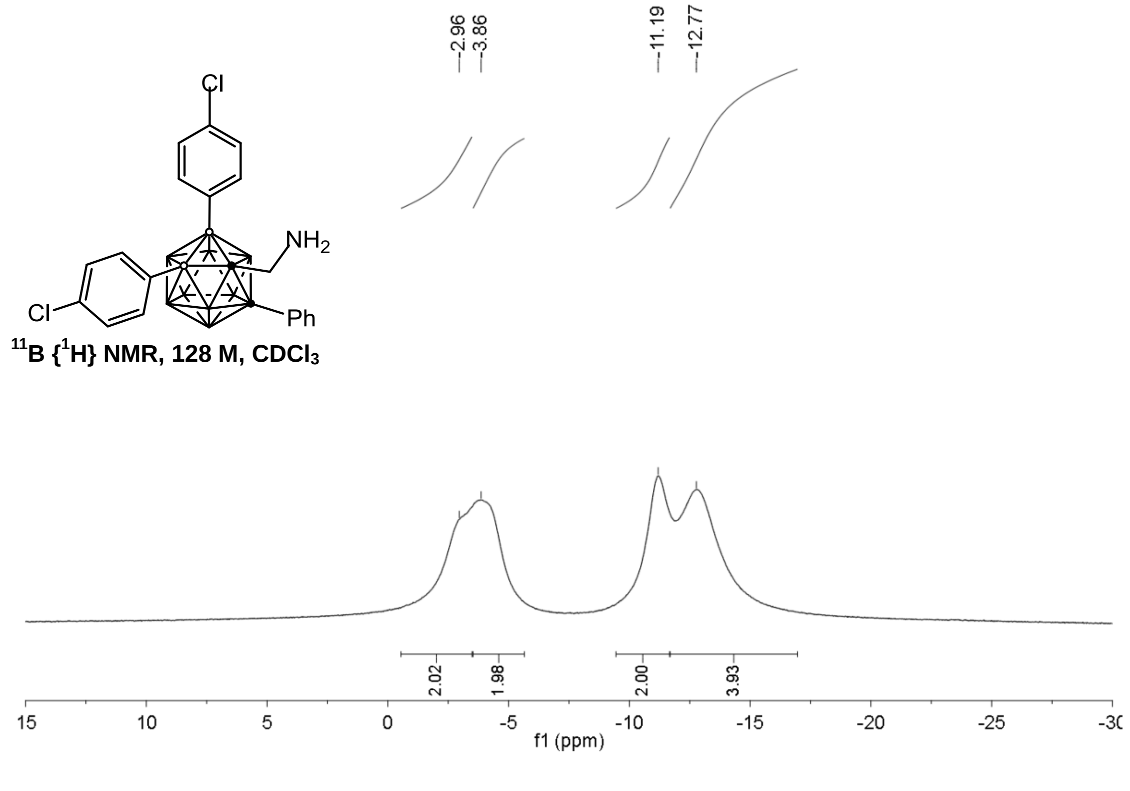

-2.96
-3.86
-11.19
-12.77
Cl
NH2
Cl
Ph
11B {1H} NMR, 128 M, CDCl3
2.02
1.98
2.00
3.93
15
10
5
0
-5
-10
-15
-20
-25
-30
f1 (ppm)

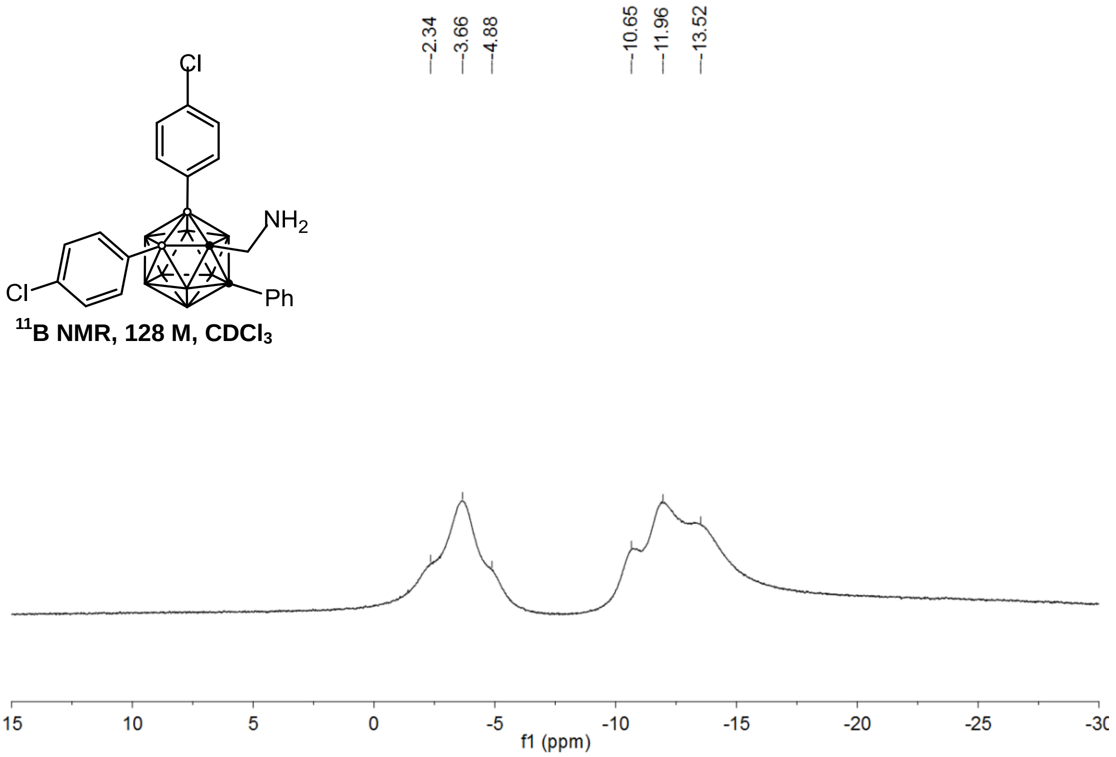
-2.34
-3.66
-4.88
-10.65
-11.96
-13.52
Cl
NH2
Cl
Ph
11B NMR, 128 M, CDCl3
15
10
5
0
-5
-10
-15
-20
-25
f1 (ppm)

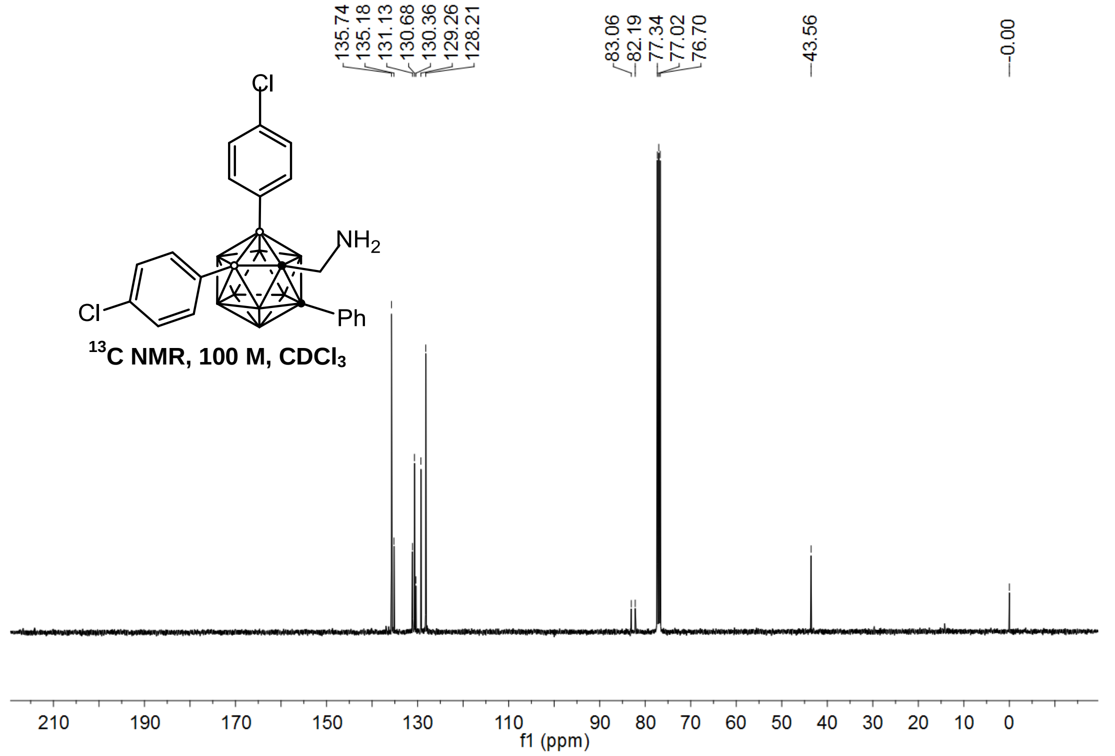
135.74
135.18
131.13
130.68
130.36
129.26
128.21
83.06
82.19
77.34
77.02
76.70
43.56
0.00
Cl
NH2
Cl
Ph
13C NMR, 100 M, CDCl3
210
190
170
150
130
110
90
80
70
60
50
40
30
20
10
0
f1 (ppm)

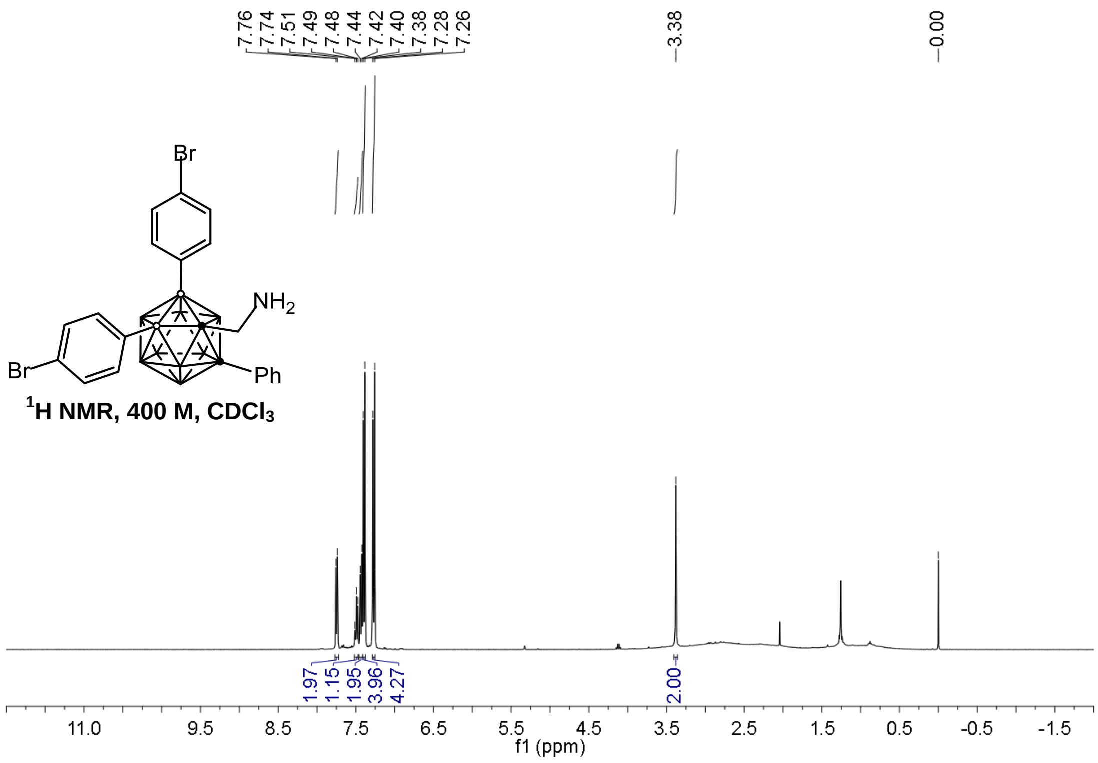
7.76
7.74
7.51
7.49
7.48
7.44
7.42
7.40
7.38
7.28
7.26
3.38
0.00
Br
NH2
Br
Ph
1H NMR, 400 M, CDCl3
1.97
1.15
1.95
3.96
4.27
2.00
11.0
9.5
8.5
7.5
6.5
5.5
4.5
3.5
2.5
1.5
0.5
-0.5
-1.5
f1 (ppm)

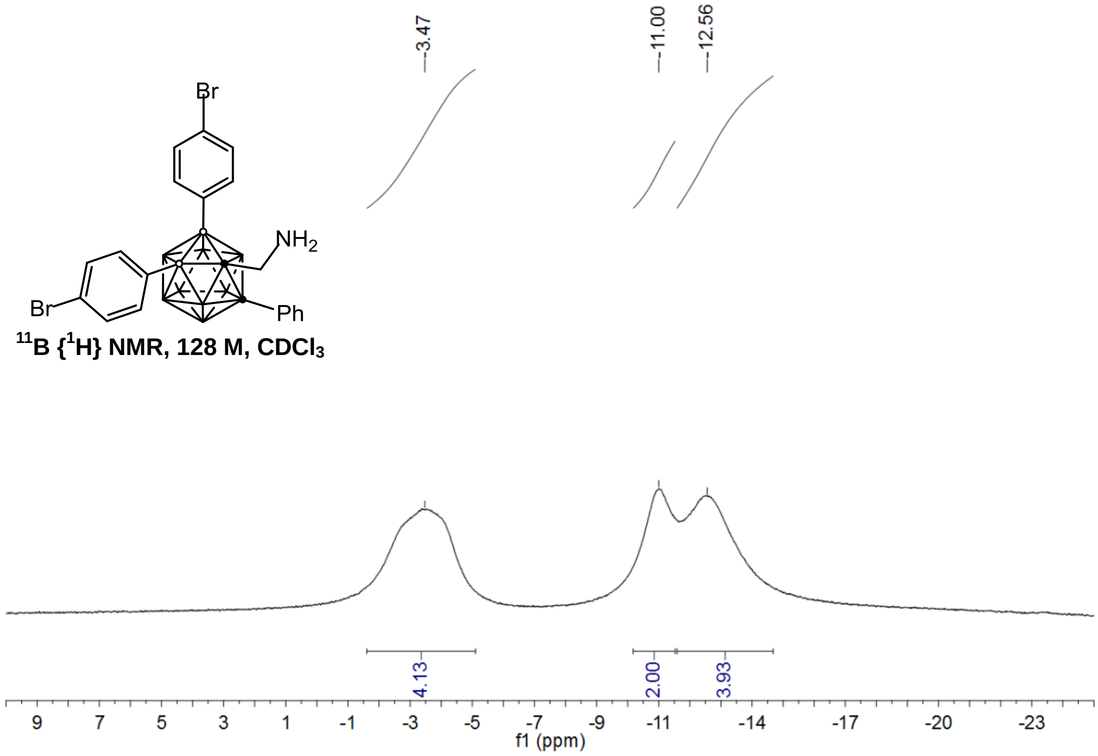
-3.47
-11.00
-12.56
Br
NH2
Br
Ph
11B {1H} NMR, 128 M, CDCl3
4.13
2.00
3.93
9
7
5
3
1
-1
-3
-5
-7
-9
-11
-14
-17
-20
-23
f1 (ppm)

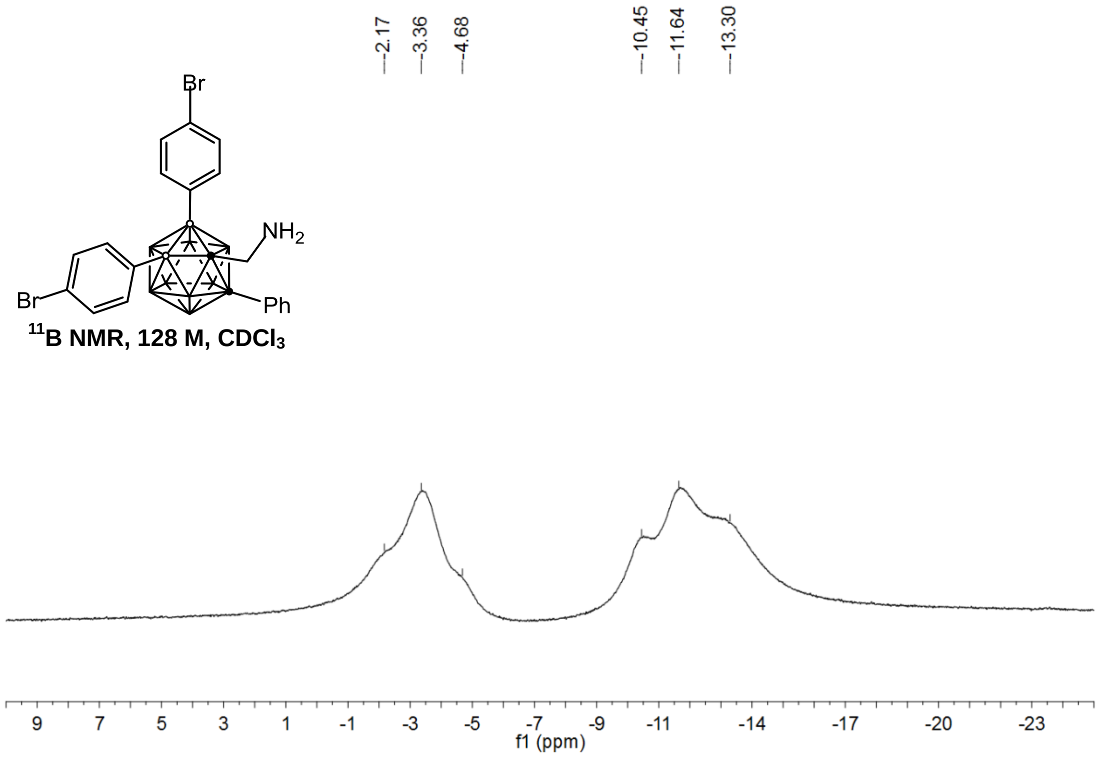

^{11}B NMR, 128 M, CDCl$_3$

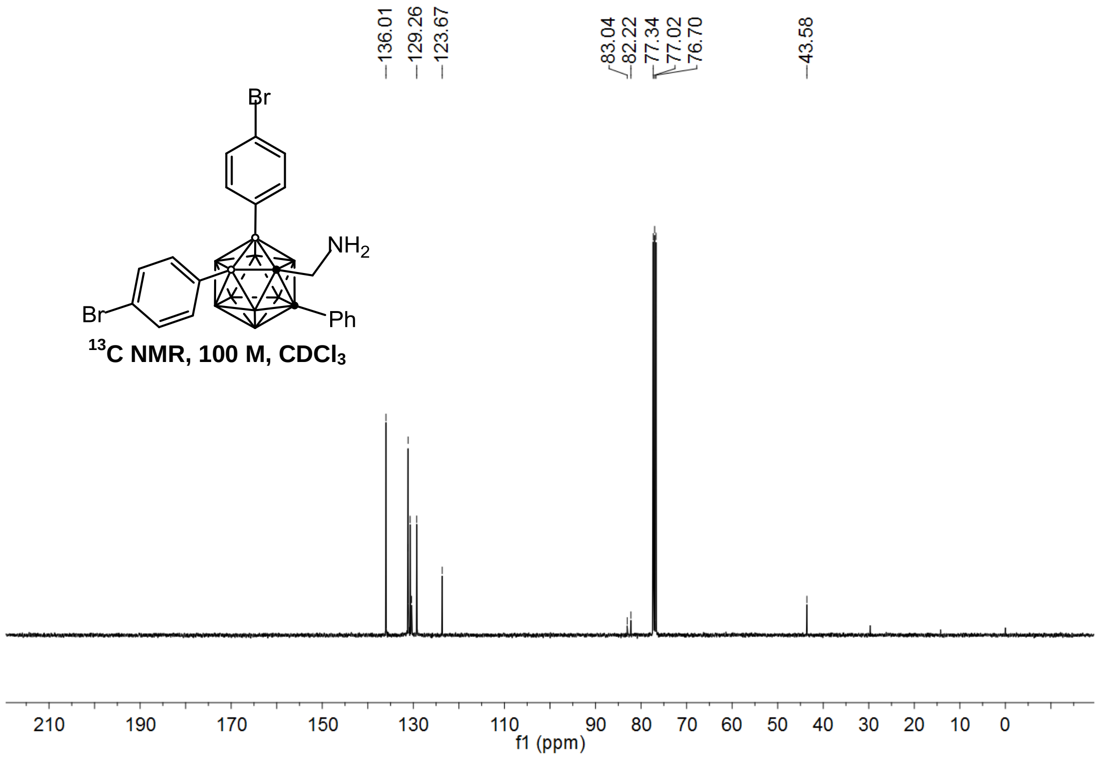

^{13}C NMR, 100 M, CDCl$_3$

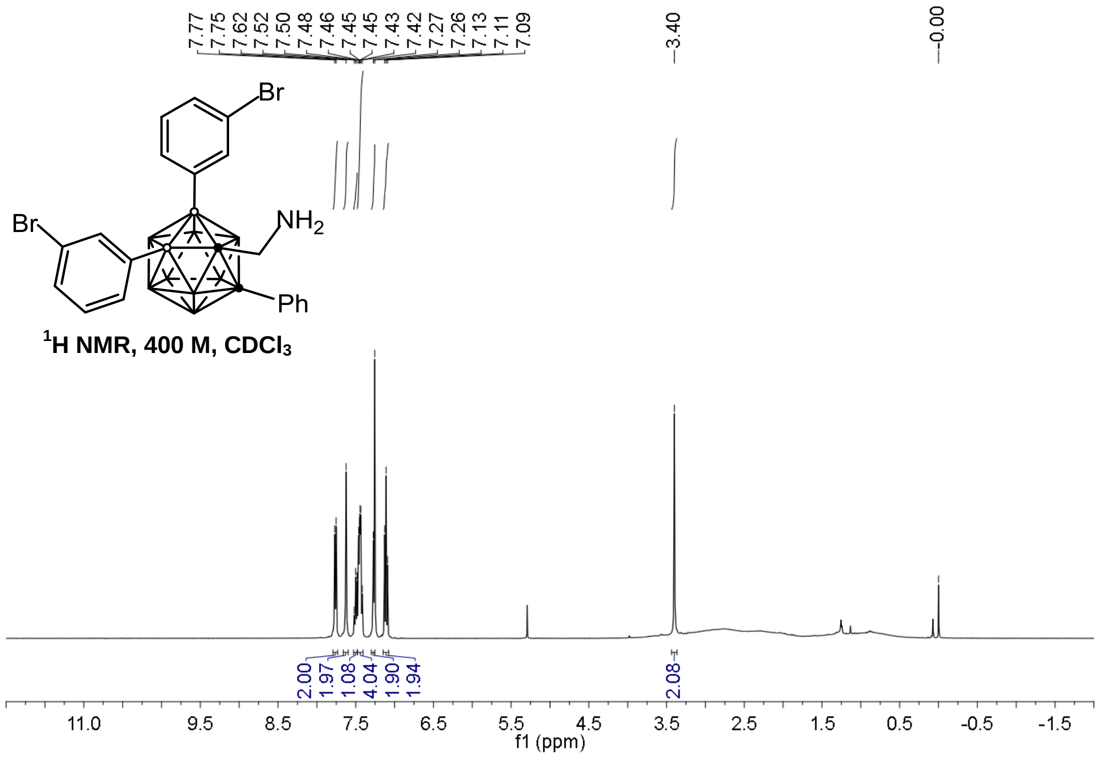

7.77
7.75
7.62
7.52
7.50
7.48
7.46
7.45
7.45
7.43
7.42
7.27
7.26
7.13
7.11
7.09
3.40
0.00
Br
Br
NH2
Ph
1H NMR, 400 M, CDCl3
2.00
1.97
1.08
4.04
1.90
1.94
2.08
11.0
9.5
8.5
7.5
6.5
5.5
4.5
3.5
2.5
1.5
0.5
-0.5
-1.5
f1 (ppm)

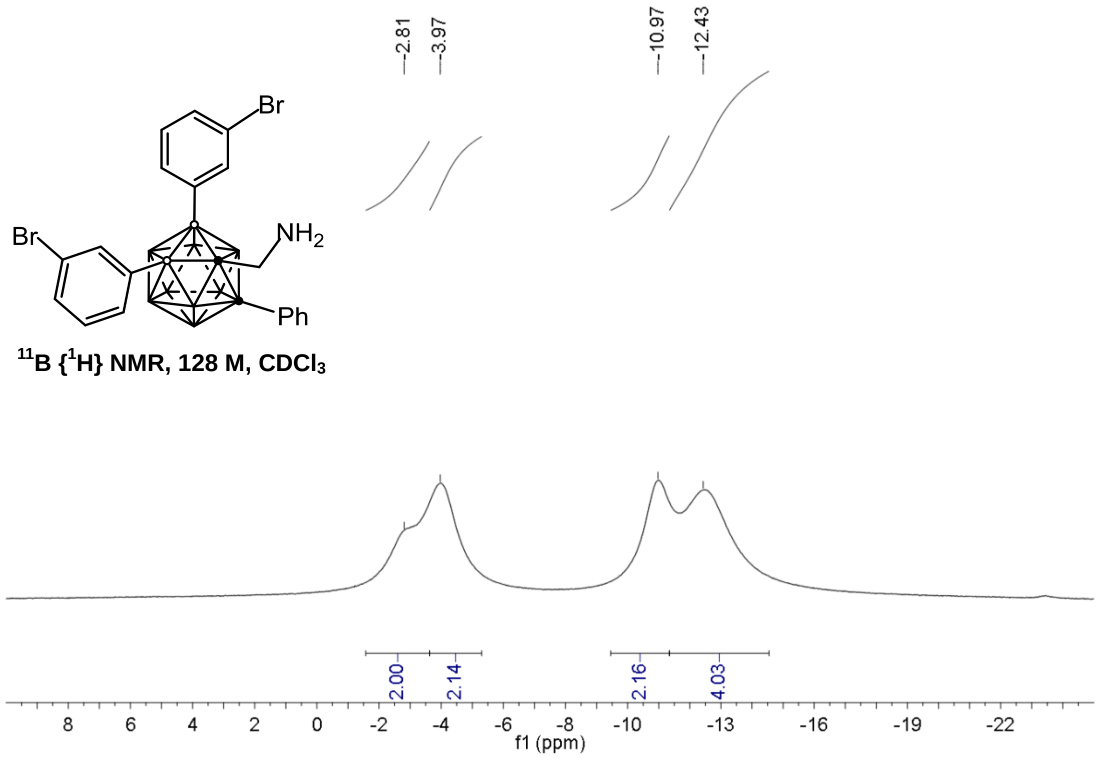

-2.81
-3.97
-10.97
-12.43
Br
Br
NH2
Ph
11B {1H} NMR, 128 M, CDCl3
2.00
2.14
2.16
4.03
8
6
4
2
0
-2
-4
-6
-8
-10
-13
-16
-19
-22
f1 (ppm)

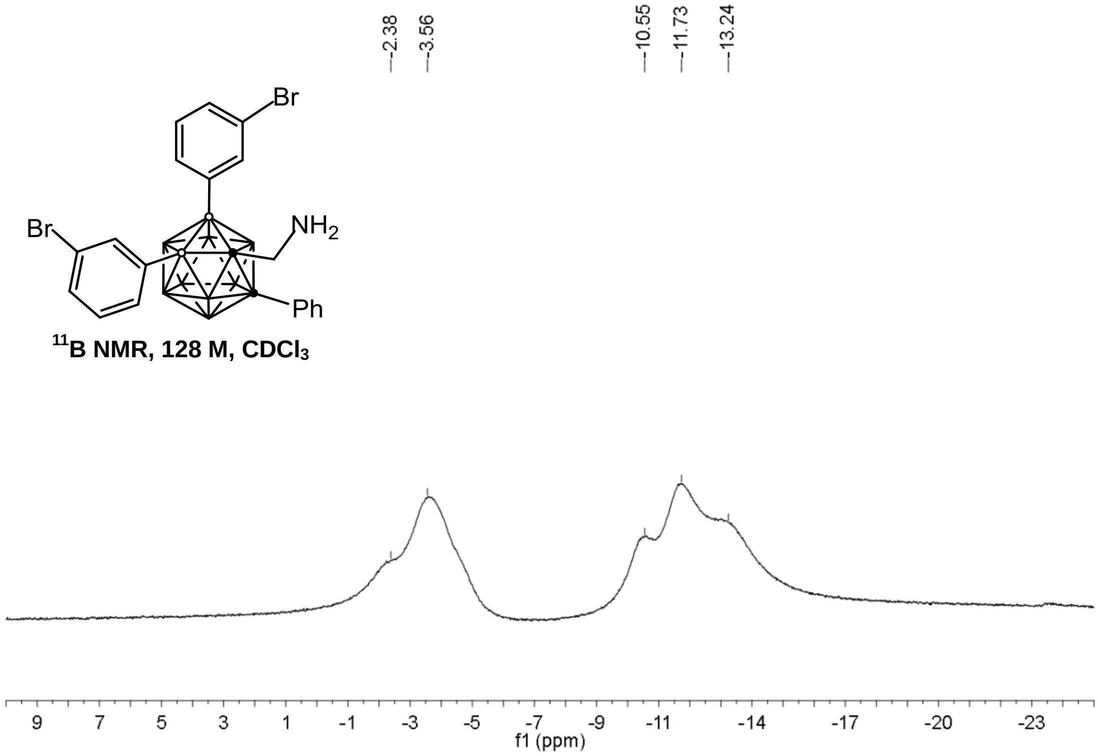

-2.38
-3.56
-10.55
-11.73
-13.24
Br
Br
NH2
Ph
11B NMR, 128 M, CDCl3
9 7 5 3 1 -1 -3 -5 -7 -9 -11 -14 -17 -20 -23
f1 (ppm)

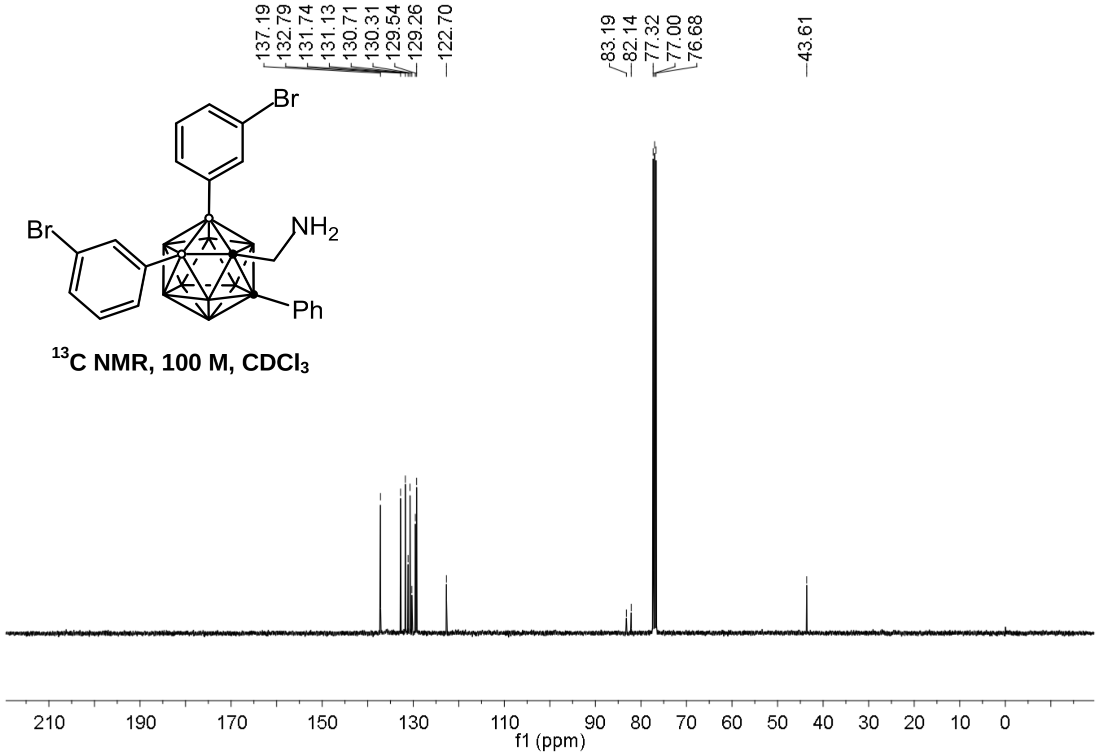

137.19
132.79
131.74
131.13
130.71
130.31
129.54
129.26
122.70
83.19
82.14
77.32
77.00
76.68
43.61
Br
Br
NH2
Ph
13C NMR, 100 M, CDCl3
210 190 170 150 130 110 90 80 70 60 50 40 30 20 10 0
f1 (ppm)

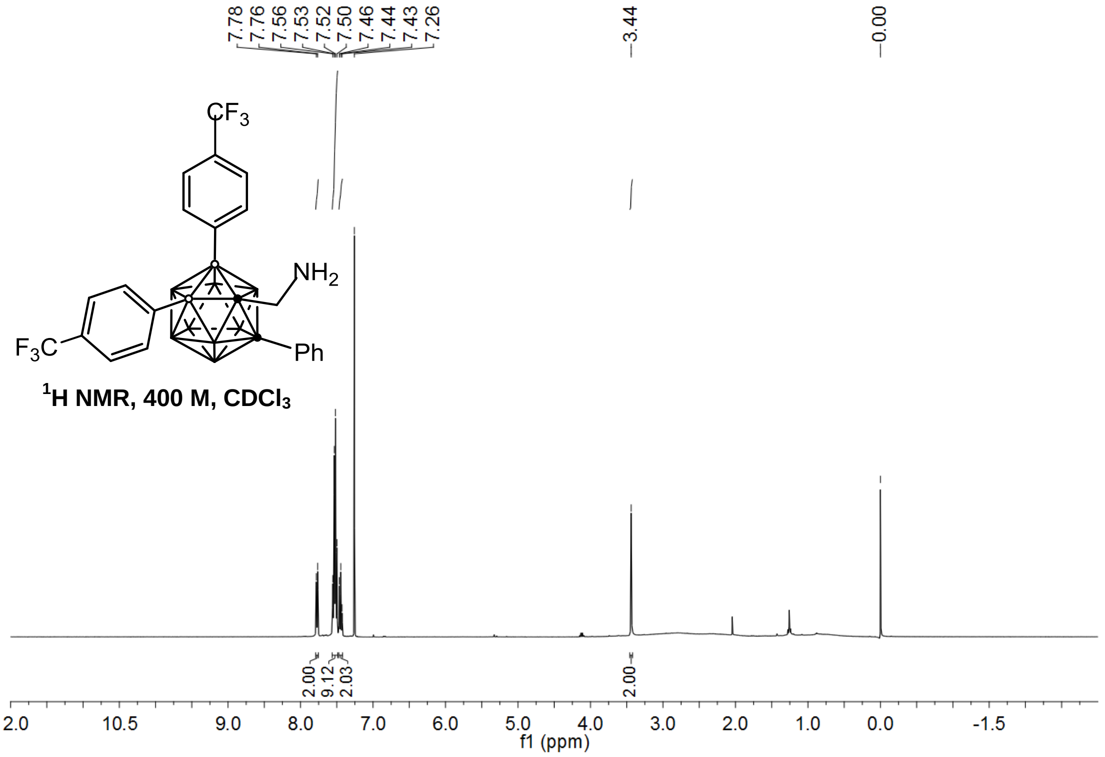
7.78
7.76
7.56
7.53
7.52
7.50
7.46
7.44
7.43
7.26
3.44
0.00
CF3
NH2
F3C
Ph
1H NMR, 400 M, CDCl3
2.00
9.12
2.03
2.00
f1 (ppm)

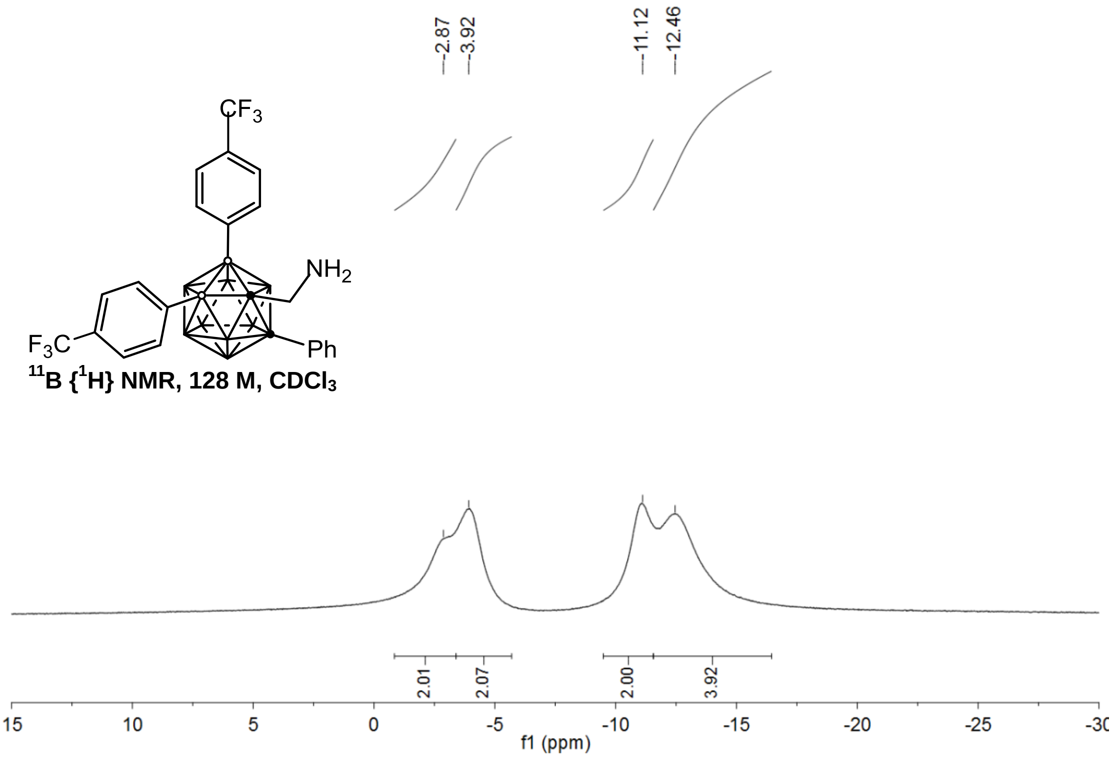
-2.87
-3.92
-11.12
-12.46
CF3
NH2
F3C
Ph
11B {1H} NMR, 128 M, CDCl3
2.01
2.07
2.00
3.92
f1 (ppm)

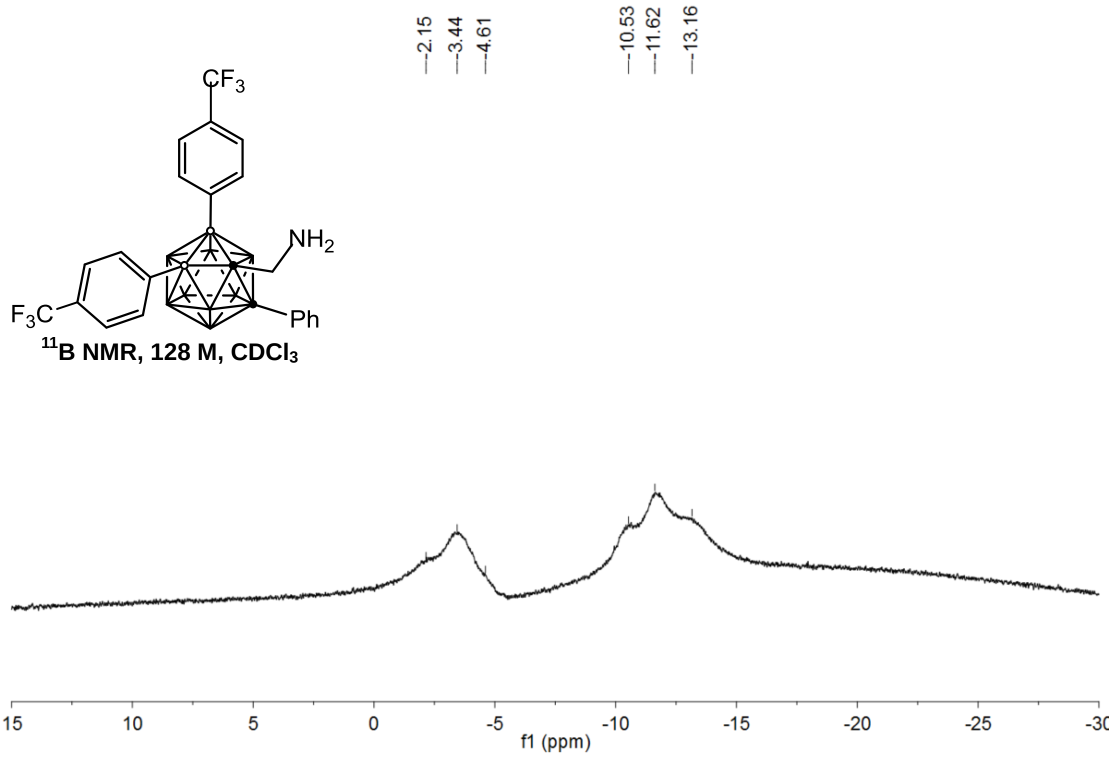

CF3
NH2
F3C
Ph
11B NMR, 128 M, CDCl3
-2.15
-3.44
-4.61
-10.53
-11.62
-13.16
15
10
5
0
-5
-10
-15
-20
-25
f1 (ppm)

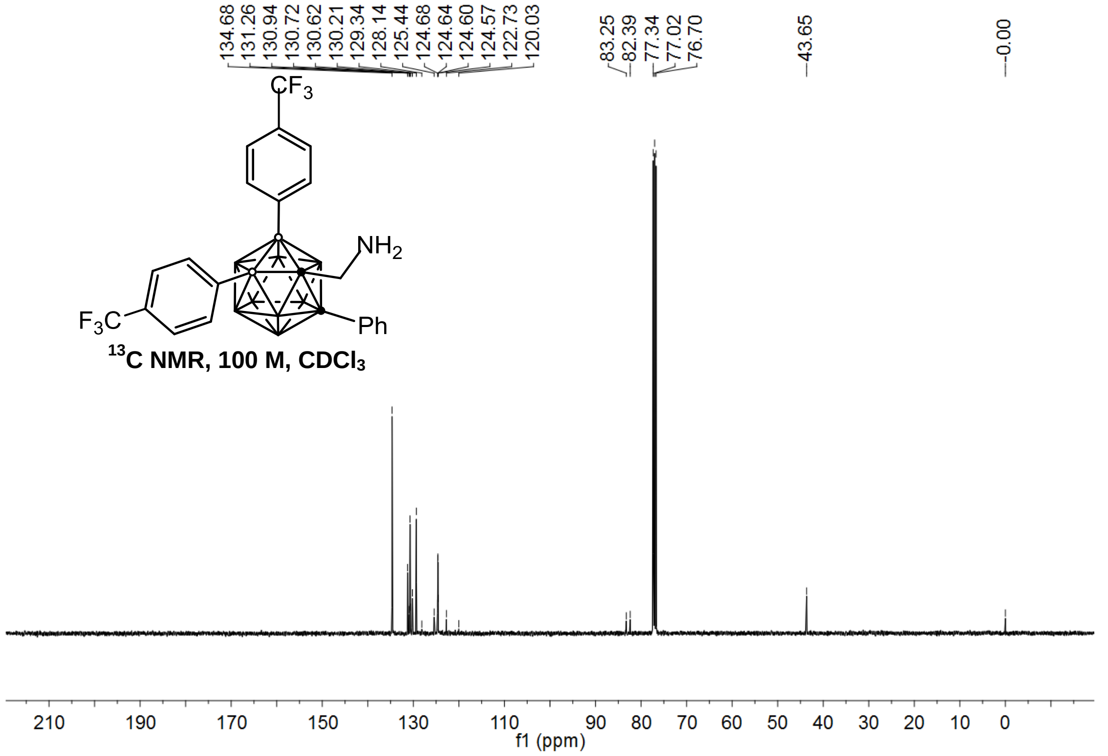

134.68
131.26
130.94
130.72
130.62
130.21
129.34
128.14
125.44
124.68
124.64
124.60
124.57
122.73
120.03
83.25
82.39
77.34
77.02
76.70
43.65
0.00
CF3
NH2
F3C
Ph
13C NMR, 100 M, CDCl3
210
190
170
150
130
110
90
80
70
60
50
40
30
20
10
0
f1 (ppm)

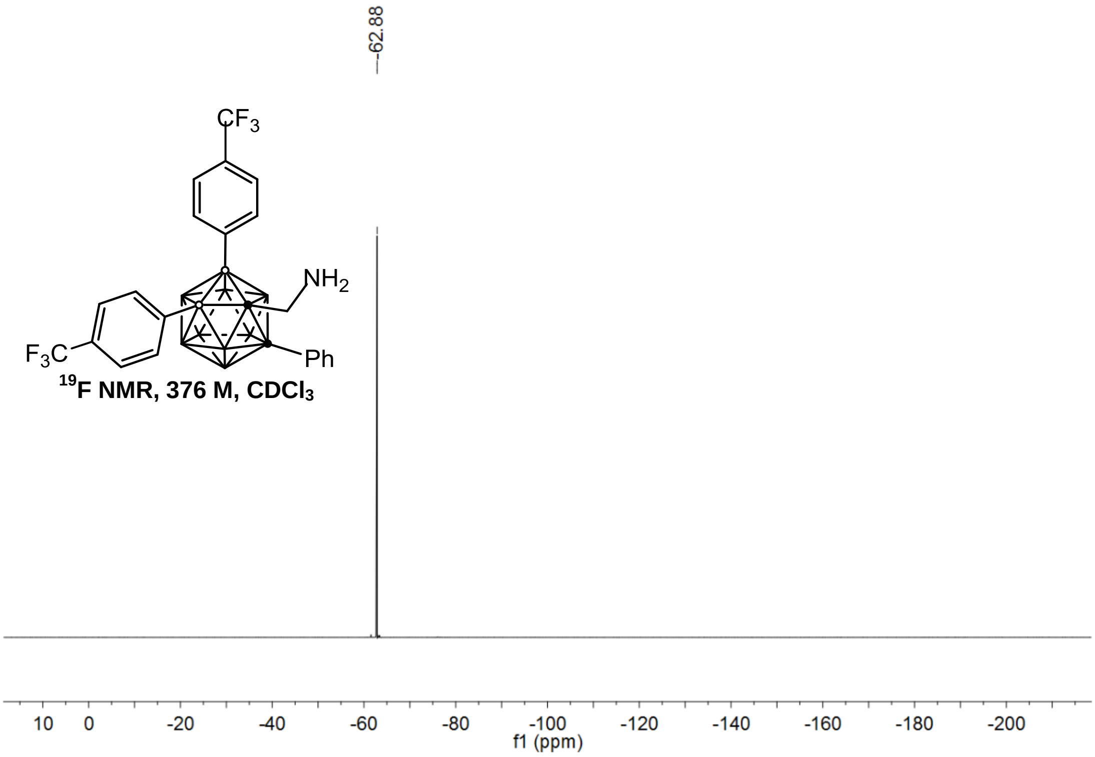

^{19}F NMR, 376 M, $CDCl_3$

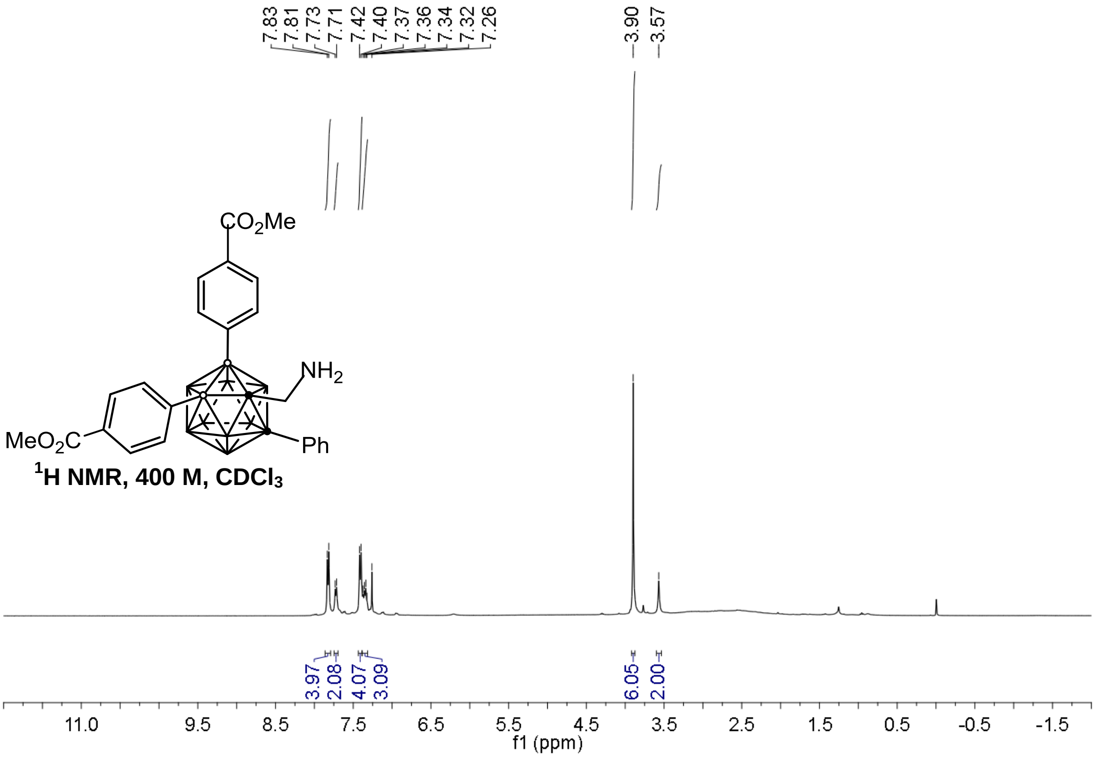

^{1}H NMR, 400 M, $CDCl_3$

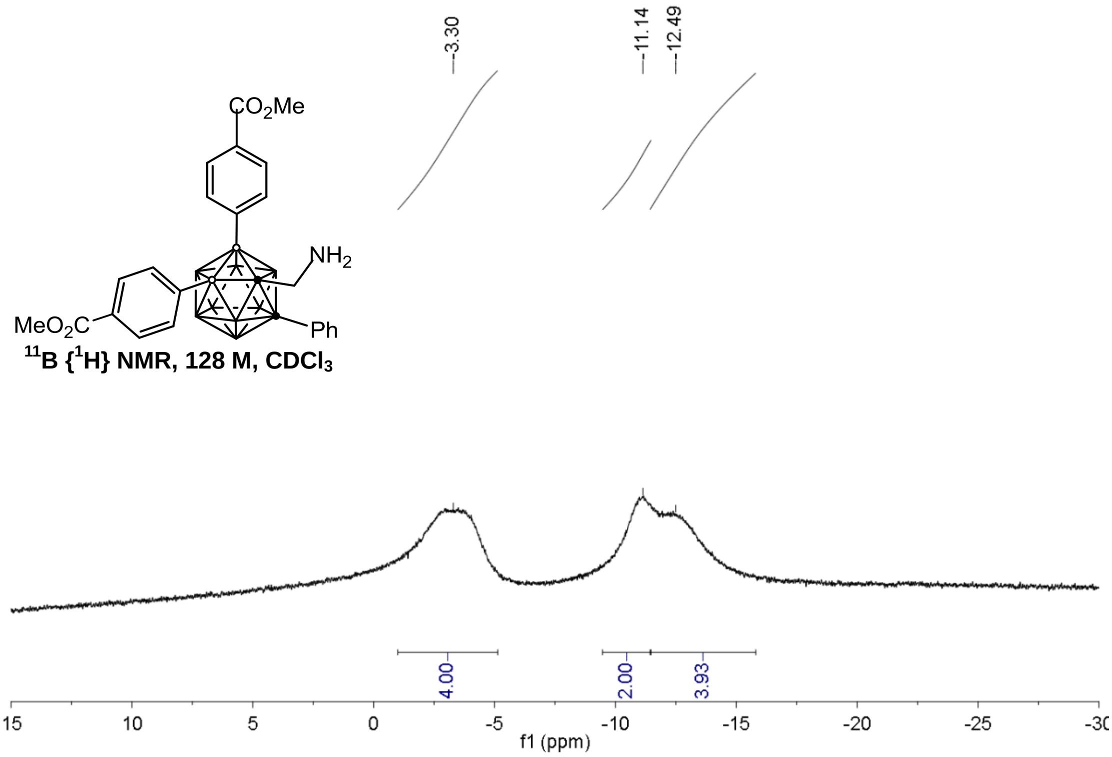

-3.30
-11.14
-12.49
CO_2Me
NH_2
MeO_2C
Ph
$^{11}B\{^1H\}$ NMR, 128 M, $CDCl_3$
4.00
2.00
3.93
15
10
5
0
-5
-10
-15
-20
-25
-30
f1 (ppm)

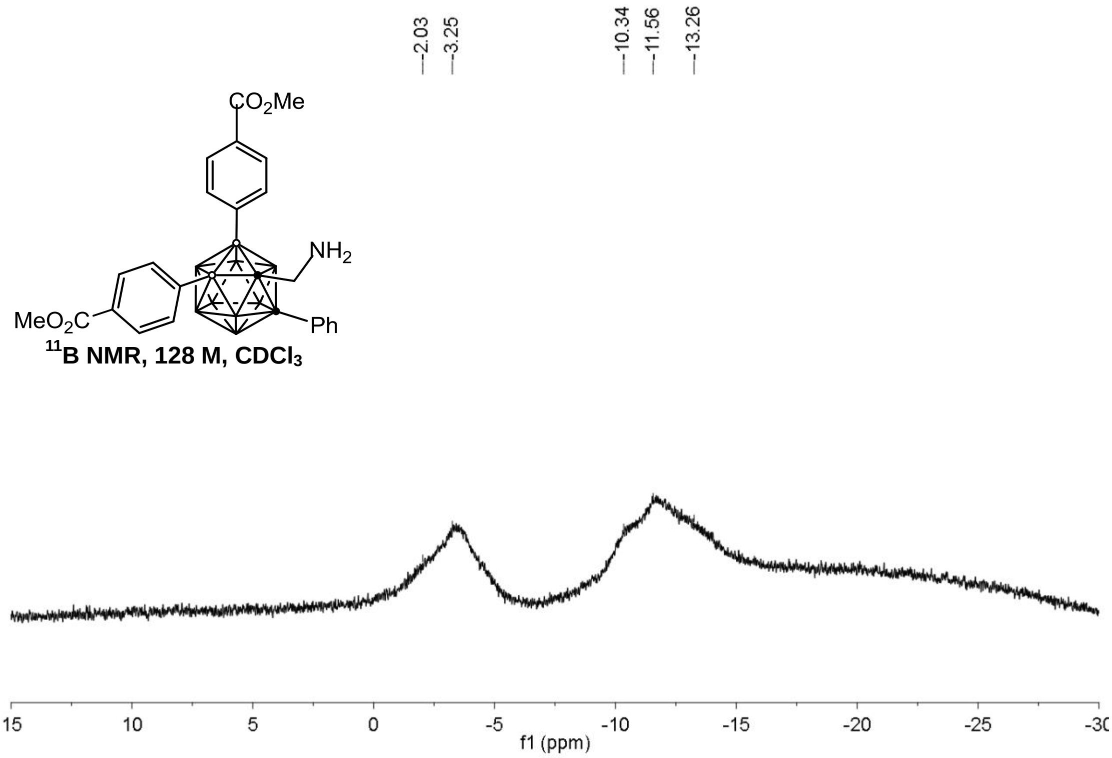

-2.03
-3.25
-10.34
-11.56
-13.26
CO_2Me
NH_2
MeO_2C
Ph
^{11}B NMR, 128 M, $CDCl_3$
15
10
5
0
-5
-10
-15
-20
-25
-30
f1 (ppm)

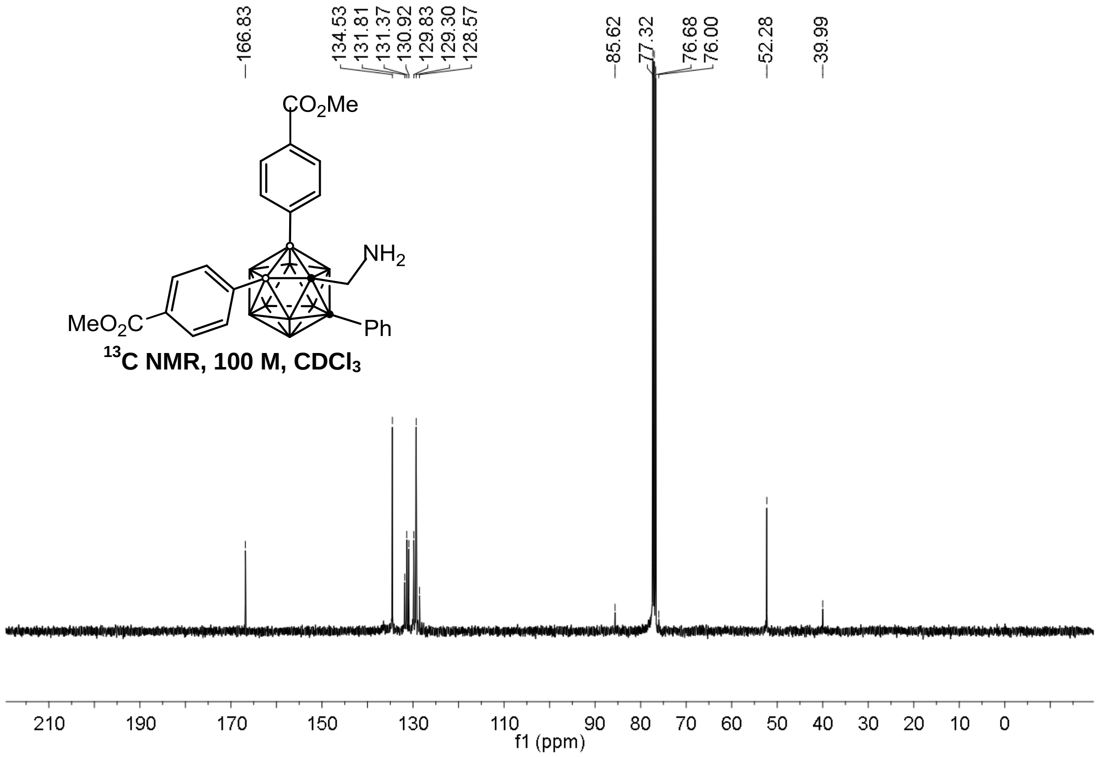
166.83
134.53
131.81
131.37
130.92
129.83
129.30
128.57
85.62
77.32
76.68
76.00
52.28
39.99
CO2Me
NH2
MeO2C
Ph
13C NMR, 100 M, CDCl3
210 190 170 150 130 110 90 80 70 60 50 40 30 20 10 0
f1 (ppm)

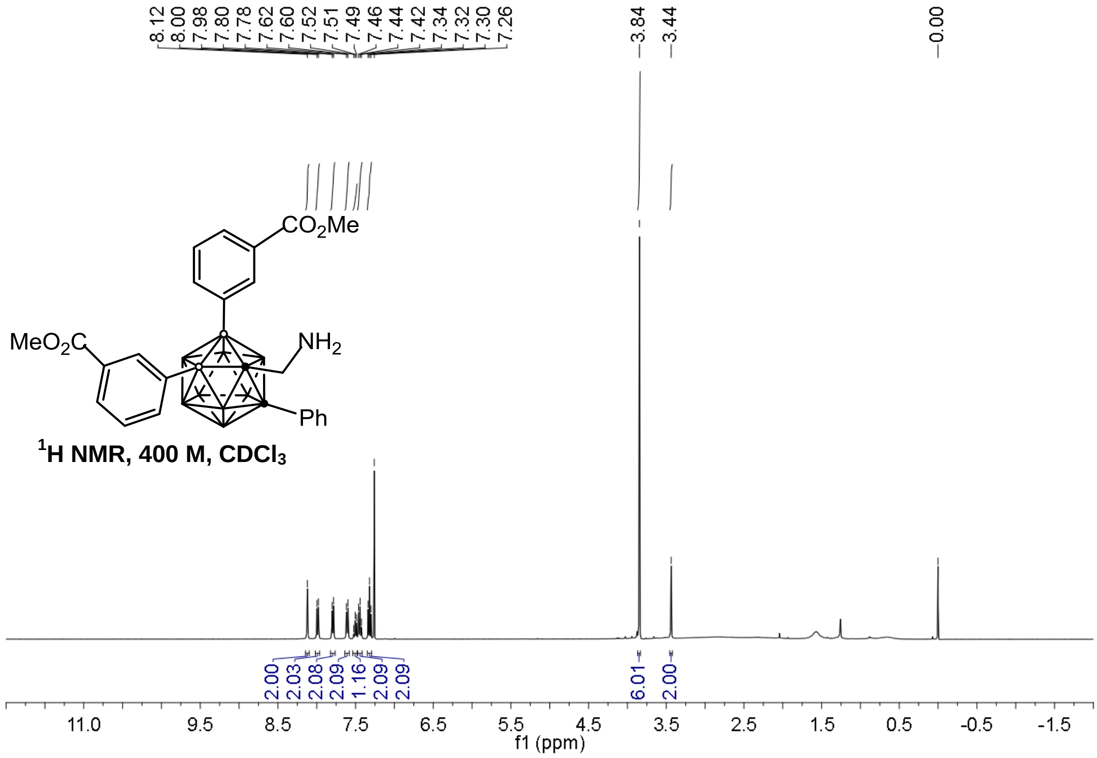
8.12
8.00
7.98
7.80
7.78
7.62
7.60
7.52
7.51
7.49
7.46
7.44
7.42
7.34
7.32
7.30
7.26
3.84
3.44
0.00
CO2Me
MeO2C
NH2
Ph
1H NMR, 400 M, CDCl3
2.00
2.03
2.08
2.09
1.16
2.09
2.09
6.01
2.00
11.0 9.5 8.5 7.5 6.5 5.5 4.5 3.5 2.5 1.5 0.5 -0.5 -1.5
f1 (ppm)

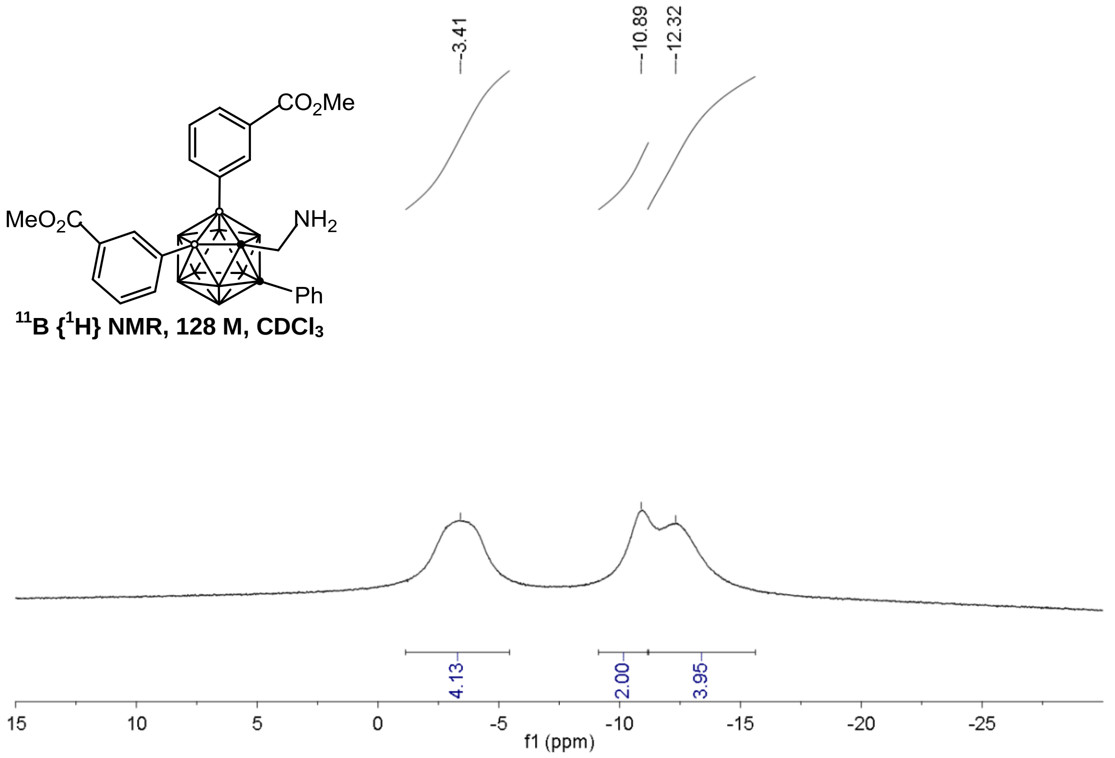
-3.41
-10.89
-12.32
CO2Me
MeO2C
NH2
Ph
11B {1H} NMR, 128 M, CDCl3
4.13
2.00
3.95
15
10
5
0
-5
-10
-15
-20
-25
f1 (ppm)

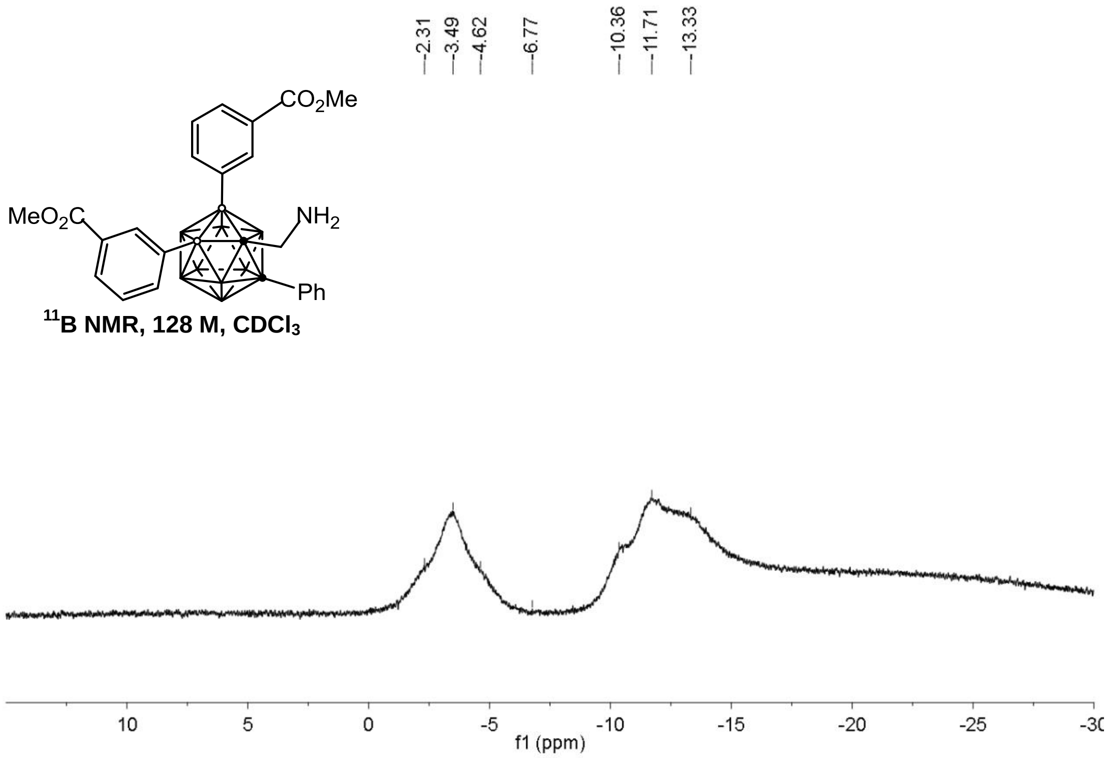
-2.31
-3.49
-4.62
-6.77
-10.36
-11.71
-13.33
CO2Me
MeO2C
NH2
Ph
11B NMR, 128 M, CDCl3
10
5
0
-5
-10
-15
-20
-25
f1 (ppm)

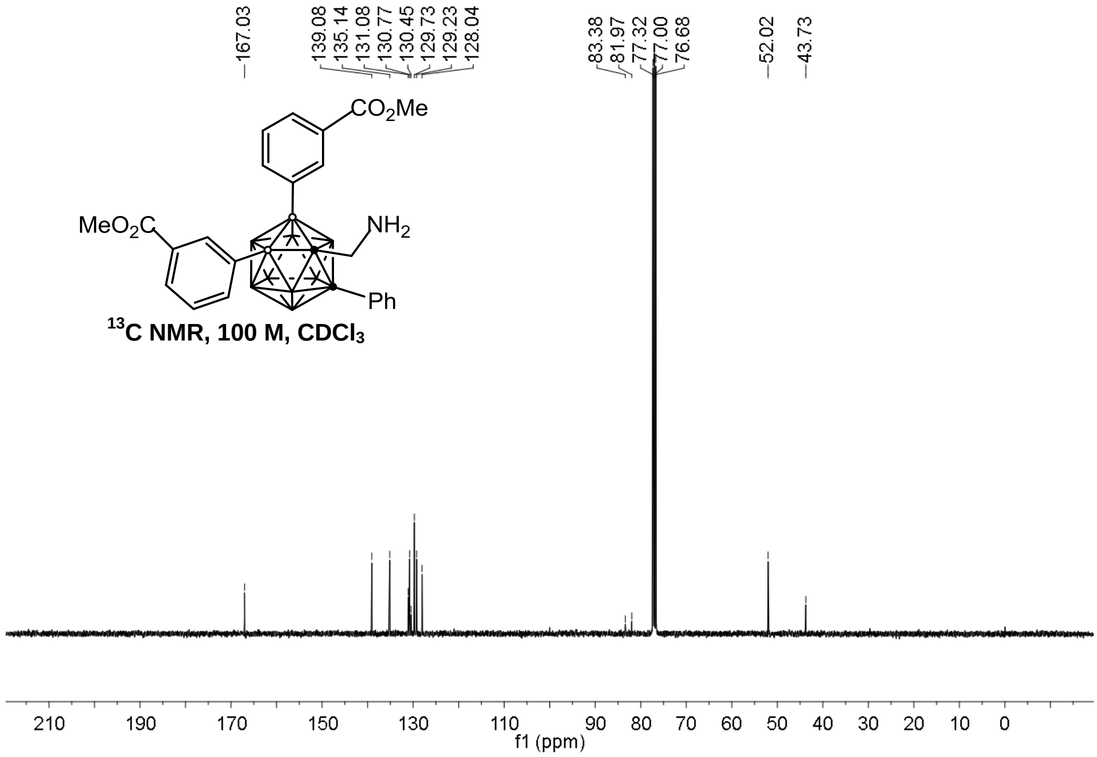

167.03
139.08
135.14
131.08
130.77
130.45
129.73
129.23
128.04
83.38
81.97
77.32
77.00
76.68
52.02
43.73
CO2Me
MeO2C
NH2
Ph
13C NMR, 100 M, CDCl3
210
190
170
150
130
110
90
80
70
60
50
40
30
20
10
0
f1 (ppm)

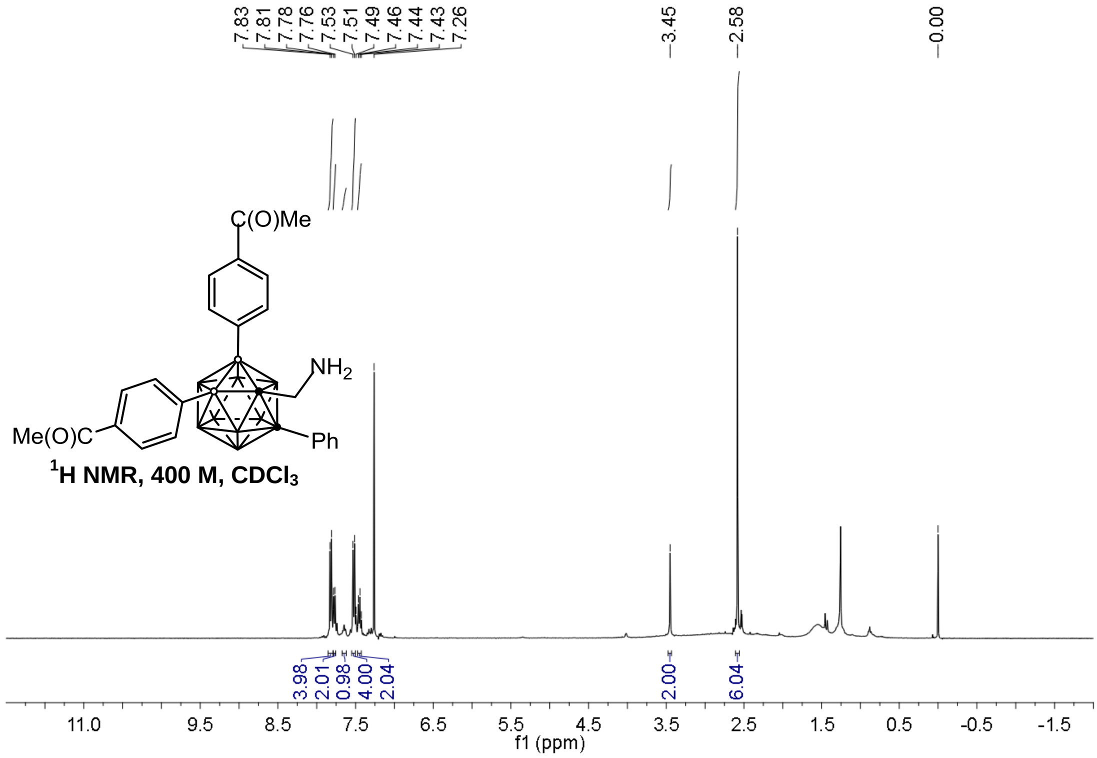

7.83
7.81
7.78
7.76
7.53
7.51
7.49
7.46
7.44
7.43
7.26
3.45
2.58
0.00
C(O)Me
Me(O)C
NH2
Ph
1H NMR, 400 M, CDCl3
3.98
2.01
0.98
4.00
2.04
2.00
6.04
11.0
9.5
8.5
7.5
6.5
5.5
4.5
3.5
2.5
1.5
0.5
-0.5
-1.5
f1 (ppm)

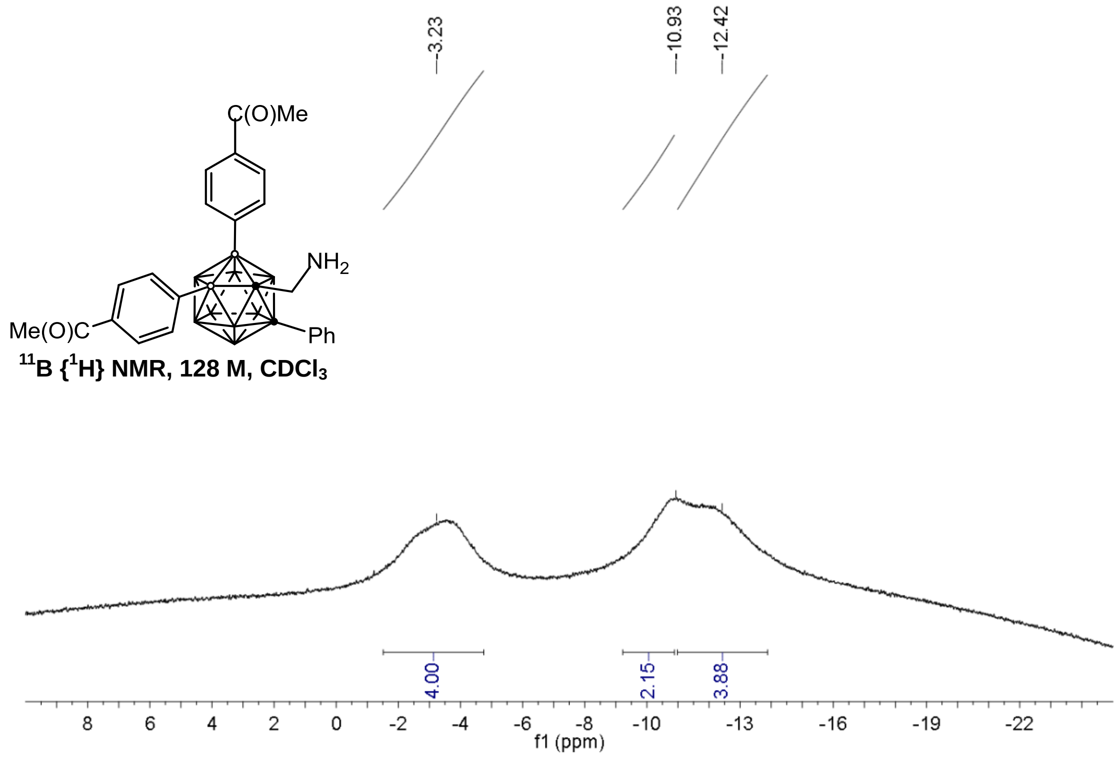

^{11}B {^{1}H} NMR, 128 M, CDCl$_3$

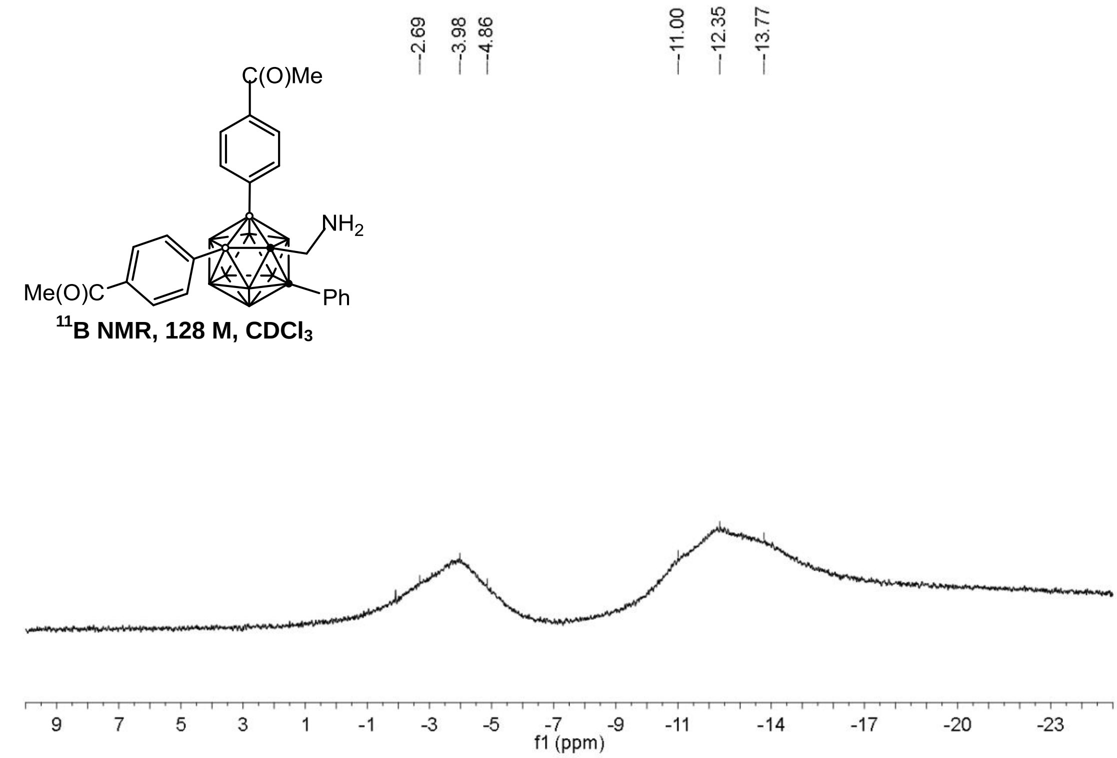

^{11}B NMR, 128 M, CDCl$_3$

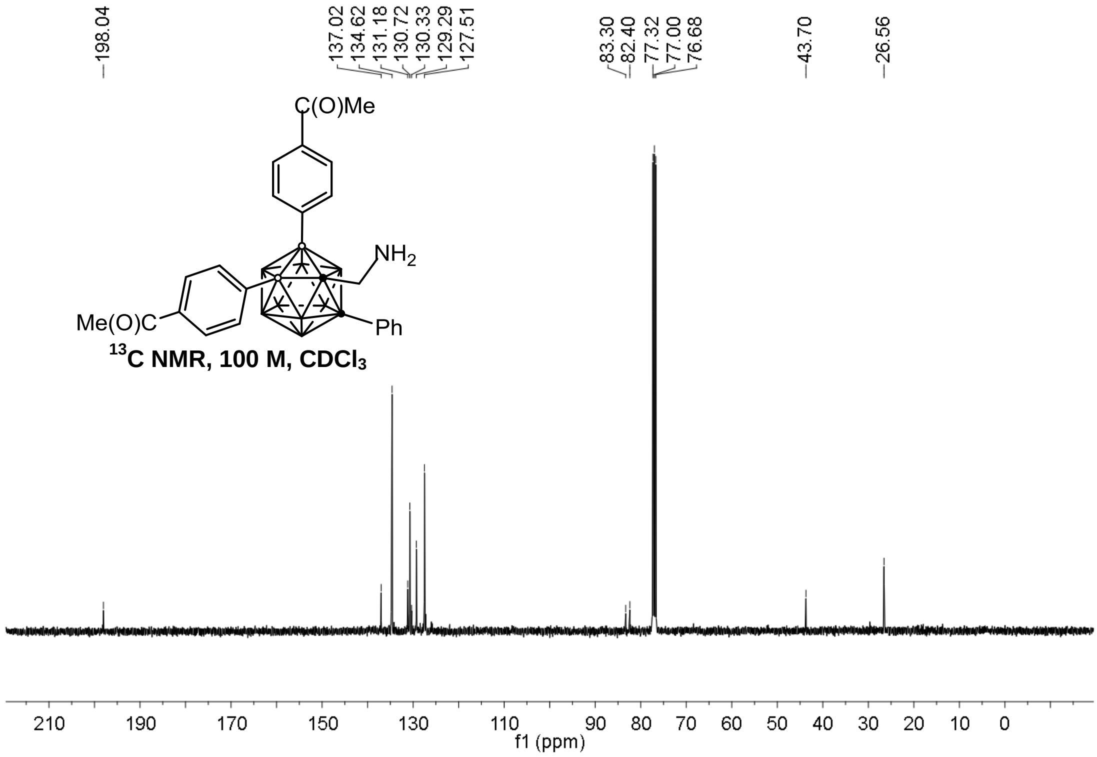
198.04
137.02
134.62
131.18
130.72
130.33
129.29
127.51
83.30
82.40
77.32
77.00
76.68
43.70
26.56
C(O)Me
NH2
Me(O)C
Ph
13C NMR, 100 M, CDCl3
210 190 170 150 130 110 90 80 70 60 50 40 30 20 10 0
f1 (ppm)

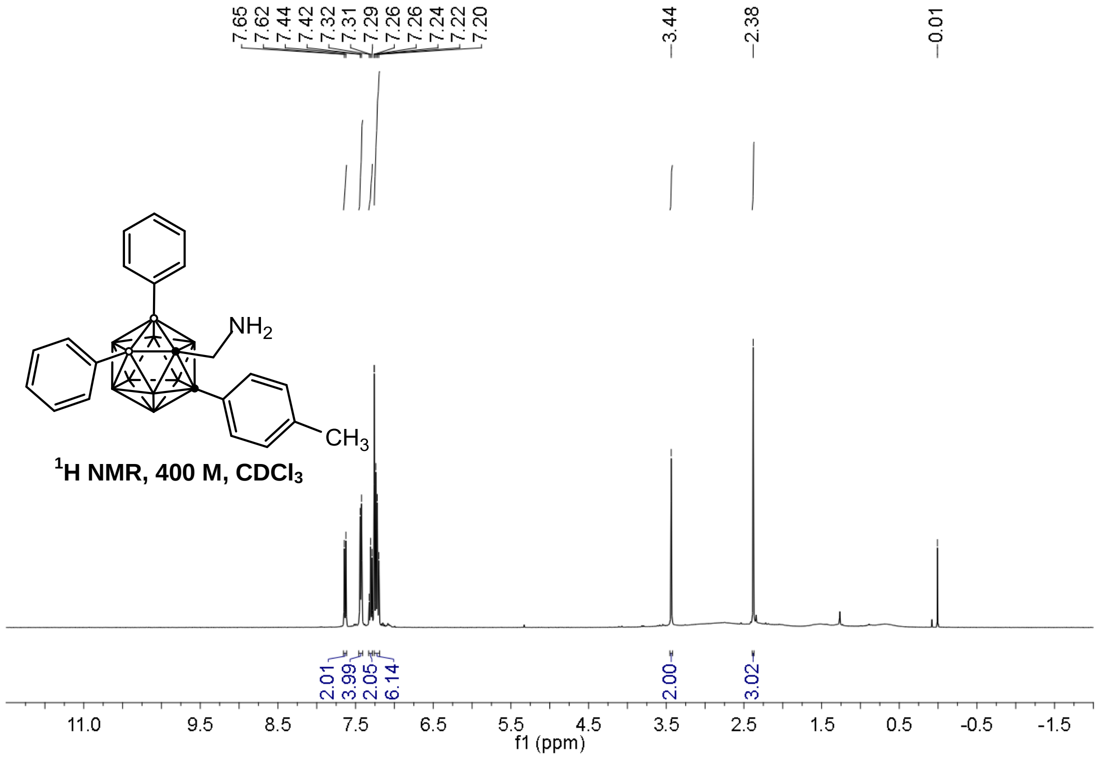
7.65
7.62
7.44
7.42
7.32
7.31
7.29
7.26
7.26
7.24
7.22
7.20
3.44
2.38
0.01
NH2
CH3
1H NMR, 400 M, CDCl3
2.01
3.99
2.05
6.14
2.00
3.02
11.0 9.5 8.5 7.5 6.5 5.5 4.5 3.5 2.5 1.5 0.5 -0.5 -1.5
f1 (ppm)

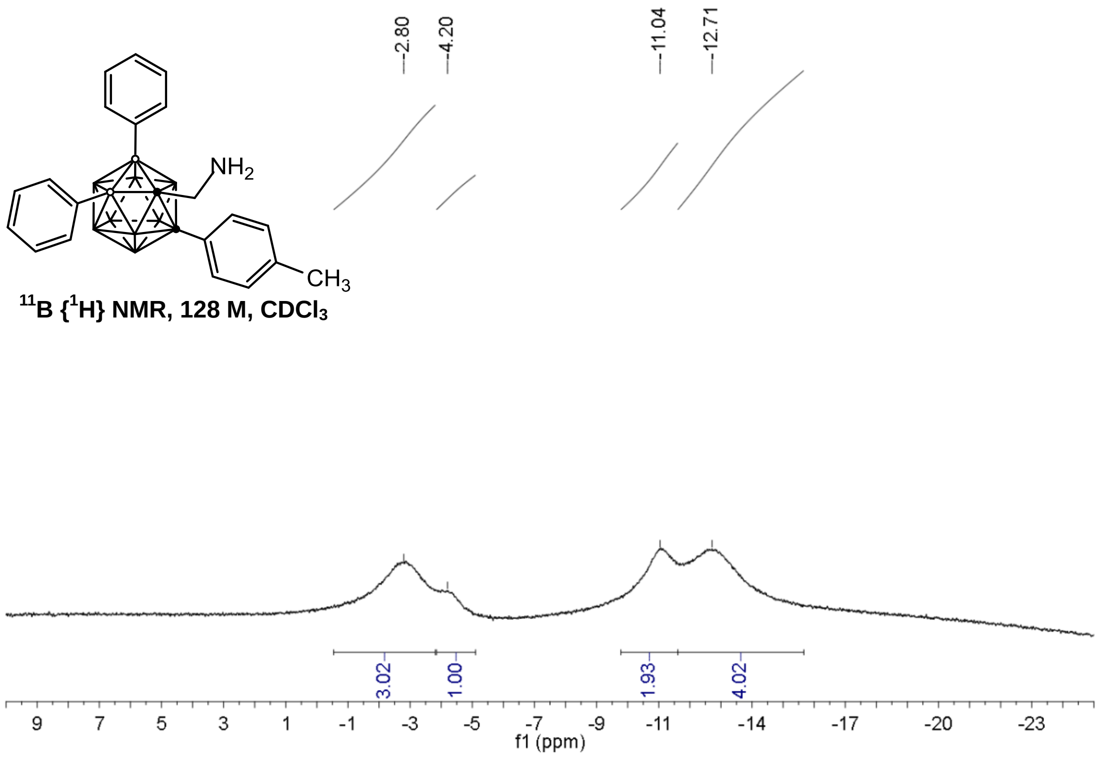

NH2
CH3
11B {1H} NMR, 128 M, CDCl3
-2.80
-4.20
-11.04
-12.71
3.02
1.00
1.93
4.02
f1 (ppm)

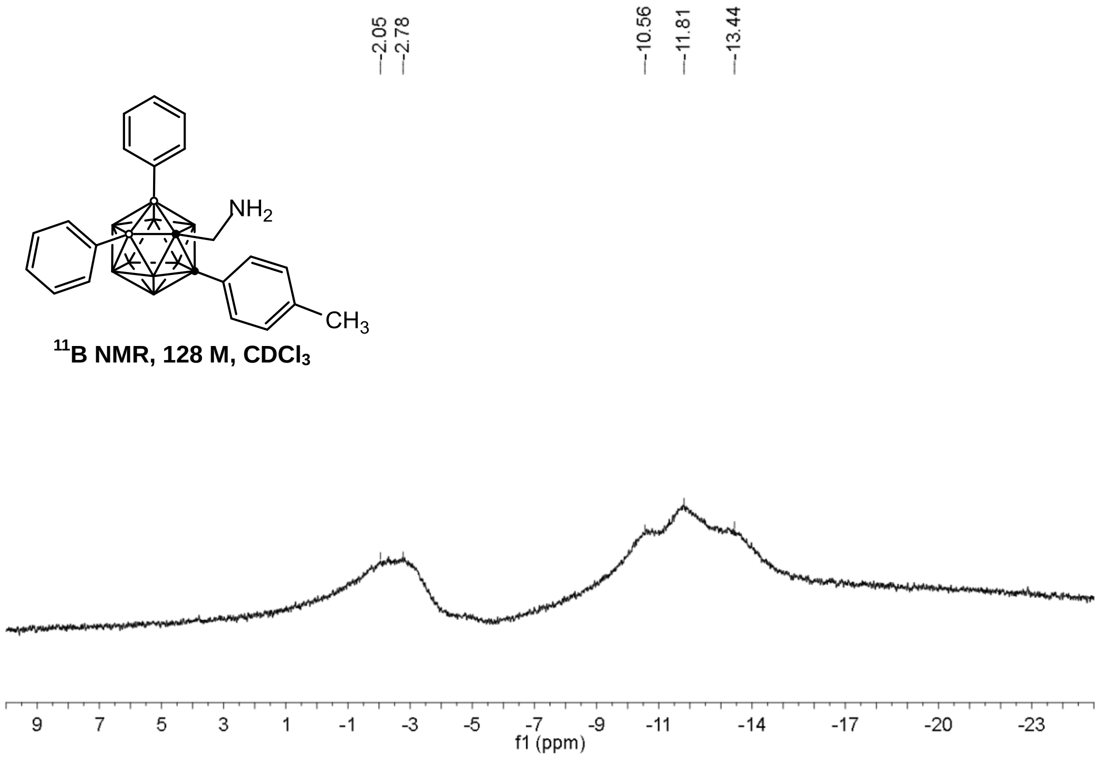

NH2
CH3
11B NMR, 128 M, CDCl3
-2.05
-2.78
-10.56
-11.81
-13.44
f1 (ppm)

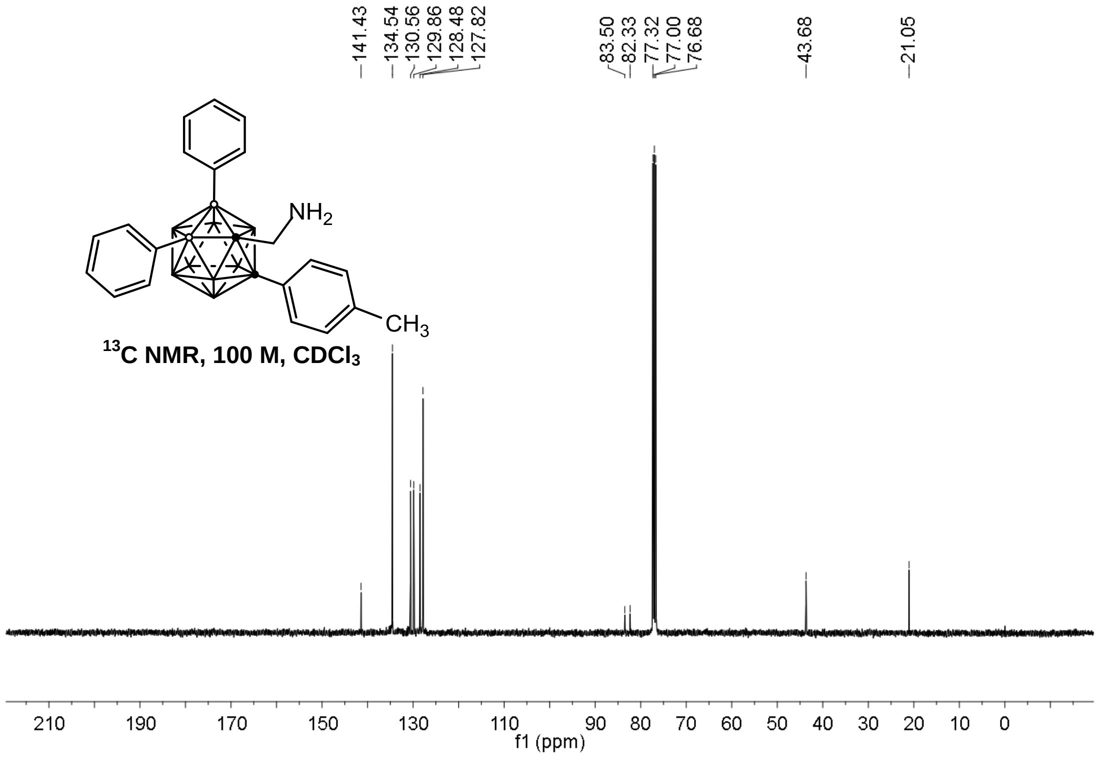

141.43
134.54
130.56
129.86
128.48
127.82
83.50
82.33
77.32
77.00
76.68
43.68
21.05
NH2
CH3
13C NMR, 100 M, CDCl3
210 190 170 150 130 110 90 80 70 60 50 40 30 20 10 0
f1 (ppm)

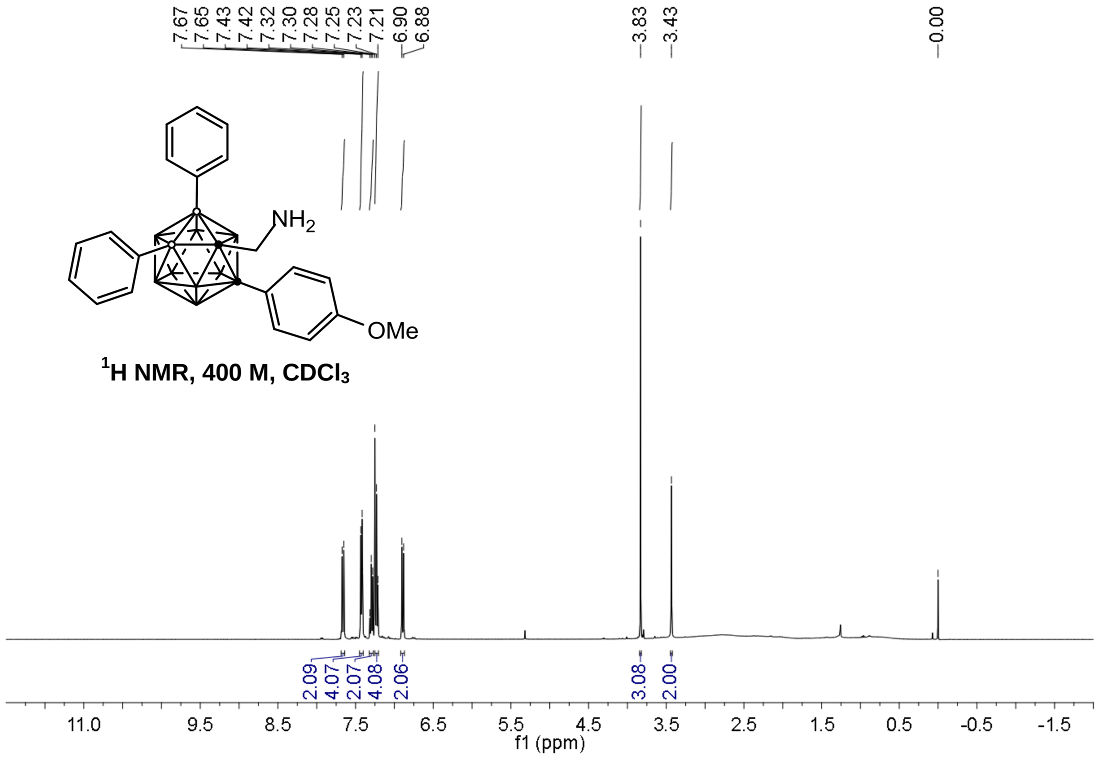

7.67
7.65
7.43
7.42
7.32
7.30
7.28
7.25
7.23
7.21
6.90
6.88
3.83
3.43
0.00
NH2
OMe
1H NMR, 400 M, CDCl3
2.09
4.07
2.07
4.08
2.06
3.08
2.00
11.0 9.5 8.5 7.5 6.5 5.5 4.5 3.5 2.5 1.5 0.5 -0.5 -1.5
f1 (ppm)

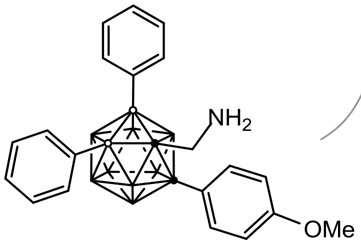

$^{11}B\{^{1}H\}$ NMR, 128 M, $CDCl_3$

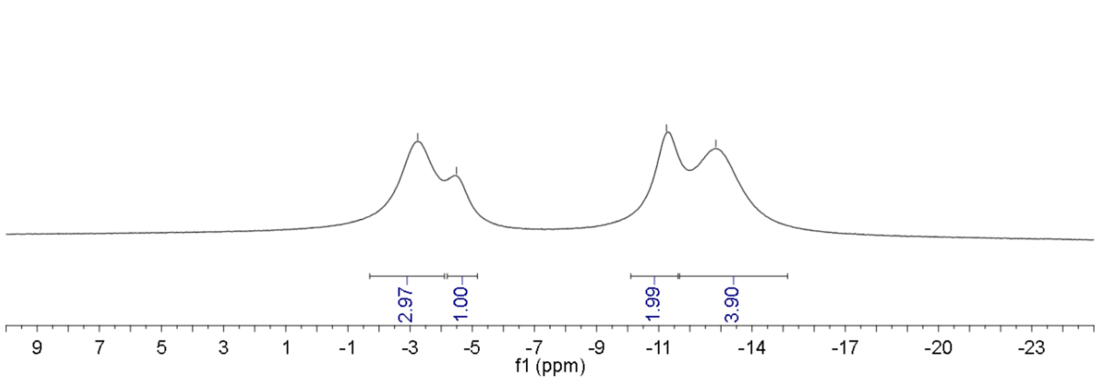

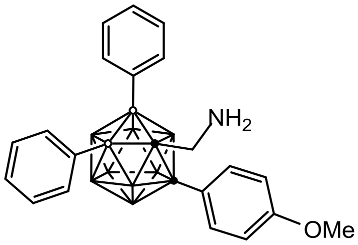

^{11}B NMR, 128 M, $CDCl_3$

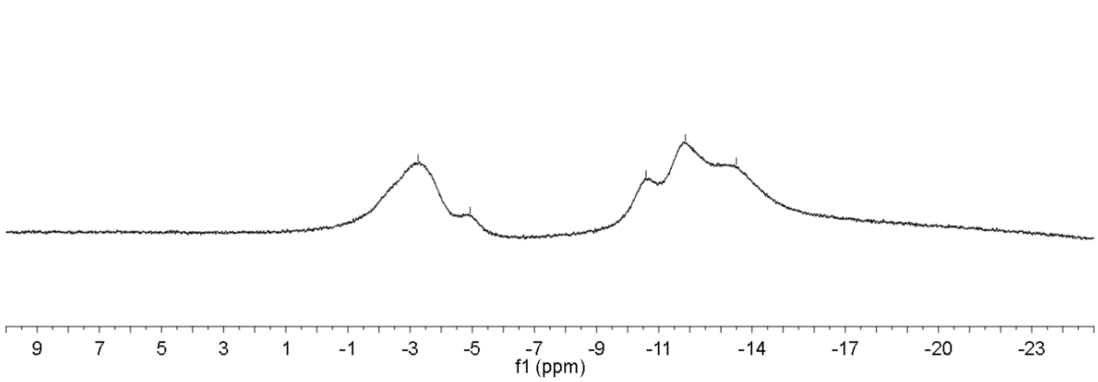

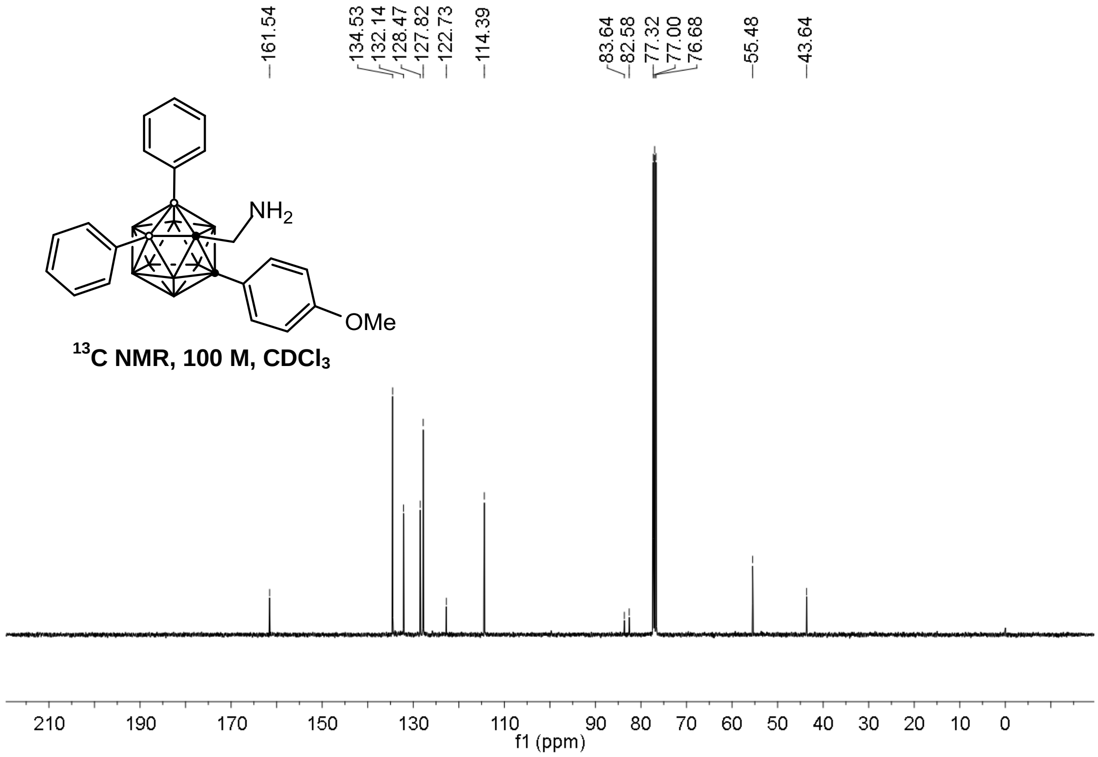

161.54
134.53
132.14
128.47
127.82
122.73
114.39
83.64
82.58
77.32
77.00
76.68
55.48
43.64
NH_2
OMe
^{13}C NMR, 100 M, CDCl$_3$
210 190 170 150 130 110 90 80 70 60 50 40 30 20 10 0
f1 (ppm)

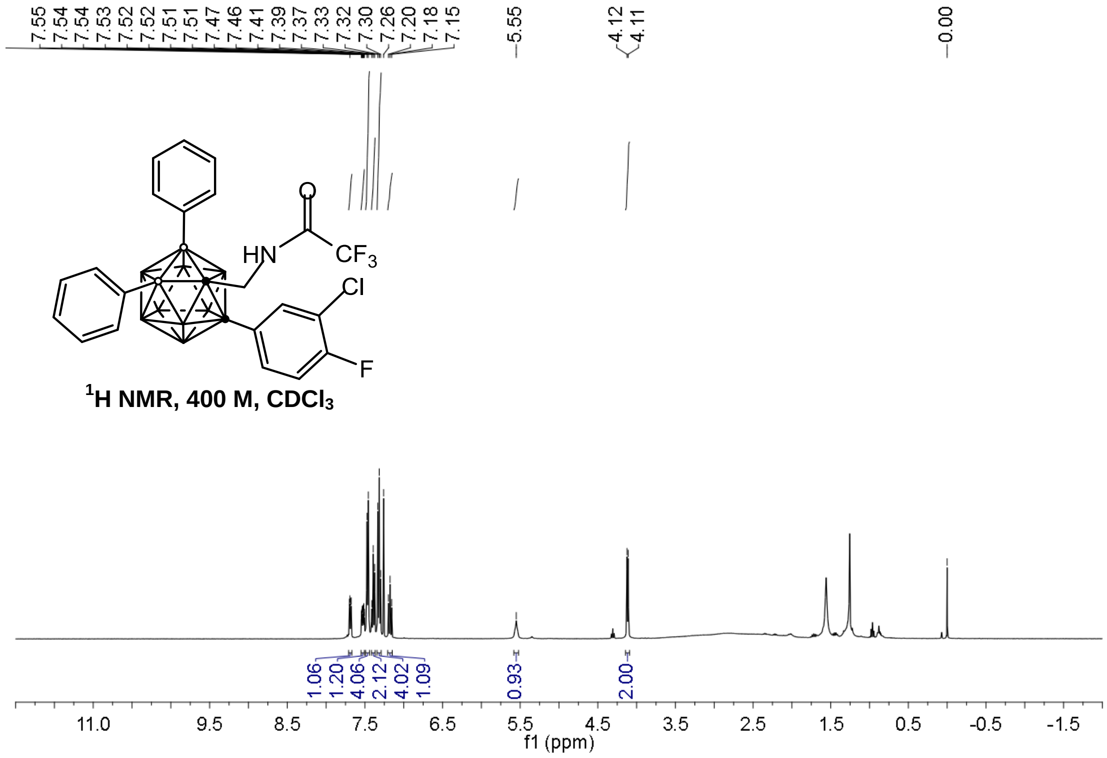

7.55 7.54 7.54 7.53 7.52 7.52 7.51 7.51 7.47 7.46 7.41 7.39 7.37 7.33 7.32 7.30 7.26 7.20 7.18 7.15
5.55
4.12
4.11
0.00
O
HN
CF_3
Cl
F
^{1}H NMR, 400 M, CDCl$_3$
1.06 1.20 4.06 2.12 4.02 1.09
0.93
2.00
11.0 9.5 8.5 7.5 6.5 5.5 4.5 3.5 2.5 1.5 0.5 -0.5 -1.5
f1 (ppm)

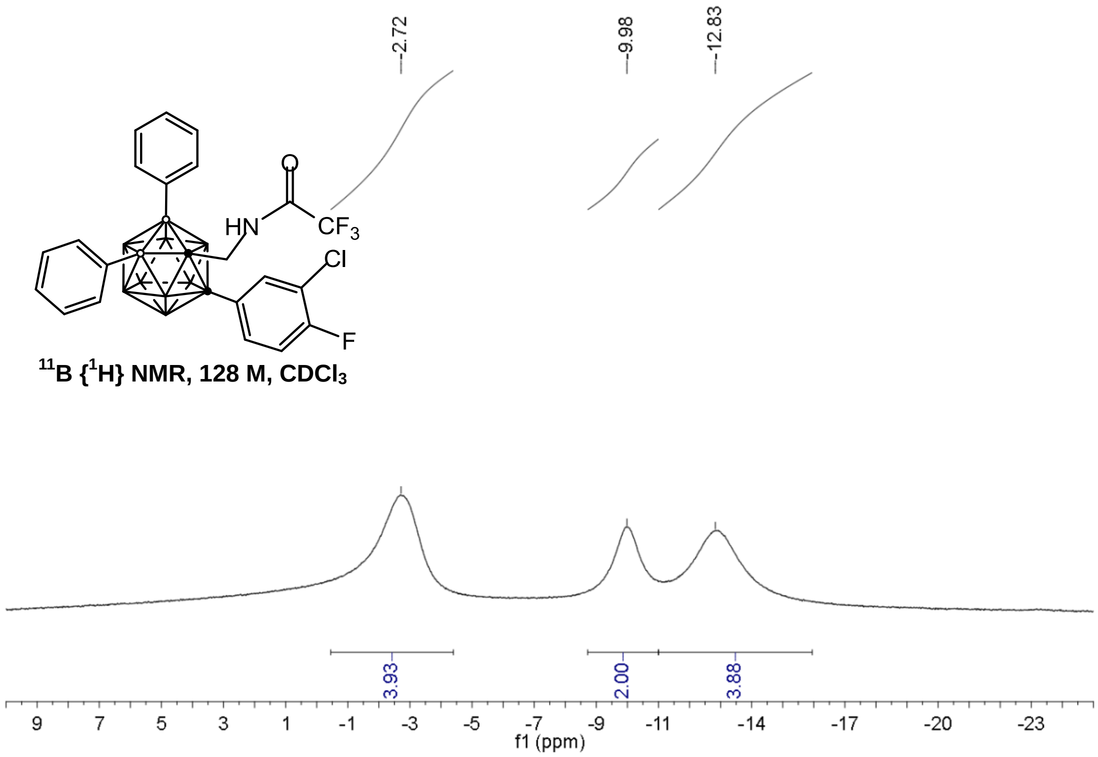

$^{11}B\ \{^{1}H\}$ NMR, 128 M, $CDCl_3$

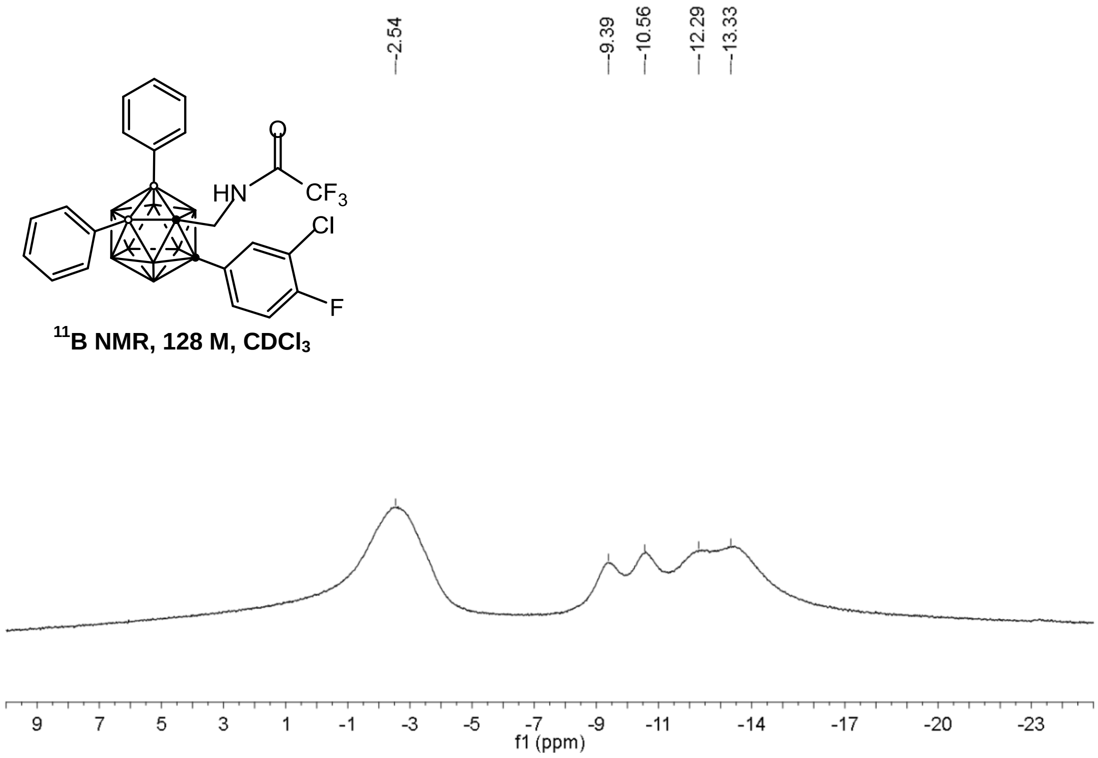

^{11}B NMR, 128 M, $CDCl_3$

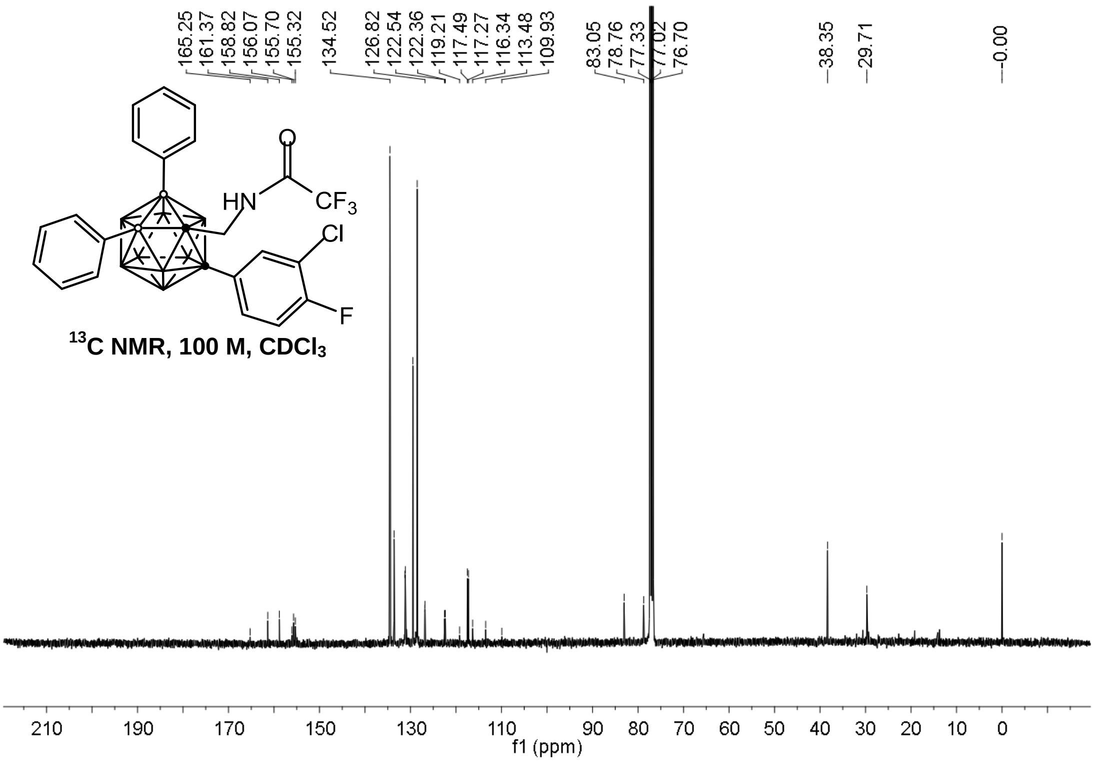
165.25
161.37
158.82
156.07
155.70
155.32
134.52
126.82
122.54
122.36
119.21
117.49
117.27
116.34
113.48
109.93
83.05
78.76
77.33
77.02
76.70
38.35
29.71
0.00
HN
O
CF3
Cl
F
13C NMR, 100 M, CDCl3
210
190
170
150
130
110
90
80
70
60
50
40
30
20
10
0
f1 (ppm)

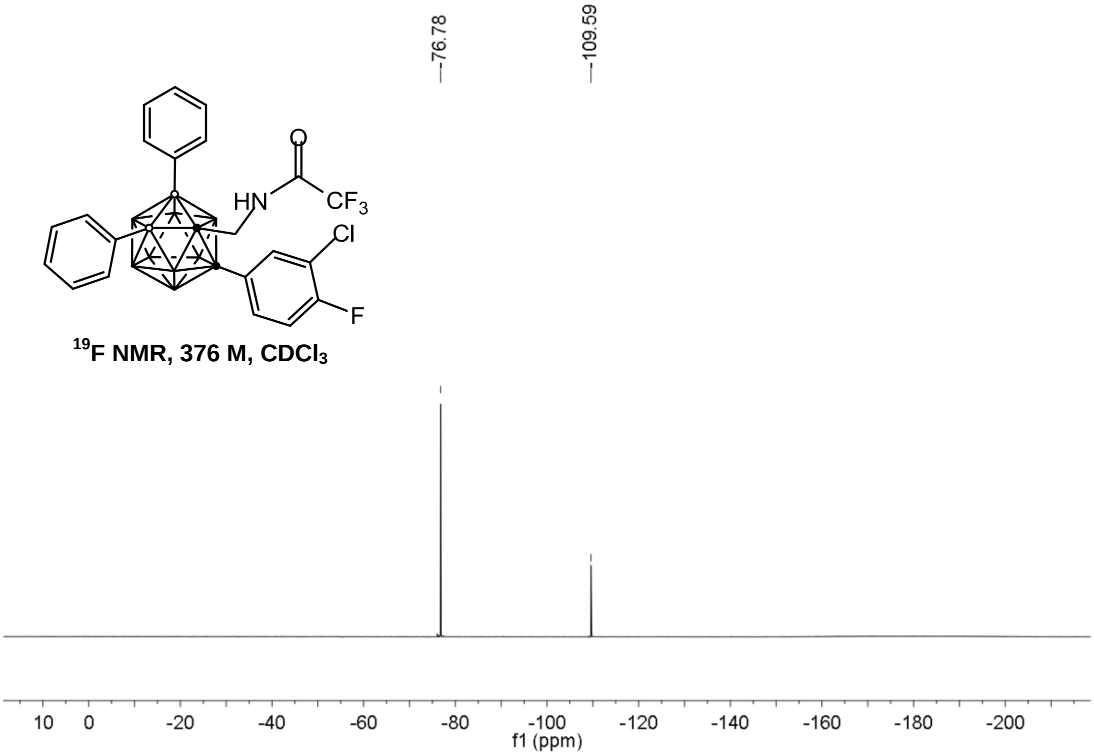
-76.78
-109.59
HN
O
CF3
Cl
F
19F NMR, 376 M, CDCl3
10
0
-20
-40
-60
-80
-100
-120
-140
-160
-180
-200
f1 (ppm)

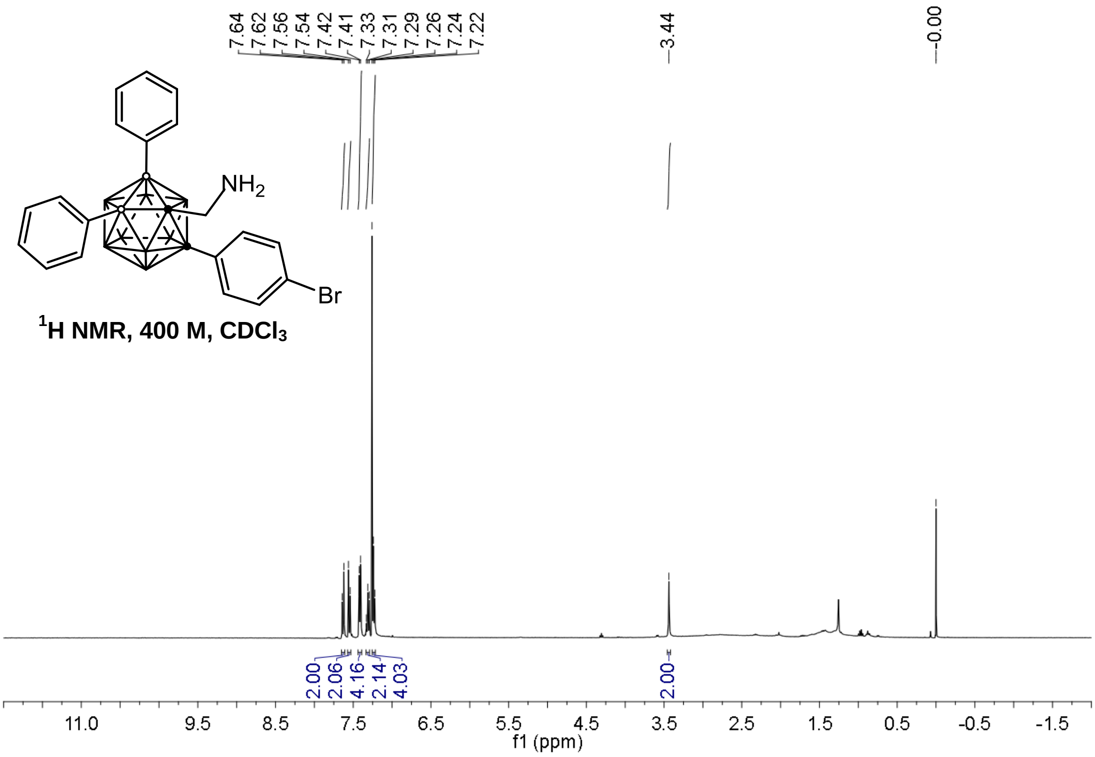

7.64
7.62
7.56
7.54
7.42
7.41
7.33
7.31
7.29
7.26
7.24
7.22
3.44
0.00
NH2
Br
1H NMR, 400 M, CDCl3
2.00
2.06
4.16
2.14
4.03
2.00
11.0
9.5
8.5
7.5
6.5
5.5
4.5
3.5
2.5
1.5
0.5
-0.5
-1.5
f1 (ppm)

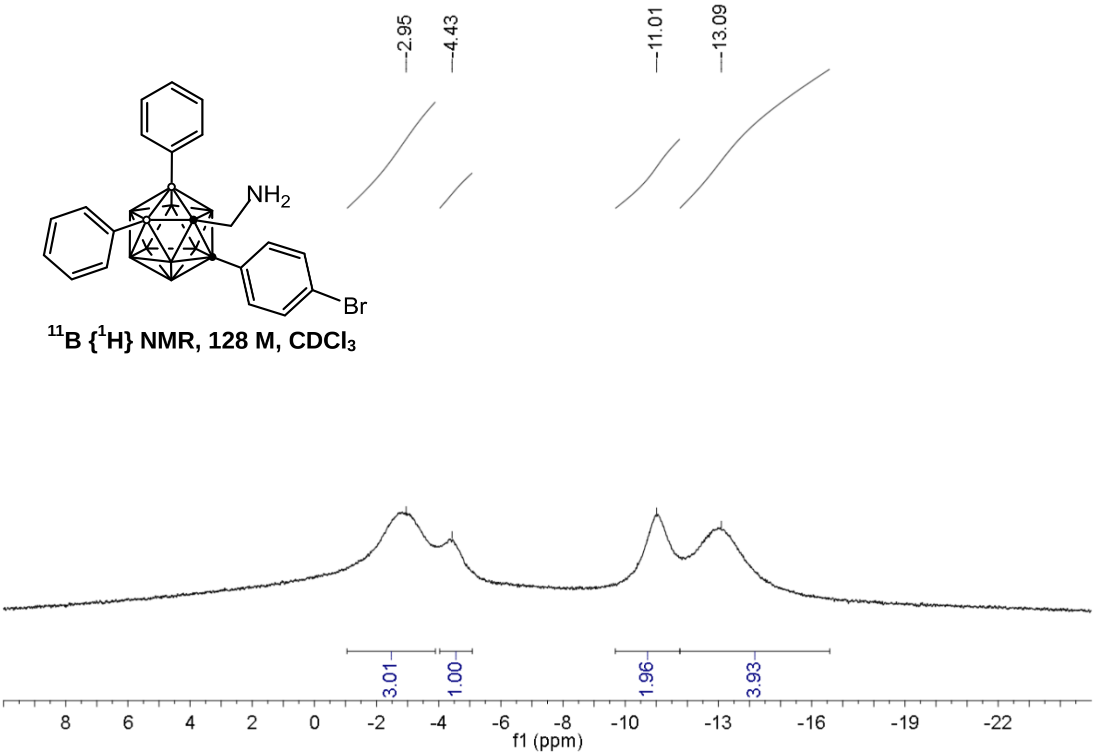

-2.95
-4.43
-11.01
-13.09
NH2
Br
11B {1H} NMR, 128 M, CDCl3
3.01
1.00
1.96
3.93
8
6
4
2
0
-2
-4
-6
-8
-10
-13
-16
-19
-22
f1 (ppm)

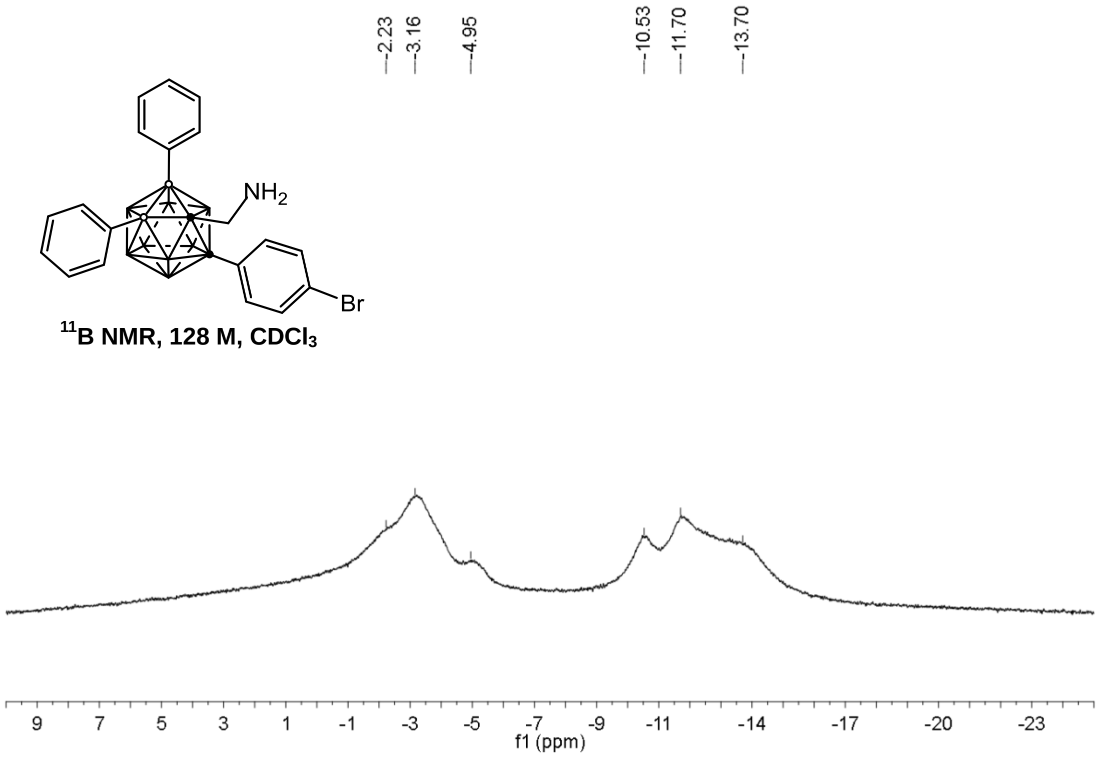

2.23
3.16
4.95
10.53
11.70
13.70
NH2
Br
11B NMR, 128 M, CDCl3
9 7 5 3 1 -1 -3 -5 -7 -9 -11 -14 -17 -20 -23
f1 (ppm)

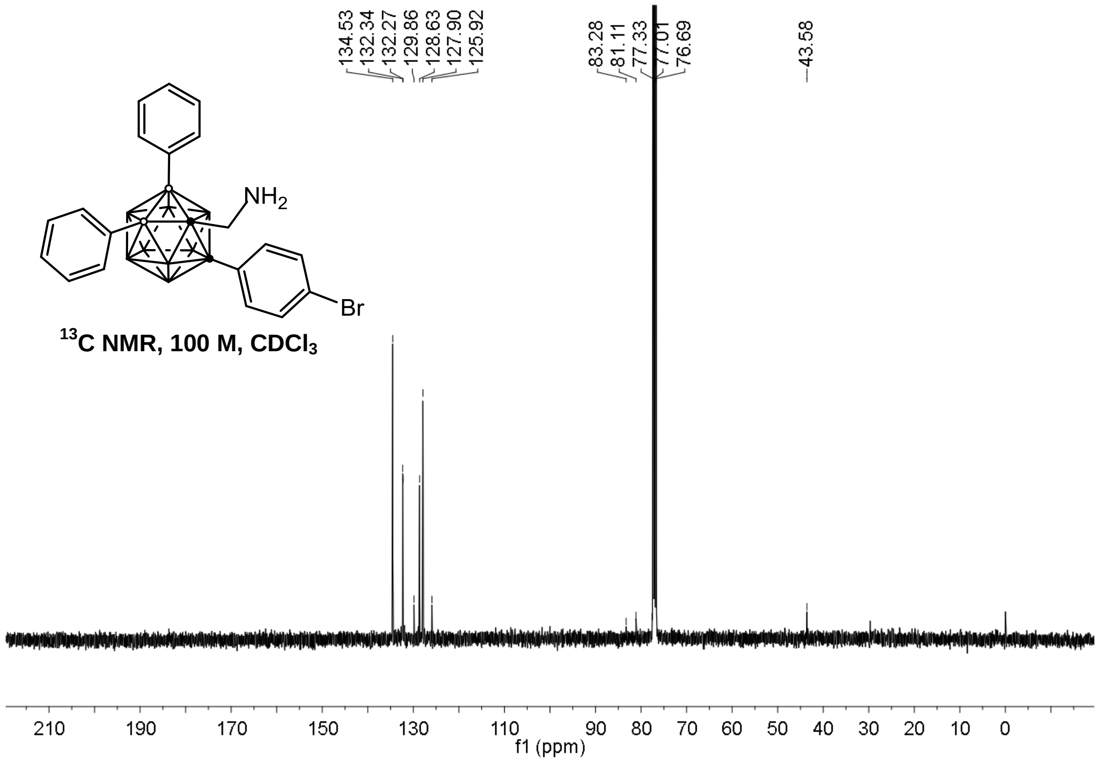

134.53
132.34
132.27
129.86
128.63
127.90
125.92
83.28
81.11
77.33
77.01
76.69
43.58
NH2
Br
13C NMR, 100 M, CDCl3
210 190 170 150 130 110 90 80 70 60 50 40 30 20 10 0
f1 (ppm)

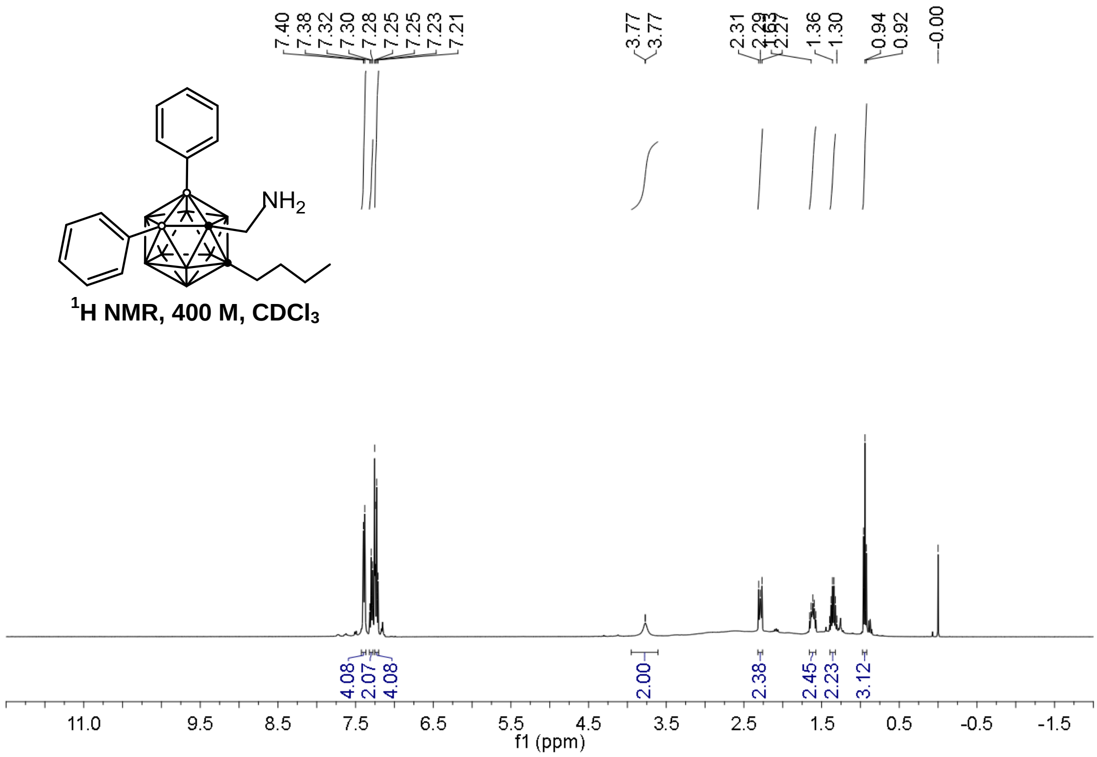

NH2
1H NMR, 400 M, CDCl3
7.40
7.38
7.32
7.30
7.28
7.25
7.25
7.23
7.21
3.77
3.77
2.31
2.29
2.27
1.36
1.30
0.94
0.92
0.00
4.08
2.07
4.08
2.00
2.38
2.45
2.23
3.12
f1 (ppm)

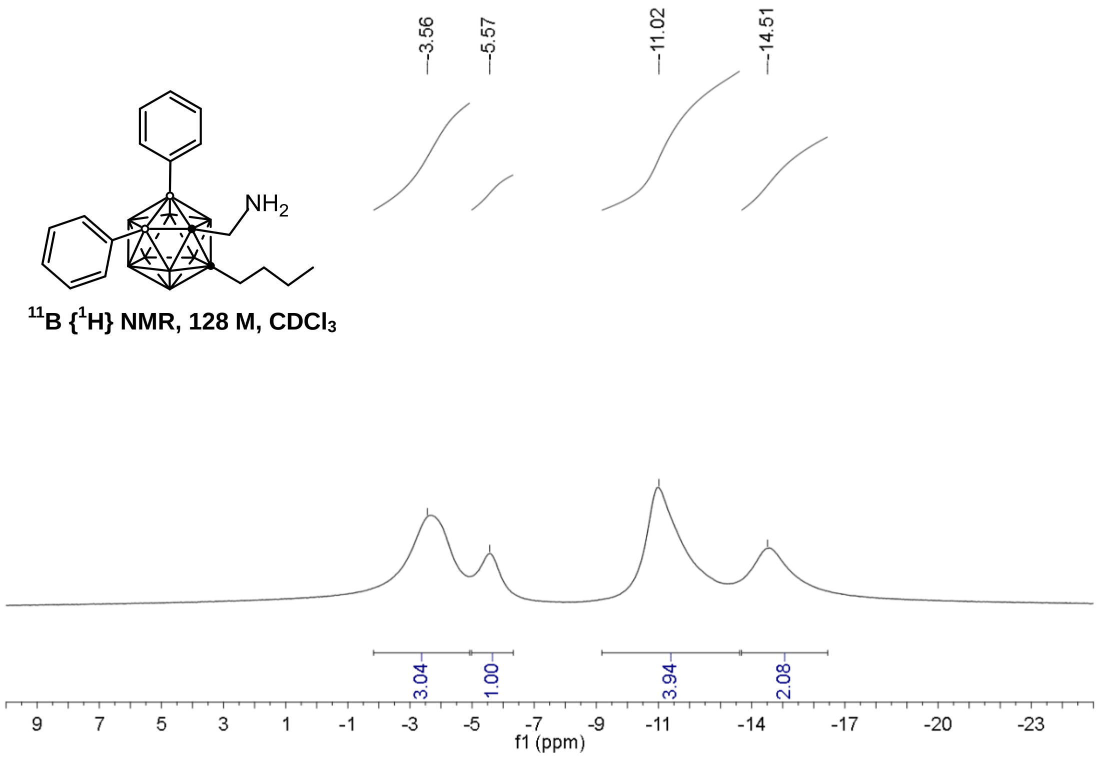

NH2
11B {1H} NMR, 128 M, CDCl3
-3.56
-5.57
-11.02
-14.51
3.04
1.00
3.94
2.08
f1 (ppm)

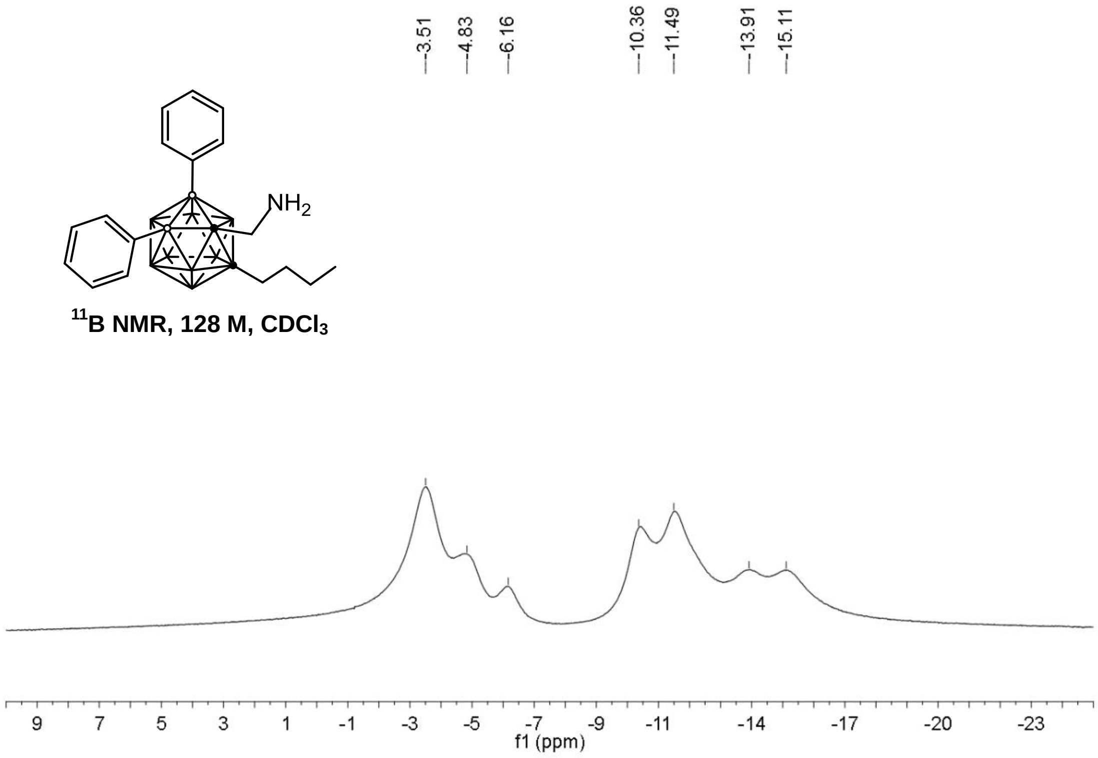

NH2
11B NMR, 128 M, CDCl3
-3.51
-4.83
-6.16
-10.36
-11.49
-13.91
-15.11
f1 (ppm)

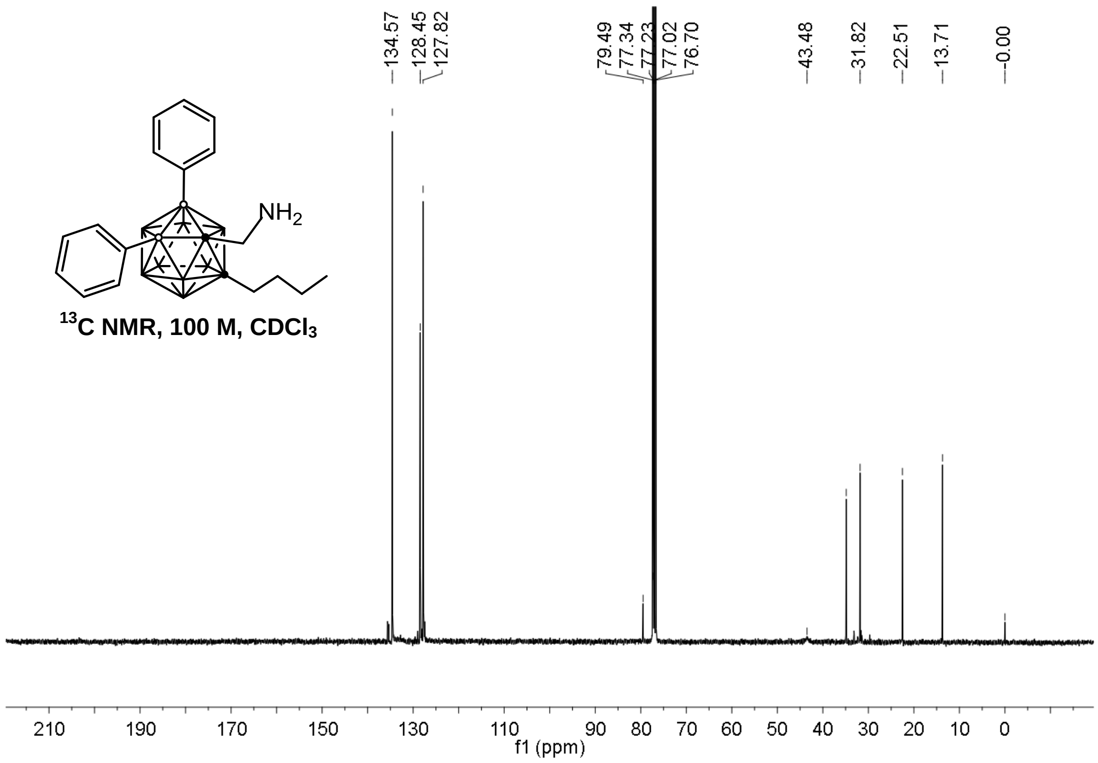

NH2
13C NMR, 100 M, CDCl3
134.57
128.45
127.82
79.49
77.34
77.23
77.02
76.70
43.48
31.82
22.51
13.71
0.00
f1 (ppm)

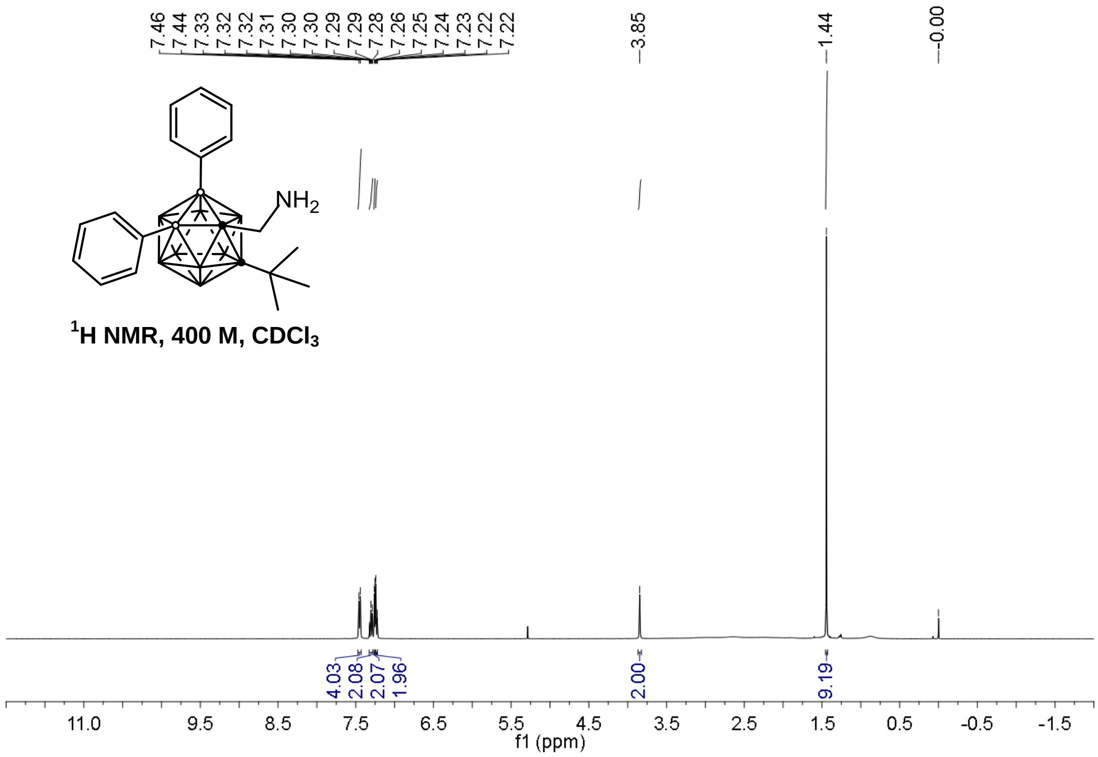

7.46
7.44
7.33
7.32
7.32
7.31
7.30
7.30
7.29
7.29
7.28
7.26
7.25
7.24
7.23
7.22
7.22
3.85
1.44
0.00
NH2
1H NMR, 400 M, CDCl3
4.03
2.08
2.07
1.96
2.00
9.19
f1 (ppm)

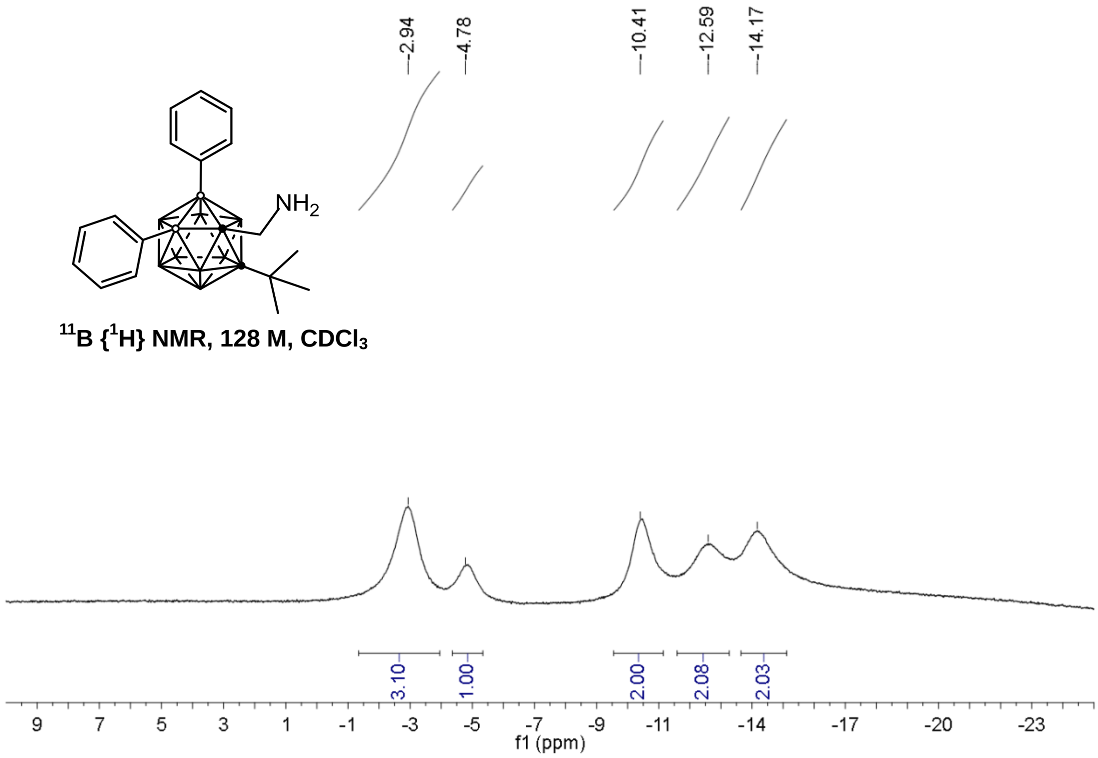

-2.94
-4.78
-10.41
-12.59
-14.17
NH2
11B {1H} NMR, 128 M, CDCl3
3.10
1.00
2.00
2.08
2.03
f1 (ppm)

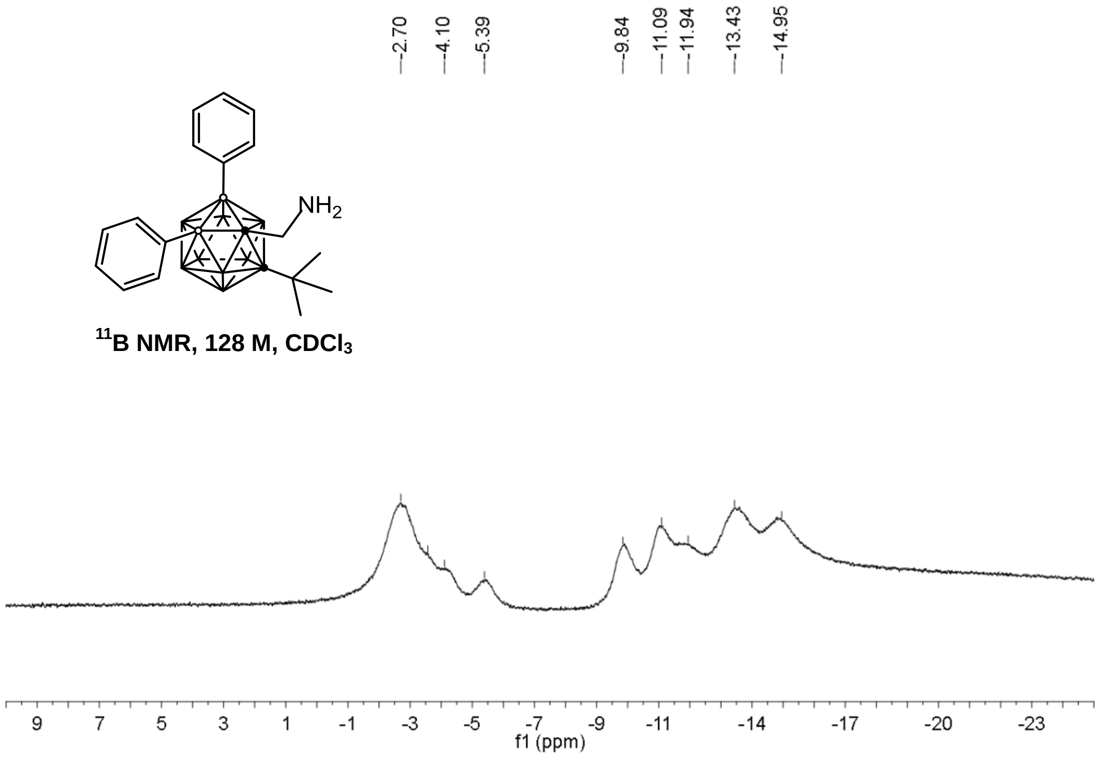
-2.70
-4.10
-5.39
-9.84
-11.09
-11.94
-13.43
-14.95
NH2
11B NMR, 128 M, CDCl3
9 7 5 3 1 -1 -3 -5 -7 -9 -11 -14 -17 -20 -23
f1 (ppm)

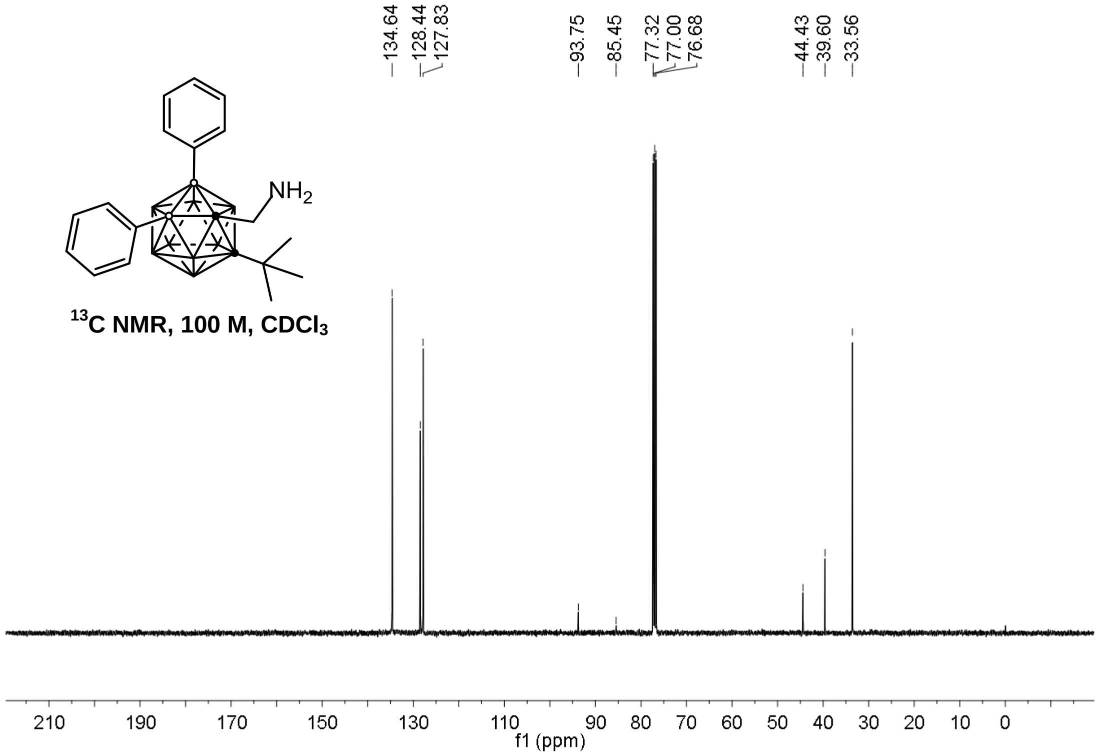
134.64
128.44
127.83
93.75
85.45
77.32
77.00
76.68
44.43
39.60
33.56
NH2
13C NMR, 100 M, CDCl3
210 190 170 150 130 110 90 80 70 60 50 40 30 20 10 0
f1 (ppm)

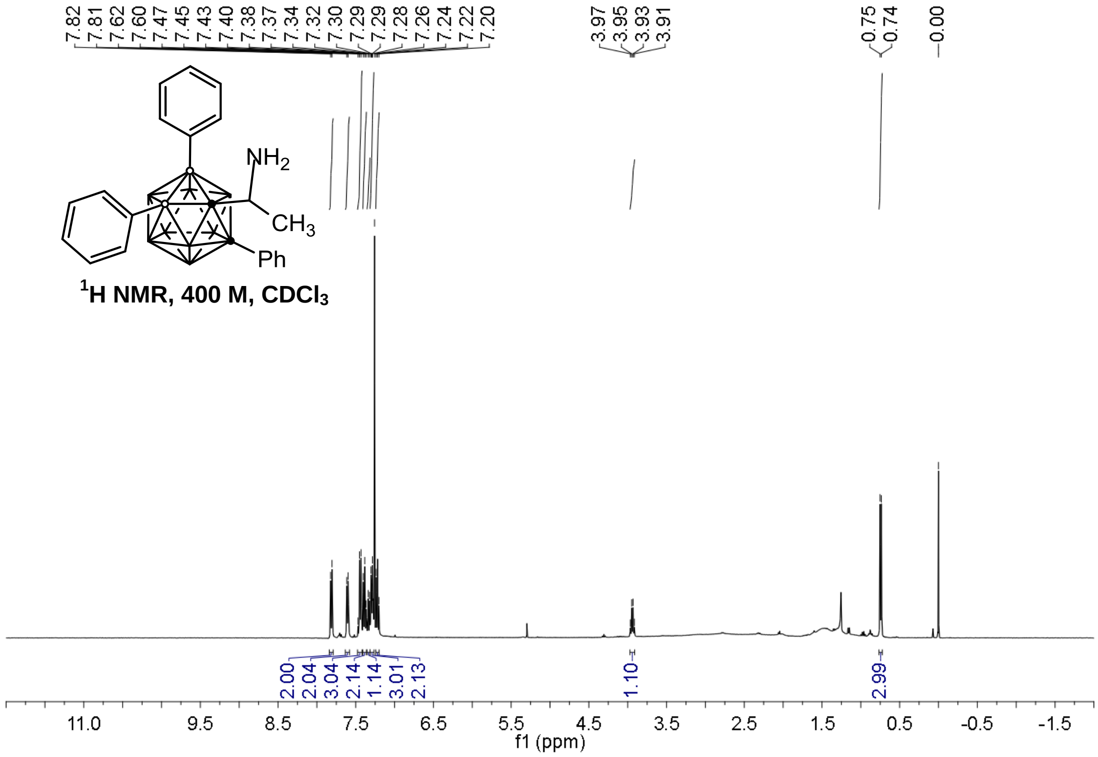
7.82
7.81
7.62
7.60
7.47
7.45
7.43
7.40
7.38
7.37
7.34
7.32
7.30
7.29
7.29
7.28
7.26
7.24
7.22
7.20
3.97
3.95
3.93
3.91
0.75
0.74
0.00
NH2
CH3
Ph
1H NMR, 400 M, CDCl3
2.00
2.04
3.04
2.14
1.14
3.01
2.13
1.10
2.99
f1 (ppm)

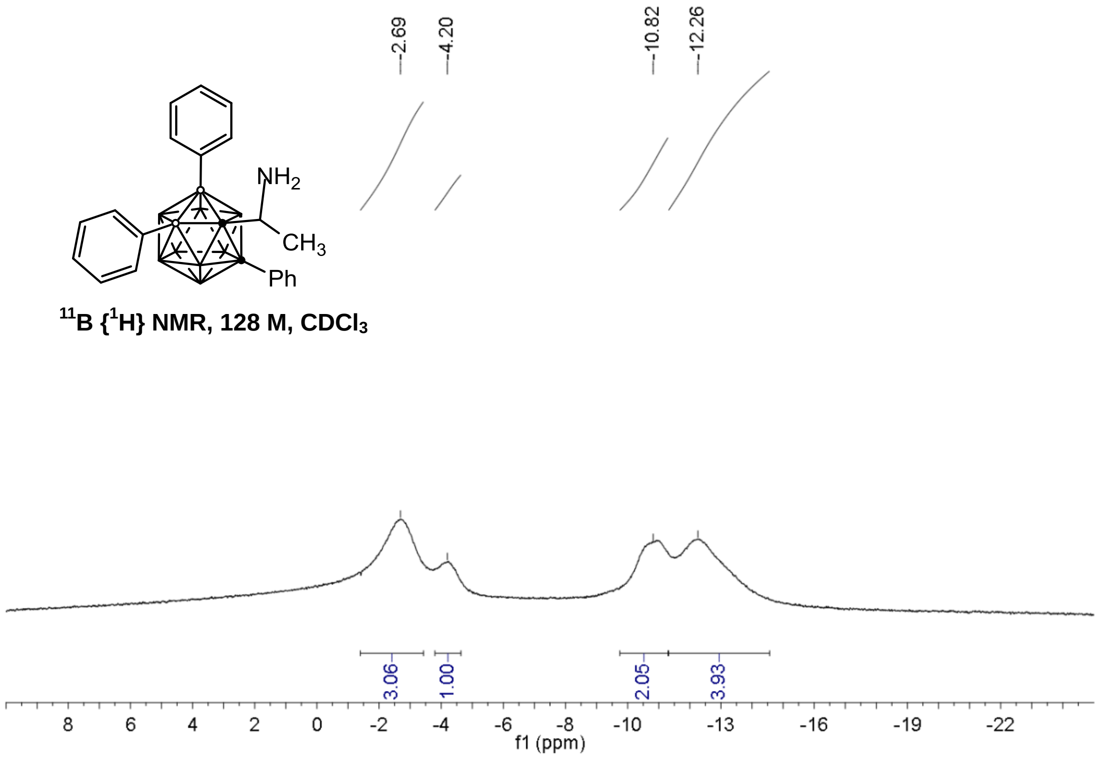
-2.69
-4.20
-10.82
-12.26
NH2
CH3
Ph
11B {1H} NMR, 128 M, CDCl3
3.06
1.00
2.05
3.93
f1 (ppm)

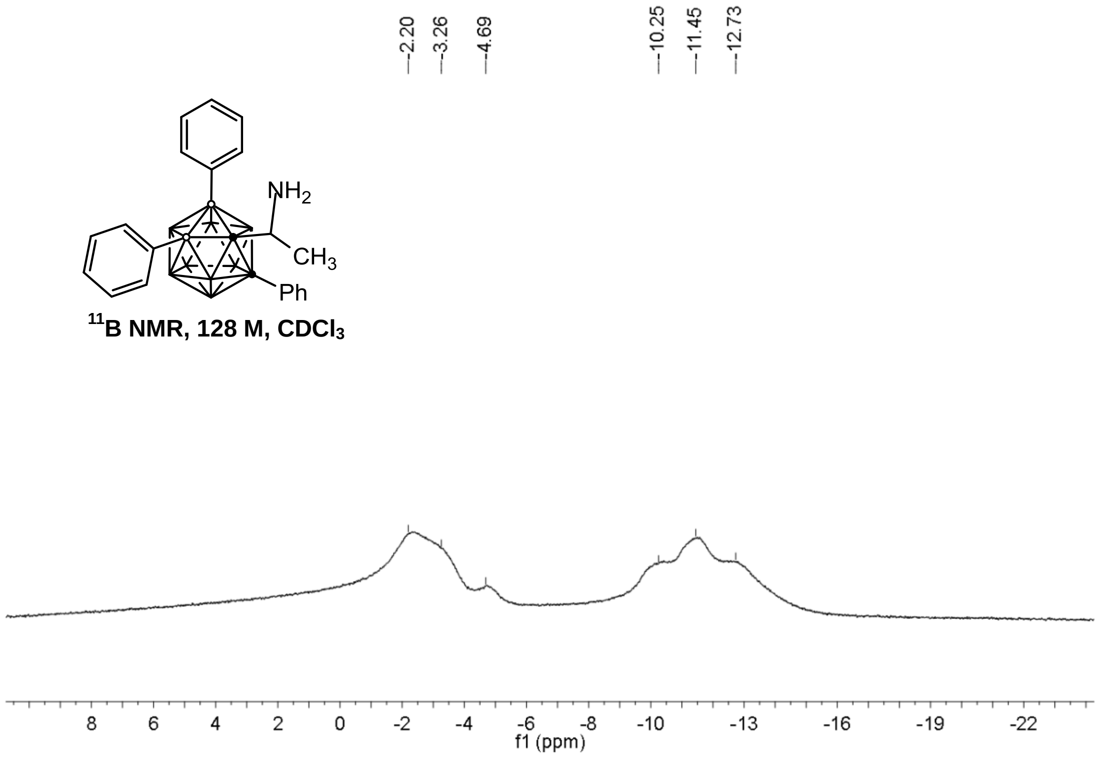

-2.20
-3.26
-4.69
-10.25
-11.45
-12.73
NH2
CH3
Ph
11B NMR, 128 M, CDCl3
8 6 4 2 0 -2 -4 -6 -8 -10 -13 -16 -19 -22
f1 (ppm)

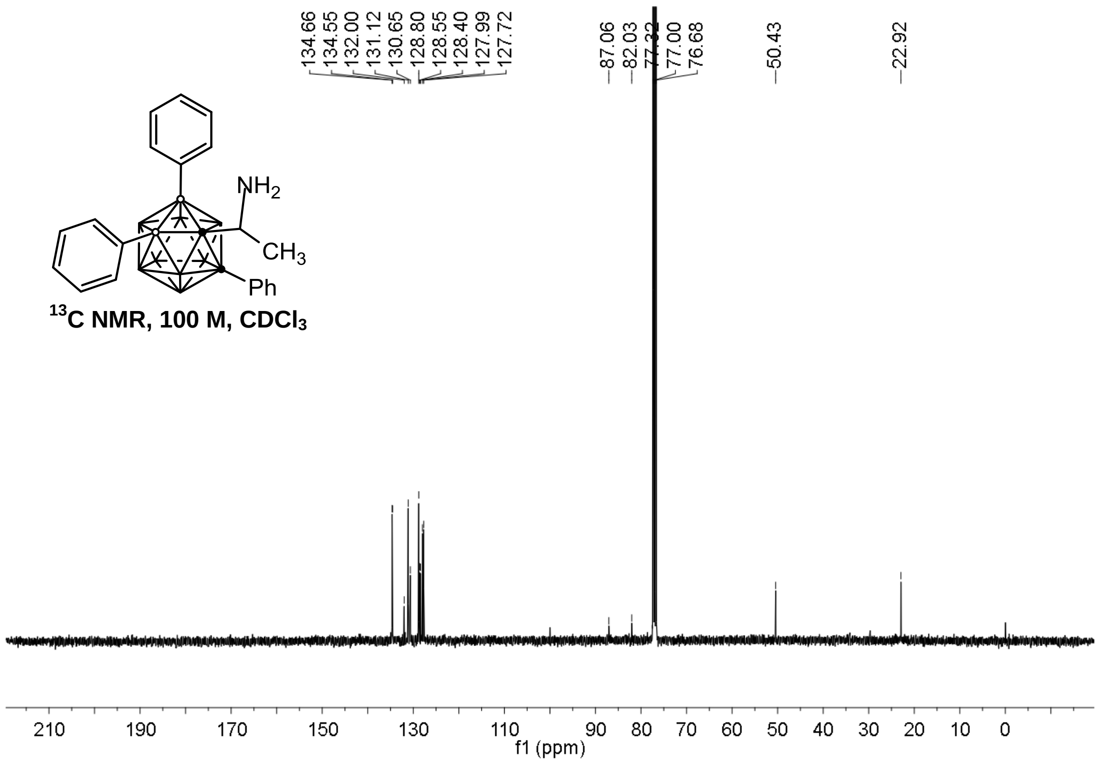

134.66
134.55
132.00
131.12
130.65
128.80
128.55
128.40
127.99
127.72
87.06
82.03
77.32
77.00
76.68
50.43
22.92
NH2
CH3
Ph
13C NMR, 100 M, CDCl3
210 190 170 150 130 110 90 80 70 60 50 40 30 20 10 0
f1 (ppm)

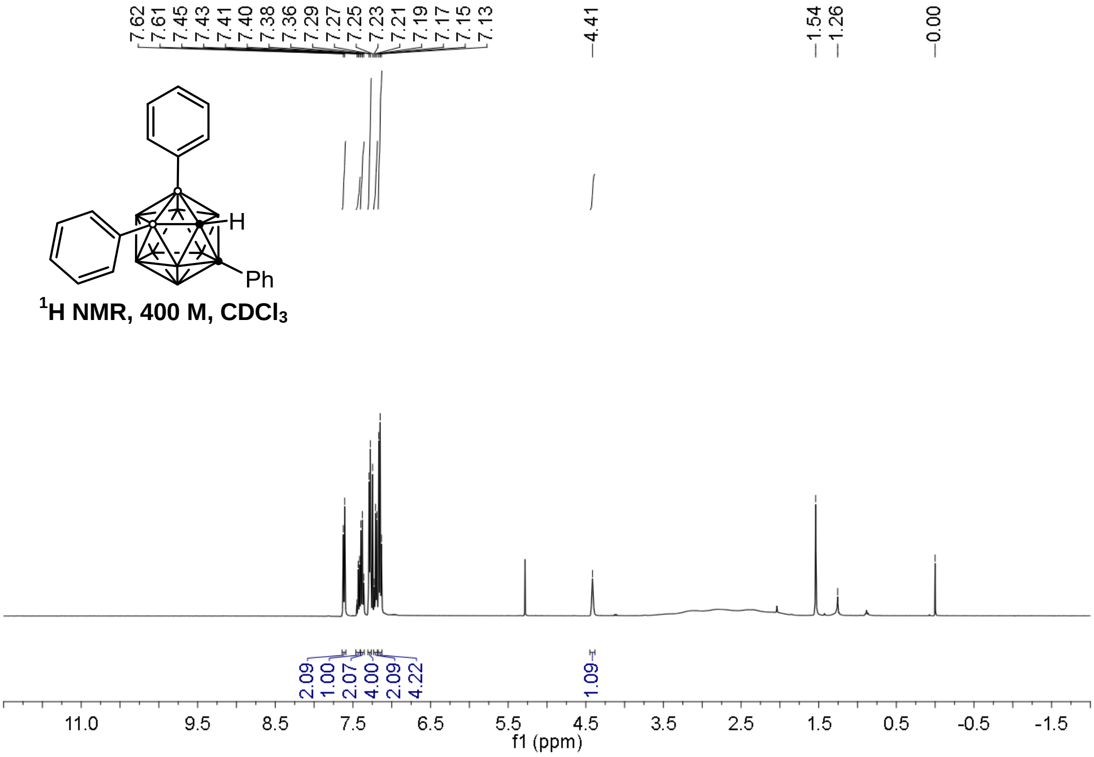
7.62
7.61
7.45
7.43
7.41
7.40
7.38
7.36
7.29
7.27
7.25
7.23
7.21
7.19
7.17
7.15
7.13
4.41
1.54
1.26
0.00
H
Ph
^{1}H NMR, 400 M, $CDCl_3$
2.09
1.00
2.07
4.00
2.09
4.22
1.09
11.0
9.5
8.5
7.5
6.5
5.5
4.5
3.5
2.5
1.5
0.5
-0.5
-1.5
f1 (ppm)

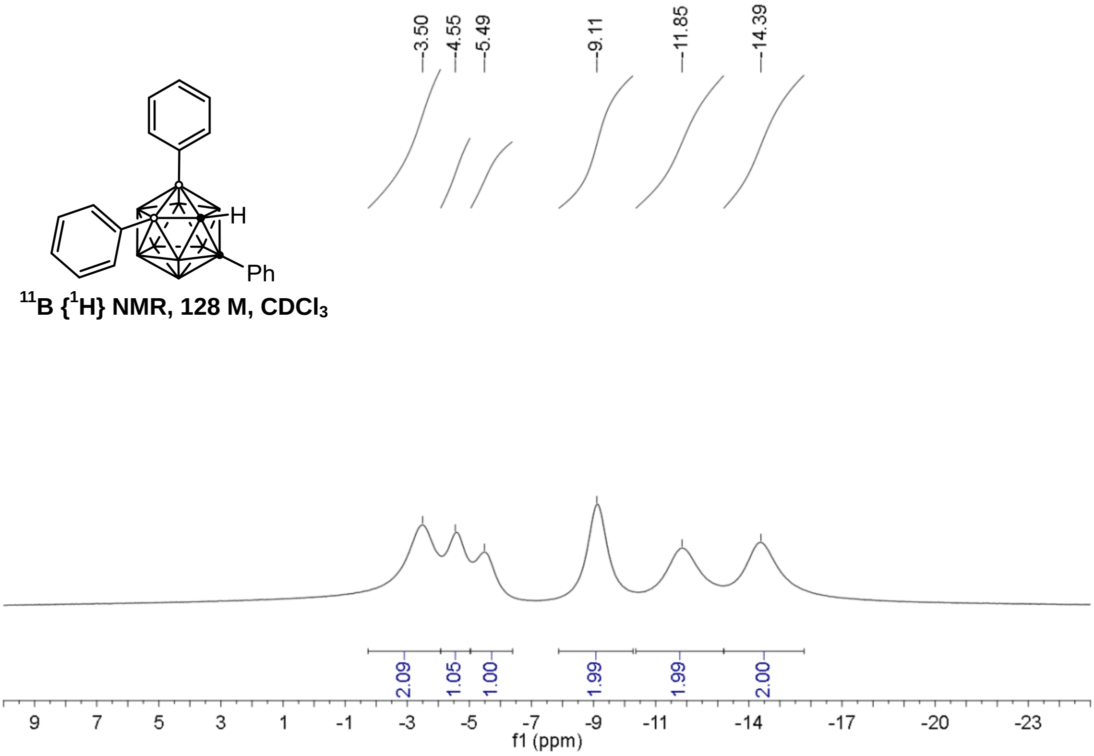
-3.50
-4.55
-5.49
-9.11
-11.85
-14.39
H
Ph
$^{11}B\{^{1}H\}$ NMR, 128 M, $CDCl_3$
2.09
1.05
1.00
1.99
1.99
2.00
9
7
5
3
1
-1
-3
-5
-7
-9
-11
-14
-17
-20
-23
f1 (ppm)

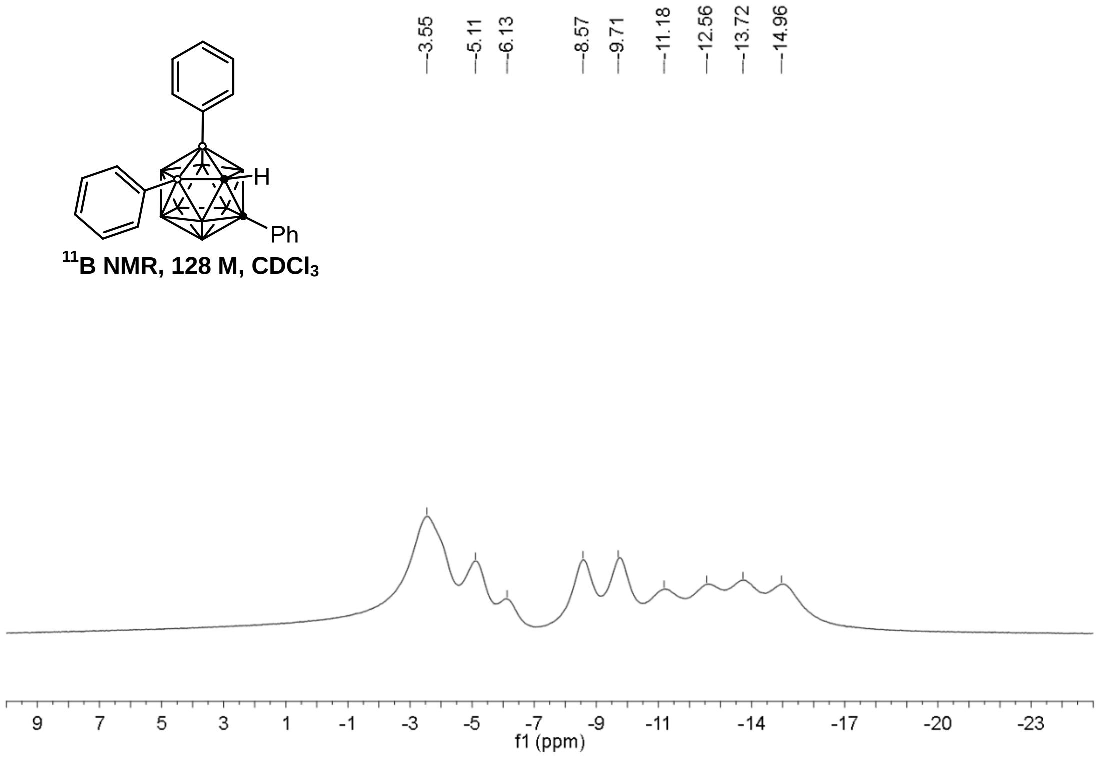

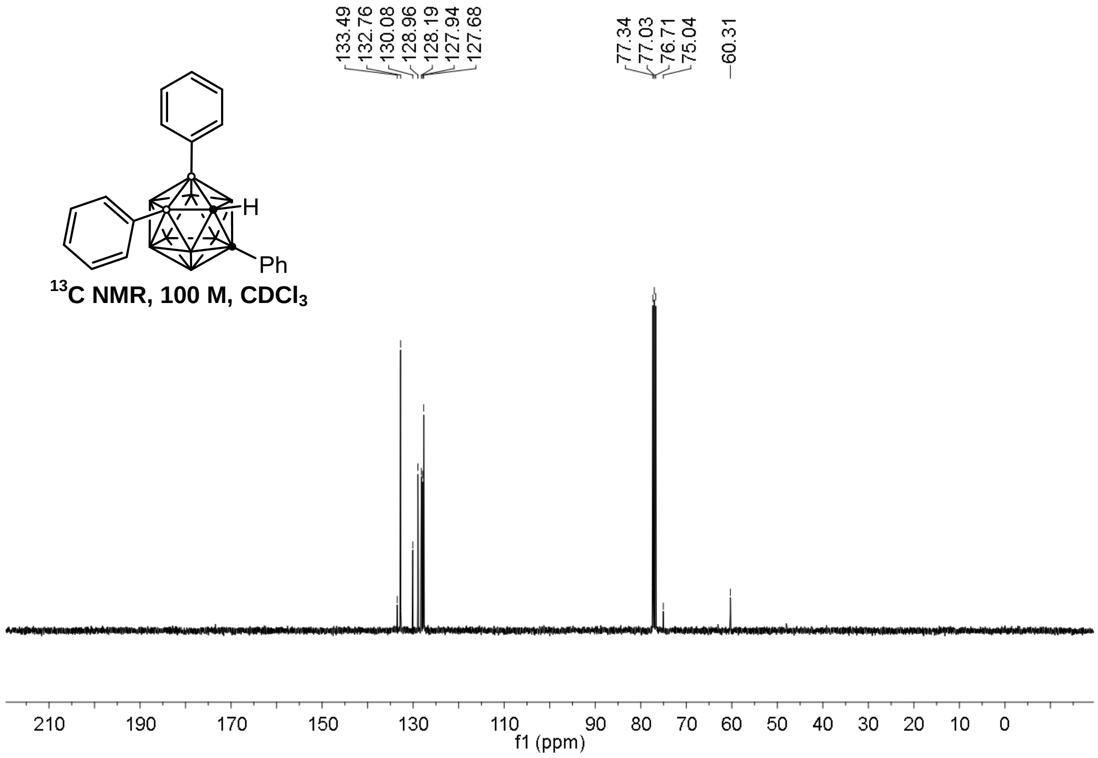

5.3 Mechanistic Study

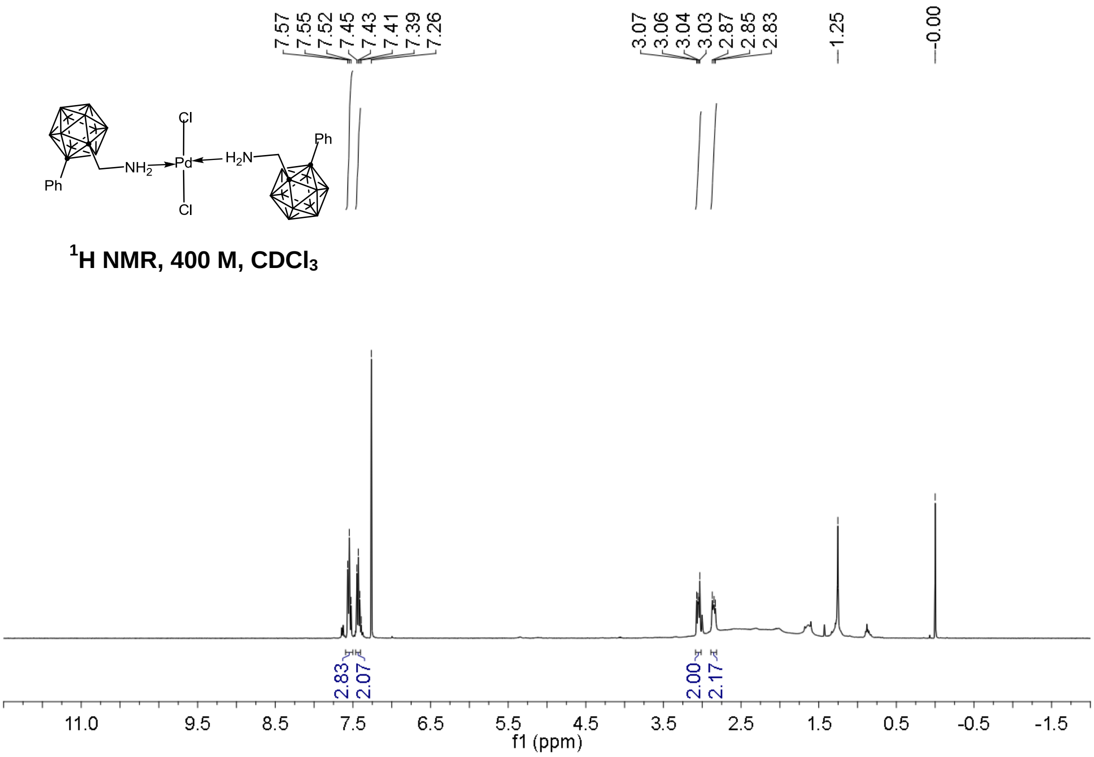
7.57
7.55
7.52
7.45
7.43
7.41
7.39
7.26
3.07
3.06
3.04
3.03
2.87
2.85
2.83
1.25
0.00
Cl
Ph
NH2
Pd
H2N
Ph
Cl
1H NMR, 400 M, CDCl3
2.83
2.07
2.00
2.17
11.0
9.5
8.5
7.5
6.5
5.5
4.5
3.5
2.5
1.5
0.5
-0.5
-1.5
f1 (ppm)

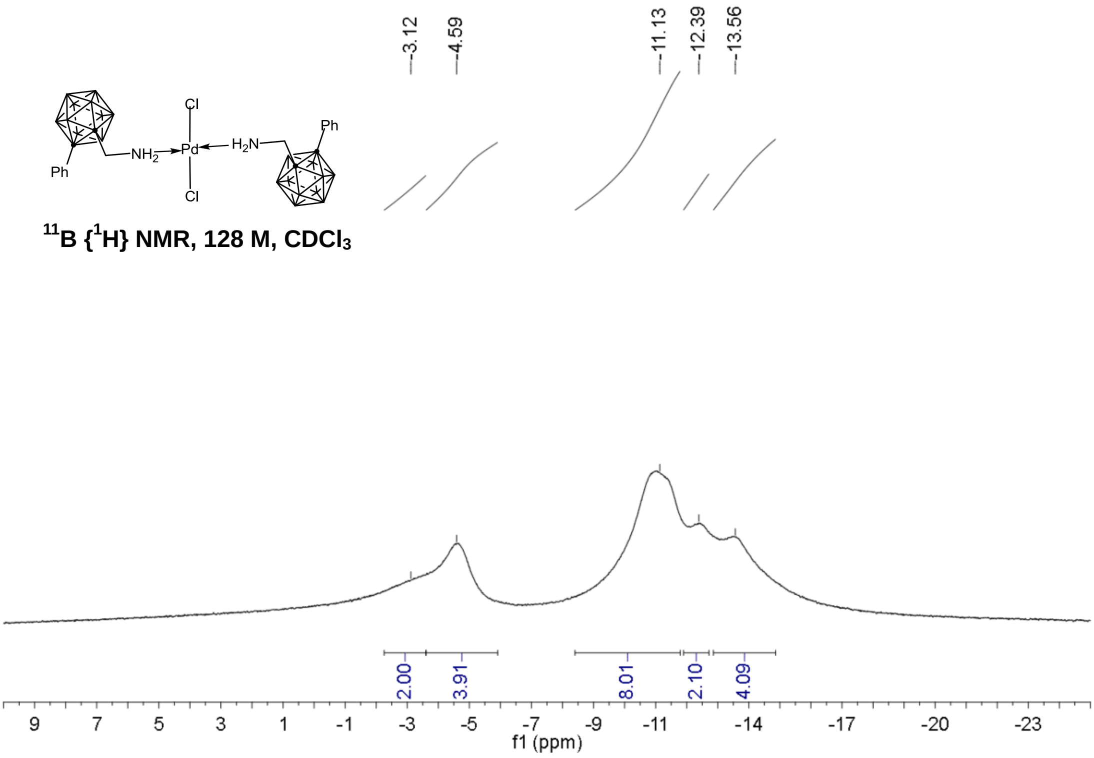
-3.12
-4.59
-11.13
-12.39
-13.56
Cl
Ph
NH2
Pd
H2N
Ph
Cl
11B {1H} NMR, 128 M, CDCl3
2.00
3.91
8.01
2.10
4.09
9
7
5
3
1
-1
-3
-5
-7
-9
-11
-14
-17
-20
-23
f1 (ppm)

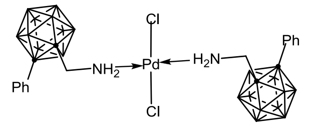

^{11}B NMR, 128 M, CDCl$_3$

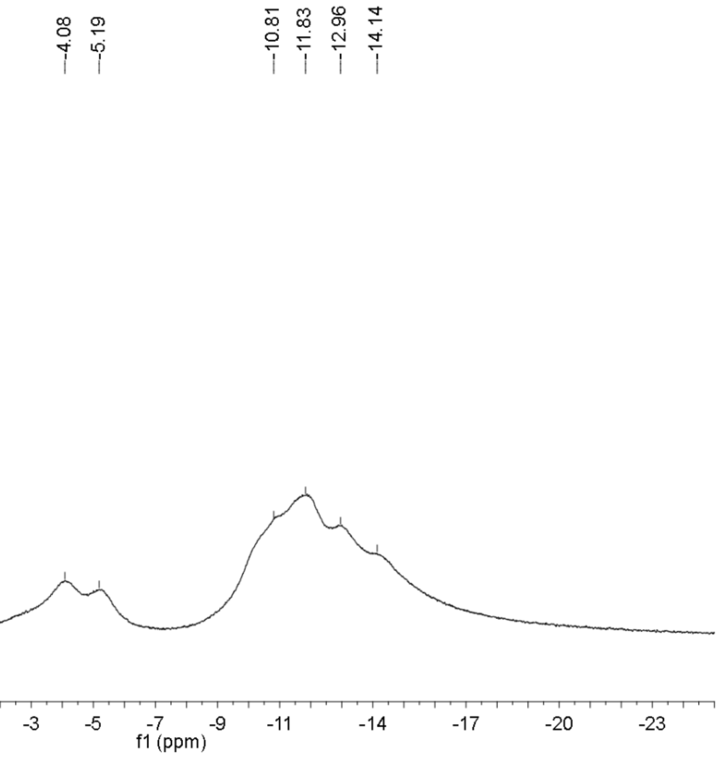

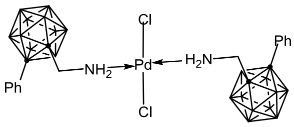

^{13}C NMR, 100 M, CDCl$_3$

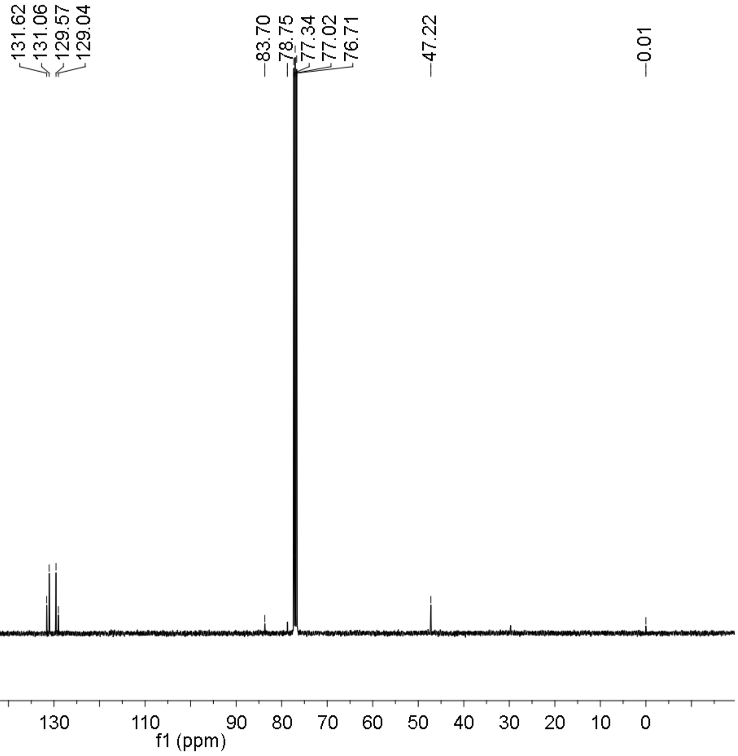

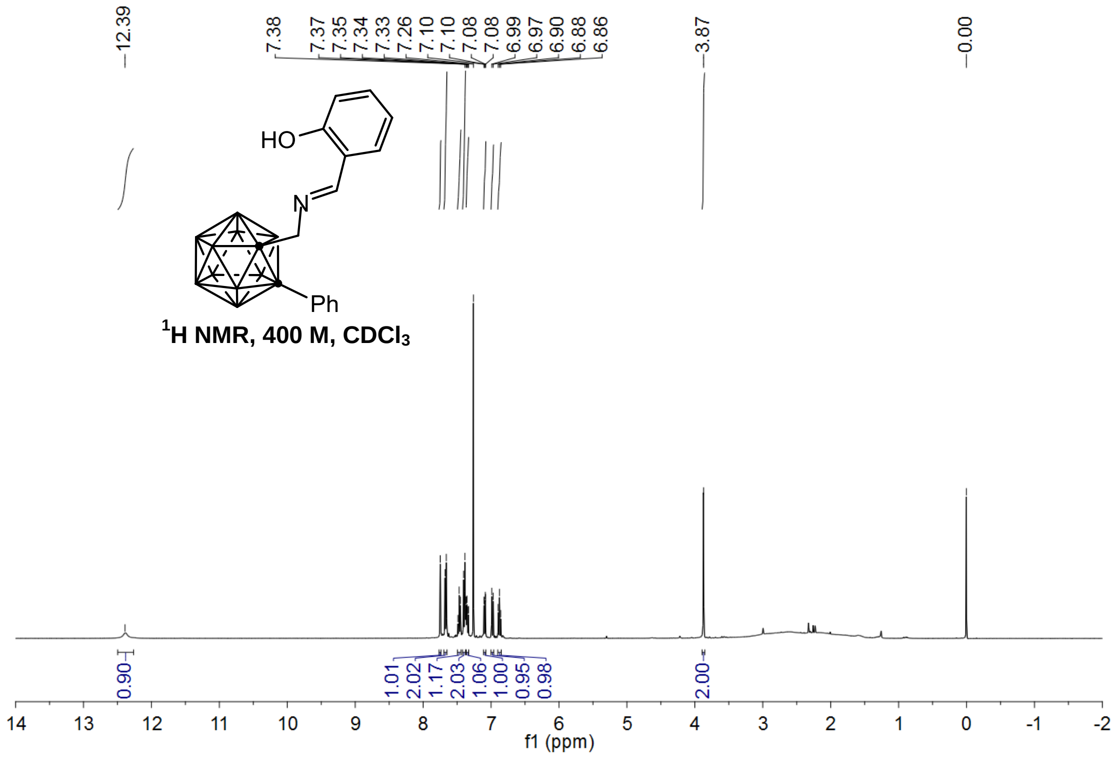
12.39
7.38
7.37
7.35
7.34
7.33
7.26
7.10
7.10
7.08
7.08
6.99
6.97
6.90
6.88
6.86
3.87
0.00
HO
N
Ph
¹H NMR, 400 M, CDCl₃
0.90
1.01
2.02
1.17
2.03
1.06
1.00
0.95
0.98
2.00
f1 (ppm)

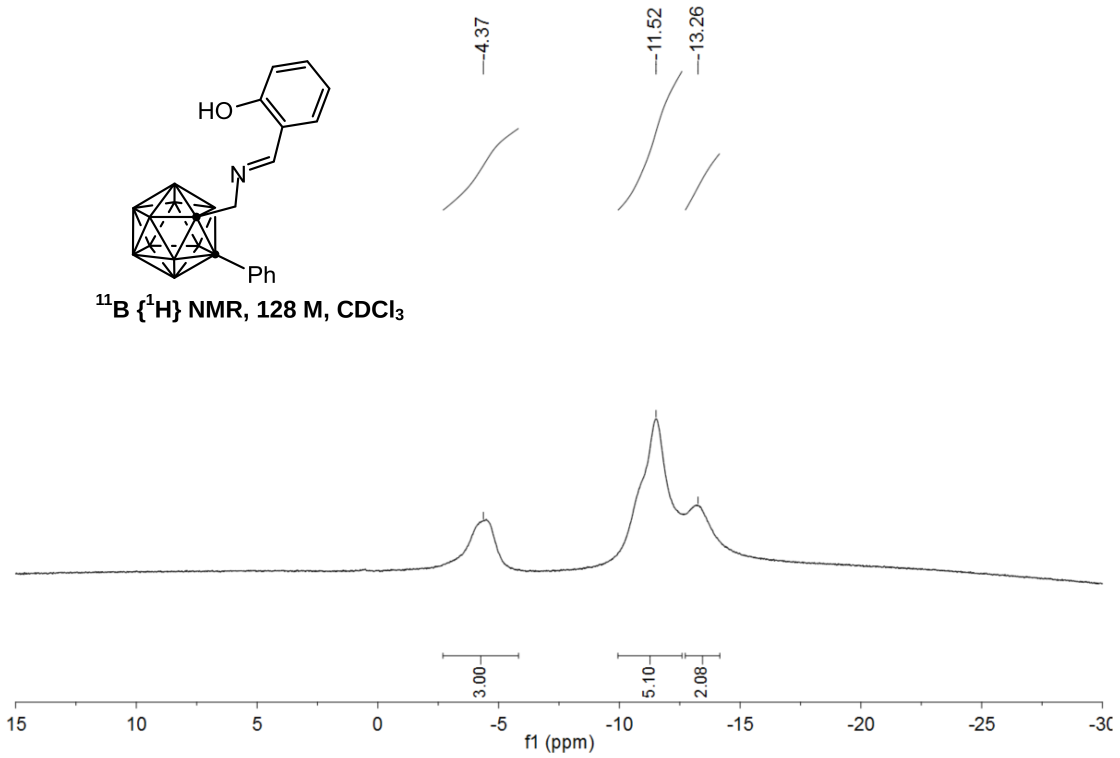
-4.37
-11.52
-13.26
HO
N
Ph
¹¹B {¹H} NMR, 128 M, CDCl₃
3.00
5.10
2.08
f1 (ppm)

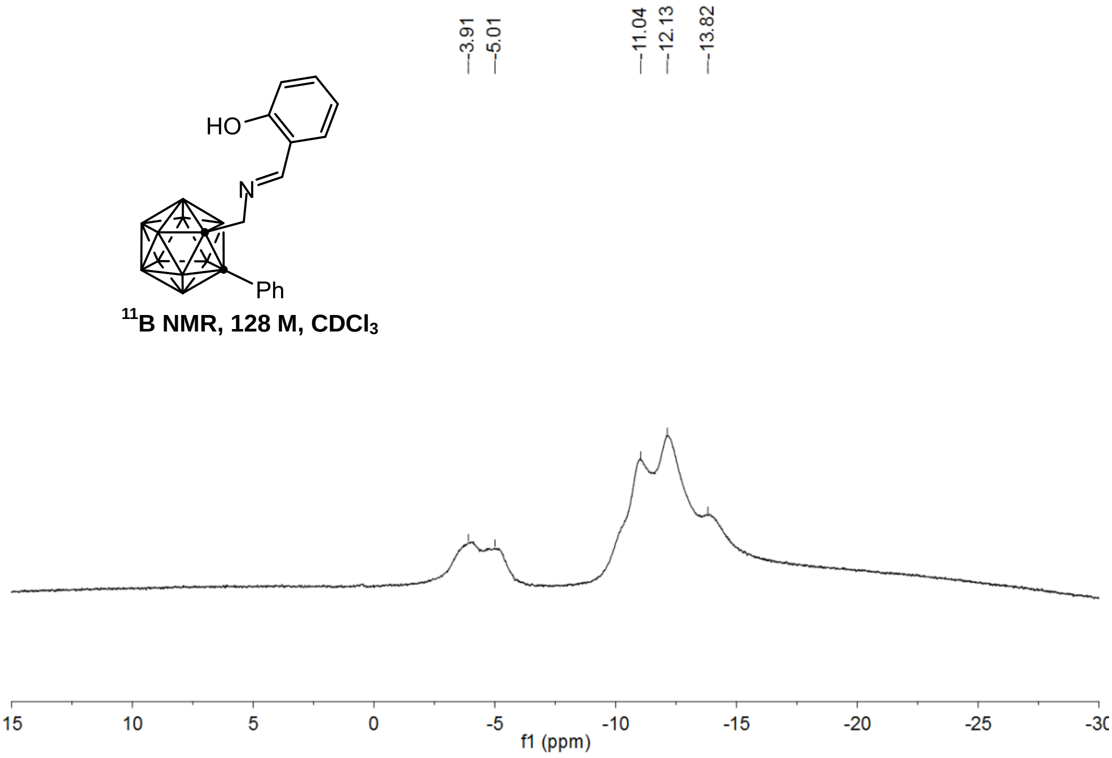

3.91
5.01
11.04
12.13
13.82
HO
N
Ph
^{11}B NMR, 128 M, $CDCl_3$
15
10
5
0
-5
-10
-15
-20
-25
-30
f1 (ppm)

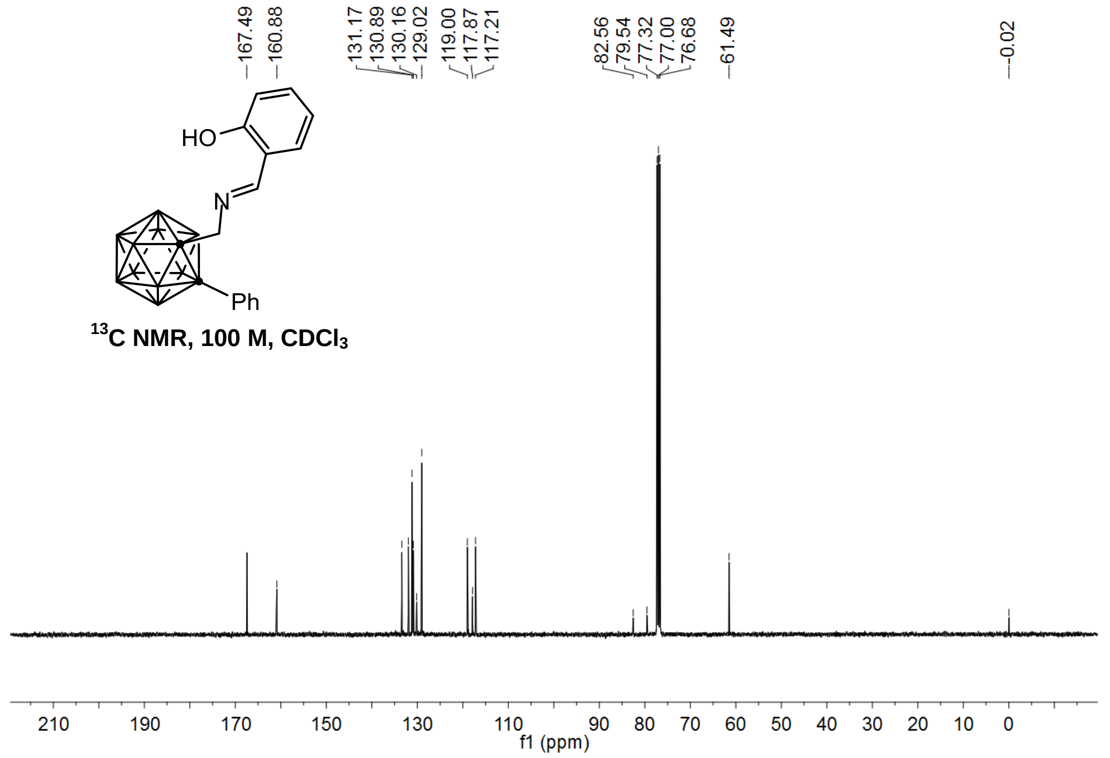

167.49
160.88
131.17
130.89
130.16
129.02
119.00
117.87
117.21
82.56
79.54
77.32
77.00
76.68
61.49
0.02
HO
N
Ph
^{13}C NMR, 100 M, $CDCl_3$
210
190
170
150
130
110
90
80
70
60
50
40
30
20
10
0
f1 (ppm)

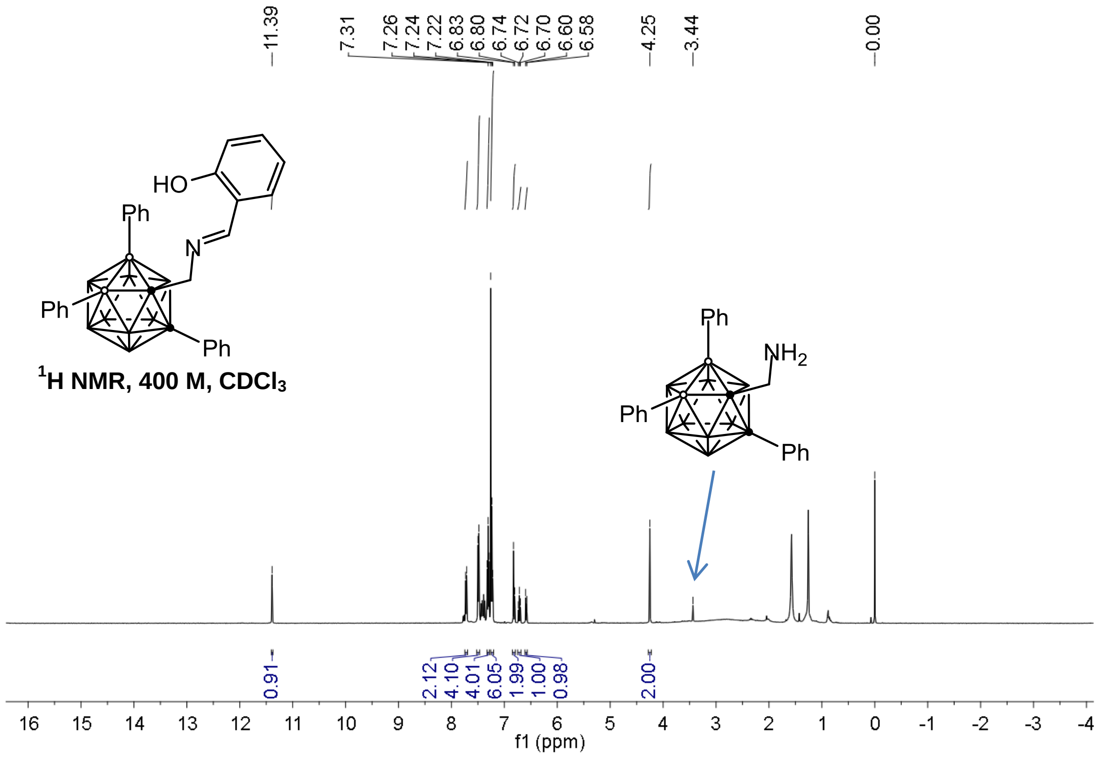
11.39
7.31
7.26
7.24
7.22
6.83
6.80
6.74
6.72
6.70
6.60
6.58
4.25
3.44
0.00
HO
N
Ph
Ph
Ph
^{1}H NMR, 400 M, CDCl$_3$
Ph
NH$_2$
Ph
Ph
0.91
2.12
4.10
4.01
6.05
1.99
1.00
0.98
2.00
f1 (ppm)

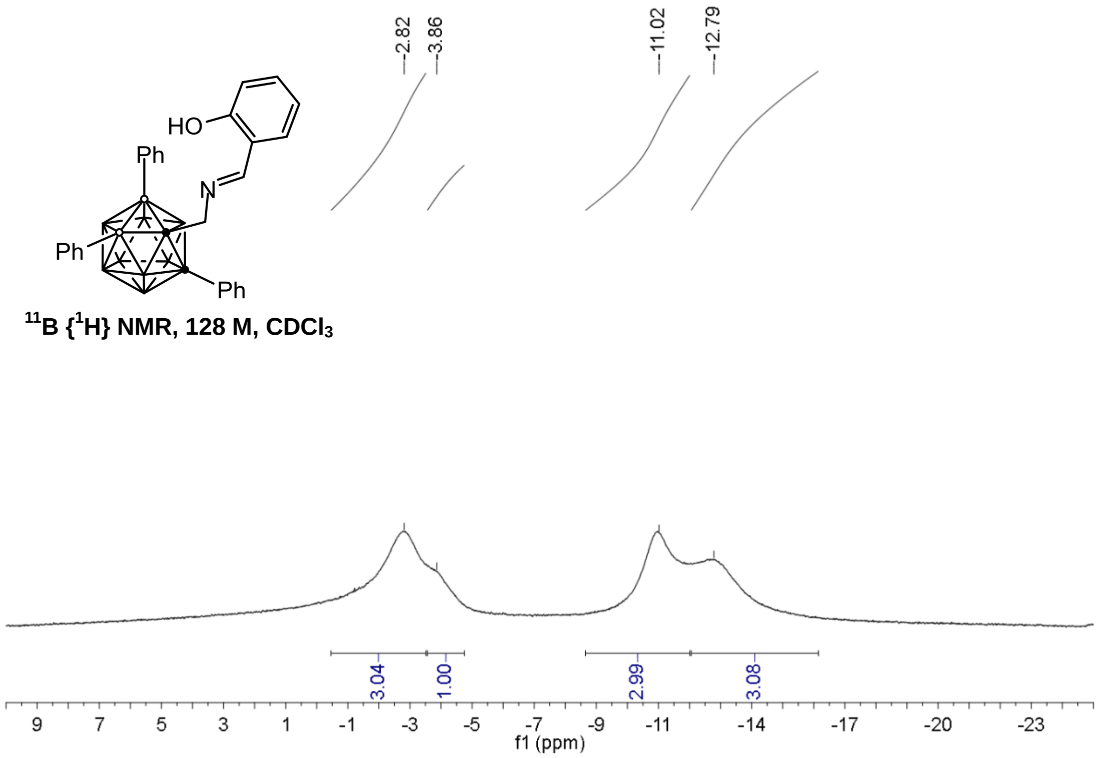
-2.82
-3.86
-11.02
-12.79
HO
N
Ph
Ph
Ph
^{11}B {^{1}H} NMR, 128 M, CDCl$_3$
3.04
1.00
2.99
3.08
f1 (ppm)

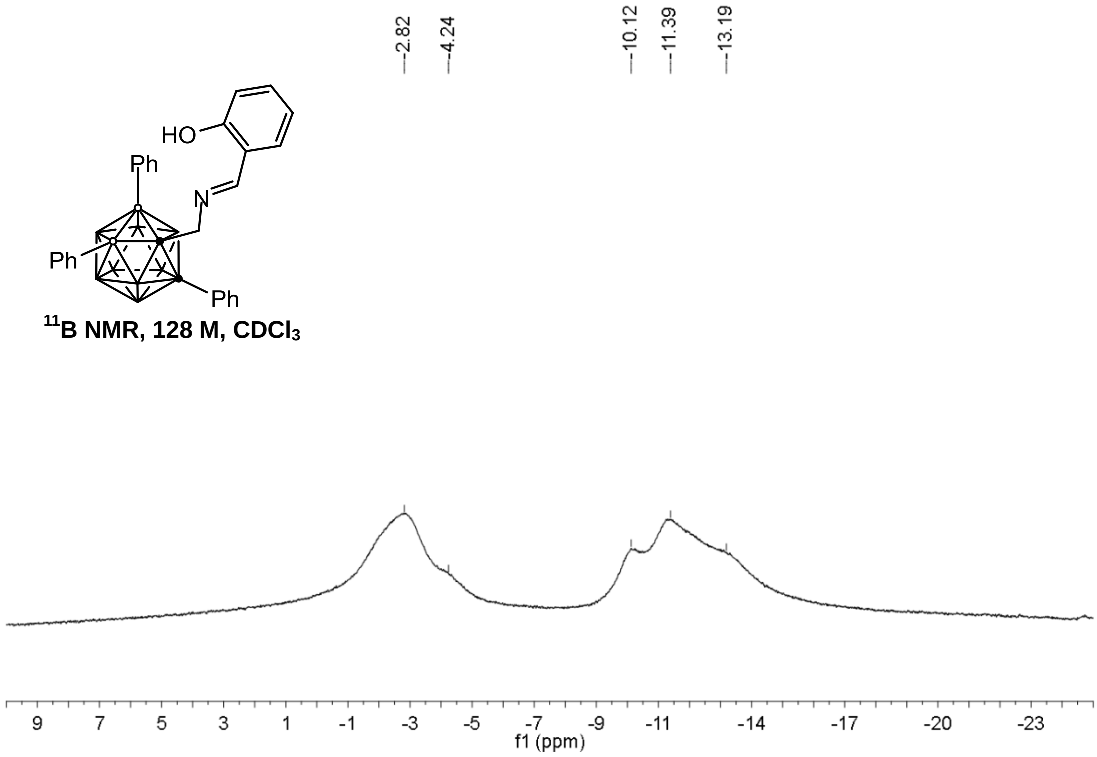
-2.82
-4.24
-10.12
-11.39
-13.19
HO
Ph
N
Ph
Ph
11B NMR, 128 M, CDCl3
9 7 5 3 1 -1 -3 -5 -7 -9 -11 -14 -17 -20 -23
f1 (ppm)

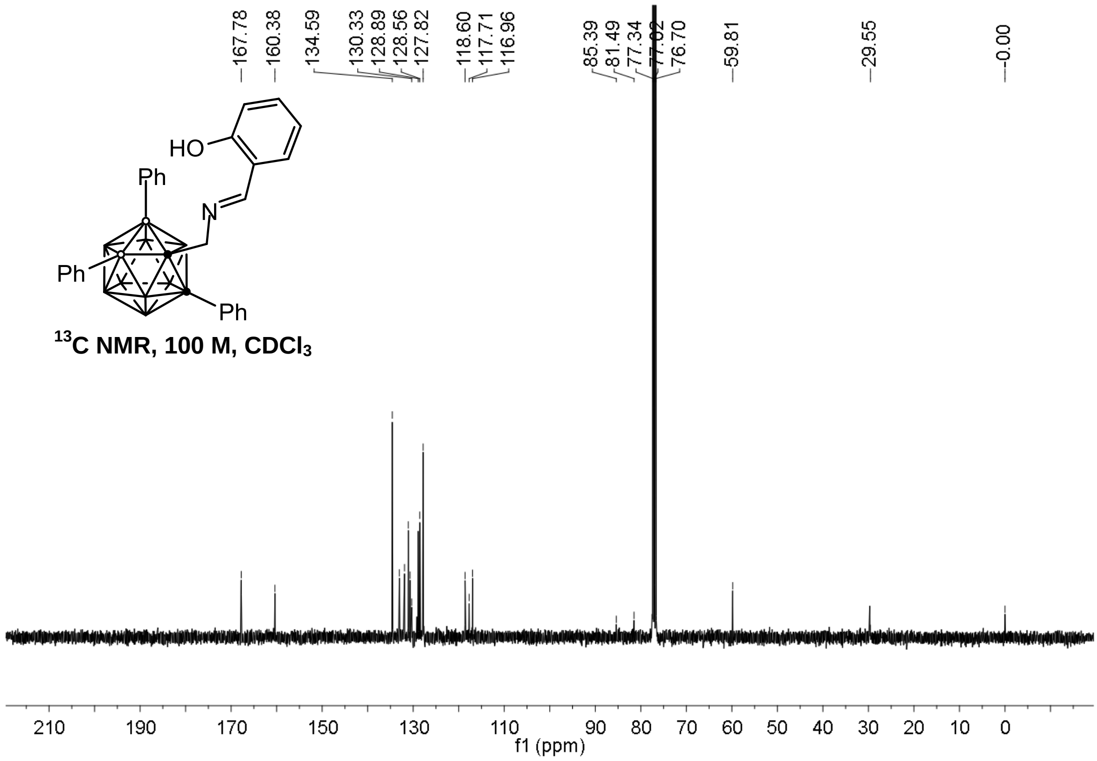
167.78
160.38
134.59
130.33
128.89
128.56
127.82
118.60
117.71
116.96
85.39
81.49
77.34
77.02
76.70
59.81
29.55
0.00
HO
Ph
N
Ph
Ph
13C NMR, 100 M, CDCl3
210 190 170 150 130 110 90 80 70 60 50 40 30 20 10 0
f1 (ppm)

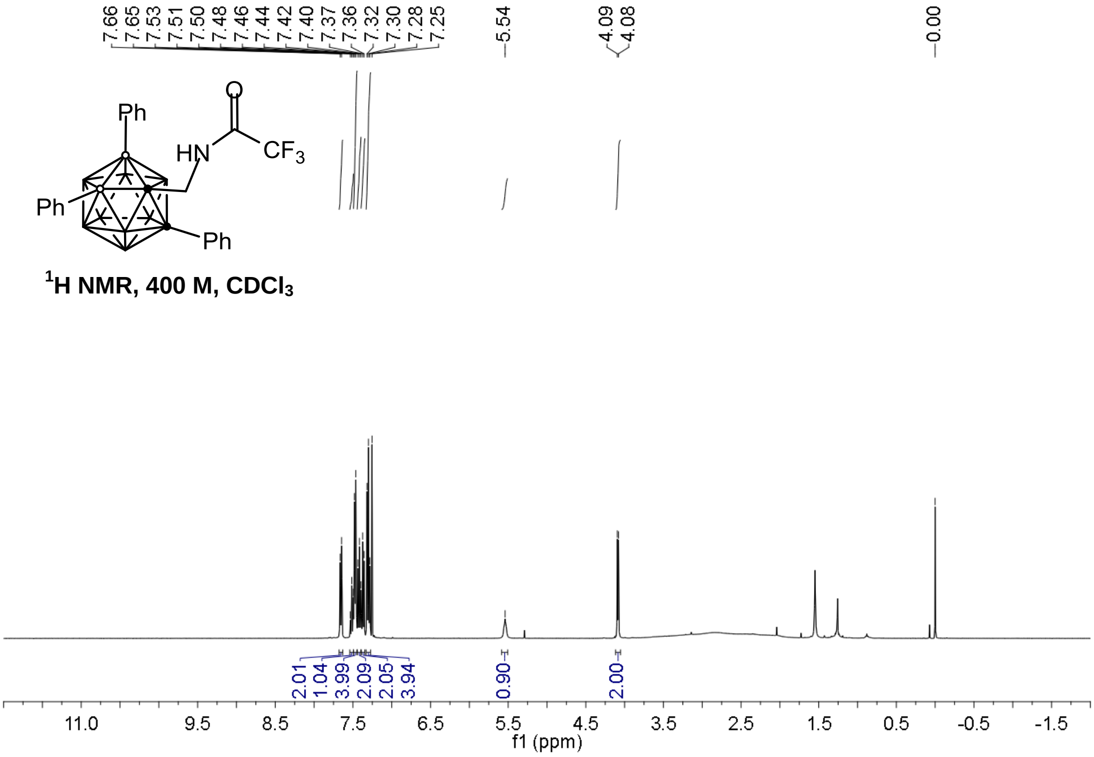
Ph
HN
O
CF3
Ph
Ph
1H NMR, 400 M, CDCl3
f1 (ppm)

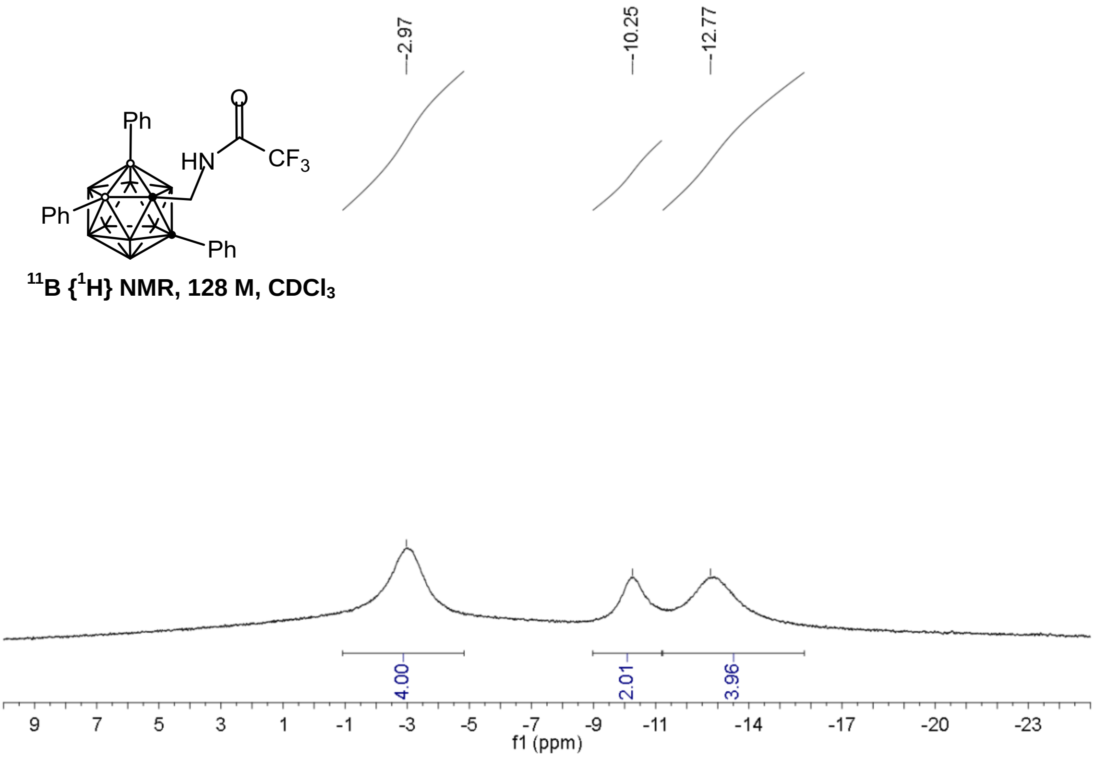
Ph
HN
O
CF3
Ph
Ph
11B {1H} NMR, 128 M, CDCl3
f1 (ppm)

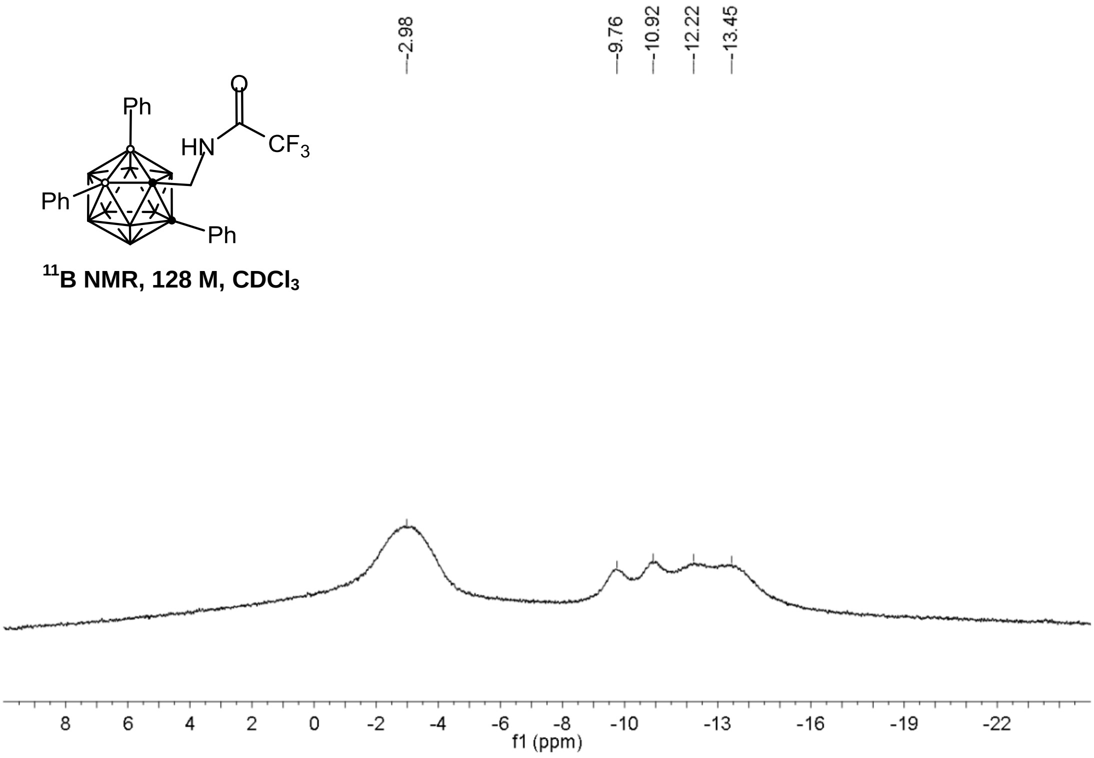
-2.98
-9.76
-10.92
-12.22
-13.45
Ph
HN
CF3
O
Ph
Ph
11B NMR, 128 M, CDCl3
8 6 4 2 0 -2 -4 -6 -8 -10 -13 -16 -19 -22
f1 (ppm)

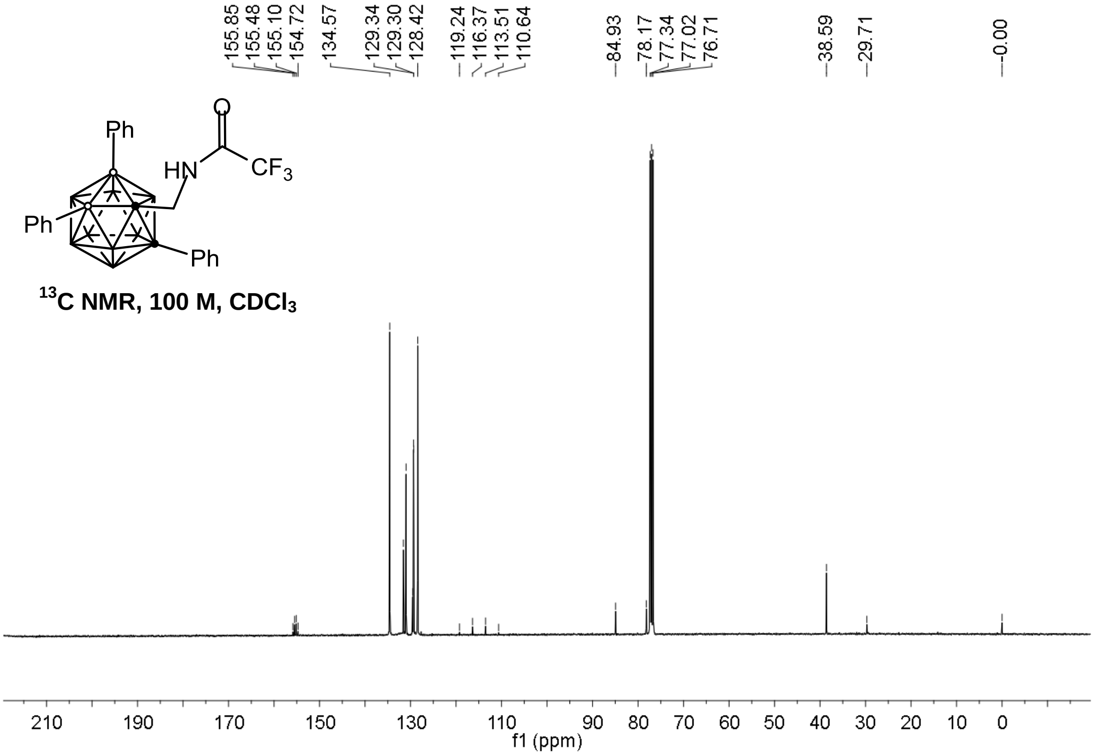
155.85
155.48
155.10
154.72
134.57
129.34
129.30
128.42
119.24
116.37
113.51
110.64
84.93
78.17
77.34
77.02
76.71
38.59
29.71
0.00
Ph
HN
CF3
O
Ph
Ph
13C NMR, 100 M, CDCl3
210 190 170 150 130 110 90 80 70 60 50 40 30 20 10 0
f1 (ppm)

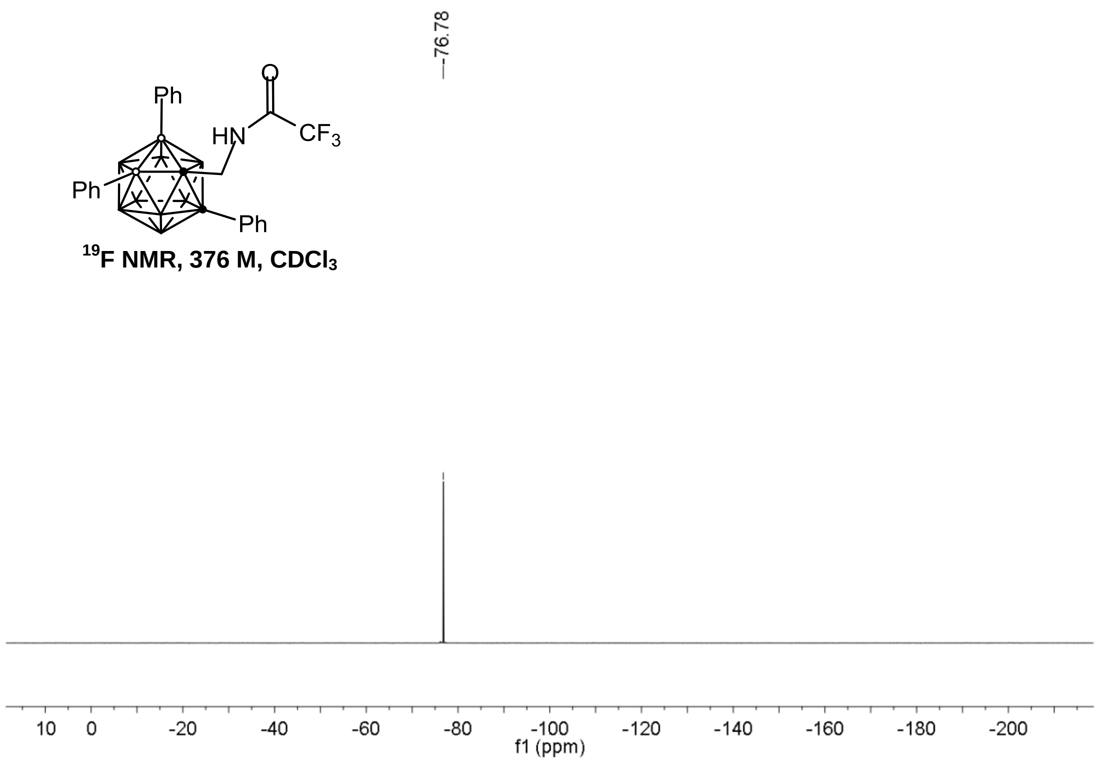

-76.78
Ph
HN
O
CF3
Ph
Ph
19F NMR, 376 M, CDCl3
10 0 -20 -40 -60 -80 -100 -120 -140 -160 -180 -200
f1 (ppm)

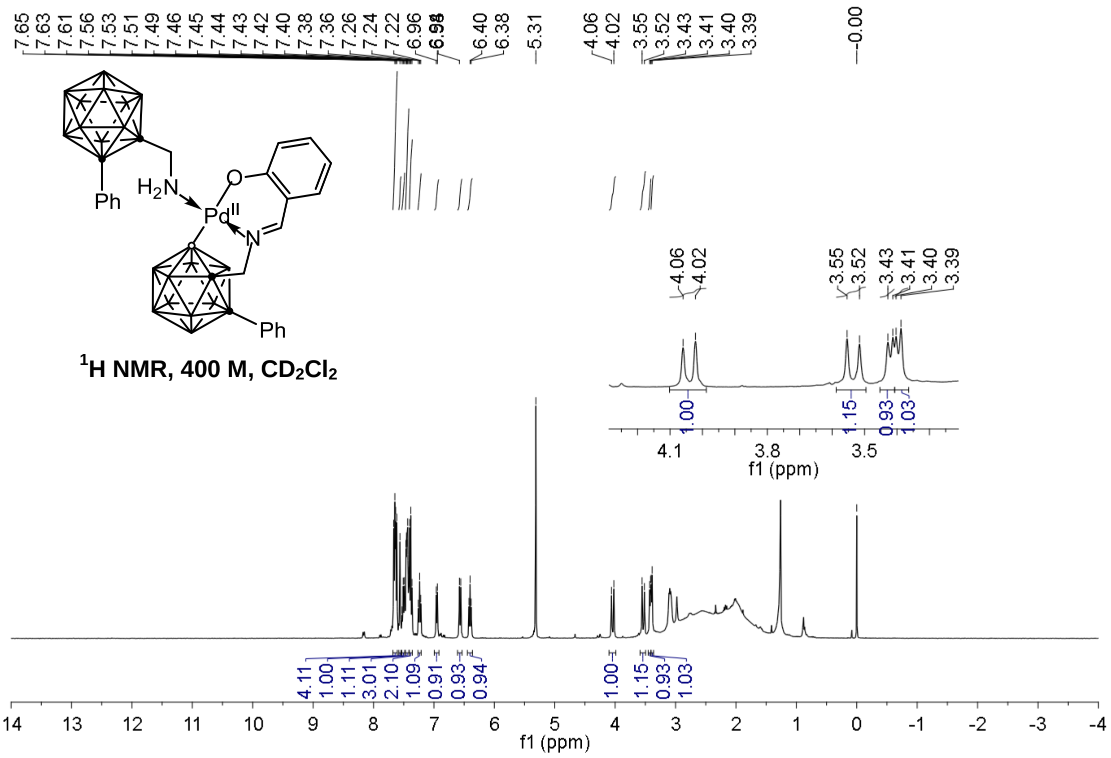

7.65 7.63 7.61 7.56 7.53 7.51 7.49 7.46 7.45 7.44 7.43 7.42 7.40 7.38 7.36 7.26 7.24 7.22 6.96 6.40 6.38 5.31 4.06 4.02 3.55 3.52 3.43 3.41 3.40 3.39 -0.00
H2N
O
PdII
N
Ph
Ph
1H NMR, 400 M, CD2Cl2
4.06 4.02 3.55 3.52 3.43 3.41 3.40 3.39
1.00 1.15 0.93 1.03
4.1 3.8 3.5
f1 (ppm)
4.11 1.00 1.11 3.01 2.10 1.09 0.91 0.93 0.94 1.00 1.15 0.93 1.03
14 13 12 11 10 9 8 7 6 5 4 3 2 1 0 -1 -2 -3 -4
f1 (ppm)

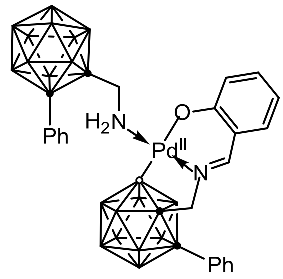

$^{11}B\{^{1}H\}$ NMR, 128 M, CD_2Cl_2

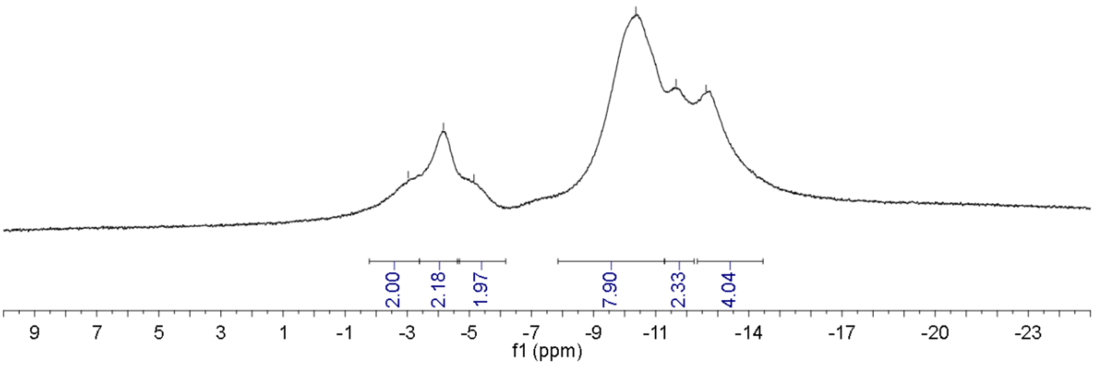

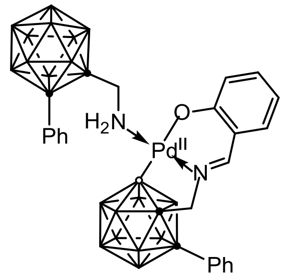

^{11}B NMR, 128 M, CD_2Cl_2

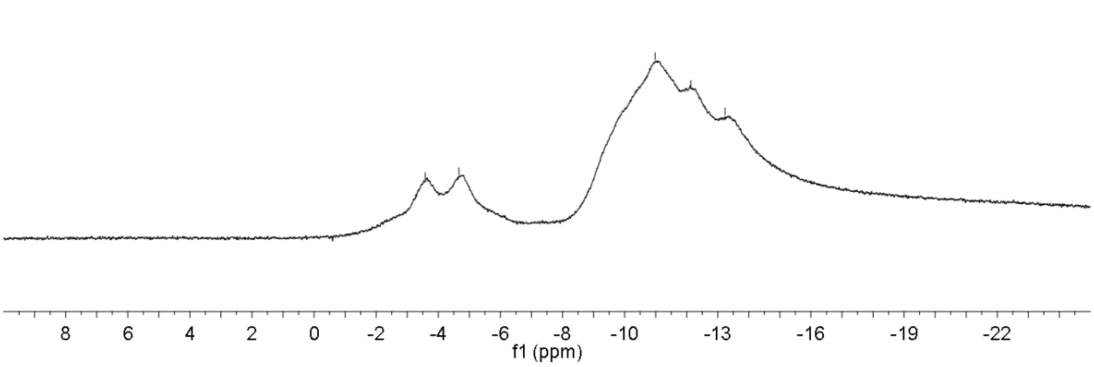

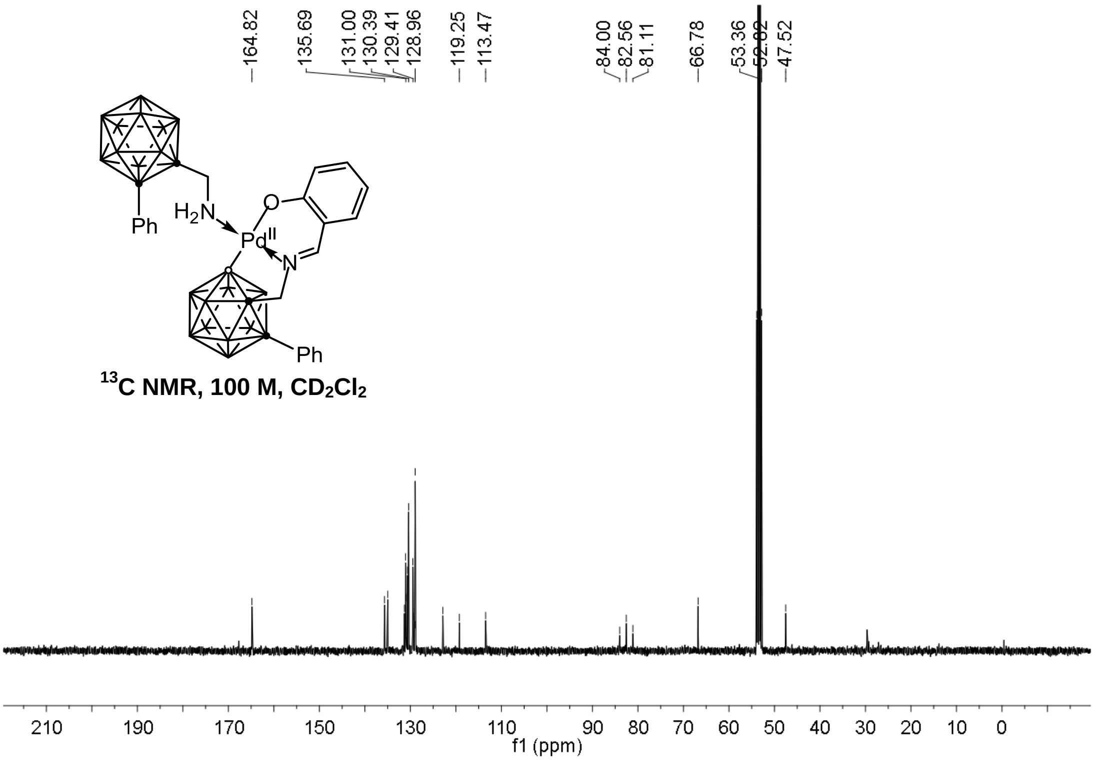
164.82
135.69
131.00
130.39
129.41
128.96
119.25
113.47
84.00
82.56
81.11
66.78
53.36
52.82
47.52
Ph
H2N
O
Pd II
N
Ph
13C NMR, 100 M, CD2Cl2
210 190 170 150 130 110 90 80 70 60 50 40 30 20 10 0
f1 (ppm)

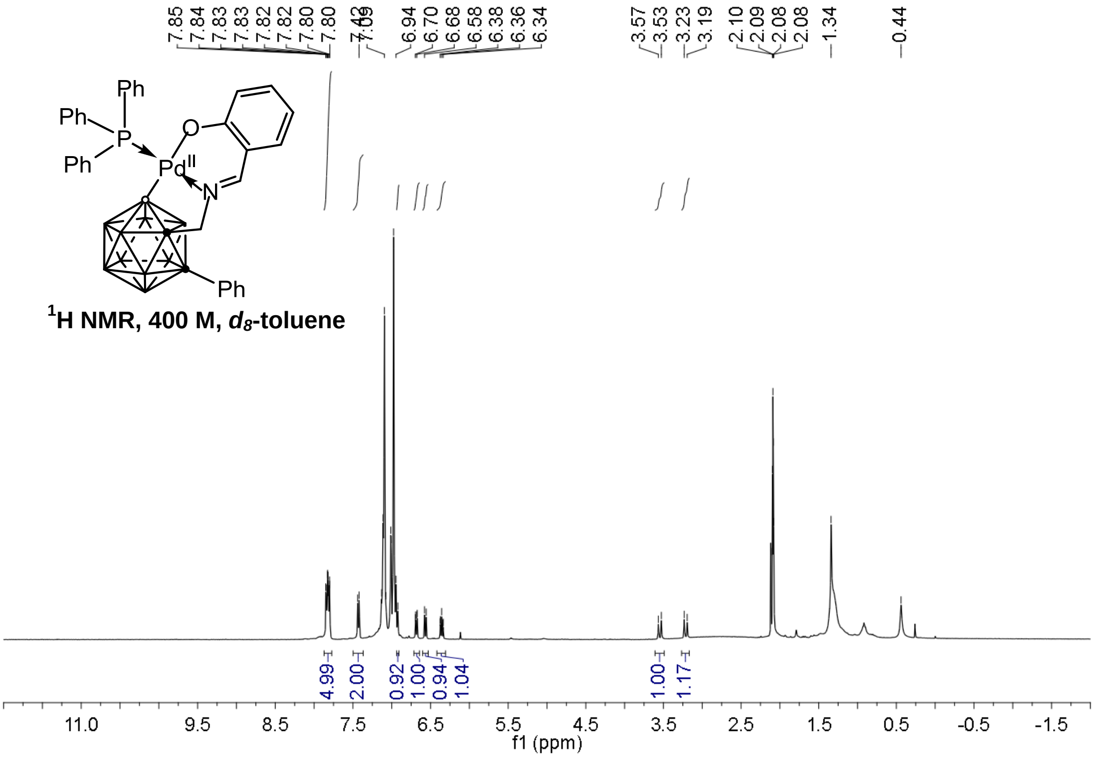
7.85
7.84
7.83
7.83
7.82
7.82
7.80
7.80
7.42
7.09
6.94
6.70
6.68
6.58
6.38
6.36
6.34
3.57
3.53
3.23
3.19
2.10
2.09
2.08
2.08
1.34
0.44
Ph
Ph
P
Ph
O
Pd II
N
Ph
1H NMR, 400 M, d8-toluene
4.99
2.00
0.92
1.00
0.94
1.04
1.00
1.17
11.0 9.5 8.5 7.5 6.5 5.5 4.5 3.5 2.5 1.5 0.5 -0.5 -1.5
f1 (ppm)

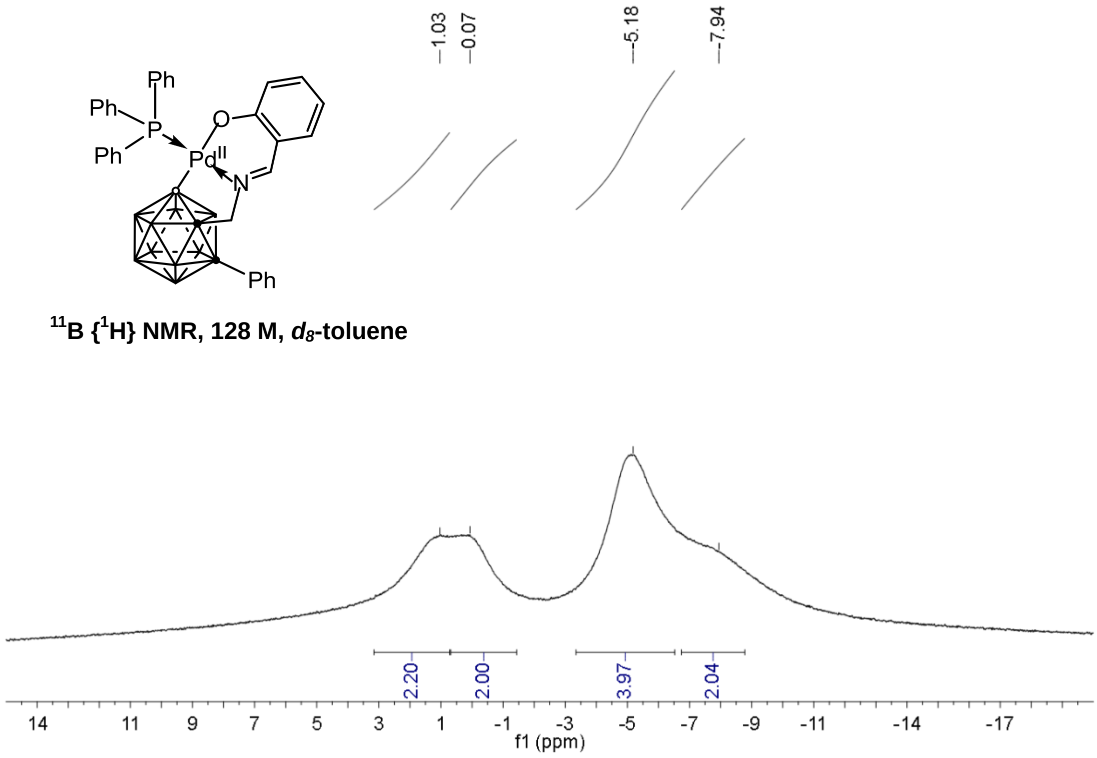

$^{11}B\ \{^{1}H\}$ NMR, 128 M, d_8-toluene

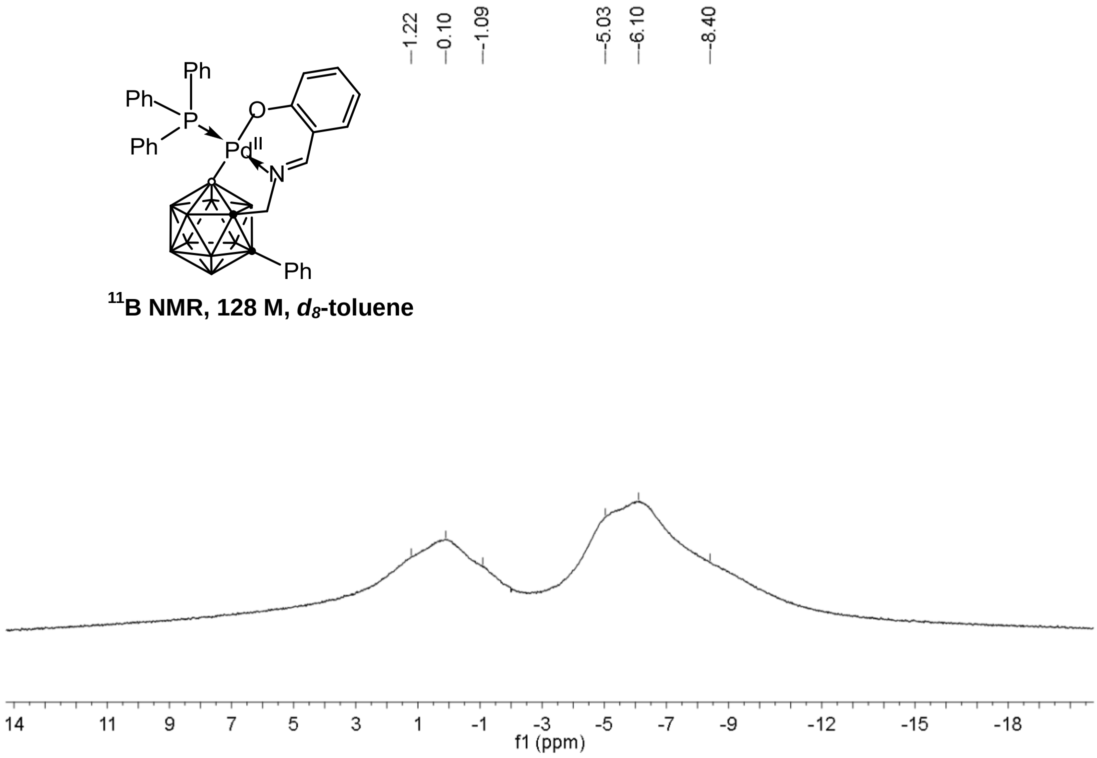

^{11}B NMR, 128 M, d_8-toluene

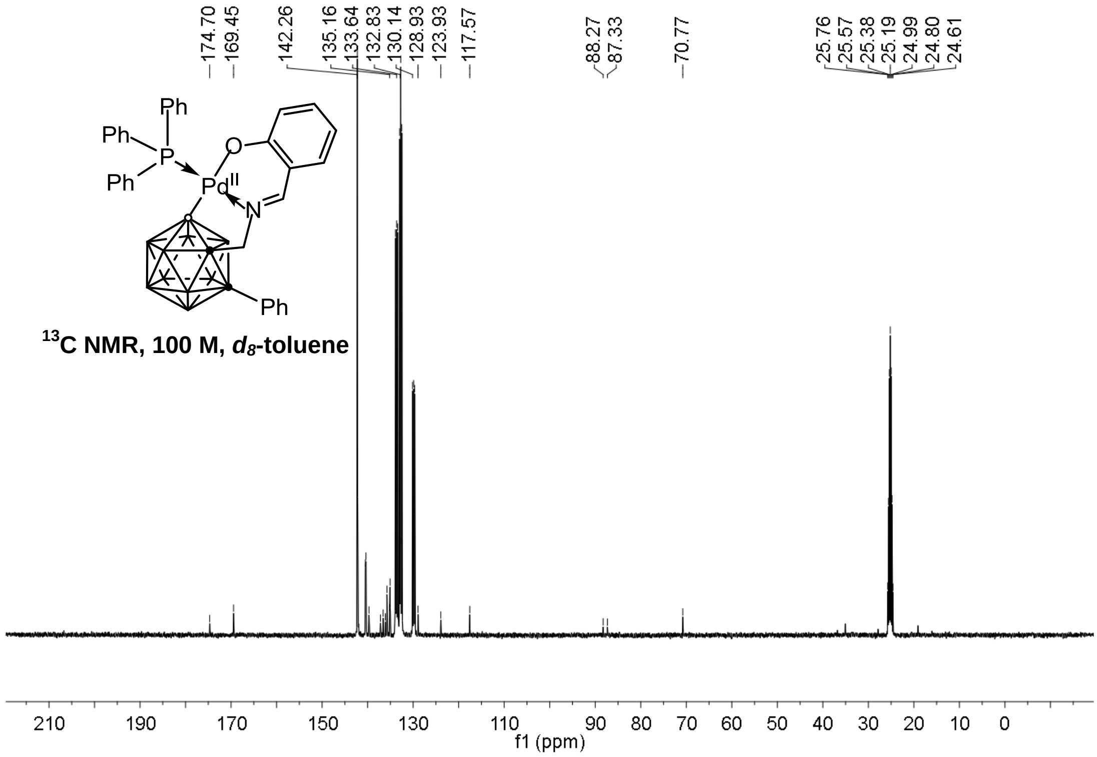

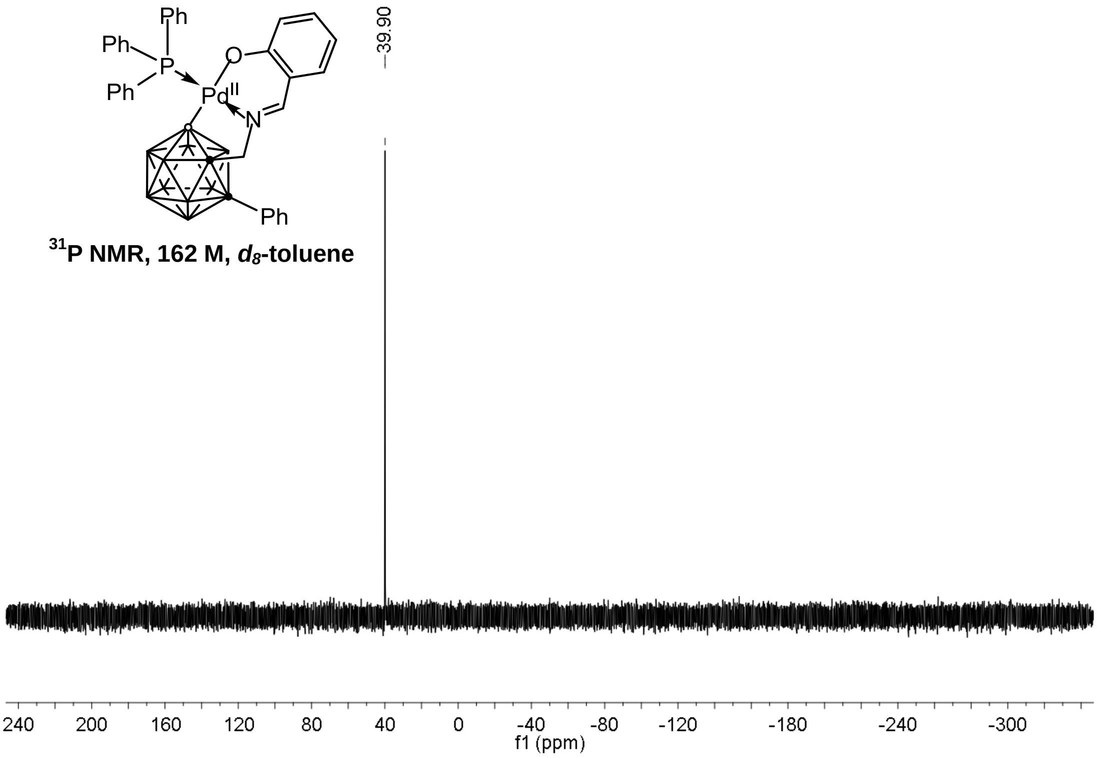

5.4 Further transformation

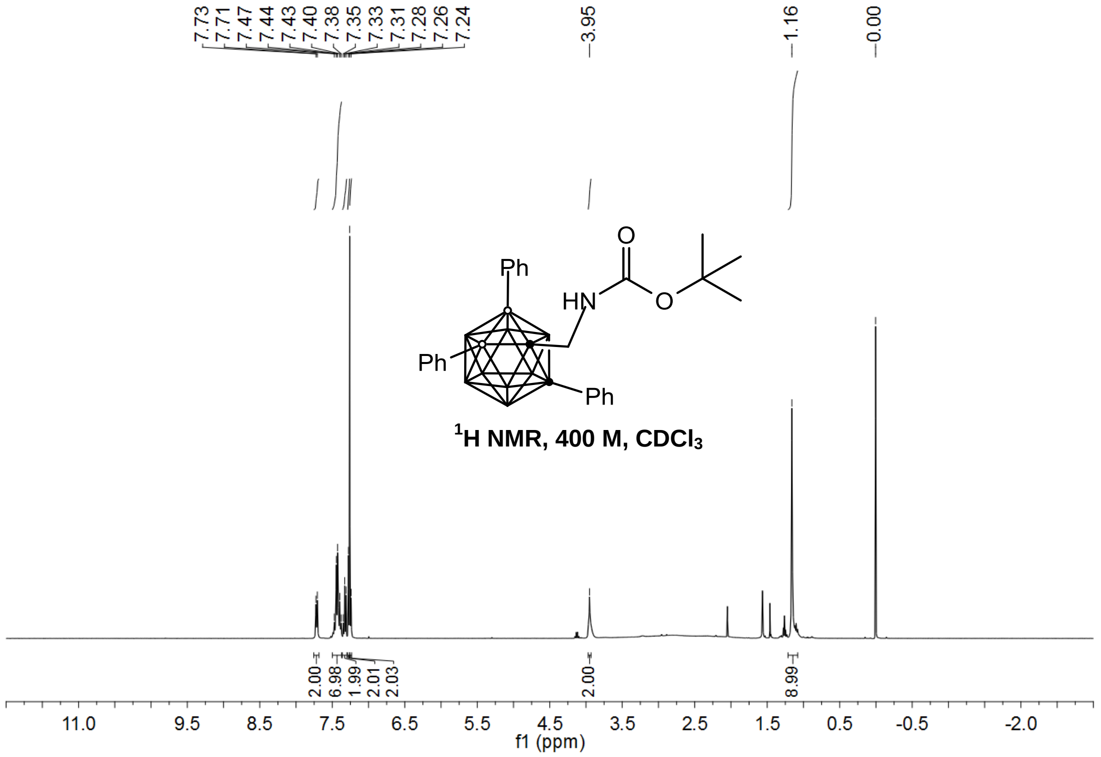
7.73
7.71
7.47
7.44
7.43
7.40
7.38
7.35
7.33
7.31
7.28
7.26
7.24
3.95
1.16
0.00
Ph
HN
O
O
Ph
Ph
1H NMR, 400 M, CDCl3
2.00
6.98
1.99
2.01
2.03
2.00
8.99
11.0
9.5
8.5
7.5
6.5
5.5
4.5
3.5
2.5
1.5
0.5
-0.5
-2.0
f1 (ppm)

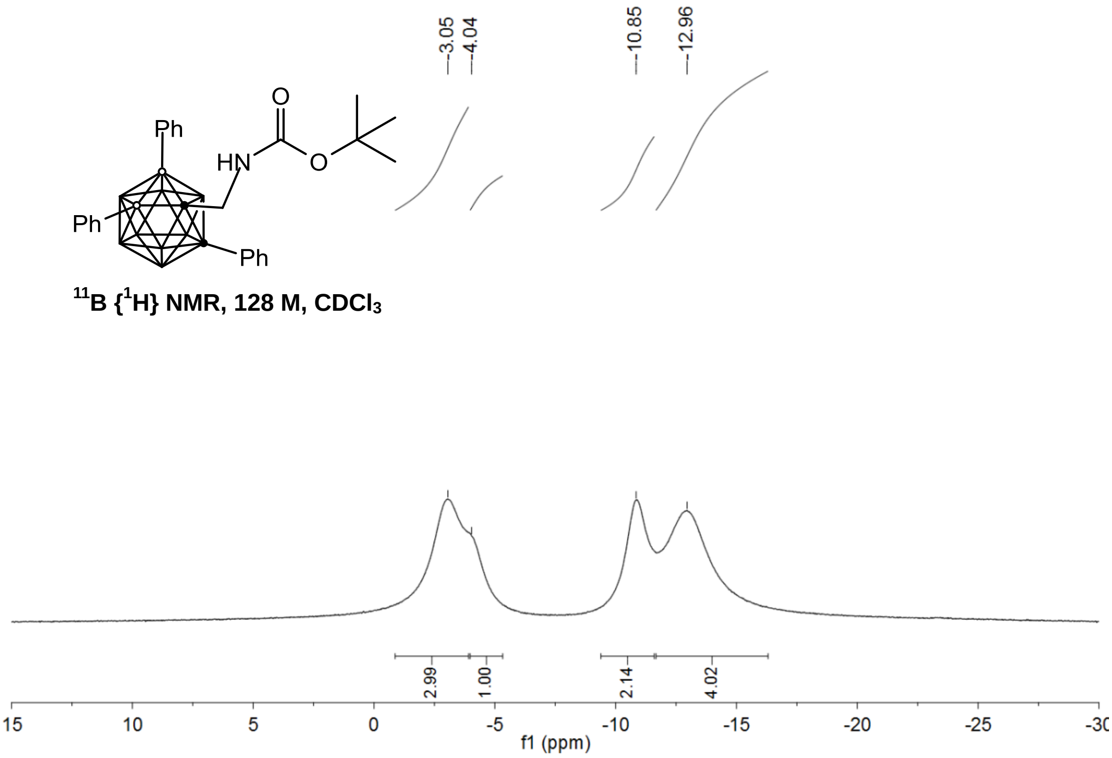
-3.05
-4.04
-10.85
-12.96
Ph
HN
O
O
Ph
Ph
11B {1H} NMR, 128 M, CDCl3
2.99
1.00
2.14
4.02
15
10
5
0
-5
-10
-15
-20
-25
-30
f1 (ppm)

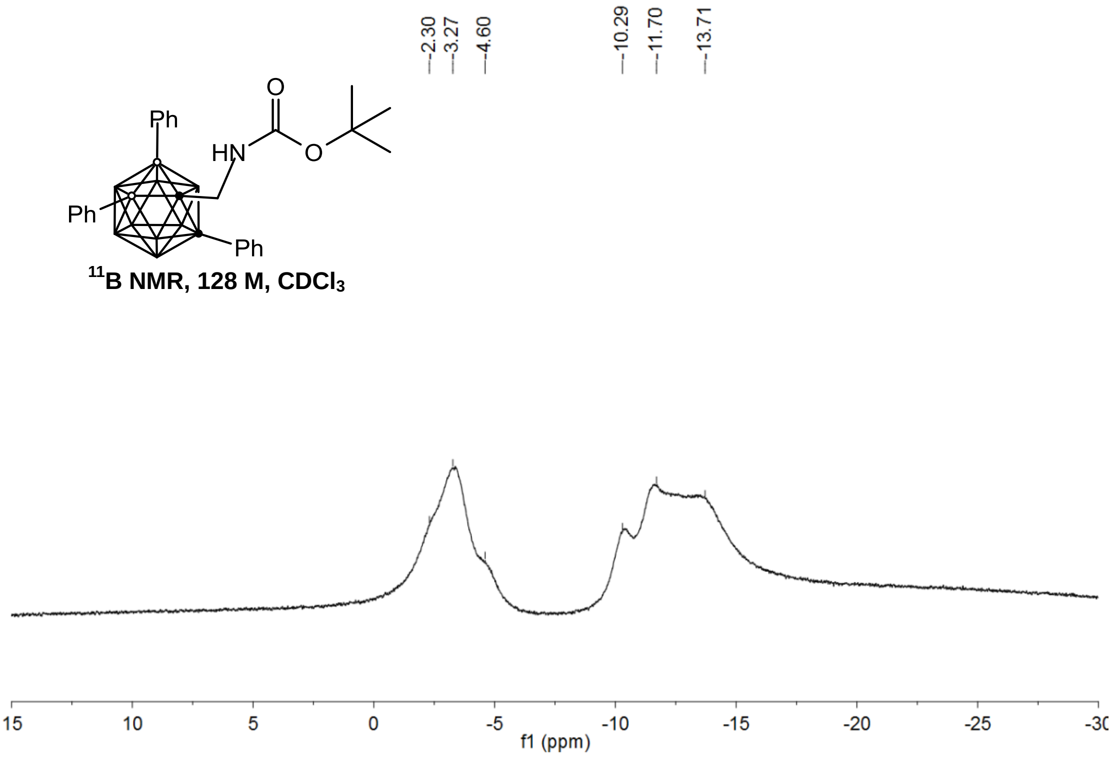

2.30
3.27
4.60
10.29
11.70
13.71
O
Ph
HN
O
Ph
Ph
^{11}B NMR, 128 M, CDCl$_3$
15
10
5
0
-5
-10
-15
-20
-25
f1 (ppm)

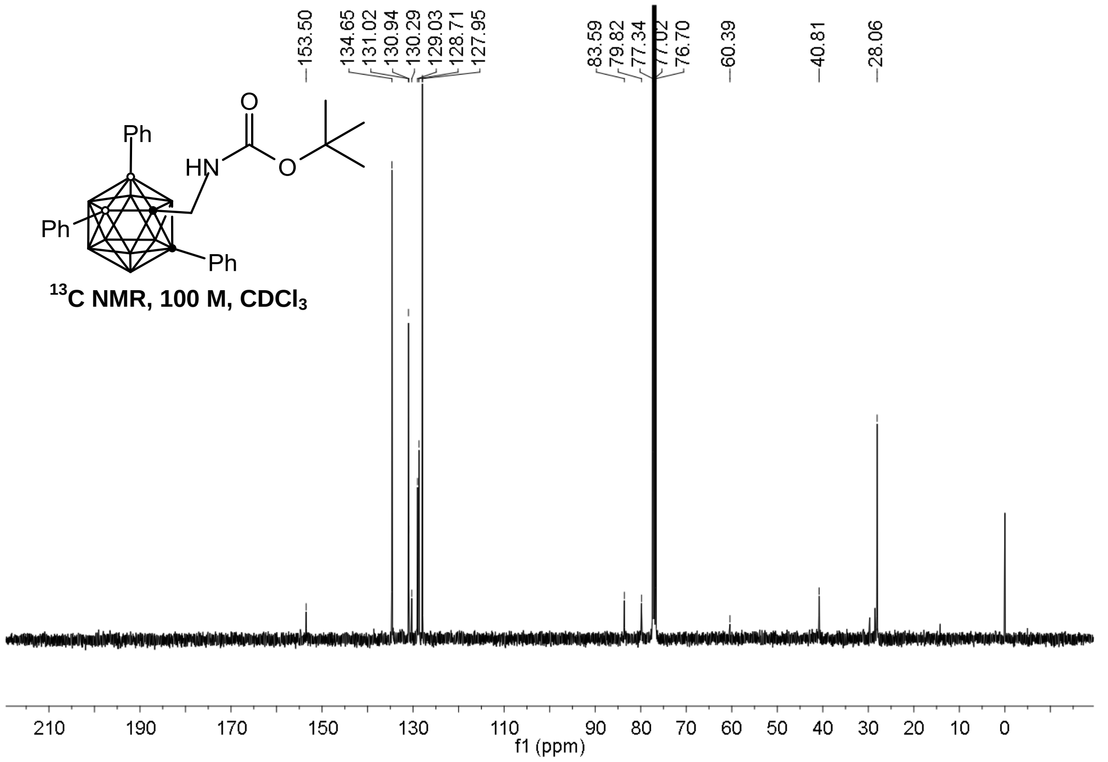

153.50
134.65
131.02
130.94
130.29
129.03
128.71
127.95
83.59
79.82
77.34
77.02
76.70
60.39
40.81
28.06
O
Ph
HN
O
Ph
Ph
^{13}C NMR, 100 M, CDCl$_3$
210
190
170
150
130
110
90
80
70
60
50
40
30
20
10
0
f1 (ppm)

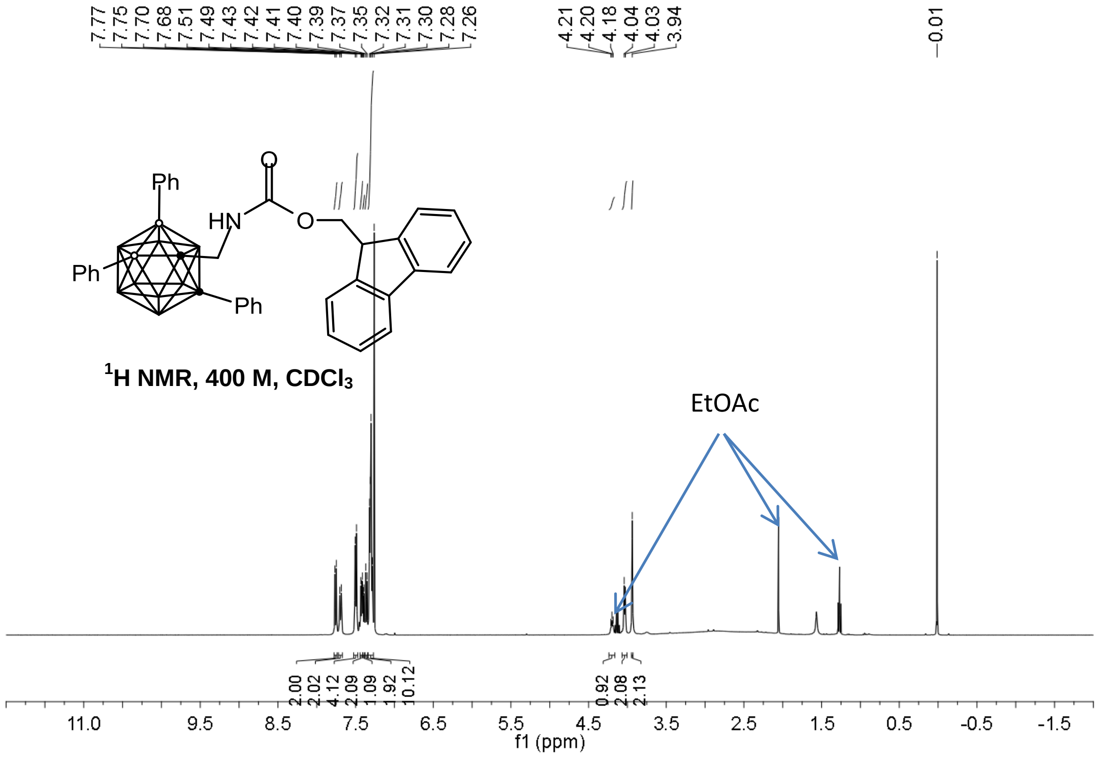
7.77 7.75 7.70 7.68 7.51 7.49 7.43 7.42 7.41 7.40 7.39 7.37 7.35 7.32 7.31 7.30 7.28 7.26
4.21 4.20 4.18 4.04 4.03 3.94
-0.01
Ph
HN
O
Ph
Ph
^{1}H NMR, 400 M, CDCl$_3$
EtOAc
2.00 2.02 4.12 2.09 1.09 1.92 10.12
0.92 2.08 2.13
11.0 9.5 8.5 7.5 6.5 5.5 4.5 3.5 2.5 1.5 0.5 -0.5 -1.5
f1 (ppm)

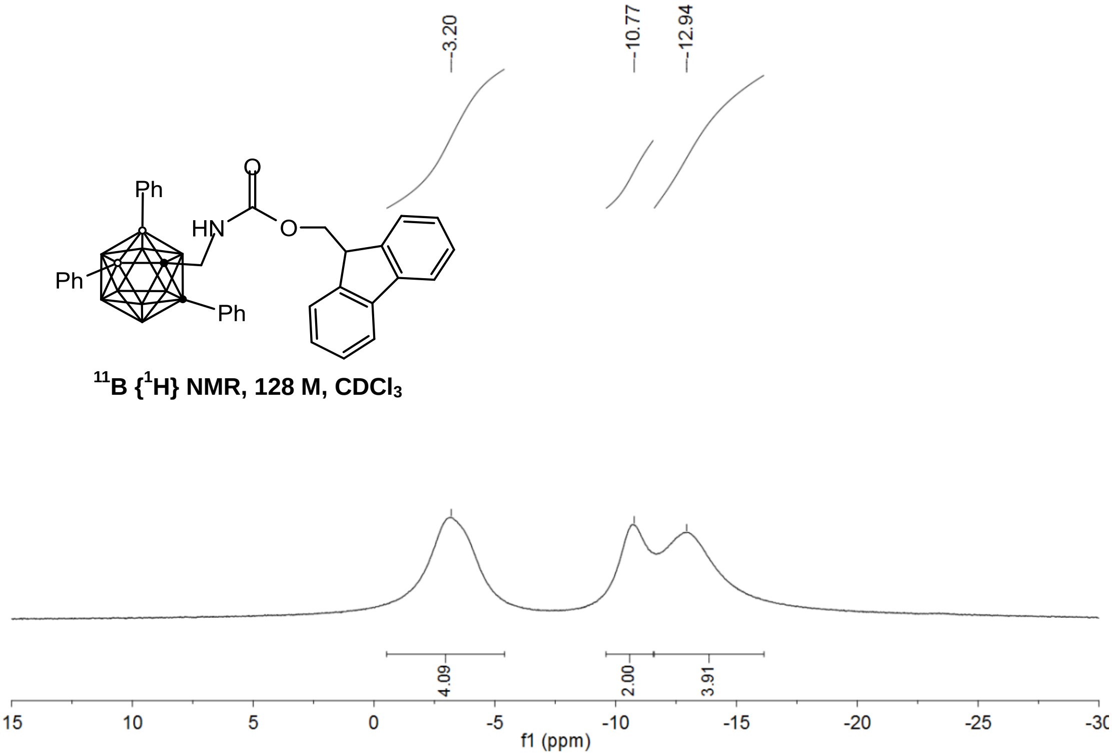
-3.20
-10.77
-12.94
Ph
HN
O
Ph
Ph
^{11}B {^{1}H} NMR, 128 M, CDCl$_3$
4.09
2.00
3.91
15 10 5 0 -5 -10 -15 -20 -25 -30
f1 (ppm)

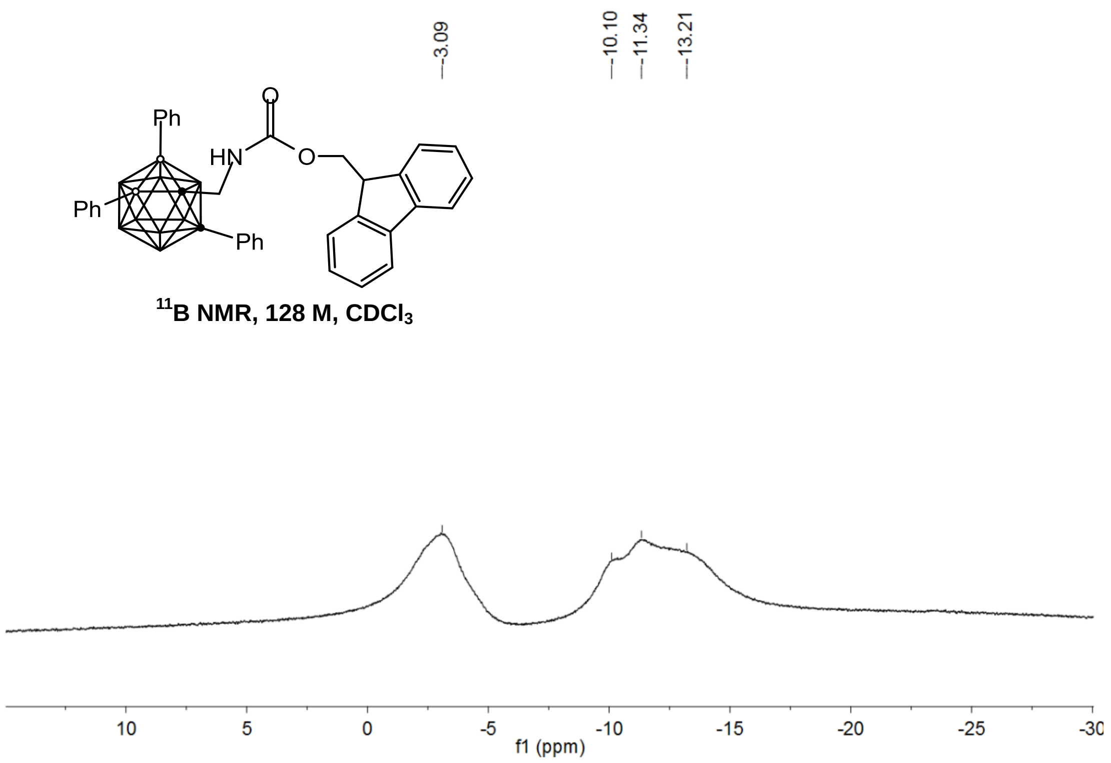
-3.09
-10.10
-11.34
-13.21
Ph
HN
O
O
Ph
Ph
11B NMR, 128 M, CDCl3
10
5
0
-5
-10
-15
-20
-25
-30
f1 (ppm)

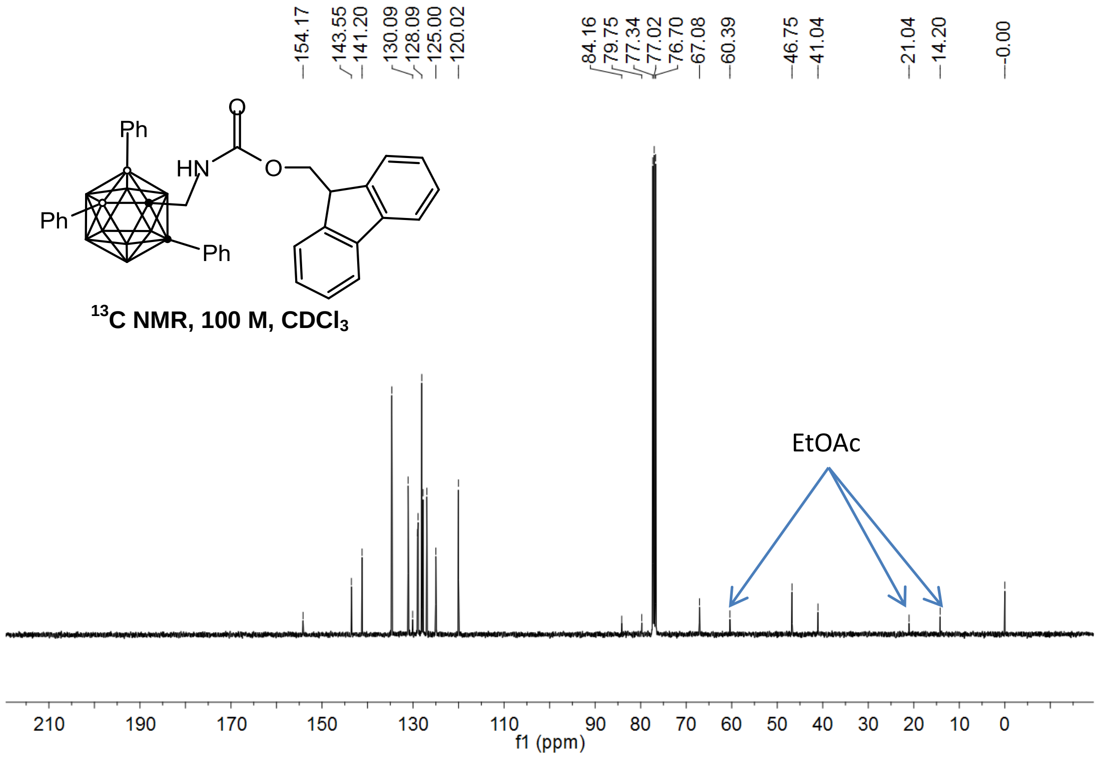
154.17
143.55
141.20
130.09
128.09
125.00
120.02
84.16
79.75
77.34
77.02
76.70
67.08
60.39
46.75
41.04
21.04
14.20
0.00
Ph
HN
O
O
Ph
Ph
13C NMR, 100 M, CDCl3
EtOAc
210
190
170
150
130
110
90
80
70
60
50
40
30
20
10
0
f1 (ppm)

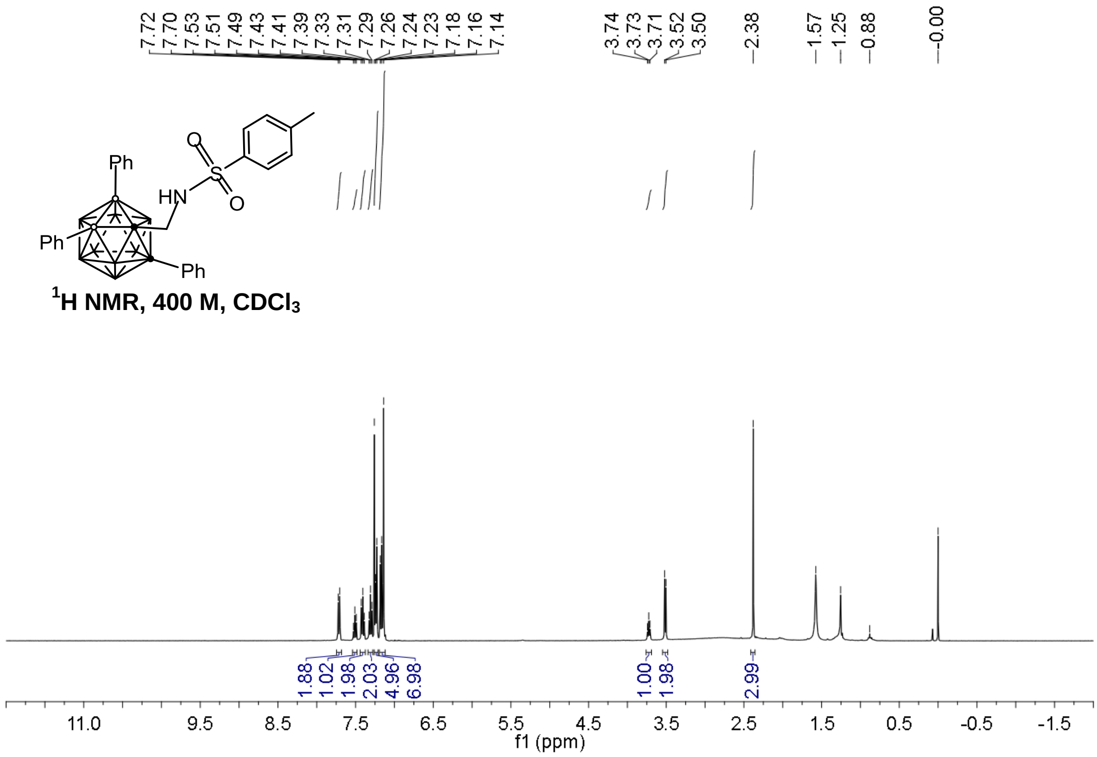
7.72
7.70
7.53
7.51
7.49
7.43
7.41
7.39
7.33
7.31
7.29
7.26
7.24
7.23
7.18
7.16
7.14
3.74
3.73
3.71
3.52
3.50
2.38
1.57
1.25
0.88
0.00
Ph
HN
S
O
O
Ph
Ph
1H NMR, 400 M, CDCl3
1.88
1.02
1.98
2.03
4.96
6.98
1.00
1.98
2.99
11.0
9.5
8.5
7.5
6.5
5.5
4.5
3.5
2.5
1.5
0.5
-0.5
-1.5
f1 (ppm)

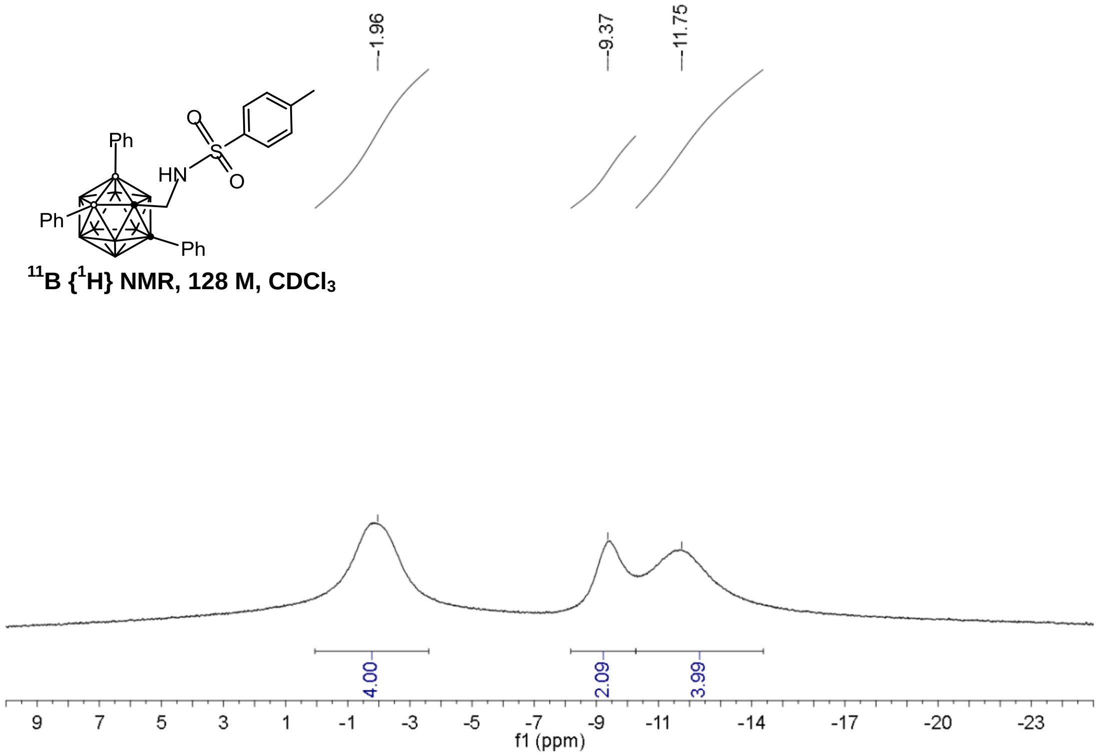
-1.96
-9.37
-11.75
Ph
HN
S
O
O
Ph
Ph
11B {1H} NMR, 128 M, CDCl3
4.00
2.09
3.99
9
7
5
3
1
-1
-3
-5
-7
-9
-11
-14
-17
-20
-23
f1 (ppm)

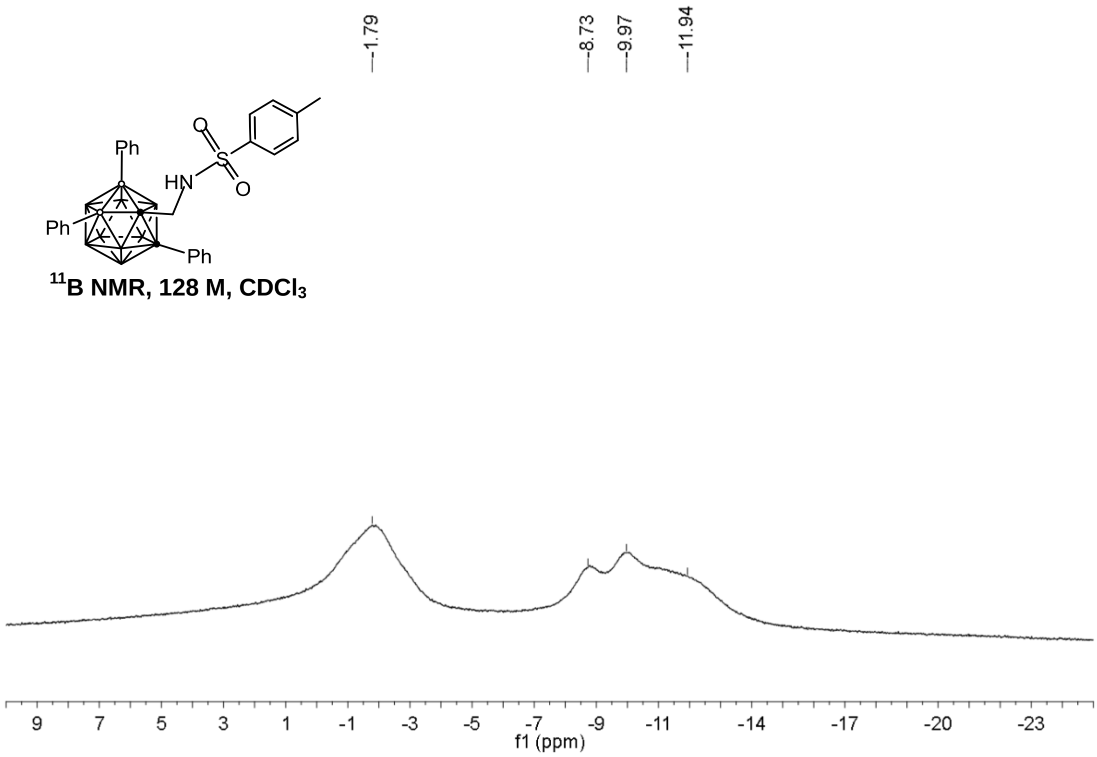

-1.79
-8.73
-9.97
-11.94
Ph
HN
O
S
O
Ph
Ph
^{11}B NMR, 128 M, $CDCl_3$
9 7 5 3 1 -1 -3 -5 -7 -9 -11 -14 -17 -20 -23
f1 (ppm)

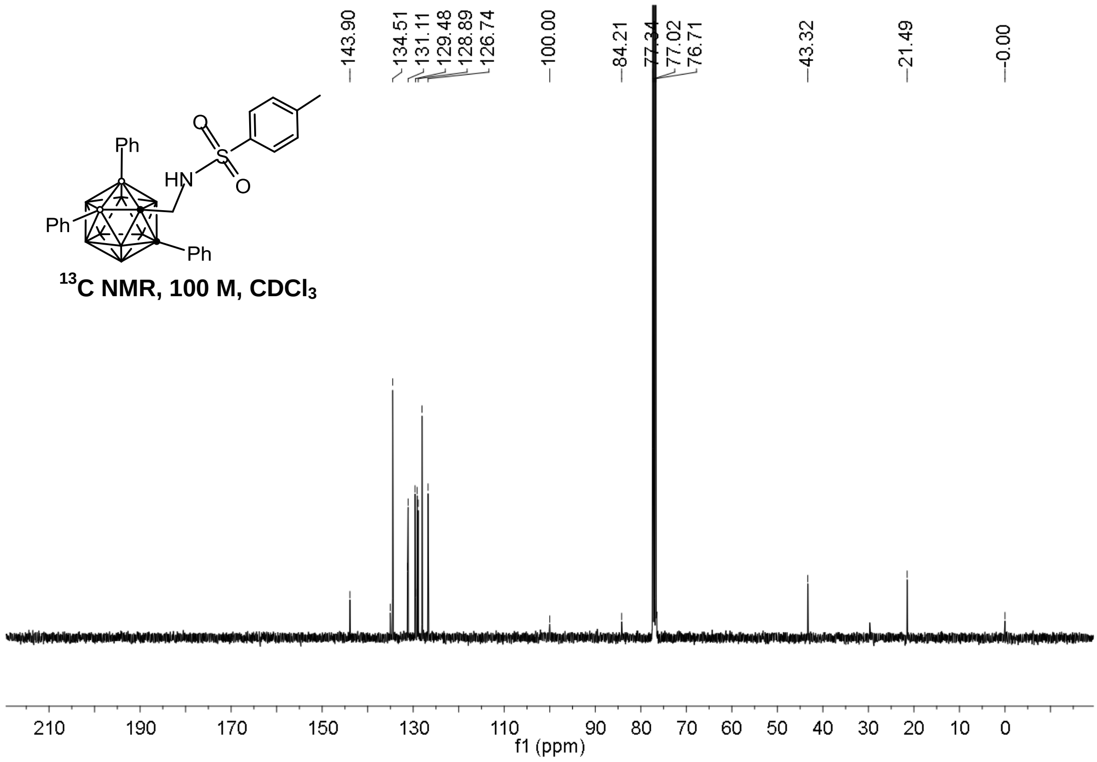

143.90
134.51
131.11
129.48
128.89
126.74
100.00
84.21
77.34
77.02
76.71
43.32
21.49
0.00
Ph
HN
O
S
O
Ph
Ph
^{13}C NMR, 100 M, $CDCl_3$
210 190 170 150 130 110 90 80 70 60 50 40 30 20 10 0
f1 (ppm)

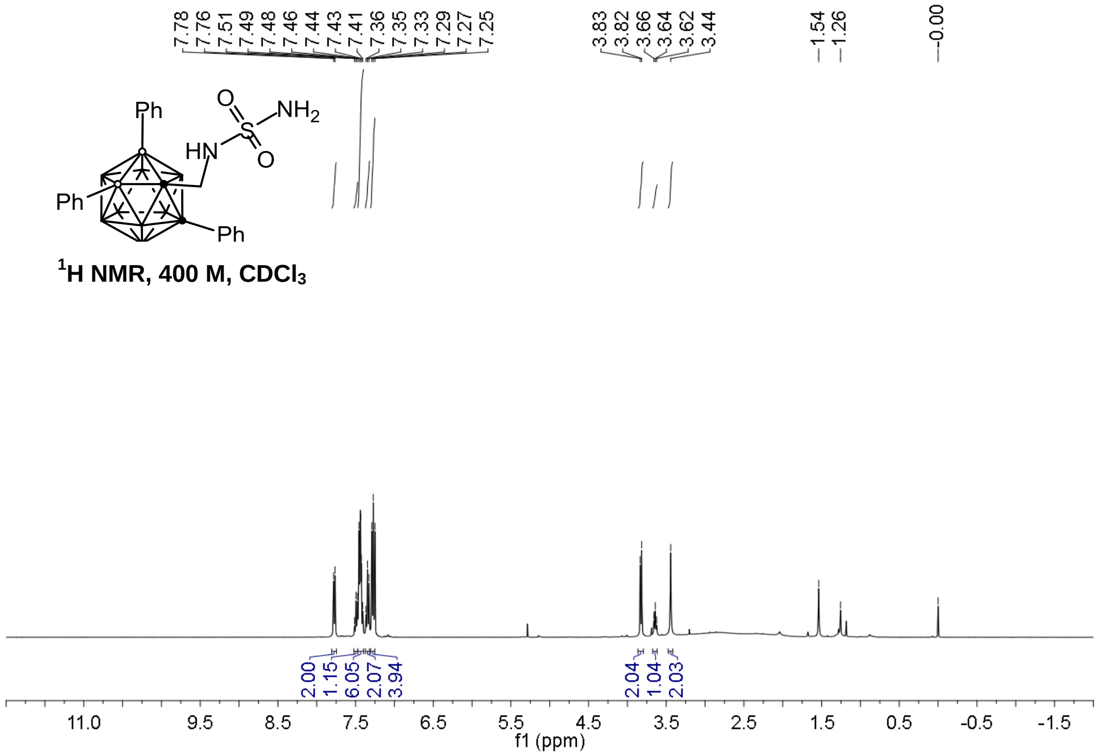

^{1}H NMR, 400 M, CDCl$_3$

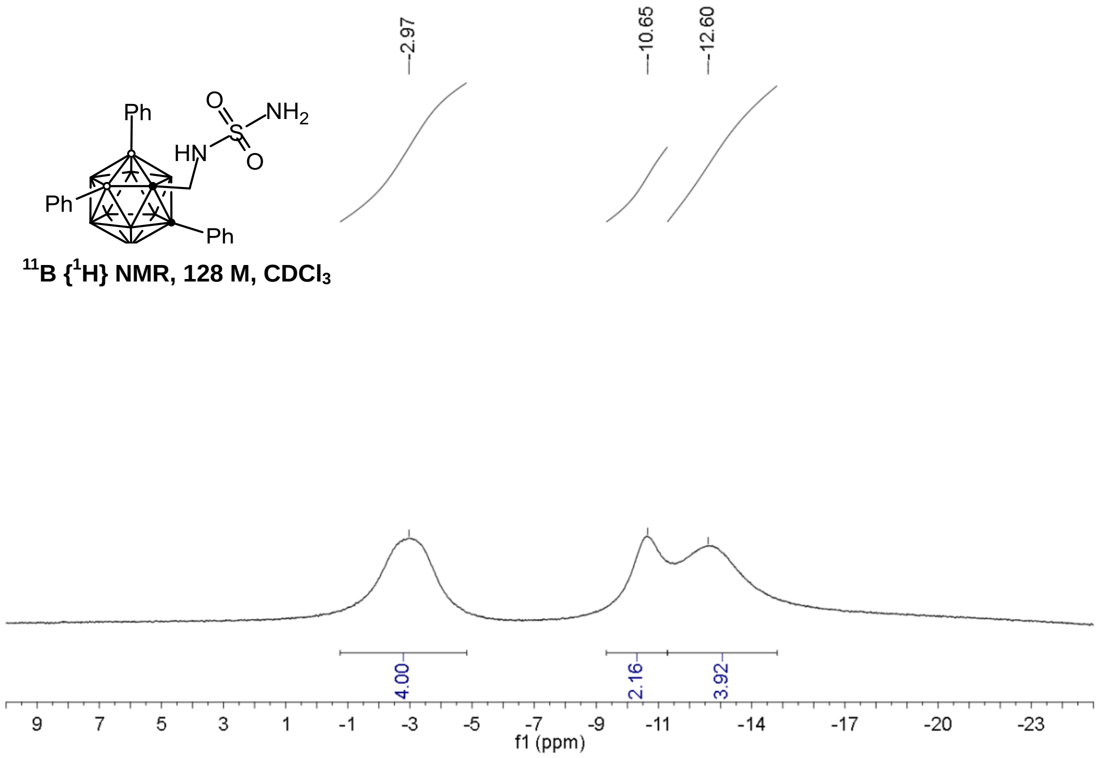

^{11}B {^{1}H} NMR, 128 M, CDCl$_3$

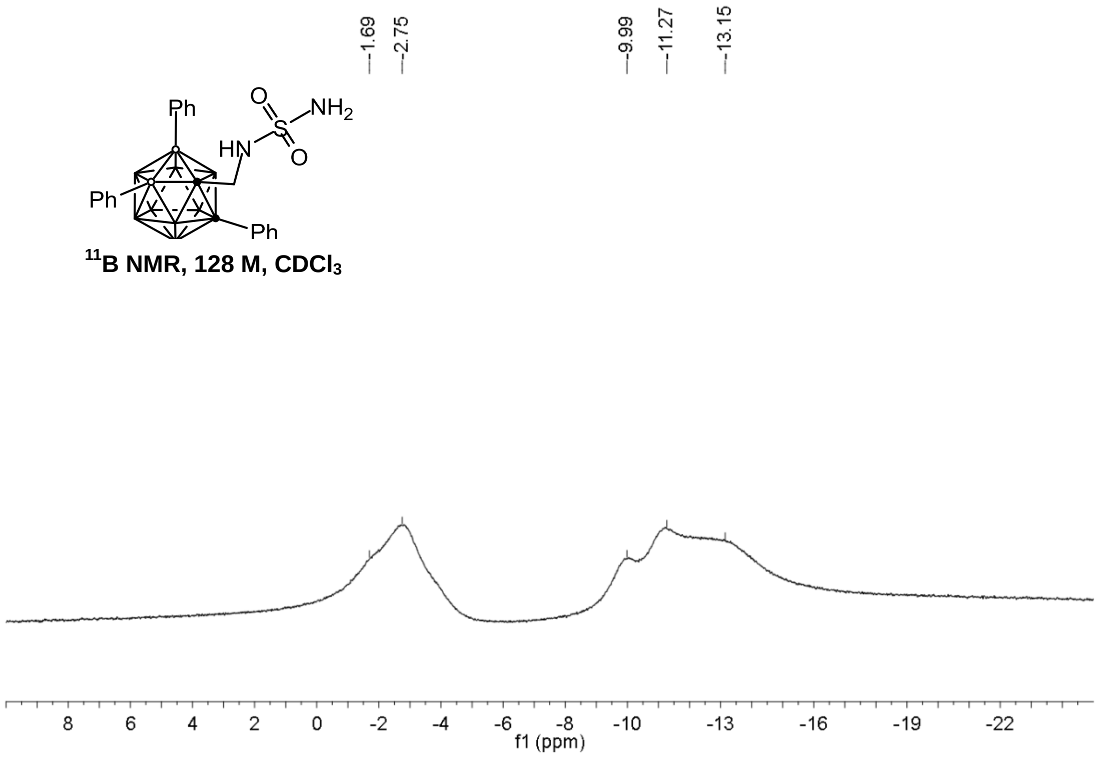
-1.69
-2.75
-9.99
-11.27
-13.15
Ph
Ph
Ph
HN
O
S
NH2
O
11B NMR, 128 M, CDCl3
8 6 4 2 0 -2 -4 -6 -8 -10 -13 -16 -19 -22
f1 (ppm)

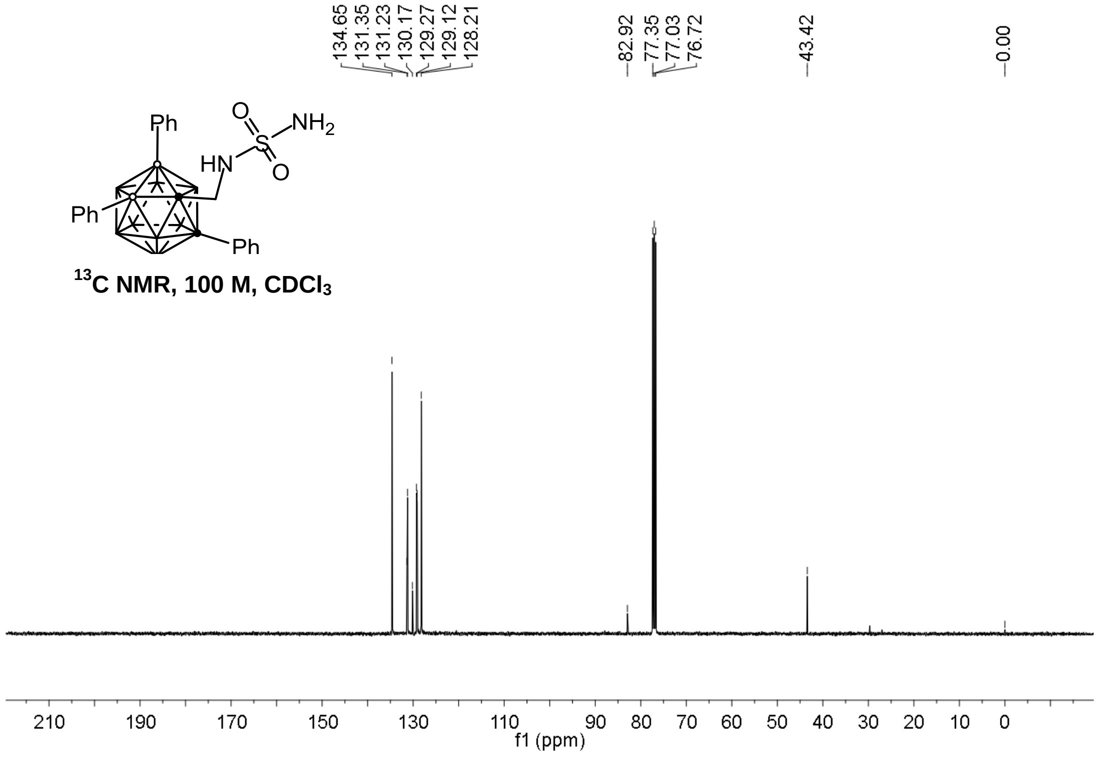
134.65
131.35
131.23
130.17
129.27
129.12
128.21
82.92
77.35
77.03
76.72
43.42
0.00
Ph
Ph
Ph
HN
O
S
NH2
O
13C NMR, 100 M, CDCl3
210 190 170 150 130 110 90 80 70 60 50 40 30 20 10 0
f1 (ppm)
